# Regulation of mammalian cellular metabolism by endogenous cyanide production

**DOI:** 10.1038/s42255-025-01225-w

**Published:** 2025-03-03

**Authors:** Karim Zuhra, Maria Petrosino, Lucia Janickova, Jovan Petric, Kelly Ascenção, Thibaut Vignane, Moustafa Khalaf, Thilo M. Philipp, Stella Ravani, Abhishek Anand, Vanessa Martins, Sidneia Santos, Serkan Erdemir, Sait Malkondu, Barbara Sitek, Taha Kelestemur, Anna Kieronska-Rudek, Tomas Majtan, Luis Filgueira, Darko Maric, Stefan Chlopicki, David Hoogewijs, György Haskó, Andreas Papapetropoulos, Brian A. Logue, Gerry R. Boss, Milos R. Filipovic, Csaba Szabo

**Affiliations:** 1 Section of Pharmacology, Department of Oncology, Microbiology and Immunology, Faculty of Science and Medicine, University of Fribourg, Fribourg, Switzerland; 2 Leibniz Institute for Analytical Sciences, Dortmund, Germany; 3 Department of Chemistry and Biochemistry, South Dakota State University, Brookings, SD USA; 4 Clinical, Experimental Surgery and Translational Research Center, Biomedical Research Foundation of the Academy of Athens, Athens, Greece; 5 Selcuk University, Science Faculty, Department of Chemistry, Konya, Turkey; 6 Giresun University, Faculty of Engineering, Department of Environmental Engineering, Giresun, Turkey; 7 Jagiellonian Centre for Experimental Therapeutics, Jagiellonian University, Krakow, Poland; 8 Department of Anesthesiology, Columbia University, New York, NY USA; 9 Section of Anatomy, Department of Oncology, Microbiology and Immunology, Faculty of Science and Medicine, University of Fribourg, Fribourg, Switzerland; 10 Department of Endocrinology, Metabolism and Cardiovascular System, Faculty of Science and Medicine, University of Fribourg, Fribourg, Switzerland; 11 Laboratory of Pharmacology, Department of Pharmacy, National and Kapodistrian University of Athens, Athens, Greece; 12 Department of Medicine, University of California, San Diego, San Diego, CA USA; 13 School of Molecular Biosciences, University of Glasgow, Glasgow, UK

**Keywords:** Cell signalling, Mitochondria

## Abstract

Small, gaseous molecules such as nitric oxide, carbon monoxide and hydrogen sulfide are produced as signalling molecules in mammalian cells. Here, we show that low concentrations of cyanide are generated endogenously in various mammalian tissues and cells. We detect cyanide in several cellular compartments of human cells and in various tissues and the blood of mice. Cyanide production is stimulated by glycine, occurs at the low pH of lysosomes and requires peroxidase activity. When generated at a specific rate, cyanide exerts stimulatory effects on mitochondrial bioenergetics, cell metabolism and cell proliferation, but impairs cellular bioenergetics at high concentrations. Cyanide can modify cysteine residues via protein *S*-cyanylation, which is detectable basally in cells and mice, and increases in response to glycine. Low-dose cyanide supplementation exhibits cytoprotective effects in hypoxia and reoxygenation models in vitro and in vivo. Conversely, pathologically elevated cyanide production in nonketotic hyperglycinaemia is detrimental to cells. Our findings indicate that cyanide should be considered part of the same group of endogenous mammalian regulatory gasotransmitters as nitric oxide, carbon monoxide and hydrogen sulfide.

## Abstract (web summary)

Zuhra and Petrosino et al. report evidence that cyanide acts as a regulatory gasotransmitter in mammalian cells, where it is shown to affect cellular bioenergetics, most likely via protein *S*-cyanylation.

## Main

Nitric oxide (NO), carbon monoxide (CO) and hydrogen sulfide (H_2_S) are small endogenous gaseous signalling molecules^1–7^. Hydrogen cyanide is recognized as an endogenous regulator in various plants and bacteria. However, in mammalian cells and tissues, it is generally regarded as a cytotoxic molecule^8^. Here we investigated whether cyanide is produced in mammalian cells and tissues and, if so, whether it serves regulatory roles.

## Enzymatic production of cyanide by mammalian cells and tissues

From a chemical standpoint, hydrogen cyanide is a weak acid (pKa = 9.2). At physiological pH, approximately 95% exists in the volatile undissociated form (HCN) and 5% in the dissociated form (cyanide, CN^−^). In this paper, we refer to both cyanide species present in biological systems as ‘cyanide’.

**Fig. 1:**
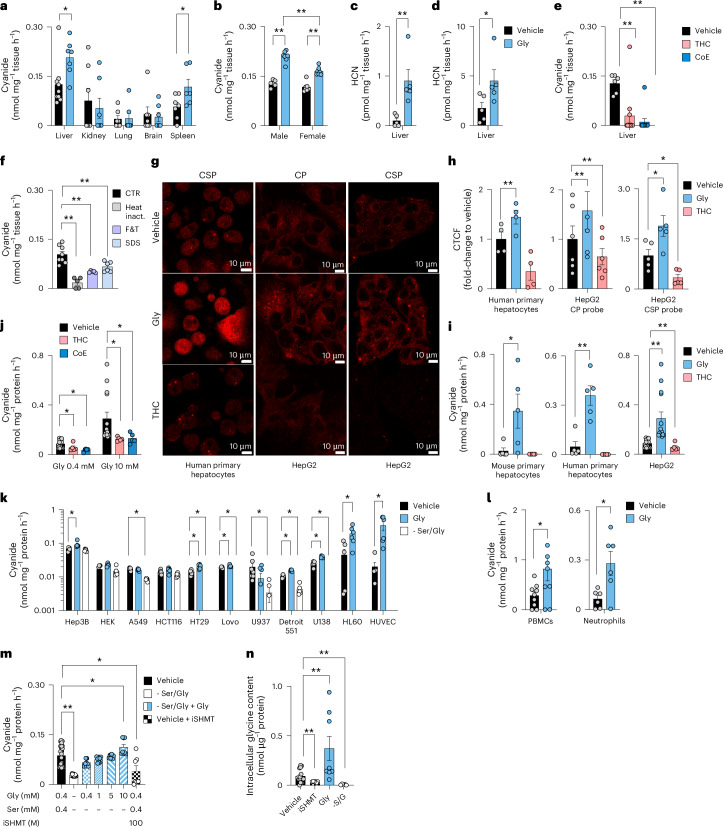
Cyanide is endogenously produced in mouse tissues and human cells. **a**–**d**, Cyanide production rates from tissue homogenates in homogenization medium containing 0.4 mM glycine (vehicle) or in medium supplemented with 10 mM glycine (Gly) were determined by the electrochemical (ECh) method after alkalinization. **a**, Comparison of cyanide generation from various tissues (at least *n* = 6 per group, biological replicates). **b**, Comparison of cyanide generation from male-versus-female mice (*n* = 6 per group, biological replicates). **c**, Detection of cyanide generation from liver homogenates using the Cyanalyzer LC–MS/MS method (*n* = 5 per group, biological replicates). **d**, Detection of cyanide generation from liver homogenates obtained from male mice using the spectrophotometric method (*n* = 5 per group, biological replicates). **e**, Treatment with HCN scavengers THC or CoE (10 µM), lowered the cyanide generation (ECh method) from mouse liver homogenates (*n* = 6 per group, biological replicates). **f**, Heat inactivation of proteins (HI), physical inactivation of proteins by multiple cycles of freezing and thawing (F&T) or SDS-induced protein denaturation (SDS) lowered the cyanide signal (ECh method) from mouse liver homogenates, compared to the control (CTR; at least *n* = 5 per group, biological replicates). **g**,**h**, Intracellular visualization (**g**) and quantification (**h**) of cyanide by confocal microscopy. Quantification of cyanide-specific signal using corrected total cell fluorescence (CTCF) values using two different cyanide-sensitive fluoroprobes Chemosensor P (CP) and a spiropyrane derivative of cyanobiphenyl (CSP) in human primary hepatocytes (*n* = 4 per group, biological replicates) and a human hepatoma line (HepG2; *n* = 6 per group or *n* = 5 per group, biological replicates, using CP or CSP probes, respectively) treated with a vehicle, 10 mM glycine (Gly) or 10 µM THC. Created with BioRender.com. **i**, Cyanide production in primary mouse and human hepatocytes and HepG2 cells treated with vehicle in standard medium containing 0.4 mM glycine (vehicle), addition of 10 mM glycine (Gly) or addition of 10 µM THC in control medium (ECh method; *n* = 4 per group biological replicates for primary human hepatocytes, *n* = 6 per group biological replicates for HepG2 cells). **j**, Effect of THC or CoE (10 µM) on the cyanide signal in HepG2 cells (ECh method; at least *n* = 6 per group, biological replicates). **k**, Cyanide production in a panel of mammalian cell lines in normal medium containing 0.4 mM glycine (vehicle), in medium supplemented with 10 mM glycine (Gly) or in −Ser/Gly medium for 24 h (ECh method; at least *n* = 5 per group, biological replicates). **l**, Cyanide production from human PBMCs and human neutrophils under basal conditions and after incubation with 10 mM glycine (Gly) for 4 h (ECh method; *n* = 6 per group, biological replicates). **m**, Cyanide production in HepG2 cells grown for 24 h in normal medium (containing 0.4 mM glycine) in the absence or presence of 100 µM SHMT inhibitor or in −Ser/Gly medium supplemented with 0.4–10 mM glycine (ECh method; at least *n* = 7 per group, biological replicates). **n**, Glycine levels in HepG2 cells under baseline conditions, after pharmacological inhibition of SHMT (iSHMT), after addition of 10 mM glycine to the culture medium or in −Ser/Gly medium for 24 h (ECh method; at least *n* = 5 per group, biological replicates). Data in **a**–**f** and **h**–**n** are expressed as the mean ± s.e.m. Data in **a**, **b**, **e**, **f** and **h**–**n** were analysed with a two-way analysis of variance (ANOVA) followed by Bonferroni’s multiple-comparisons test. Data in **c**, **d** and **l** were analysed with a two-sided Student’s *t*-test. *P < 0.05 and **P < 0.01 indicate significant differences. Source data

**Extended Data Fig. 1:**
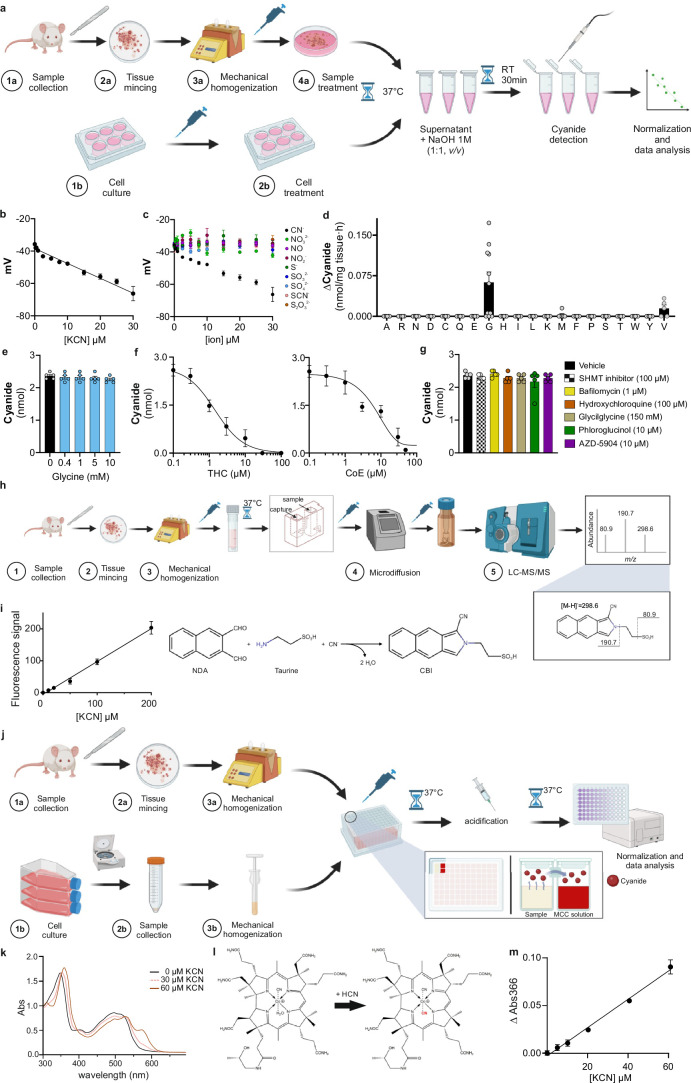
Cyanide detection methods used. The electrochemical (ECh) method **(h-i)**, the LC-MS/MS method, and **(j-m)** the monocyano-cobinamide (MCC) method of cyanide quantification. **(a)** Workflow of HCN detection using the electrochemical method (ECh) used in the current study. RT – room temperature. Created with BioRender.com. **(b)** Standard curve using potassium cyanide (KCN) prepared in 0.5 M NaOH in closed Eppendorf tubes and measured after 30 min incubation at room temperature (at least n = 5 per group, technical replicates). **(c)** Ion-selectivity of the ECh method (at least n = 3/group, technical replicates). **(d)** Increase of HCN generation as compared to the vehicle (Δ Cyanide) measured in the presence of different proteinogenic amino acids (10 mM) in mouse liver tissue (at least n = 5/group, technical replicates). **(e)** Increasing concentrations of glycine do not interfere with KCN detection (n = 5/group, technical replicates). **(f)** Decrease of free HCN levels in the presence of 0-100 µM HCN scavengers trihistidyl-cobinamide (THC) or dicobalt edetate (CoE) (at least n = 3/group, technical replicates). **(g)** The pharmacological modulators used in the study do not interfere with cyanide detection: 100 µM SHMT inhibitor, 1 µM bafilomycin, 100 µM hydroxychloroquine, 150 mM glycine-glycine, 10 µM phloroglucinol and 10 µM AZD-5904 (n = 5/group, technical replicates). **(h)** Workflow of HCN detection using LC-MS/MS. Created with BioRender.com. **(i)** Standard-curve of potassium cyanide (KCN) and reaction of HCN with 2,3-naphtalenedialdehyde (NDA) and taurine to produce the fluorescent product 1-cyano-benzoisoindole (CBI) (n = 3/group, technical replicates). **(j)** Workflow of HCN detection using the monocyano-cobinamide (MCC) method. Created with BioRender.com. **(k)** UV-Vis absorption spectra of MCC exposed to increased concentrations of HCN (provided as KCN). **(l)** Molecular structure of MCC which, in the presence of HCN, is converted to dicyano-cobinamide. **(m)** Standard curve of potassium cyanide (KCN) using the MCC method (n = 2/group, technical replicates). Data in **c, d, e, f, g, m** are expressed as the mean ± s.e.m. Source data

The cyanide-selective electrode method^9^ is based on trapping volatile cyanide in solution via alkalization and subsequent selective detection of CN^−^. Using this technique, substantial cyanide generation was detected from gently homogenized mouse tissues, with liver producing the highest amounts (Fig. 1a). Next, we tested whether amino acids could stimulate cyanide generation. Adding glycine to liver or spleen homogenates increased cyanide generation (Fig. 1a and Extended Data Fig. 1a–e), while none of the other 19 proteinogenic amino acids had such an effect (Extended Data Fig. 1e). Basal and glycine-stimulated cyanide generation were observed in both male and female liver homogenates, with glycine inducing more cyanide generation from livers of male mice than from livers of female mice (Fig. 1b). We confirmed the glycine-mediated stimulation of cyanide generation in liver homogenates using two additional methods that measure volatile HCN in the gaseous form: a liquid chromatography with tandem mass spectrometry (LC–MS/MS) method where HCN is trapped in a chamber containing naphthalene dialdehyde and taurine^10^ (Fig. 1c and Extended Data Fig. 1h,i), and a spectroscopic method, where HCN is captured by the cyanide scavenger monocyano-cobinamide (MCC), followed by a colour change^11^ (Fig. 1d and Extended Data Fig. 1j–m). The specificity of the cyanide signal was confirmed using the cyanide scavengers trihistidyl-cobinamide (THC) and dicobalt edetate (CoE)^12,13^ (Fig. 1e and Extended Data Fig. 1f).

Having shown that glycine stimulates mammalian cyanide generation, we sought to determine if this process is enzymatically regulated. We found that cyanide generation was reduced by several different methods that denature proteins in the tissue homogenate, suggesting the involvement of an enzymatic process (Fig. 1f).

**Extended Data Fig. 2:**
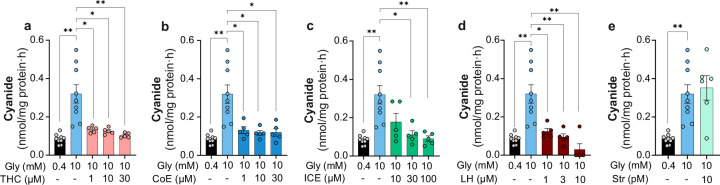
Modulation of HCN levels in HepG2 cells. Increasing concentrations of **(a)** the HCN scavenger trihistidyl-cobinamide (THC, 0-30 µM) (at least n = 5/group, biological replicates), **(b)** the HCN scavenger dicobalt edetate (CoE, 0-30 µM) (at least n = 5/group, biological replicates), **(c)** the glycine transporter GlyT-1 inhibitor iclepertin (ICE 10-100 µM) (at least n = 5/group, biological replicates), and **(d)** the SHMT inhibitor lometrexol hydrate (LH, 1-10 µM) (at least n = 5/group, biological replicates) abrogate the glycine-induced increase in HCN signal from HepG2 cells, as measured by electrochemical method. **(e)** The glycine receptor antagonist strychnine (Str) does not affect glycine-stimulated cyanide generation (at least n = 6/group, biological replicates). Data in **a**, **b**, **c**, **d**, **e**, are expressed as the mean ± s.e.m. Data were analysed with a two-way ANOVA followed by Bonferroni’s multiple-comparisons test. *p < 0.05 and **p < 0.01 indicate significant differences. Source data

Human and mouse primary hepatocytes and the human hepatoma cell line HepG2 also produced cyanide, when assayed in standard culture medium containing 400 µM glycine (Fig. 1g–i). The cyanide signal increased by glycine supplementation and was reduced by the cyanide scavenger THC, as shown by confocal microscopy using two structurally different fluorescent cyanide probes^14,15^ (Fig. 1g,h) and also quantified by the electrochemical method (Fig. 1i,j and Extended Data Fig. 2). Cyanide generation was detectable from various cultured human cell lines including those of lung and colonic epithelial and myeloid lineage; the cells responded to glycine supplementation or deprivation with increased or decreased cyanide production, respectively (Fig. 1k). Cyanide production was also detectable in human peripheral blood mononuclear cells (PBMCs) and neutrophils, and was further stimulated by glycine (Fig. 1l). Of the parenchymal cells investigated, cells of hepatic origin—including primary hepatocytes and human hepatoma lines—exhibited the highest rates of cyanide production (Fig. 1h–l).

We selected the HepG2 cells for subsequent studies. Cyanide generation was suppressed when HepG2 cells were grown in serine/glycine-free (−Ser/Gly) medium; reconstituting glycine restored cyanide generation (Fig. 1m). The clinically used drug iclepertin, which inhibits the glycine transporter GlyT-1/SLC6A9 on the cell membrane, inhibited cyanide generation (Extended Data Fig. 2c). Cyanide production was also attenuated by inhibition of serine hydroxymethyltransferase (SHMT)^16^, the enzyme which interconverts glycine and serine (Fig. 1m and Extended Data Fig. 2d). Measurement of intracellular glycine concentrations (Fig. 1n) confirmed the modulation of intracellular glycine concentrations by the above interventions. Glycine-stimulated cyanide generation was not inhibited by glycine receptor blockade with its antagonist strychnine, suggesting that cyanide production occurs intracellularly and is not related to glycine receptors on the cell membrane (Extended Data Fig. 2e).

## Cyanide production occurs primarily in the lysosomes

**Fig. 2:**
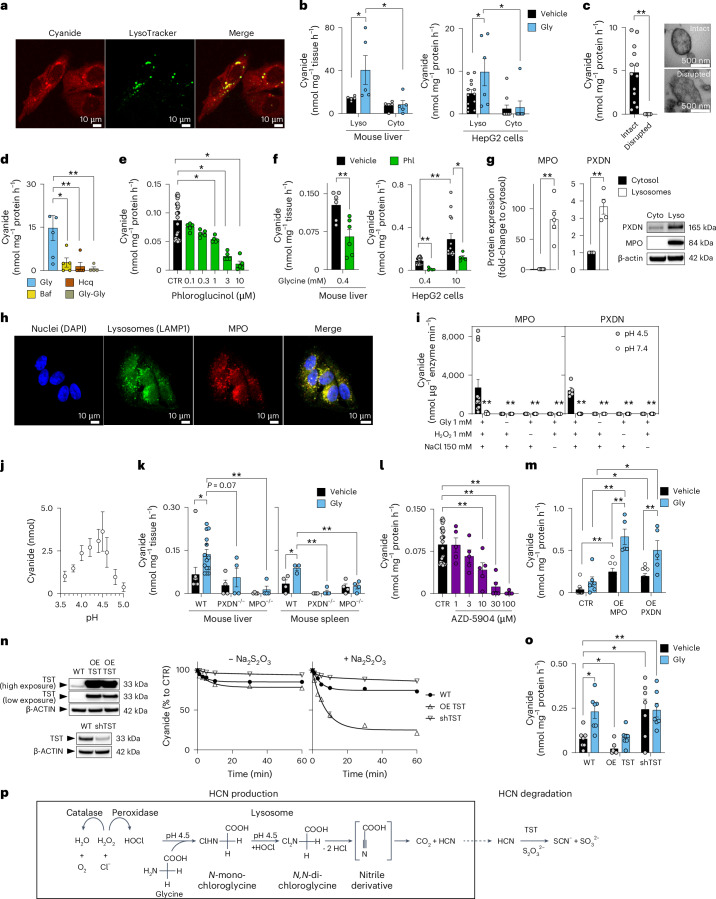
Cyanide is enzymatically generated by lysosomal peroxidases. **a**, The cyanide signal in HepG2 cells partially colocalizes with lysosomes (confocal microscopy using Chemosensor P). Images shown are representative of *n* = 3 biological replicates per group. **b**,**c**, Cyanide generation in lysosomal and cytosolic fractions (Lyso and Cyto, respectively) obtained from mouse liver or HepG2 cells ± 10 mM glycine (Gly; ECh method; at least *n* = 5 per group, biological replicates; **b**) or from isolated intact versus disrupted lysosomes (ECh method; at least *n* = 5 per group, biological replicates; **c**) as visually confirmed by electron microscopy. **d**, Cyanide generation in isolated lysosomes after treatment with 1 µM bafilomycin (Baf), 30 µM hydroxychloroquine (Hcq) or 150 mM glycylglycine dipeptide (Gly-Gly; ECh method; *n* = 5 per group, biological replicates). **e**, Cyanide detection in HepG2 cells with 0.1–10 µM phloroglucinol (Phl; ECh method; at least *n* = 5 per group, biological replicates). **f**, Cyanide detection from isolated lysosomes obtained from mouse liver homogenates incubated with 0.4 mM glycine in the absence or presence of 10 µM Phl (left) or HepG2 cells incubated with 0.4 mM or 10 mM glycine in the absence or presence of 10 µM Phl (ECh method; at least *n* = 5 per group, biological replicates). **g**, MPO and PXDN expression in lysosomal (L) and cytosolic (C) fractions of HepG2 cells (*n* = 5 biological replicates for MPO and *n* = 4 biological replicates for PXDN). **h**, Confocal microscopy of MPO localization in lysosomes. Nuclei were chemically stained using DAPI, while lysosomes and MPO were immunohistochemically detected using LAMP1 and MPO antibodies, respectively. Images shown are representative of *n* = 3 biological replicates per group. **i**, MPO or PXDN catalyse cyanide generation at pH 4.5 (enzyme was incubated with various combinations of 1 mM glycine, 1 mM H_2_O_2_ and 150 mM NaCl; ECh method; at least *n* = 5 per group, technical replicates). **j**, Determination of an optimal pH for cyanide generation using equimolar concentrations of HOCl and glycine (optimum pH 4.5; ECh method; *n* = 9 per group, technical replicates). **k**, Cyanide production in liver and spleen homogenates of WT, PXDN^+/−^ and MPO^−/−^ male mice under baseline conditions and after the addition of 10 mM glycine (Gly; ECh method; at least *n* = 4 per group, biological replicates). **l**, Detection of cyanide in HepG2 cells treated with 1–100 µM MPO inhibitor AZD-5904 (ECh method; at least *n* = 5 per group, biological replicates). **m**, Detection of cyanide in HEK293T cells overexpressing (OE) MPO or PXDN in the absence or presence of an additional 10 mM glycine (ECh method; at least *n* = 5 per group, biological replicates). **n**, Impact of overexpression of human rhodanese (OE-TST, cells from two different passages) or its downregulation (shTST) in HepG2 cells (as confirmed by western blots) on cellular capacity to degrade exogenously administered 100 µM KCN in the absence or presence of 1 mM sodium thiosulfate (ECh method; *n* = 5 per group, biological replicates). **o**, Overexpression of human rhodanese (OE-TST) or its downregulation (shTST) in HepG2 cells resulted in the reduction or accumulation, respectively, of endogenous cyanide in HepG2 cells (ECh method; *n* = 7 per group, biological replicates). Data in **b**–**g**, **i**, **j**–**m** and **o** are expressed as the mean ± s.e.m. Data in **b**–**f**, **i** and **k**–**o** were analysed with a two-way ANOVA followed by Bonferroni’s multiple-comparisons test. Data in **c** and **g** were analysed with a two-sided Student’s *t*-test. *P < 0.05 and **P < 0.01 indicate significant differences. **p**, Our proposed scheme of lysosomal cyanide generation. In lysosomes, at pH 4.5, glycine undergoes a two-step chlorination reaction in the presence of peroxidase-derived HOCl. The subsequent hydrolysis of *N*,*N*-dichloroglycine leads to the formation of an unstable nitrile derivative intermediate, which spontaneously decomposes to carbon dioxide (CO_2_) and hydrogen cyanide (HCN). HCN, in turn, is converted to SCN^−^ and CO_2_ via rhodanese/TST using thiosulfate in the extra-lysosomal cell compartment. Source data

**Extended Data Fig. 3:**
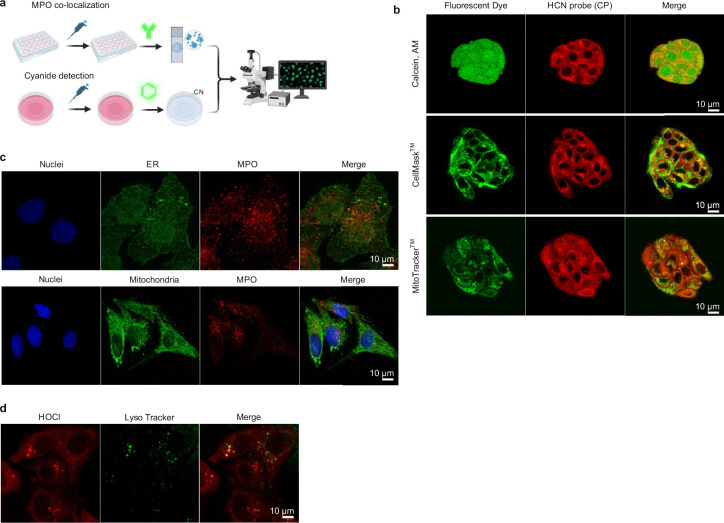
Confocal microscopy analysis of cyanide and HOCl generation in HepG2 cells. **(a)** Workflow of HCN detection using confocal microscopy. MPO – myeloperoxidase. Created with BioRender.com. **(b)** Confocal microscopy of HepG2 cells incubated with 10 µM selective HCN probe for 1 h incubation together with other cell-permeant dyes (10 µM Calcein AM, 1 µM CellMask Green Actin Tracking Stain or 200 nM MitoTracker Deep Red FM) for 30 minutes at 37 °C and 5% CO_2_. At the end of the incubation, cells were washed 3x and visualized using confocal microscope. Following excitation and emission spectra were used: HCN probe (Ex405/Em584-620 nm), Calcein AM (Ex488/Em517 nm), CellMask Plasma Membrane Stain (Ex488/Em535 nm) MitoTracker Deep Red FM (Ex644/Em665 nm). Images shown are representative of n = 3 biological replicates/group. **(c)** Co-localization of MPO with various subcellular organelles. Representative confocal microscopy images of HepG2 showing that MPO does not localize to the endoplasmic reticulum (ER) nor mitochondria. ER was visualized by using 500 nM ER tracker green. Primary anti-MPO antibody (mouse, dilution 1:10,000, Sigma-Aldrich) followed by incubation with appropriate secondary antibody goat anti-rabbit IgG (H + L) Highly Cross-Adsorbed Secondary Antibody Alexa Fluor Plus 647 (1:1,000 dilution) was used for detection of MPO. Mitochondria were visualized by using 200 nM MitoTracker Deep Red FM stain, while cellular nuclei were stained by 5 µg/ml DAPI. Images were collected at Leica 8 STELLARIS Falcon using 63x magnification. For co-localization with ER, ER tracker was visualized at Ex504/Em511 nm and MPO at Ex647/Em665 nm. For co-localization with mitochondria, MitoTracker was visualized at Ex647/Em665 nm and MPO at Ex568/Em603 nm. Images shown are representative of n = 3 biological replicates/group. **(d)** Co-localization of HOCl with lysosomes. Representative confocal microscopy image of HepG2 showing that HOCl localizes with lysosomes. HepG2 cells were loaded with 10 µM Chemosensor P in HBSS for 1 h together with 50 nM LysoTrackerGreen for 30 min at 37 °C and 5% CO_2_. At the end of the incubation, cells were washed 3x and visualized using confocal microscope. Following excitation and emission spectra were used: HOCl probe (Ex405/Em450-550 nm) and LysoTrackerGreen Ex488/Em517 nm. Images shown are representative of n = 3 biological replicates/group. Source data

Confocal live cell imaging revealed that cyanide is present throughout the cell, as expected for a diffusible molecule (Fig. 1g). Thus, cyanide was detected in the cytosol—as evidenced by partial colocalization with calcein AM, and in mitochondria—as evidenced by partial colocalization with the mitochondrial marker MitoTracker (Extended Data Fig. 3a,b). However, the strongest cyanide signal was detected in the lysosomes—as evidenced by colocalization with LysoTracker (Fig. 2a).

**Extended Data Fig. 4:**
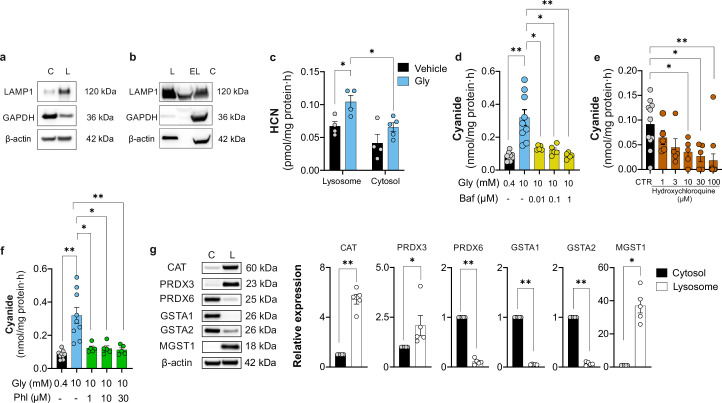
Mechanisms of lysosomal HCN production. **(a)** Western blot of cytosolic (C) *vs* lysosomal (L) fractions from HepG2 cells. LAMP-1: lysosomal associated membrane protein 1; GAPDH: glyceraldehyde 3-phosphate dehydrogenase. **(b)** Western blot of lysosomal (L), extra-lysosomal (EL) and cytosolic (C) fractions from mouse liver. **(c)** HCN detection in the lysosomal *vs* cytosolic fractions of HepG2 cells, with or without incubation with glycine (10 mM), as measured with the LC-MS/MS method (at least n = 4, biological replicates). **(d)** Inhibitory effect of the lysosomal proton pump inhibitor bafilomycin (Baf, 0.1- 1 µM) on cyanide production in HepG2 cells in the presence of increasing concentration (10 mM) of glycine (Gly) (at least n = 4/group, biological replicates). **(e)** Inhibitory effect of the lysosomal deacidifier hydroxychloroquine (1-100 µM) on cyanide production in HepG2 cells (at least n = 4/group, biological replicates). **(f)** Inhibitory effect of the peroxidase inhibitor phloroglucinol (Phl, 1- 30 µM) on cyanide production in HepG2 cells (at least n = 4/group, biological replicates). **(g)** Western blots of the expression of catalase and various peroxidases – catalase, peroxiredoxin3 (PRDX3), peroxiredoxin6 (PRDX6), glutathione S-transferase alpha1 (GSTA1), glutathione S-transferase alpha2 (GSTA2), and microsomal glutathione S-transferase1 (MGST1) – in cytosolic (C) vs. lysosomal (L) fractions of HepG2 cells, including densitometric quantification of their relative expression (n = 5/group, biological replicates). Data in **c**, **d**, **e**, **f**, **g**, are expressed as the mean ± s.e.m. Data in **c**, **d**, **e**, **f** were analysed with a two-way ANOVA followed by Bonferroni’s multiple-comparisons test. Data in **g** were analysed with a two-sided Student’s t-test. *p < 0.05 and **p < 0.01 indicate significant differences. Source data

Because the cyanide signal was most prominent in the lysosomes, we next compared the cyanide-generating capacity of isolated lysosomal-versus-cytosolic fractions obtained from liver tissue and HepG2 cells. Higher basal cyanide was detected in the lysosomal fraction than in the cytosolic fraction; adding glycine selectively increased cyanide generation in the lysosomal—but not the cytosolic—fraction (Fig. 2b and Extended Data Fig. 4a–d). Electron microscopy confirmed that our standard homogenization protocol preserved the structural integrity of subcellular organelles, including lysosomes. A more drastic homogenization protocol, which disrupted the integrity of the lysosomes, abrogated the ability of glycine to stimulate cyanide generation (Fig. 2c), suggesting that lysosomal integrity is essential for mammalian cyanide generation.

We confirmed the importance of functional lysosomes for cyanide generation by testing the effect of the lysosomal proton pump inhibitor bafilomycin^17^, the lysosomal alkalinizer hydroxychloroquine (a lipophilic and lysosomotropic drug, which penetrates cell membranes, and accumulates in the acidic lysosomes and, as a consequence, increases the pH in lysosomes from the normal values of 4.7–4.8 to 6)^18^ and Gly-Gly dipeptide, which inhibits the lysosomal glycine transporter LYAAT-1 (ref. ^19^). All of these pharmacological agents suppressed cyanide generation in isolated lysosomes (Fig. 2d). The inhibitory effect of bafilomycin and hydroxychloroquine on cyanide generation was also confirmed in HepG2 cells (Extended Data Fig. 4d,e).

## Cyanide production requires peroxidase activity

Enzymatic conversion of glycine to cyanide would require oxidation of glycine’s α-amino group to a nitrile group, a reaction that hydrolytic enzymes, the primary constituents of lysosomes, cannot catalyse. However, peroxidases, which generate strong oxidants, such as hypochlorous acid (HOCl), may catalyse glycine’s oxidation. Therefore, we tested the effect of the broad-spectrum peroxidase inhibitor phloroglucinol^20^ in liver homogenates and HepG2 cells and found that it exerts a concentration-dependent inhibitory effect on cyanide production (Fig. 2e,f and Extended Data Fig. 4f). As in most cells^21^, multiple peroxidases were detected in HepG2 cells, with myeloperoxidase (MPO) and peroxidasin (PXDN) exhibiting preferential lysosomal localization (Fig. 2g and Extended Data Fig. 4g). Confocal microscopy confirmed MPO localization to the lysosomes, but not to the endoplasmic reticulum or mitochondria (Fig. 2h and Extended Data Fig. 3c).

**Fig. 3:**
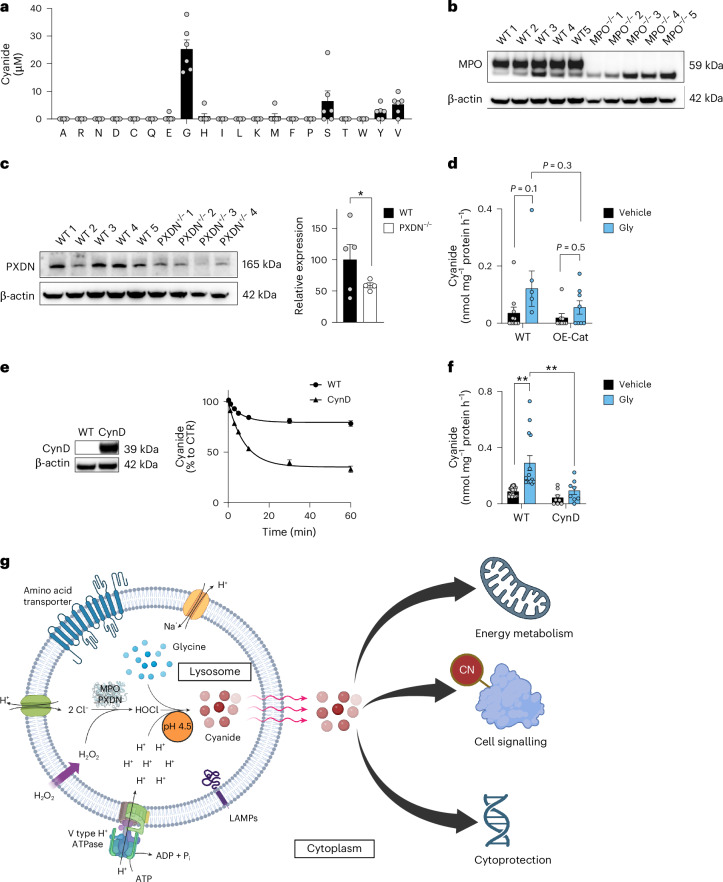
HOCl-catalysed lysosomal HCN generation. **a**, HCN generation after mixing equimolar amounts of HOCl and different proteinogenic amino acids at pH 4.5 in 50 mM sodium citrate buffer as quantified by the ECh method (*n* = 6 per group, biological replicates). **b**, Western blot analysis of MPO expression in liver homogenates from MPO^−/−^ male mice compared to WT male controls. No quantification was performed for MPO expression due to no detectable protein band in the MPO^−/−^ samples. **c**, Western blot analysis of PXDN expression in liver homogenates from PXDN^+/−^ male mice compared to WT male controls, followed by the densitometric quantification of PDXN expression (at least *n* = 4 per group, biological replicates). **d**, HCN generation in HEK293T cells (WT) and HEK293T cells overexpressing catalase (OE-CAT) in the absence or presence of 10 mM glycine by using the ECh method (at least *n* = 5 per group, biological replicates). **e**, Decomposition of exogenously supplied potassium cyanide by HepG2 cells (WT) and HepG2 cells overexpressing CynD (*n* = 10 per group, biological replicates). **f**, Cyanide production in HepG2 cells (WT) and HepG2 cells overexpressing CynD (CynD) in the absence and presence of 10 mM glycine (at least *n* = 8 per group, biological replicates). **g**, Proposed mechanism and consequences of cyanide generation in mammalian cells. Lysosomal peroxidases, mainly MPO and PXDN, catalyse the production of HOCl from H_2_O_2_ and Cl^−^. At physiological lysosomal pH 4.5, glycine is chlorinated by HOCl to generate *N*,*N*-dichloroglycine, which spontaneously decomposes into cyanide, CO_2_ and HCl. Cyanide diffuses through the lysosomal membrane to the cytosol where it acts as a signalling molecule (in part through *S*-cyanylation of target proteins), directly stimulates bioenergetics and provides cytoprotective effects. Data in **a**, **c**, **d** and **f** are expressed as the mean ± s.e.m. Data in **a**, **d** and **f** were analysed with a two-way ANOVA followed by Bonferroni’s multiple-comparisons test. Data in **c** were analysed with a two-sided Student’s *t*-test. *P < 0.05 and **P < 0.01 indicate significant differences. Source data

Because mammalian cyanide production (i) requires glycine, (ii) occurs in the acidic pH of lysosomes and (iii) is peroxidase dependent, we hypothesized that cyanide generation is dependent on the peroxidase product HOCl, which is predominantly produced in lysosomes^22^. Indeed, we found that HOCl mainly localized to the lysosomes, and to a lesser extent to the cytosol of HepG2 cells (Extended Data Fig. 3d). Therefore, we conducted in vitro biochemical experiments, where we incubated MPO or PXDN enzyme with glycine, hydrogen peroxide (H_2_O_2_) and chloride (Cl^−^), and observed cyanide production with an optimum pH of 4.5 (Fig. 2i). Additionally, when glycine was incubated with HOCl in the absence of any enzyme, cyanide was also produced with the same optimum pH of 4.5 (Fig. 2j). Adding other proteinogenic amino acids did not yield significant amounts of cyanide under the same conditions (Fig. 3a).

Liver and spleen homogenates obtained from MPO^−/−^ or PXDN^+/−^ mice generated less cyanide than homogenates from wild-type (WT) animals (Figs. 2k and 3b,c). The MPO inhibitor AZD-5904 (ref. ^23^) also inhibited cyanide generation in a concentration-dependent manner (Fig. 2l). Overexpression of MPO or PXDN in HEK293 cells markedly increased cyanide production (Fig. 2m), while overexpression of catalase exerted an inhibitory effect (Fig. 3d). These data indicate that peroxidase-catalysed glycine oxidation in lysosomes is the predominant mechanism of endogenous cyanide generation in mammalian cells (Fig. 3g).

Thiosulfate sulfurtransferase (TST, also known as rhodanese) is considered the main cyanide detoxification enzyme in eukaryotes^24^. Its overexpression in HepG2 cells increased the cells’ ability to decompose exogenously added cyanide (applied as the salt form, KCN, to the cells; Fig. 2n) and decreased endogenous cyanide concentrations in HepG2 cells (Fig. 2o). Similar effects were observed when the bacterial cyanide degradation enzyme cyanide dihydratase (CynD)^25^ was overexpressed (Fig. 3e,f). Conversely, knockdown of the *TST* gene (shTST) resulted in reduced KCN degradation rates (Fig. 2n) and increased endogenous cyanide levels in HepG2 cells (Fig. 2o).

Based on all these results, combined with prior biochemical findings^26^, we propose the following model of mammalian cyanide generation (Fig. 2p): peroxidases—MPO, PXDN and possibly others—produce HOCl, using H_2_O_2_ and chloride as substrates; HOCl, in the acidic milieu of the lysosome, reacts with glycine, yielding *N*-monochloroglycine, which undergoes acid-catalysed conversion to *N*,*N*-dichloroglycine. The latter molecule decomposes to the corresponding nitrile, cyanocarboxylic acid (CN-COOH), releasing hydrogen cyanide and CO_2_. Due to its gaseous properties, cyanide can exit the lysosome and enter the cytosol to reach various cellular components, and may also diffuse out of the cell and act as a paracrine mediator (Fig. 3g).

## Endogenously generated cyanide induces protein cyanylation

**Fig. 4:**
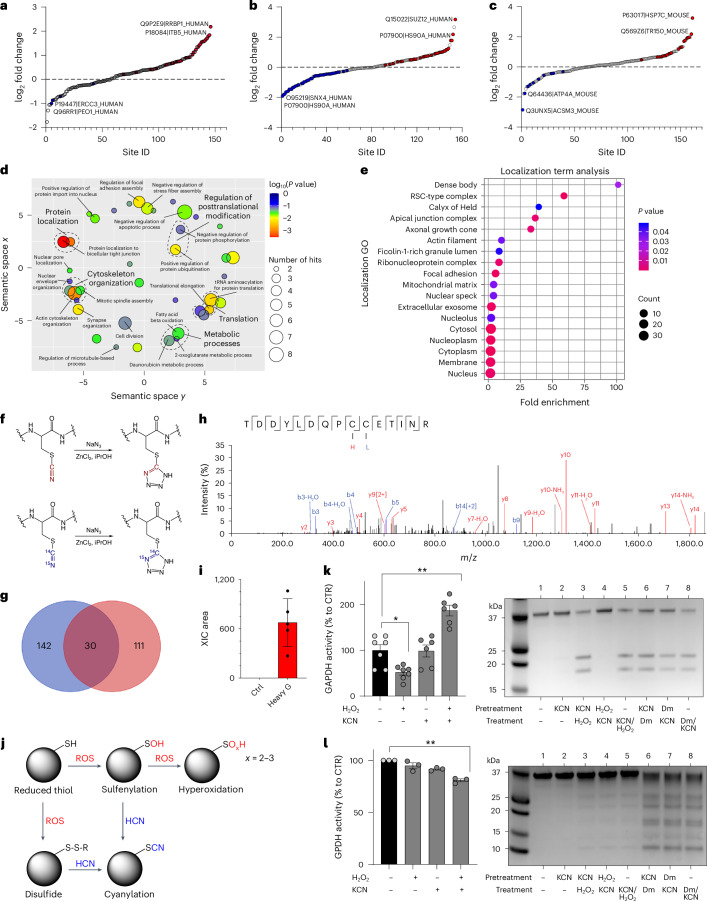
Cyanide induces posttranslational protein modifications. **a**–**c**, Proteome-wide and site-specific changes in *S*-cyanylation in HepG2 cells treated with 1 µM KCN (**a**), HepG2 cells treated with 10 mM glycine (Gly; **b**) and mouse liver tissue lysates treated with 10 mM Gly (**c**; *n* = 5 per group, biological replicates). **d**, Gene Ontology (GO) term enrichment (biological process) of the proteins whose S-cyanylation is significantly increased in HepG2 cells treated with Gly and cyanide releasers and decreased in cells cultured in glycine/serine-free medium. Using DAVID for enrichment, the outcomes were visualized through REVIGO. Significant GO terms passed the Benjamini-adjusted *P*-value threshold of 0.01. Circle dimensions denote the protein count within specific GO terms, while colour gradients communicate the degree of significance. **e**, GO term enrichment analysis (cellular localization) of cyanylated proteins. **f**, Zn^2+^-catalysed transformation of cyanylated peptides to light and heavy tetrazole, used to increase specificity of detected modifications. **g**, Venn diagram comparing the proteins found to contain ^13^C^15^N (heavy cyano) cyanylation with the endogenously cyanylated proteins (light cyano) in liver tissue lysates treated with ^13^C^15^N-labelled Gly (*n* = 5, biological replicates). **h**,**i**, Annotated MS/MS spectrum of peptides from Rab GDP dissociation inhibitor beta (UniProt accession: Q61598) displaying two cysteine sites—C203 labelled with light tetrazole (blue L) and C202 labelled with heavy tetrazole (red H; **h**)—and corresponding quantification of extracted ion chromatogram (XIC) area (*n* = 5 per group, biological replicates; **i**). **j**, Proposed scheme of protein S-cyanylation. After reaction with reactive oxygen species (ROS), thiols (RSH) become oxidized to either sulfenic acid (RSOH) or disulfides (RSSR), which could be both intramolecular and intermolecular disulfides. Both ROSH and RSSR could react with HCN leading to protein cyanylation (RSCN). When SH groups are hyperoxidized, they are no longer reactive to cyanide. **k**, Left, Enzymatic activity of GAPDH pre-incubated with H_2_O_2_ (10 µM), KCN (10 µM) or H_2_O_2_/KCN (at least *n* = 6 per group, biological replicates). Right, Detection of high-pH-induced peptide bond cleavage at cyanylation sites of GAPDH that was treated with a different combination of KCN, H_2_O_2_ or diamide (Dm; SDS–PAGE analysis). **l**, Left, Enzymatic activity of GPDH pre-incubated with H_2_O_2_ (10 µM), KCN (10 µM) or H_2_O_2_/KCN (*n* = 3 per group, biological replicates). Right, Detection of high-pH-induced peptide bond cleavage at cyanylation sites of GPDH that was treated with a different combination of KCN, H_2_O_2_ or diamide (Dm; SDS–PAGE analysis). Data in **i**, **k** and **l** are expressed as the mean ± s.e.m. Data in **k** and **l** were analysed with a two-way ANOVA followed by Bonferroni’s multiple-comparisons test. *P < 0.05 and **P < 0.01 indicate significant differences. Source data

**Extended Data Fig. 5:**
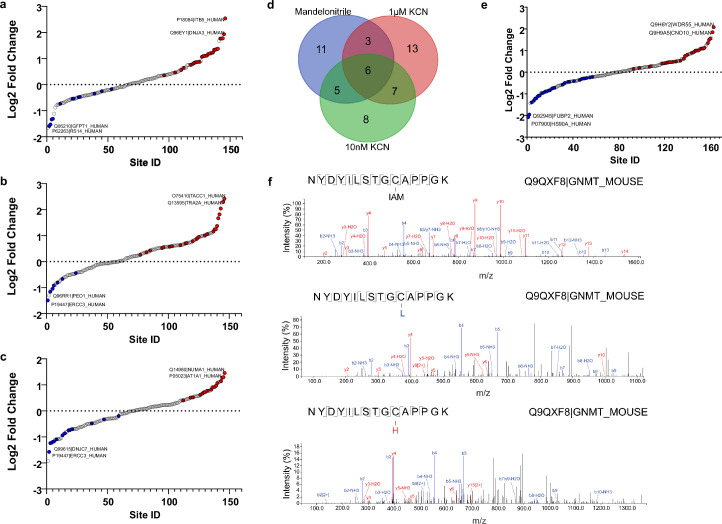
S-cyanylome remodeling caused by glycine or various cyanide donors. The proteome-wide and site-specific changes in *S*-cyanylation in **(a)** HepG2 cells treated with 10 nM KCN, **(b)** 100 µM mandelonitrile and **(c)** 100 µM amygdalin. N = 5/group, biological replicates. Colored dots represent cyanylated peptides found to be statistically significantly affected by the treatment; p < 0.05. **(d)** Venn diagram of proteins whose S-cyanylation was found to be significantly increased in HepG2 cells treated with 1 nM KCN, 10 nM KCN, and 100 µM mandelonitrile. **(e)** Proteome-wide and site-specific changes in *S*-cyanylation in HepG2 cells grown in glycine/serine free medium. **(f)** Annotated MS/MS spectra of peptide from glycine N-methyltransferase (UniProt accession: Q9QXF8) with C186 site either as carbamidomethylated (IAM) or containing light (blue L) or heavy (red H) tetrazole. Source data

Posttranslational modifications (PTMs) of protein cysteine residues include cysteine *S*-nitrosylation by NO or cysteine persulfidation by H_2_S^1–7^. Cyanylation (RSCN), the addition of a CN group to the sulfur atom in cysteine residues, has been recently described in plants and was suggested to affect protein functions^27^. To test if protein cyanylation is also present in mammalian cells and if it could be increased by adding exogenous cyanide, we next incubated HepG2 cells with KCN or various cyanide-releasing compounds. At 1 µM, KCN significantly increased cyanylation of 30 sites (Fig. 4a) and even a lower concentration of KCN (10 nM) elicited a similar response (Extended Data Fig. 5a). The organic cyanide donors amygdaline and mandelonitrile also increased cyanylation of ~25 sites (Extended Data Fig. 5b–d).

Next, to test for the presence of endogenous protein cyanylation in mammalian cells and tissues, we evaluated proteins from mouse liver and HepG2 cells for evidence of cyanylation. In the absence of a chemoselective method to assess cyanylation, we analysed proteome data for loss of a hydrogen atom and addition of a CN group (*m/z* + 24.995249 Da). We lysed tissues or cells in the presence of iodoacetamide to block all available thiols and found cyanylation of 161 cysteines on 143 proteins in mouse liver under baseline conditions and 163 cysteines on 146 proteins in HepG2 cells (Fig. 4b). To determine if cyanylation is also endogenously regulated by glycine, we incubated HepG2 cells with 10 mM glycine and observed increased cyanylation at 33 sites on 33 proteins (Fig. 4b and Supplementary Fig. 1). Glycine also increased protein cyanylation in mouse liver lysates (Fig. 4c and Supplementary Fig. 2).

Depriving cells of serine and glycine decreased cyanylation at 42 cysteine sites (Extended Data Fig. 5e). All of the affected proteins are involved in metabolic processes (for example, as fatty acid beta oxidation), regulation of PTMs (for example, as phosphorylation and ubiquitination), cytoskeleton organization, protein localization and protein translation (Fig. 4d). Cyanylated proteins were spread throughout cells and could be found in all cellular compartments in accordance with the diffusible nature of HCN (Fig. 4e). Interestingly, the most enriched compartment comprised dense bodies (that is, late-stage lysosomes, which often accumulate undigested materials over time). Furthermore, ficolin-1-rich granules, rich in MPO, also formed a compartment enriched for cyanylation.

To further highlight the importance of glycine as an endogenous source for HCN and protein cyanylation, we incubated mouse liver lysates with heavy glycine (^13^C, ^15^N-labelled). As a step towards method development for selective cyanide labelling, we then transformed light and heavy cyanylated peptides to light and heavy tetrazoles, using Zn^2+^-catalysed click chemistry developed by Demko and Sharpless^28,29^ (Fig. 4f). We observed 124 sites modified by heavy tetrazole in at least three of five biological replicates (Fig. 4g). While a small number of sites were found to be overlapping (Extended Data Fig. 5f), most of the sites labelled with heavy tetrazole were not found as endogenously cyanylated (Fig. 4h,i and Supplementary Figs. 3 and 4). There are at least two explanations for this observation: (i) as we could not perform peptide enrichment, most of the cyanylated sites were not detected and (ii) as intracellular crowding and protein compartmentalization are disturbed in tissue lysates, the heavy Gly-produced HCN acts in a manner similar to exogenous HCN donors. Nonetheless, the Zn^2+^-catalysed click chemistry could represent a strategy for chemoselective method development in future.

**Extended Data Fig. 6:**
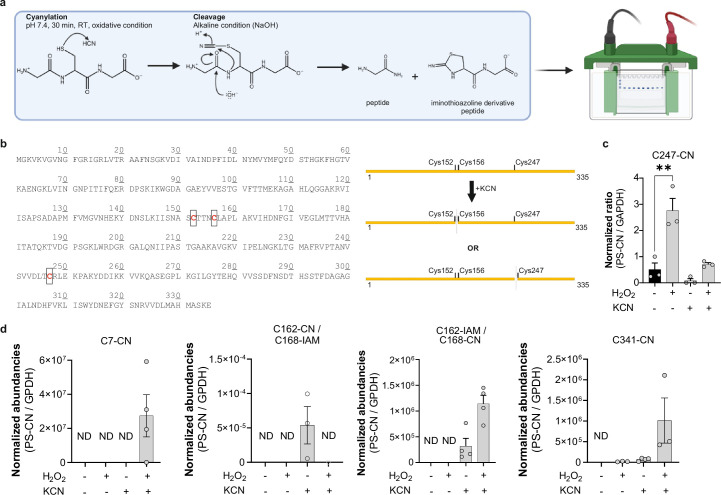
S-Cyanylation of glyceraldehyde 3-phosphate dehydrogenase (GAPDH) and glycerol-3-phosphate dehydrogenase (GPDH). **(a)** Schematic representation of chemical cleavage of polypeptide’s backbone occurring after S-cyanylation of target cysteine residues under alkaline conditions. Cleavage is then detected by SDS-PAGE followed by Coomassie-staining. Created with BioRender.com. **(b)** Human GAPDH contains three cysteine residues. S-cyanylation of GAPDH results in the generation of two visible bands that are consistent with one single cleavage reaction and one S-cyanylation site. **(c)** MS label-free quantification of S-cyanylation of C247 peptide. Peptide intensity is normalized to the total GAPDH in each sample. 0.55 µM GAPDH was treated with 10 µM KCN, 10 µM H_2_O_2_ or their combination (n=XX/group, technical replicates). **(d)** MS label-free quantification of GPDH treated with 10 µM KCN, 10 µM H_2_O_2_ or their combination (n = 5/group, biological replicates). ND – not detected. Data in **c**, **d** are expressed as the mean ± s.e.m. Data in **c** were analysed with a two-way ANOVA followed by Bonferroni’s multiple-comparisons test. **p < 0.01 indicates significant differences. Source data

How does cyanide modify proteins? Direct reaction of a thiol with HCN would not be possible, so HCN would have to react with electrophilic sulfur. Based on the known reactivity profile of cyanide with thiol groups^30–32^, we propose that cyanide reaction with cysteine residues requires first that the SH group is first oxidized to either sulfenic acid (-SOH) or a disulfide (S-S or S-SR) and then cyanide reacts to yield the -SCN product (Fig. 4j). To test this model and to better understand the potential outcome of cyanylation on enzyme activity, we used glyceraldehyde-3-phosphate dehydrogenase (GAPDH) and glycerol-3-phosphate dehydrogenase (GPDH) as two model proteins. Incubation of GAPDH with H_2_O_2_ inhibited its catalytic activity, while addition of KCN had no effect. However, incubating the enzyme with equimolar amounts of H_2_O_2_ and KCN yielded almost a twofold increase in enzyme activity (Fig. 4k). From gel electrophoresis and MS experiments, we found evidence for S-cyanylation of cysteine residues based on characteristic peptide bond cleavage at the cyanylation site after alkalization (Fig. 4k and Extended Data Fig. 6a–c). In the case of GPDH, cyanide inhibited enzyme activity (Fig. 3l), which was associated with cyanylation of multiple residues (Extended Data Fig. 6d).

Taken together, the above data demonstrate that (i) mammalian proteins are endogenously cyanylated, (ii) cyanylation originates from glycine as a source of cyanide and could occupy a substantial portion of protein’s thiol pool, and (iii) cyanide can remodel the intracellular landscape of cysteine PTMs, with a functional outcome (for example, enzyme activation or inactivation); cyanide may also serve as a redox switch from one PTM (such as SH oxidation or glutathionylation) to another (cyanylation).

## Endogenous cyanide supports bioenergetics and proliferation

**Fig. 5:**
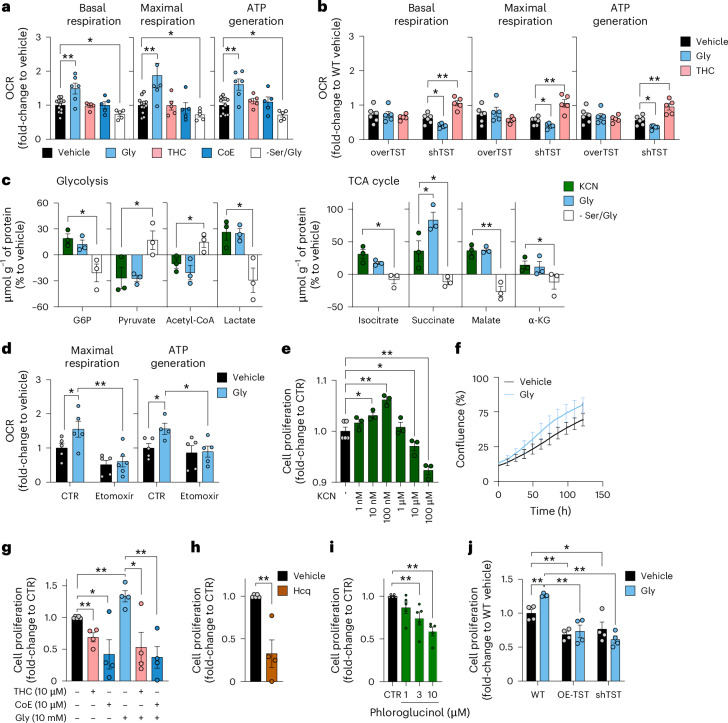
Endogenous cyanide generation supports cellular bioenergetics and proliferation. **a**, Bioenergetic profile of HepG2 cells treated with 10 mM glycine for 24 h in the absence (Gly) or presence of 10 µM cyanide scavenger THC or CoE for the last 3 h, or grown in Ser/Gly-free medium (at least *n* = 5 per group, biological replicates). **b**, Bioenergetic profile of WT versus TST-overexpressing (OE-TST) versus TST-knockdown (shTST) HepG2 cells in the absence (vehicle) or presence of 10 mM glycine for 24 h (Gly) or 10 µM THC for 3 h (at least *n* = 5 per group, biological replicates). **c**, Targeted metabolomic analysis of HepG2 cells subjected to exogenous 10 nM KCN or 10 mM glycine, or grown in −Ser/Gly medium for 24 h. G6P, glucose-6-phosphate; α-KG, α-ketoglutarate (*n* = 3 per group, biological replicates). **d**, FFA oxidation analysis of HepG2 cells in the absence and presence of 10 mM glycine for 24 h (at least *n* = 5 per group, biological replicates). **e**–**i**, Proliferation of HepG2 cells in the presence of 1 nM–100 µM KCN (**e**), in the presence of 10 mM glycine (measured by the IncuCyte system) (**f**), in the presence of 0.4 mM (standard medium—control) or 10 mM glycine in the presence or absence of 10 µM THC or 10 µM CoE (**g**), 10 µM Hcq (**h**) or 1–10 µM Phl determined at 24 h by the 5-bromo-2′-deoxyuridine (BrdU) assay (**i**; at least *n* = 3 per group, biological replicates). **j**, Proliferation of WT versus OE-TST versus shTST HepG2 cells in the absence or presence of 10 mM glycine determined at 24 h by the BrdU assay (*n* = 4 per group, biological replicates). Data are expressed as the mean ± s.e.m. and were analysed with a two-way ANOVA followed by Bonferroni’s multiple-comparisons test. *P < 0.05 and **P < 0.01 indicate significant differences. TCA, tricarboxylic acid. Source data

Based on the recently demonstrated stimulatory effects of low concentrations of exogenously administered KCN on various bioenergetic parameters^32^, we tested the role of endogenously generated cyanide on cellular bioenergetics (Fig. 5a–d) and cell proliferation (Fig. 5e–g) in HepG2 cells. Glycine increased mitochondrial electron transport and ATP generation and cyanide scavengers abrogated glycine’s stimulatory effect (Fig. 5a–c). Serine/glycine deprivation exerted inhibitory effects on various bioenergetic parameters (Fig. 5a–c).

**Extended Data Fig. 7:**
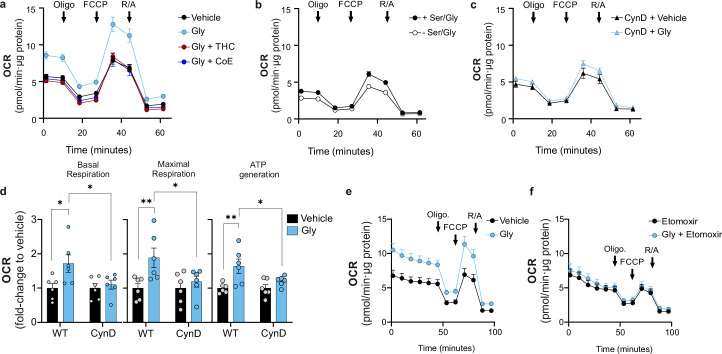
Endogenous HCN modulates cellular bioenergetics in HepG2 cells: extracellular flux analysis. **(a)** Glycine-dependent stimulation of cellular bioenergetics is abrogated by HCN scavengers. Extracellular flux analysis of HepG2 cells incubated with 10 mM glycine (Gly) for 24 h followed by 3 h with 10 µM of the cyanide scavengers (trihistidyl-cobinamide (THC) or dicobalt edetate (CoE). OCR: oxygen consumption rate (at least n = 5/group, biological replicates). **(b)** HepG2 cells incubated with serine/glycine free medium (-Ser/Gly) or with serine/glycine free medium re-supplemented with 0.4 mM serine and 0.4 mM glycine (+Ser/Gly) (at least n = 5/group, biological replicates). **(c,d)**. Glycine-dependent stimulation of cellular bioenergetics is abrogated by overexpression of the HCN-catabolizing enzyme cyanide dihydratase (CynD) in HepG2 cells, compared to normal control wild-type (WT) cells. Extracellular flux analysis (n = 6/group, biological replicates). **(e,f)** Free fatty acid oxidation extracellular flux analysis of (**e**) control HepG2 cells and (**f**) HepG2 cells pretreated with 4 µM etomoxir (carnitine palmitoyl transferase-1 inhibitor). In both cases, cells were pre-incubated in presence of 10 mM glycine or vehicle for 24 h. Arrows in panels **a**, **b**, **c**, **e** and f represent the times of the addition of the ATP synthase inhibitor oligomycin, the uncoupling agent carbonyl cyanide-4-(trifluoromethoxy)phenylhydrazone (FCCP) and the combined addition of the mitochondrial Complex I inhibitor rotenone and the mitochondrial Complex III inhibitor antimycin (R/A) in the Extracellular Flux Analysis protocol (at least n = 5/group, biological replicates). Data are expressed as the mean ± s.e.m. Data were analysed with a two-way ANOVA followed by Bonferroni’s multiple-comparisons test. *p < 0.05 and **p < 0.01 indicate significant differences. Source data

The bioenergetic effect of glycine was also attenuated in cells overexpressing either TST or CynD (Fig. 5a,b and Extended Data Fig. 7a–c). In TST-overexpressing cells, basal bioenergetic parameters were ~30% lower than in wild-type control cells (Fig. 5b). Importantly, basal cellular bioenergetics were also reduced in cells where TST was knocked down (shTST; Fig. 5b). In shTST cells, the cyanide scavenger THC improved bioenergetic function (Fig. 5b), while in TST-overexpressing cells adding glycine or scavenging cyanide did not affect bioenergetics (Fig. 5b). The above data suggest that—similarly to the bell-shaped concentration responses associated with NO, CO and H_2_S^1–7^—endogenously produced cyanide supports cellular bioenergetics with a concentration optimum: cellular bioenergetic function is impaired both when endogenous cyanide levels are decreased or increased beyond optimal levels. Indeed, the inhibition of cytochrome C oxidase and consequent suppression of mitochondrial function in response to high cyanide concentrations are well established in the toxicological literature^8,33^.

In cells supplemented with glycine, several enzymes that regulate lipid metabolism and free fatty acid (FFA) oxidation were also upregulated; the latter findings suggest that cyanide may induce a shift towards FFA utilization. Thus, we tested the effect of etomoxir (a carnitine palmitoyltransferase-1 inhibitor that suppresses FFA oxidation-derived acetyl-coenzyme-A entry into the Krebs cycle) on the bioenergetic profile of HepG2 cells. Etomoxir exerted a more pronounced inhibitory effect on mitochondrial respiration in glycine-treated cells than in control cells and attenuated the stimulatory effect of glycine on mitochondrial respiration (Fig. 5d and Extended Data Fig. 7d–f).

**Fig. 6:**
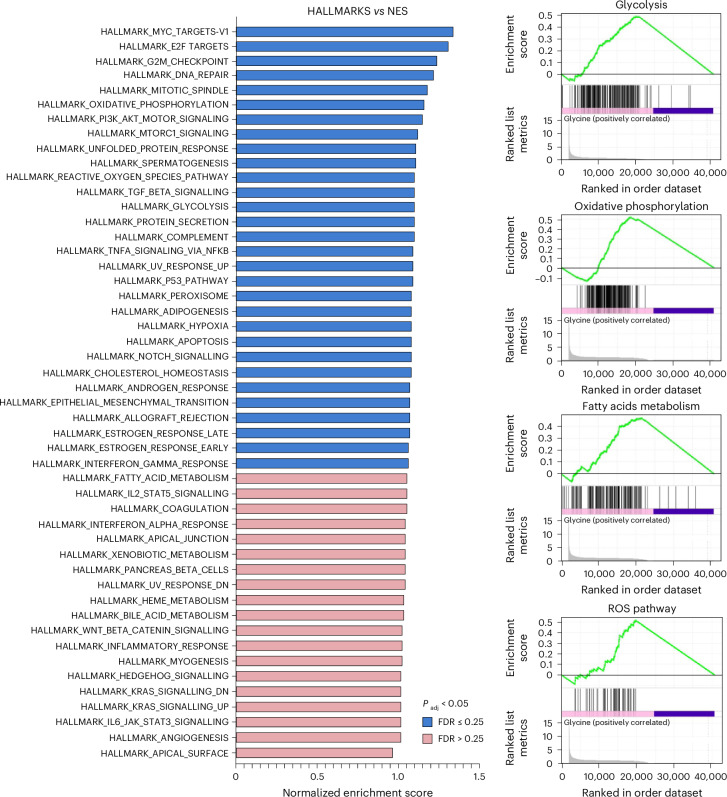
Glycine-induced transcriptomic changes. Gene-set enrichment analysis (GSEA), using the hallmark pathway gene sets of HepG2 cells incubated with 10 mM glycine for 24 h compared to vehicle. Data were obtained from RNA-seq of *n* = 3 biological replicates per group. FDR, false discovery rate. Source data

**Extended Data Fig. 8:**
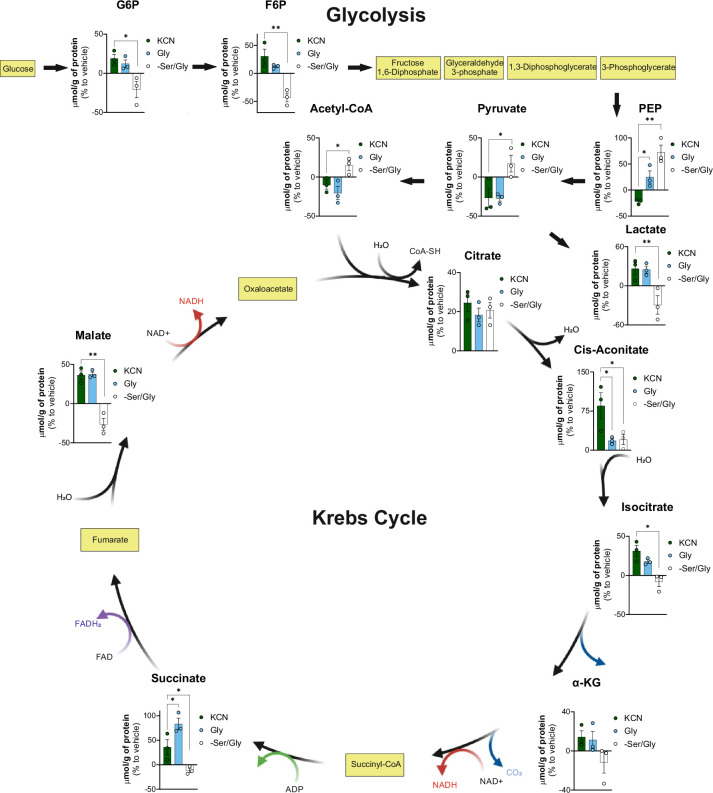
Effect of KCN, glycine supplementation or serine/glycine deprivation on glycolysis and Krebs cycle. Metabolite levels analyzed by LCMS in HepG2 cells cultured in normal medium, in normal medium supplemented with 10 nM KCN, 10 mM glycine (Gly) or under deprivation of serine and glycine (-Ser/Gly). Relative % changes compared to the baseline levels of the metabolites (detected in normal culture medium) are shown. Metabolites in yellow squares were not detected by the method used. Data are expressed as the mean ± s.e.m. of n = 3 independent experiments and were analysed with a two-way ANOVA followed by Bonferroni’s multiple-comparisons test. *p < 0.05 and **p < 0.01 indicate significant differences. Source data

Metabolomic analysis demonstrated that glycine—similarly to low KCN concentrations—stimulates glycolysis and activates the Krebs cycle, while serine/glycine deprivation exerts inhibitory effects (Fig. 5c and Extended Data Fig. 8). These effects are partially transcriptional: RNA-sequencing (RNA-seq) analysis revealed that glycine increases the expression of several enzymes that regulate glycolysis and oxidative phosphorylation (Fig. 6 and Supplementary Table 1).

Cell proliferation requires cellular ATP generation. Exogenously administered cyanide exerted a bell-shaped effect on HepG2 cell proliferation, increasing proliferation at low (nanomolar) concentrations and reducing proliferation at higher (10 micromolar and above) concentrations (Fig. 5e). In line with these observations, cyanide scavengers decreased, while glycine increased cell proliferation; this latter effect was suppressed by cyanide scavengers (Fig. 5f,g). Conversely, inhibition of cyanide production—via either lysosomal alkalization or inhibition of peroxidase activity—decreased cell proliferation (Fig. 5h,i). Importantly, both TST overexpression and TST silencing decreased cell proliferation (Fig. 5j), consistent with the concept that an optimal, physiological range of endogenous cyanide is necessary to support cell proliferation, and significant deviations in either direction impair bioenergetic function (Fig. 2o).

## Low-dose cyanide donation exerts cytoprotective and organ-protective effects

**Fig. 7:**
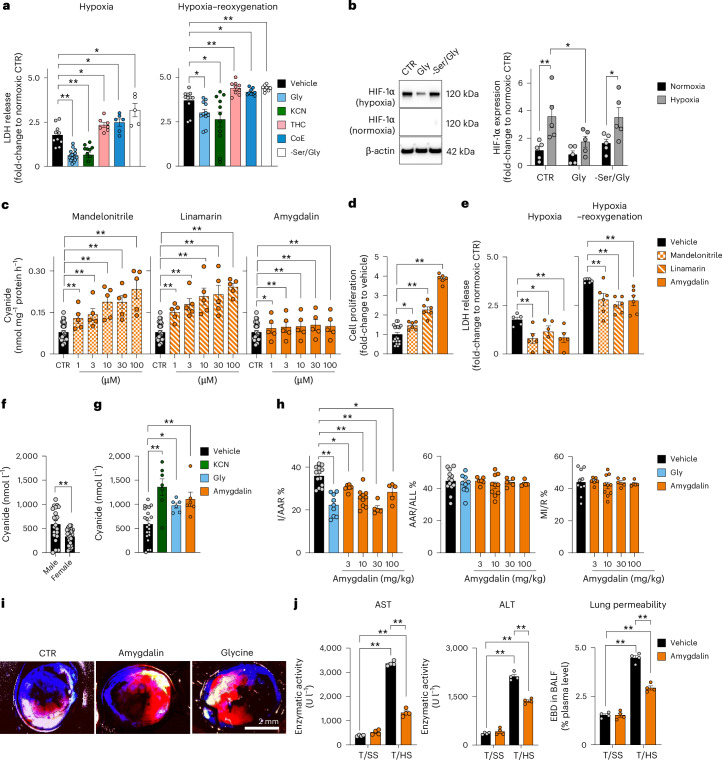
Controlled cyanide supplementation exerts cytoprotective effects. **a**,**b**, Effect of treatment with 10 mM glycine, 10 nM KCN, 10 µM THC, 10 µM CoE and the −Ser/Gly medium in HepG2 cells subjected to hypoxia (48 h) or hypoxia–reoxygenation (48/24 h) on lactate dehydrogenase (LDH) release (at least *n* = 5 per group, biological replicates; **a**) and HIF-1α expression (*n* = 5 per group, biological replicates; **b**). **c**, Cyanide release from mandelonitrile, linamarin and amygdalin (ECh method; at least *n* = 5 per group, biological replicates). **d**,**e**, Effect of 300 µM mandelonitrile, linamarin and amygdalin on cell proliferation (**d**) and hypoxia-induced and hypoxia–reoxygenation-induced cell injury (measured as LDH release) in HepG2 cells (at least *n* = 5 per group, biological replicates; **e**). **f**,**g**, Cyanide concentrations in mouse blood under baseline conditions (**f**) and after administration of 0.1 mg per kg body weight KCN, 100 mg per kg glycine or 10 mg per kg amygdalin (at least *n* = 6 per group, biological replicates; **g**). **h**,**i**, Effect of 300 mg per kg glycine or 3–300 mg per kg amygdalin on infarct size in a model of myocardial ischaemia–reperfusion in male C57BL/6J mice. The infarct size (I) relative to the area at risk (AAR), AAR relative to the whole myocardial area (ALL) and myocardial ischaemia (MI) relative to reperfusion (R) are shown (at least *n* = 5 per group, biological replicates). Images shown in **i** are representative of *n* = 6 biological replicates per group. **j**, Effect of 10 mg per kg amygdalin on markers of organ damage (AST, aspartate aminotransferase; ALT, alanine transaminase; lung permeability) in a model of haemorrhagic shock in male C57BL/6J mice. T/SS, sham-shock; T/HS, haemorrhagic shock (*n* = 4 per group, biological replicates). Data in **a**–**h** and **j** are expressed as the mean ± s.e.m. Data in **a**–**e**, **g**, **h** and **j** were analysed with a two-way ANOVA followed by Bonferroni’s multiple-comparisons test. Data in **f** were analysed with a two-sided Student’s *t*-test. *P < 0.05 and **P < 0.01 indicate significant differences. BALF, bronchoalveolar lavage fluid; EBD, Evans blue dye. Source data

Donation of small amounts of NO, CO and H_2_S to cells exerts protective effects^1–7^. We, therefore, hypothesized that endogenously generated cyanide may also exert a similar effect. Glycine supplementation protected HepG2 cells from hypoxia and hypoxia–reoxygenation-induced cell death (Fig. 7a). A low KCN concentration (10 nM) recapitulated this protection (Fig. 7a). On the other hand, cyanide scavenging (THC, CoE) or omission of glycine/serine from the culture medium exacerbated cell death (Fig. 7a). Glycine supplementation under normoxia upregulated several genes involved in the oxidative stress response (Fig. 6), while in hypoxic conditions glycine attenuated the upregulation of hypoxia-inducible factor 1-alpha (HIF-1α) expression (Fig. 7b). Significant changes in gene expression were also observed both with TST overexpression and TST silencing, including effects on multiple key biochemical pathways relevant for cell metabolism, proliferation and viability (Supplementary Table 2).

Similarly to low concentrations of exogenous KCN^32^, the cyanogenic compounds mandelonitrile, linamarin and amygdalin increased cyanide concentrations (Fig. 7c), stimulated cell proliferation (Fig. 7d), recapitulated the cytoprotective effect of glycine and cyanide in hypoxia and reoxygenation (Fig. 7e).

Basal cyanide concentration in mouse blood was 585 nM ± 73 nM in male mice and 364 nM ± 31 nM in female mice (Fig. 7f); administration of amygdalin (10 mg per kg) or glycine (100 mg per kg) to male mice increased the blood cyanide concentration 2–3-fold to ~1 µM. A comparable increase in the blood cyanide concentration could be achieved by the administration of a low, subtoxic dose (0.1 mg per kg body weight) of KCN (Fig. 7g). Using the detection method used in the current study, the cyanide concentration in the blood of healthy non-smoking humans was previously quantified as 540 nM ± 10 nM (*n* = 45)^34^.

In a mouse model of myocardial ischaemia–reperfusion, glycine supplementation reduced infarct size (Fig. 7h,i). In the same model, amygdalin also exerted a protective effect, and exhibited a bell-shaped dose–response effect, with the most pronounced protective effect obtained at 10–30 mg per kg body weight. Similarly, in a mouse model of haemorrhagic shock, amygdalin reduced the degree of hepatic and pulmonary injury (Fig. 7j).

## Cyanide is overproduced in NKH

**Fig. 8:**
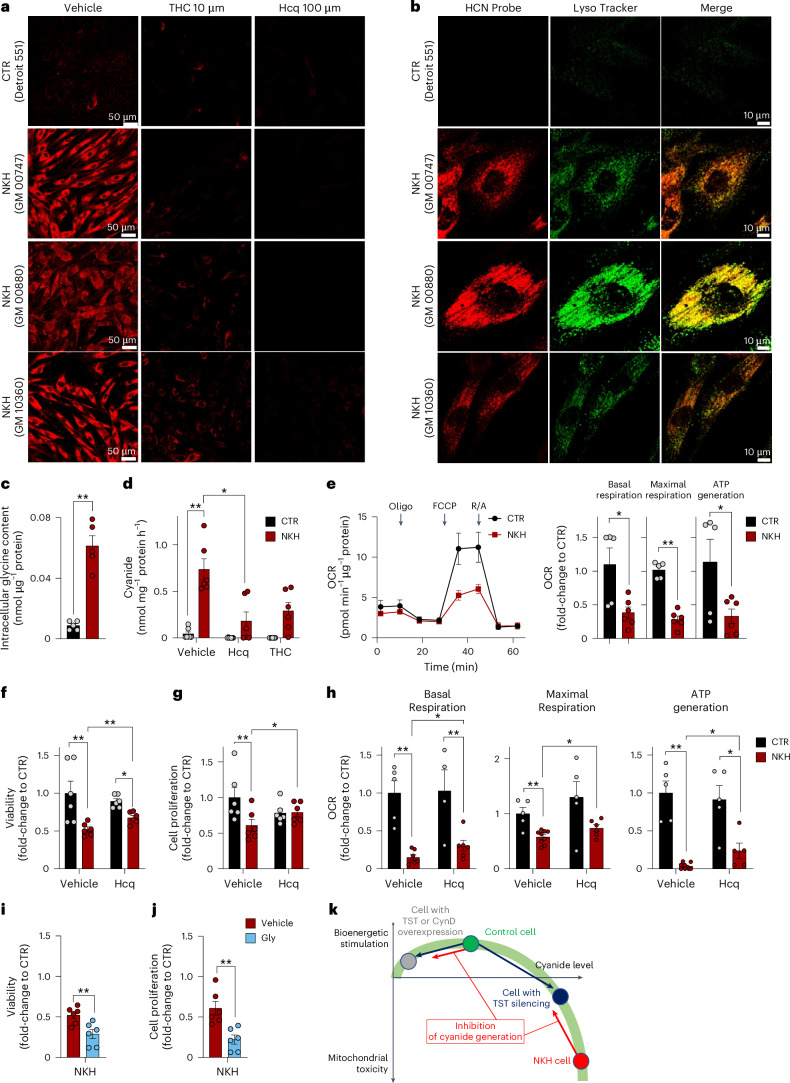
Endogenous cyanide generation is increased and cell function is diminished in fibroblasts derived from individuals with NKH. **a**, Confocal microscopy images showing increased endogenous cyanide levels in fibroblasts derived from individuals with NKH (cell lines GM00880, GM00747 and GM10360) compared to control fibroblasts from healthy individuals (Detroit 551) in controls (vehicle) and in the presence of 10 µM THC or 100 µM Hcq as visualized by CSP cyanide-selective probe. Images shown are representative of *n* = 3 biological replicates per group. **b**, Confocal microscopy images showing the partial colocalization of cyanide (CSP probe) with lysosomes (LysoTracker) in NKH fibroblasts compared to healthy controls. Images shown are representative of *n* = 3 biological replicates per group. **c**, Quantification of intracellular glycine concentrations in control (CTR) and NKH fibroblasts (*n* = 5 per group, biological replicates). **d**, Cyanide production in CTR and NKH fibroblasts under basal conditions (vehicle) and after 24 h treatment with 10 µM Hcq or 10 µM THC (ECh method; at least *n* = 6 per group, biological replicates). **e**, Bioenergetic profile measured by extracellular flux analysis in healthy control and NKH fibroblasts indicating mitochondrial dysfunction. OCR, oxygen consumption rate. Arrows represent the addition of ATP synthase inhibitor oligomycin, the uncoupling agent carbonyl cyanide-4-(trifluoromethoxy)phenylhydrazone (FCCP) and the combined addition of the mitochondrial complex I inhibitor rotenone and the mitochondrial complex III inhibitor antimycin (R/A) in the extracellular flux analysis protocol. **f**–**h**, Cellular viability (LDH release; *n* = 6 per group, biological replicates; **f**), proliferation rate (BrdU incorporation; *n* = 6 per group, biological replicates; **g**) and cellular bioenergetic parameters (**h**) in healthy controls and NKH fibroblasts in the absence or presence of 30 µM hydroxychloroquine for 72 h (at least *n* = 5 per group, biological replicates). **i**,**j**, Cell viability (*n* = 6 per group, biological replicates; **i**) and proliferation (*n* = 6 per group, biological replicates; **j**) of NKH fibroblasts at the baseline (vehicle) or treated with 10 mM glycine for 72 h. **k**, Proposed scheme of the bell-shaped concentration–response curve of cyanide in mammalian cells. At physiological concentrations, cyanide supports mitochondrial function, stimulates metabolism and supports proliferation. TST or CynD overexpression results in an increased decomposition of endogenously generated cyanide, and attenuates these stimulatory effects (black arrow). A similar mechanism is responsible for the bioenergetic effect of cyanide scavengers or inhibitors of cyanide generation in healthy control cells (red arrow). TST silencing attenuates the decomposition of endogenously generated cyanide (blue arrow). Cyanide accumulates and reaches levels at which it impairs mitochondrial function, suppresses bioenergetics and proliferation. When cyanide is generated at very high rates (such as in NKH cells, which accumulate glycine), cyanide reaches concentrations where it markedly impairs metabolism and proliferation and exerts cytotoxic effects. Inhibition of cyanide generation in NKH cells attenuates these toxic effects (red arrow). Data in **c**–**j** are expressed as the mean ± s.e.m. Data in **d**–**h** were analysed with a two-way ANOVA followed by Bonferroni’s multiple-comparisons test. Data in **c**, **i** and **j** were analysed with a two-sided Student’s *t*-test. *P < 0.05 and **P < 0.01 indicate significant differences. Source data

Glycine encephalopathy (also known as nonketotic hyperglycinaemia or NKH)^35^ is a devastating disease caused by mutations in the genes *GLDC* or *AMT* (genes that encode essential proteins of the glycine cleavage enzyme system), which lead to a pathological build-up of glycine in the cells and blood of individuals with NKH. We hypothesized that NKH could also result in the cellular accumulation of endogenous intracellular cyanide, potentially contributing to cytotoxic effects. Confocal microscopy revealed that fibroblasts derived from individuals with NKH—in the standard culture medium containing 400 µM glycine—show a markedly elevated cyanide signal compared to control fibroblasts (Fig. 8a). The cyanide signal was strongest in the lysosomes, but was also markedly distributed throughout the cytosol (Fig. 8b). As expected, intracellular glycine was markedly higher in NKH cells than in normal control fibroblasts from healthy individuals (Fig. 8c). NKH fibroblasts generated cyanide at approximately a 30-fold higher rate than control fibroblasts (Fig. 8d) and treatment with the lysosomal alkalinizer hydroxychloroquine or the cyanide scavenger THC reduced cyanide levels (Fig. 8d). Mitochondrial electron transport chain activity, ATP generation (Fig. 8e), cell viability and proliferation rate (Fig. 8f,g) were significantly lower in NKH fibroblasts than control cells. Hydroxychloroquine improved the bioenergetics, viability and the proliferation rate of NKH fibroblasts (Fig. 8f–h), while glycine supplementation reduced their viability and proliferation (Fig. 8i,j). These findings suggest that cyanide generation in NKH cells reaches cytotoxic levels (Fig. 8k).

## Discussion

Cyanide is generally considered a toxic molecule to mammalian cells due to inhibition of mitochondrial respiration at complex IV^8^. However, recent studies indicate that KCN administration to mammalian cells, at nanomolar to low micromolar concentrations, stimulates mitochondrial respiration, promotes cell proliferation and protects the cells from oxidative damage^32,36^. While these data suggested that cyanide may have regulatory roles in mammalian cells, the question of whether cyanide is an endogenous mammalian gaseous mediator remained to be addressed. Cyanide is known to play such roles in certain bacteria and plants^27,37^. The current report demonstrates that mammalian liver and spleen produce cyanide under basal conditions, and that cyanide generation can be increased with glycine supplementation. Biochemical assays demonstrated that cyanide production occurs at an optimum pH of 4.5, a pH typically found only in lysosomes. Indeed, lysosomes were shown to produce cyanide in situ as well as ex vivo and cyanide generation was stimulated by glycine. Conversely, disrupting the lysosomal pH or inhibiting the lysosomal glycine transporter reduced cyanide production. Similarly to recent findings regarding low concentrations of exogenously supplied KCN^30^, we conclude that endogenously produced cyanide in mammalian cells and tissues exerts physiological roles such as (i) support of mitochondrial respiration, (ii) stimulation of cell proliferation and (iii) cytoprotection. These roles appear to be applicable for various parenchymal cells; while the current project focused on hepatocytes, detectable cyanide generation was also found in cultured epithelial cells, endothelial cells and fibroblasts. Moreover, cells of monocytic and neutrophilic lineage (U937, HL60) and primary human PBMCs and neutrophils also produced high amounts of cyanide, both basally and after incubation with glycine. The role of cyanide generation in the function of immune cells is intriguing and remains a subject of further investigation.

The mechanism of cyanide’s action in mammalian cells is likely multifaceted. In the current study, we focused on the cyanylation of protein cysteine residues. Cyanylated proteins have previously been detected in plants and human plasma, and sub-micromolar concentrations of cyanide have been found in human blood^30,31,34,38^. The current report shows that hundreds of proteins are physiologically cyanylated in mammalian cells and that the portion of the proteins affected by this modification could be quite high. Based on the experiments with GAPDH and GPDH, the functional response to cyanylation can be either stimulatory or inhibitory, depending on the particular protein. This is similar to the PTMs of protein -SH group elicited by other gasotransmitters—nitrosylation by NO and persulfidation by H_2_S—which can be stimulatory or inhibitory, depending on the particular protein and on the particular thiol involved^6,7^. Among the proteins whose cyanylation was found to be affected by glycine supplementation or cyanide donors, very-long-chain acyl-CoA synthetase, short-chain specific acyl-CoA dehydrogenase (mitochondrial), medium-chain specific acyl-CoA dehydrogenase (mitochondrial), acyl-coenzyme A synthetase ACSM5 (mitochondrial) and GPDH (mitochondrial) are of particular interest, since these proteins are directly involved in beta oxidation and fatty acid biosynthesis. We propose that endogenous cyanide—through a combination of transcriptional, translational and posttranslational mechanisms—acts as a global regulator of cell function. One aspect of this reprogramming is a shift towards oxidation of FFAs, which likely occurs through the effects of cyanide at multiple enzymes that regulate FFA catabolism. This type of shift is known to occur physiologically, for instance, in response to fasting or prolonged exercise^39^.

Glycine is a common, non-essential amino acid that is generated endogenously in mammalian cells through de novo synthesis, and is supplied by dietary sources. It enters cells from the extracellular space through various transporters, and is used for de novo synthesis of proteins and nucleotides^40^. Its intracellular concentration is in the 3–10-mM range, while its lysosomal concentration is ~0.5 mM (refs. ^41–43^). Glycine supplementation is cytoprotective in various models of hypoxia and ischaemia and reperfusion in vitro and in vivo^43^. Based on the current data, we propose that part of the beneficial effect of glycine may be due to its ability to stimulate the synthesis of low—cytoprotective—concentrations of cyanide. Direct measurements of blood cyanide concentration showed that 100 mg per kg body weight glycine produces a peak blood cyanide concentration of approximately 1,200 nM. Thus, at the therapeutically effective doses of glycine, cyanide concentration in the blood likely remains in the low-micromolar concentration range. We do not propose that slight changes in extracellular glycine can affect cellular cyanide generation, because of the high concentrations of intracellular glycine in mammalian cells. The fact that modest changes in the extracellular concentration of an amino acid precursor do not affect the generation of various gasotransmitters in healthy normal cells is well known in the biology of NO and H_2_S^1–7^. Although l-arginine is the precursor of NO, addition of extracellular l-arginine in most cases does not stimulate NO production. Likewise, H_2_S is produced from cysteine and homocysteine by mammalian cells, but addition of these molecules to cells does not drive additional H_2_S generation in most normal cells and tissues^1–7^.

Cytoprotective concentrations of cyanide may also be generated through administration of low concentrations of cyanogenic molecules—as exemplified in the current report by amygdalin, mandelonitrile and linamarin. These molecules are primarily known for their potential toxicity due to cyanide release; they have been tried as cytotoxic agents in the treatment of cancer, an approach that is severely hampered by toxicity to the host^44^. In sharp contrast to this prior concept, the current data suggest that administration of low doses of cyanogenic compounds could be an experimental therapeutic strategy to exert cytoprotective effects against various local or systemic ischaemic conditions. When mandelonitrile, linamarin or amygdalin is added to HepG2 cells at concentrations of 1–100 µM, cyanide generation rates are in the range of 0.1–0.2 nmol per mg of protein per hour, which is comparable to the endogenous cyanide generation rate in these cells. Based on direct blood measurements (Fig. 7g), we estimate that cyanide blood concentrations in the therapeutic dose range of amygdalin (10–30 mg per kg body weight) are in the 200-nM–1-µM range.

The bell-shaped dose–response effect, a fundamental characteristic of NO, CO and H_2_S biology^1–7^, emphasizes the balance between cytoprotective and cytotoxic concentrations of diffusible mammalian gaseous mediators. This balance depends on the cell’s capacity to produce and detoxify these compounds, and once this capacity is exceeded, the result is often detrimental. Indeed, the current and prior findings^32,33,36^ demonstrate a bell-shaped concentration–response of exogenously administered cyanide on cellular bioenergetics and proliferation, and a similar bell-shaped concentration–response curve applies for endogenously generated cyanide, as well (Fig. 8k). A bell-shaped concentration–response was also evident for amygdalin in our myocardial infarction model (Fig. 7h); similar bell-shaped concentration–response effects have been previously observed for NO or H_2_S in this model^45,46^. In fibroblasts from individuals with NKH, the pathological accumulation of glycine leads to markedly increased (~30-fold) cyanide production rates, crossing the cytotoxic threshold. The improved bioenergetic function and viability of NKH-derived fibroblasts following treatment with the lysosomal alkalinizer hydroxychloroquine indicate that lysosomal generation of cyanide plays a role in the observed cellular dysfunction. The marked reduction in mitochondrial electron transport chain activity and cellular proliferation rates in fibroblasts from individuals with NKH further support this view (Fig. 8k). These findings could have implications for understanding the pathophysiology of glycine encephalopathy and potentially offer new a therapeutic approach for this condition.

Although most of the scientific literature related to the role of cyanide in mammals focuses on its toxicological properties, one should emphasize that cyanide is generated endogenously in several bacterial and plant species, serving various regulatory effects, such as quorum sensing, biocontrol, germination development and immunity^37,47,48^. Indeed, cyanide and H_2_S were already present on the planet several billions of years ago, before the appearance of atmospheric oxygen, and bacteria and plants have been linked to early biochemical processes that led to evolution of higher organisms^49–53^. In this context, it makes sense that enzymatic systems evolved to produce these gases at low rates, despite their obvious toxicity at higher concentrations. Likewise, their important reactions—particularly with protein cysteine residues^54–58^—play evolutionarily conserved roles and functions across the animal and plant kingdom. In this context, the current findings place cyanide—together with NO, CO and H_2_S—in the group of mammalian regulatory gasotransmitter species (Supplementary Table 3). These molecules play various regulatory roles, which can be distinct, overlapping, cooperative or opposing^1,6,53–58^.

The current report characterizes the mechanism and action of endogenously generated cyanide in mammalian cells and highlights its role in the regulation of cell metabolism. Nevertheless, further studies remain to be conducted to further characterize these regulatory roles, including further details of how cyanide production is regulated, whether and how cyanylation is targeted to specific proteins, and how cyanylation of specific targets and of the cyanyl-proteome overall contributes to metabolic and physiological functions in mammalian cells. Additional work also remains to be conducted on the delineation of potential interactions of cyanide with various other gaseous mediators in mammalian cells, and on the potential regulatory roles of cyanide on gene expression, metabolism and cell viability/cell death in physiological and pathophysiological conditions.

## Methods

### Animals

The protocol used for these studies was approved by the Institutional Animal Care and Use Committee of the University of Fribourg (Fribourg, Switzerland). C57BL/6J male and female mice were purchased from Janvier Laboratories (Le Genest-Saint-Isle, France). MPO knockout male mice (*Mpo*^−/−^, strain 004265) and PXDN heterozygous mice (*Pxdn*^+/−^; strain 042166), both on C57BL/6J background, were purchased from Jackson Laboratories. Despite repeated breeding efforts, we were unable to generate *Pxdn*^−/−^ mice, and thus tissues from *Pxdn*^+/−^ mice (male) were used. Animals were housed in a light-controlled room with a 12-h light–dark cycle and had ad libitum access to food and water. The room temperature for mice was 20–24 °C (68–75 °F) and was kept as stable as possible. All studies were performed on 12–18-week-old mice. The measurement of cyanide blood levels was compared between male and female mice, and the production of cyanide from liver homogenates was also compared between livers from male and female mice. The myocardial infarction and the haemorrhagic shock studies were performed in male mice.

### Myocardial ischaemia–reperfusion injury model

The protocol used for these studies was approved by the Institutional Animal Care and Use Committee of the University of Athens (Athens, Greece). Myocardial infarction was induced by ligation of the left coronary artery^59^. Eight- to ten-week-old male C57BL/6J mice were randomly divided into six groups. Controls (*n* = 10) received saline vehicle only. Amygdalin (3, 10, 30 or 100 mg per kg body weight; Cayman, 26668) was administered intraperitoneally (i.p.) at 10 mg per kg body weight, 1 h before ischaemia (*n* = 7). Glycine (300 mg per kg body weight, Fisher Scientific, BP381) was administered i.p. at 300 mg per kg body weight, 1 h before ischaemia (*n* = 9). Animals were anaesthetized by i.p. injection of ketamine–xylazine. Anaesthetic depth was evaluated by the loss of pedal reflex to toe-pinch stimulus and breathing rate. Additional anaesthesia (a quarter of the initial dose) was applied during the first hour of reperfusion. A tracheotomy was performed for artificial respiration at 150 strokes per minute with a tidal volume of 200 μl. A thoracotomy was then performed, and the pericardium was carefully retracted to visualize the left anterior descending coronary artery, which was ligated using a 6-0 silk suture (Ethicon, W888) placed 3 mm below the tip of the left atrium with the help of a 5-mm piece of a 1-mm-diameter catheter tube. The heart was allowed to stabilize for 15 min before ligation to induce ischaemia. After the ischaemic period, the ligature was released to induce the reperfusion of the myocardium. Throughout the procedure, body temperature was maintained at 37 + 0.5 °C with a heating pad. After reperfusion, hearts were rapidly excised from mice and directly cannulated through the aorta and washed with Krebs buffer (118.5 mM NaCl, 25 mM NaHCO_3_, 4.7 mM KCl, 1.2 mM MgSO_4_, 1.2 mM KH_2_PO_4_, 11 mM glucose and 1.5 mM CaCl_2_, pH = 7.4) for blood removal. Hearts were perfused with 500 μl 2% Evans blue, diluted in Krebs buffer. Afterwards, they were kept at −80 °C for 1 h and then sliced in 2-mm sections parallel to the atrioventricular groove. The slices were incubated in 2 ml of 1% TTC phosphate buffer (PBS pH = 7.4) at 37 °C for 10 min. Slices were then compressed between glass plates 1 mm apart and photographed with a Leica DFC310 FX Digital Color Camera through a Nikon SMZ800 stereoscope and measured with the National Institutes of Health ImageJ software. The myocardial area at risk as well as the infarcted and the total area were automatically transformed into volumes. Infarct and risk area volumes were expressed in cm^3^ and the percentage of infarct-to-risk area ratio (%I/AAR), of area at risk to whole myocardial area (% AAR/All) and of myocardial ischaemia to reperfusion injury (%MI/R) were calculated.

### Haemorrhagic shock model

The protocol used for these studies was approved by the Institutional Animal Care and Use Committee of Columbia University. Male C57BL/6J mice were randomly assigned into the following groups: trauma/sham-shock (T/SS) receiving vehicle, T/SS receiving amygdalin, trauma/haemorrhagic shock (T/HS) receiving vehicle and T/HS receiving amygdalin. The mice were pretreated (30 min before T/SS or T/HS) i.p. with either vehicle or amygdalin (10 mg per kg body weight). Animals were anaesthetized with 1% isoflurane, and rectal temperature was maintained at 36.5–37.5 °C with a heating pad. A fixed-pressure model was used to induce haemorrhagic shock^60^. Briefly, after anaesthesia mice received a midline laparotomy of 2 cm and then the incision was closed with 4-0 silk suture (Covetrus, 034902). The right and left femoral arteries were isolated and catheters were placed for monitoring blood pressure and blood withdrawal, respectively. For blood withdrawal, a sterile 1-ml syringe with a 30-gauge needle was used, which was attached to PE-10 tubing and filled with 0.2 ml of 1% heparinized saline. Each mouse received 1 U heparin. Blood pressure was monitored using the Powerlab 8/30 continuous blood pressure monitoring system and analysed with the LabChart 8.1.30 software (AD Instruments). After 5 min of baseline recording, mice were treated with a drug or vehicle, followed by a 2.5-h period of shock. Blood pressure was maintained at 28–32 mm Hg by withdrawing or reinfusing the shed blood. At the end of the shock period, mice were resuscitated with Ringer’s lactate at three times the amount of shed blood over 15 min. Three hours after the T/HS period, mice were re-anaesthetized with isoflurane. Evans blue dye (Sigma-Aldrich, E2129) was administered through the tail vein and 5 min later about 1 ml of blood was withdrawn from tail artery. Twenty minutes later, the mice were euthanized and the trachea was isolated for BALF sample collection. After a small incision, a syringe with a 23-gauge needle filled with 1 ml of sterile saline was placed in the trachea. Lungs were injected and aspirated two times and the BALF was collected. To measure the Evans blue dye in BALF, the BALF sample was centrifuged at 4 °C at 1,500*g* for 20 min. The supernatant was collected and assayed at 620 nm by spectrophotometry. The concentration of Evans blue dye in the BALF was then expressed as a percentage of its plasma concentration. Plasma levels of the liver enzymes AST and ALT levels were also measured. Plasma samples were diluted (1:10) with AST (Thermo Fisher, TR70121) and ALT (Thermo Fisher, TR71121) reagents; data were acquired using a spectrophotometer at 340 nm and 405 nm.

### Cell culture

HepG2 hepatocellular carcinoma cells (American Type Culture Collection (ATCC) HB-8065), CynD-overexpressing HepG2 cells, TST-overexpressing HepG2 cells and TST-knockdown (shTST) HepG2 cells were grown in Dulbecco’s Modified Eagle Medium (DMEM) containing 1.0 g l^−1^ d-glucose (Gibco, 21885), supplemented with 10% (vol/vol) heat-inactivated FBS (Hyclone), 100 units per ml of penicillin and 100 µg ml^−1^ of streptomycin. Hep3B cells (ATCC HB-8064) and HL60 cells (ATCC CCL-240) were grown in DMEM culture medium containing 1.0 g l^−1^ d-glucose (Gibco, 21885), supplemented with 10% (vol/vol) heat-inactivated FBS (Hyclone), 100 units per ml of penicillin and 100 µg ml^−1^ of streptomycin. Human umbilical vein endothelial cells (ATCC CRL-1730) were grown in Endothelial Cell Growth Medium (211-500, Cell Applications). HEK293A human embryonic kidney cells (kind gift from C. Wallace, University of Colorado Anschutz Medical Campus), MPO-overexpressing HEK293T cells, PXDN-overexpressing HEK293T cells and catalase-overexpressing HEK293T cells were cultured in DMEM containing 4.5 g l^−1^ glucose (Gibco, 11965). The culture medium was supplemented with 10% (vol/vol) heat-inactivated FBS (Hyclone), 2 mM Glutamax, non-essential amino acids, 100 units per ml penicillin and 100 μg ml^−1^ streptomycin. A549 lung carcinoma epithelial cells (ATCC CCL-185) were cultured in DMEM 4.5 g l^−1^ d-glucose (PAN Biotech, P04-03500) supplemented with 10% (vol/vol) heat-inactivated FBS (Hyclone), 100 units per ml penicillin and 100 μg ml^−1^ streptomycin. HCT116 (ATCC CCL-247) and HT29 colorectal adenocarcinoma cells (ATCC HTB-38) were cultured in McCoy’s 5A medium (Gibco, 16600) supplemented with 10% (vol/vol) heat-inactivated FBS (Hyclone), 100 units per ml penicillin and 100 μg ml^−1^ streptomycin. LoVo human colorectal adenocarcinoma cells (ATCC CCL-229) were cultured in Advanced DMEM/nutrient mixture F-12 (DMEM/F-12, 1:1, 1×; Gibco, 12634) supplemented with 10% (vol/vol) heat-inactivated FBS (Hyclone), 100 units per ml penicillin and 100 μg ml^−1^ streptomycin. U937 pro-monocytic, human myeloid leukaemia cells (ATCC CRL-1593.2) were cultured in RPMI 1640 medium (ATCC 30-2001) supplemented with 10% (vol/vol) heat-inactivated FBS (Hyclone), 2 mM Glutamax, 100 units per ml penicillin and 100 μg ml^−1^ streptomycin. Human dermal fibroblasts from a healthy participant (Detroit551, ATCC CCL-110) were cultured in Advanced DMEM/nutrient mixture F-12 (DMEM/F-12, 1:1, 1×; Gibco, 11320) supplemented with 0.1% lactalbumin hydrolysate, 10% (vol/vol) heat-inactivated FBS (Hyclone), 100 units per ml penicillin and 100 μg ml^−1^ streptomycin. U138-MG human glioblastoma cells (ATCC HTB-16) were cultured in DMEM (ATCC 30-2002) supplemented with 10% (vol/vol) heat-inactivated FBS (Hyclone), 100 units per ml penicillin and 100 μg ml^−1^ streptomycin. −Ser/Gly medium was obtained from US Biological (D9802-01). Cryopreserved human primary hepatocytes (from a 48-year-old male of European ancestry, AnaBios) were thawed in HEP-005 Anabios thawing medium, plated in HEP-003 Anabios plating medium and maintained in HEP-004 Anabios maintenance medium. Human skin fibroblasts from individuals with NKH (GM00880, from a 21-year-old male of European ancestry; GM00747, from a 1.5-year-old female of European ancestry; GM10360, from a 2-month-old male of European ancestry) were obtained from Coriell Institute for Medical Research and were cultured in DMEM (Hyclone, SH30243.01), supplemented with 15% (vol/vol) heat-inactivated FBS (Hyclone), 1% non-essential amino acids (Hyclone) and 100 units per ml penicillin and 100 μg ml^−1^ streptomycin.

All cells were grown in a humidified incubator at 37 °C and 5% CO_2_ atmosphere. For experiments and sub-culturing, cells were rinsed with PBS and detached from T75 flasks by incubating with 0.25% (wt/vol) trypsin containing 0.53 mM EDTA for 2–5 min at 37 °C followed by resuspension in culture medium.

### Generation of stably transfected cell lines

The lentivirus gene expression vector pLV[Exp]-Bsd-CMV was obtained from VectorBuilder. The coding sequences of MPO, PXDN, CAT, CynD and TST were codon optimized, synthesized and subcloned into the pLV vector by GeneScript. The TST shRNA plasmid was purchased from Santa Cruz Biotechnology (sc-36418-SH). Viral particles were produced in HEK293T cells using the third-generation lentiviral system. Cells were seeded at 70–80% confluence in a six-well plate and transiently transfected 4–6 h later with pLV or TST plasmid along with packaging plasmids pLP1, pLP2 and pLP/VSVG in a ratio of 4.2:2:2.8 using JetOptimus transfection reagent (Polyplus) according to the manufacturer’s instructions. The transfection mixture and medium were replaced with fresh culture medium after overnight incubation. Lentiviral supernatants were collected after 24 h and filtered with a 0.45-μm filtration unit and subsequently aliquoted and stored at −80 °C until use. HepG2 and HEK293A cells were transduced with lentiviral supernatant in the presence of 6 μg ml^−1^ protamine sulfate. Seventy-two hours following transduction, 5 μg ml^−1^ or 45 μg ml^−1^ Blasticidin S (InvivoGen) was added to the culture to select transduced cells.

### HCN detection

KCN and its solutions are poisonous to humans; they were handled with care in a well-ventilated hood. Additionally, HCN is released from solutions with pH values near or below the pKa of HCN (pKa = 9.2). Thus, all aqueous standards containing cyanide were prepared from KCN in NaOH (10 mM or 0.5 M, depending on the detection method) to ensure that cyanide remains in solution in its non-volatile CN^−^ form.

#### Sample preparation: mouse blood

Mice (C57BL/6J, male) were divided into a control group (0.9% NaCl; *n* = 10), a KCN group (0.1 mg per kg body weight, *n* = 7), a glycine group (100 mg per kg body weight, *n* = 7) and an amygdalin group (10 mg per kg body weight, *n* = 7). These agents were administered i.p.; the dose of KCN used in this study was over 50 times lower than the LD_50_ and ten times lower than the documented lowest observable adverse effect dose in mice. After 10 min (KCN) or 3 h (glycine or amygdalin), mice were euthanized using the i.p. injection of ketamine–xylazine. Blood (200 µl) was collected via cardiac puncture into heparinized and airtight tubes, closed with screw caps and immediately frozen at −80 °C until cyanide analysis with the Cyanalyzer LC–MS/MS method.

#### Sample preparation: mouse tissue

Mice were euthanized using CO_2_ and exsanguination. The animals were perfused using 20 ml chilled PBS through the ascending aorta for 2–4 min to remove blood from the tissues. Tissues (liver, kidney, lung, brain and spleen; 20–30 mg) were placed in 2-ml microcentrifuge tubes and homogenized in 2 ml PBS using three 2.4-mm metal beads (Omni International, 19-640-3) using a bead mill 4 mini homogenizer (Fisherbrand) for 240 s, with a speed of 4 m s^−1^. Samples were treated with 10 mM glycine, 10 µM THC or 10 µM CoE and incubated at 37 °C for 24 h. For protein heat inactivation, tissue homogenates were incubated at 95 °C for 1 h. For the physical inactivation of proteins (Freeze-Thawing, F&T), homogenates were subjected to three homogenization cycles made of sonication (30-s pulse followed by 30-s pause, repeated five times using an Ultrasonic Bath Sonicator), freezing (at −20 °C for 30 min) and thawing (at 37 °C for 1 min). In a separate group of liver homogenates, proteins were denatured by incubation with 2% SDS for 24 h at 37 °C. Cyanide production rates were measured with electrochemical, LC–MS/MS and MCC methods (see below).

#### Sample preparation: cultured cells

Cells were seeded into a six-well plate at 500,000 cells per well (HepG2, Hep3B, HT29, LoVo), 300,000 cells per well (HEK293A, Detroit 551, GM00880, GM00747 and GM10360), 250,000 cells per well (A549), 200,000 cells per well (HCT116), 50,000 cells per well (U138-MG) and 2,000,000 cells per well (U937) and incubated at 37 °C and 5% CO_2_. The next day, the medium was replaced with fresh medium containing glycine (10 mM) or −Ser/Gly medium or −Ser/Gly medium supplemented with increasing concentrations of glycine (1 mM, 5 mM or 10 mM). Cells were incubated at 37 °C and 5% CO_2_ for 24 h (in the case of the suspended U937 cells, cells were seeded directly in −Ser/Gly medium ± glycine). Human primary hepatocytes were seeded at 1,000,000 cells per well in a six-well plate coated with collagen in plating medium. After 6 h, medium was replaced with maintenance medium ± 10 mM glycine and further incubated for 24 h. For human primary hepatocytes, the day after seeding, cells were treated for 3 h with 10 µM THC. For HepG2 cells, the day after seeding, cells were treated with increasing concentrations of HCN scavengers THC and CoE (1–30 µM) and incubated at 37 °C and 5% CO_2_ for 3 h. Incubation (24 h) with 10–100 µM of the glycine transporter-1 inhibitor iclepertin (HY-138935, MedChemExpress) was used to inhibit the uptake of glycine into the cells. Increasing concentrations (0–100 µM) of the peroxidase inhibitor phloroglucinol (Sigma-Aldrich, 79330) or the MPO inhibitor AZD-5904 (Sigma-Aldrich, SML3274; 24 h) were used to inhibit peroxidase activity. Hydroxychloroquine (Sigma-Aldrich, H0915) at 1–30 µM (24 h) or bafilomycin (Alfa Aesar, J61835) at 0.01–1 µM (3 h) was used to increase the intra-lysosomal pH. Glycine-Glycine (Gly-Gly, Sigma-Aldrich, G1002-25G) at 150 mM (24 h) was used to inhibit cellular glycine uptake. The SHMT inhibitor SML2699 (iSHMT, Sigma-Aldrich; 100 µM, 24 h) or lometrexol hydrate (1–10 µM, 24 h; Sigma-Aldrich, SML0040) was used to inhibit intracellular serine/glycine interconversion. The glycine receptor antagonist strychnine (Sigma-Aldrich, S0532) was used at a concentration 10 pM, for 24 h. Various HCN releasers were used to increase HCN levels in HepG2 cells. One day after seeding, cells were treated with increasing concentrations (0–100 µM) of amygdalin (Sigma-Aldrich, A6005), linamarin (Toronto, TRCL466000) or mandelonitrile (Sigma-Aldrich, 116025) and incubated at 37 °C and 5% CO_2_ for 24 h. In all cases, after treatment, an aliquot of the supernatant was mixed (1:1, vol/vol) with 1 M NaOH for HCN measurements using the ECh method.

#### Sample preparation: human primary cells

Whole-blood leucoreduction filters containing total blood leucocytes obtained from the Transfusion CRF Fribourg (Fribourg, Switzerland) were used for isolation of PBMCs using density gradient centrifugation (Ficoll-Paque PLUS Medium, Cytiva). Human peripheral blood neutrophils were purchased from StemCell Technologies. PBMCs or neutrophils were maintained in RPMI 1640 medium, 10% FBS, 100 units per ml of penicillin and 100 μg ml^−1^ of streptomycin, 2 mM l-glutamine, 1 mM sodium pyruvate, 0.055 mM 2-mercaptoethanol (all from Gibco-Thermo Fisher) and 10 mM HEPES (Cytiva). For cyanide production assays, cells were incubated in closed cryotubes with PBS or glycine (10 mM) for 3 h at 37 °C. Then 75 µl of the supernatant was collected in a 0.2 ml tube containing 75 µl of 1 M NaOH and, after incubation at room temperature for 30 min, cyanide was measured using the ECh method.

#### Mouse primary hepatocytes collection and culture

Isolation of mouse primary hepatocytes was performed as described^61^. Male C57BL/6J mice were euthanized using CO_2_ asphyxiation and venae cavae and portal veins were exposed. A 25-gauge butterfly needle, pre-filled with 10 ml of warm (37 °C) perfusion buffer (HBSS without Ca^2+^ and Mg^2+^ supplemented with 25 mM HEPES and 0.5 mM EDTA, pH 7.4) in a 10 ml syringe, was used for cannulation of the vena cava and liver perfusion was manually performed. Meanwhile, collagenase type IV (MP Biomedicals, 0219511090) was reconstituted in perfusion buffer to a final concentration of 0.4 mg ml^−1^. The resulting filter-sterilized digestion buffer was used to perfuse the liver followed by 30 min of incubation at 37 °C. Afterwards, liver tissue was transferred to a Petri dish containing the digestion buffer. Small punctures were made across the liver using sterile tweezers, while avoiding the gall bladder. A small sterile spatula was then used to gently massage the liver, causing hepatocytes to be released into the buffer. The obtained cells were resuspended in 10 ml of cold plating medium (1 g l^−1^ glucose DMEM, supplemented with 5% FBS and 1% penicillin–streptomycin), and filtered through a 70-µm filter into two 50 ml Falcon tubes, each receiving an equal volume (5 ml) of the suspension. The Petri dish was washed with an additional 10 ml of cold plating medium, and 5 ml was added to each tube. After filtration, the tubes were centrifuged at 50*g* for 2 min. The cells were gently washed twice with 10 ml of cold plating medium. The resulting cell pellet was resuspended in 1 ml of cold plating medium and the cells were seeded into six-well plates (1,000,000 cells per well) pre-coated with collagen, followed by incubation at 37 °C for 4 h. Subsequently, the plating medium was replaced with a maintenance medium (Williams E medium, supplemented with 2 mM Glutamax and 1% penicillin–streptomycin), and cells were incubated overnight for the subsequent assays.

#### Cell lysates

HepG2 overexpressing CynD or TST or knocked down for TST (shTST) were detached with 0.25% (wt/vol) trypsin containing 0.53 mM EDTA for 2–5 min at 37 °C, resuspended in culture medium and collected by centrifugation at 1,000*g* for 5 min. Pellets were washed twice with ice-cold PBS and lysed with Cell-Lytic M buffer (Merck) supplemented with 1% phosphatase/protease inhibitor cocktail (Halt, 1861281, Thermo Fisher Scientific). Lysates were incubated on ice for 30 min and sonicated with three cycles of a 15-s pulse followed by a 15-s pause on ice, using an ultrasonic bath sonicator. Lysates were centrifuged at 17,000*g* at 4 °C for 20 min. The supernatant was collected and total protein content was quantified using the BCA method (Thermo Scientific, 23225). For the HCN degradation assay, 500 ng of cell lysate was incubated in 50 mM Tris-HCl, pH 7.4, in the presence of 100 µM KCN in a final volume of 25 µl ± 1 mM sodium thiosulfate. HCN degradation activity was stopped at different time points (0–1 h) by adding 25 µl 1 M NaOH, and cyanide detection was performed with the ECh method.

#### Lysosomes

Lysosome enrichments were performed from mouse liver or from HepG2 cells. The mouse liver was perfused with PBS as described above. After perfusion, 200 mg of liver tissue was collected from the left lobe, placed in ice-cold PBS and minced into small pieces via a scalpel, transferred into a 3-ml glass-Teflon Potter–Elvehjem homogenizer and homogenized (12 strokes) in 1 ml of lysosome isolation buffer (ab234047, Abcam), supplemented with phosphatase/protease inhibitor cocktail (Halt, 1861281) on ice. After homogenization, 1 ml of lysosome enrichment buffer (ab234047, Abcam) was added, followed by centrifugation at 500*g* at 4 °C for 10 min. The supernatant was further centrifuged at 20,000*g* at 4 °C for 20 min. The resulting supernatant was the cytosolic fraction (referred to as Cyto), while the pellet was loaded in a discontinuous density gradient media (ab234047, Abcam) and centrifuged at 147,000*g* at 4 °C for 2 h using a Sorvall Discovery M120SE ultracentrifuge equipped with an S52-ST swinging-bucket rotor. The layer corresponding to the lysosomal fraction was marked as Lyso, while the other fractions were pooled and marked as the extra-lysosomal fraction (referred to as Extra-Lyso). Both lysosomal and extra-lysosomal fractions were resuspended in 200 µl of suspension buffer (10 mM HEPES, 150 mM NaCl, pH 7.4).

For each sample of HepG2 cell lysosomes, cells from five T175 confluent flasks were pooled. When treatments were performed, cells were treated before collection (DMEM culture medium (CTR), 1 µM bafilomycin for 1 h, 100 µM hydroxychloroquine for 3 h, 150 mM Gly-Gly for 24 h) and incubated at 37 °C and 5% CO_2_. After treatment, cells were washed with PBS, detached with 0.25% (wt/vol) trypsin containing 0.53 mM EDTA and resuspended in 10 ml DMEM. Cells were centrifuged at 1,000*g* at 4 °C for 5 min. The pellet was washed twice with 10 ml of ice-cold PBS, resuspended in 900 µl of hypotonic buffer at 0.1× (HypoB, 3.5 mM Tris-HCl, pH 7.8, 2.5 mM NaCl, 0.5 mM MgCl_2_ supplemented with phosphatase/protease inhibitor cocktail; Thermo Scientific, 1861281) and incubated on ice for 1 h. Cells were homogenized using a 3-ml glass-Teflon Potter–Elvehjem homogenizer (100 strokes) and 100 µl of hypertonic buffer at 10× (HyperB, 350 mM Tris-HCl, pH 7.8, 250 mM NaCl, 50 mM MgCl_2_) were added to the suspension to obtain an isotonic environment. The suspension was gently mixed and transferred into a 2-ml microcentrifuge tube using a glass Pasteur pipette and the sample was centrifuged at 1,000*g* at 4 °C for 5 min to separate cells, debris and nuclei. The supernatant was referred to as Cyto1. The pellet was resuspended in 900 µl of HypoB and the same procedure was repeated. The supernatant was referred to as Cyto2. Cyto1 and Cyto2 fractions were pooled and centrifuged at 1,000*g* at 4 °C for 5 min to separate debris and the supernatant was collected and transferred into a 2 ml tube. After centrifugation at 15,000*g* at 4 °C for 2 min, the pellet (lysosomal fraction), referred to as L, was resuspended in 200 µl of suspension buffer (10 mM HEPES, pH 7.4, 150 mM NaCl supplemented with phosphatase/protease inhibitor). The resulting supernatant (cytosolic fraction) was transferred in a 2-ml microcentrifuge tube and centrifuged at 17,000*g* at 4 °C for 20 min and the supernatant (cytosolic fraction) was marked as C. For both mouse and HepG2-derived samples, 50 µl of L, C or EL fractions was dispensed in a 0.250-ml microcentrifuge tube, supplemented with 5 µl 100 mM glycine (10 mM final concentration) or vehicle (water) and sealed with parafilm. Samples were incubated at 37 °C, for 1 h in an orbital shaker (450 rpm) and, after the incubation, the samples received 50 µl 1 M NaOH (500 mM final concentration; dispensed with an insulin syringe by punching the lid of the microcentrifuge tube, without opening the tube).

Lysosome disruption was achieved by exposing the L fraction to five cycles of a 30-s pulse followed by a 30-s pause on ice (using an ultrasonic bath sonicator), followed by three cycles of freezing (10 min at −20 °C) and thawing (3 min at 37 °C). Lysosomal disruption was confirmed by electron microscopy^62^. Briefly, isolated lysosomes (intact or disrupted) were fixed in 2.5% glutaraldehyde (Polysciences, glutaraldehyde EM grade 25%, 01909-100) in PBS for at least 1 h at room temperature, before centrifugation in a 1.5-ml microcentrifuge tube in an Eppendorf centrifuge 5430R at 14,000 rpm (rotor: FA-45-30-11) for 10 min at room temperature to get a visible pellet. After discarding the supernatant, the pellet was resuspended and post-fixed in 2% osmium tetroxide in H_2_O for 60 min at 4 °C. After centrifugation (same conditions as described above), the pellet was embedded into epon (Sigma-Aldrich; epoxy embedding medium, 45345-250ML-F; epoxy embedding medium hardener DDSA, 45346-250ML-F; epoxy embedding medium hardener MNA, 45347-250ML-F; epoxy embedding medium accelerator DMP30, 45348-250ML-F). The samples were further processed for thin sections (90-nm thickness), before staining with lead citrate and uranyl acetate (Fluka, Lead(II) nitrate, 15334; tri-sodium citrate dihydrate, 1.06430, both from Merck). The samples were imaged with a Philips CM100 transmission electron microscope. The samples with disrupted lysosomes contained substantially decreased vesicular structures with discontinued, damaged membrane with less or no luminal content, whereas all vesicles in the intact lysosome preparation had intact membranes with luminal content. Cyanide detection was performed with ECh and LC–MS/MS methods (see below).

#### Cyanide generation by MPO or PXDN isolated enzymes

MPO (200 ng per well) or PXDN (500 ng per well) cyanogenic activity assays were performed in a 50 mM phosphate-citrate buffer, pH 4.5, or PBS buffer, pH 7.4, containing 1 mM glycine, 1 mM H_2_O_2_ and 150 mM NaCl, in a final volume of 50 µl. The assay mixture was incubated for 30 min at 37 °C and the reaction was stopped by the addition of 50 µl of 1 M NaOH. Cyanide production was measured with the ECh method (see below).

#### Non-enzymatic cyanide generation

Equimolar concentrations of HOCl and glycine (10 mM) or various proteinogenic amino acids, in 50 mM phosphate-citrate buffer, pH 3.5–5.0, were incubated in 0.25-ml microcentrifuge tubes, in a final volume of 50 µl and sealed with a rubber cap. The assay mixture was incubated for 10 min at room temperature and the reaction was stopped by the addition of 50 µl of 1 M NaOH with a Hamilton syringe (without removing the cap). Cyanide production was measured with the ECh method (see below).

#### ECh method for cyanide detection

Cyanide levels (detected in its CN^−^ form) were measured using a CN^−^ selective electrode (Lazar Research Labs, LIS-146CNCM-XS micro ion)^9,63–65^. Before the measurement, samples were prepared by diluting them 1:1 (vol/vol) in 1 M NaOH (0.5 M, final concentration), followed by incubation at room temperature for 30 min, thus inducing the partition of HCN to CN^−^. The electrode was fully soaked within the sample and voltage (mV) was acquired until the signal was stabilized. The value of the blank sample was subtracted from all measurements. Cyanide concentration was calculated against a KCN standard curve; absolute cyanide concentrations were typically in the range of 1–20 µM. Data were normalized to total milligrams of protein obtained using the BCA method (Thermo Scientific, 23225) for cells and lysosomes, or total wet weight for mouse tissues and expressed as cyanide generation rate (nanomoles of cyanide per milligram of protein per hour for cells and lysosomes or nanomoles of cyanide per milligram of tissue per hour for wet tissues).

#### Cyanide measurement using the Cyanalyzer LC–MS/MS method

All solvents used were LC/MS grade. All reagents used were analytical standard grade. KCN, NaOH, sulfuric acid, ammonium formate, KH_2_PO_4_ and K_2_HPO_4_ were from Fisher Scientific). Naphthalene-2,3-dicarboxaldehyde (NDA) was from TCI America, and 2-aminoethane sulfonic acid (taurine) and sodium metaborate tetrahydrate were from Alfa Aesar. Phosphate borate buffer (50 mM; pH 8.5) and NaOH (10 mM) were prepared in deionized water and transferred into plastic containers for benchtop storage. The NDA stock solution (2 mM) was prepared in 50 mM phosphate borate buffer and 40% methanol and stored in an amber vial at room temperature. Taurine (100 mM) solution was prepared in 50 mM phosphate borate buffer and stored at room temperature. H_2_SO_4_ (2 M) was prepared in deionized water and 50% ethanol and stored at room temperature. The calibration standards were prepared from an aqueous KCN stock solution (10 mM). All the calibration standards for cyanide (0.5–200 μM) were prepared in PBS (for tissue samples) or whole blood (for blood samples).

For the measurement of cyanide generation from liver homogenates, glycine (7.5 µl of 200 mM aqueous solution) or its vehicle was added to 142.5 µl sample in a 0.5-ml screw-cap vial. The vial was capped, vortexed and incubated at 37 °C for 20 h in a benchtop Fisher Scientific Isotemp incubator. After incubation, 25 μl was removed from the vial and used for the analysis. Quantification of cyanide proceeded via active microdiffusion, chemical modification of HCN using NDA and taurine, and LC–MS/MS analysis of the CN–NDA–taurine compound^10,34,66,67^. Briefly, NDA (2 mM), taurine (100 mM) and NaOH (10 mM), 200 µl each, were added to the reagent chamber of a two-chamber sample preparation cartridge, which allows active airflow from the sample chamber to the reagent chamber. Homogenate sample or mouse blood (25 µl) was placed in the sample chamber and diluted with 80 µl of deionized water. H_2_SO_4_ (200 µl of a 2 M aqueous solution) was added to the sample chamber to ensure that cyanide was in the gaseous (HCN) form. The sample and reagent chambers were immediately capped. Carrier gas (that is, room air at 200 ml min^−1^) was pumped through the sample chamber into the capture chamber to transfer the HCN gas to the capture solution. In the capture chamber, the NDA and taurine reacted with HCN to form a CN–NDA–taurine complex. An aliquot (250 µl) of the cyanide capture chamber solution was filtered with a 0.22-μm polytetrafluoroethylene membrane into a 300-μl glass insert placed in a 2-ml high-performance liquid chromatography (HPLC) vial for subsequent HPLC–MS/MS analysis. Prepared samples were analysed using HPLC (LC20AD, Shimadzu). Separation was achieved by reversed-phase chromatography using a ZORBAX RRHT Eclipse Plus C18 column (100 × 3.0 mm, 1.8 μm, 95 Å). Each chromatographic analysis was carried out using 10 mM aqueous ammonium formate (mobile phase A) and 10 mM ammonium formate in methanol (mobile phase B). An aliquot (10 μl, injection volume) of the prepared sample was separated with gradient elution at a flow rate of 0.3 ml min^−1^ and column temperature of 40 °C as follows: the column was initially equilibrated with 50% mobile phase B, linearly increased to 100% mobile phase B over 3 min, maintained at 100% B for 1 min, and then decreased linearly back to 50% mobile phase B over 1 min, where the mobile phase composition was held constant for 2 min to re-equilibrate the column between samples. A Sciex 5500 Q-trap MS/MS (Applied Biosystems) with multiple reaction monitoring was used to detect the CN–NDA–taurine complex using electrospray ionization operated in negative ion mode. Nitrogen (20 psi) was used as the curtain gas. The ion source was operated at −4,500 V, a temperature of 500 °C and a pressure of 10 psi and 0 psi for nebulizer and drying gases, respectively. The *m/z* ratio of 298.6 corresponds to the molecular ion of the CN–NDA–taurine complex. The transitions 298.6 → 190.7 and 298.6 → 80.9 were used as the quantification and identification transitions, respectively. The collision cell was operated with a medium collision gas pressure and an entrance potential of −10 V, a declustering potential of −70.0 V, a collision energy of −25.0 V, a collision exit potential of −19.0 V and a dwell time of 100 ms. Blank values were subtracted from all measured values. HCN concentration was calculated against the standard curve; absolute concentrations measured were typically in the range of 30–300 nM. Data were normalized to total milligrams of protein measured with the BCA method (Thermo Scientific).

#### Cyanide measurement using an MCC-based spectrophotometric method

Cyanide was measured using a spectrophotometric method that exploits absorption changes that occur on conversion of MCC to dicyano-cobinamide at 366 nm (ref. ^68^). The reactions were carried out in a modified 96-well deep-well plate, with a communication bridge between adjacent wells and covered by a silicone mat. This creates a closed system enabling gas exchange between the two connected wells. The polypropylene 2-ml deep-well 96-well plates with square wells (J.T.Baker/Avantor, 43001-0020) were manually modified using a Dremel tool to connect two horizontally adjacent wells by a small hole ~2–4 mm in diameter in a dividing wall around 3 mm from the top edge. Wells on the left served to carry out the activity assay, while wells on the right contained 200 µl of 100 µM MCC in 100 mM NaOH. The plate was made airtight using a silicone mat (J.T.Baker/Avantor, 43001-0022). To stop the reaction and for a complete volatilization of HCN from enzymatic reactions, 500 µl of 0.5 M H_2_SO_4_ was injected into the wells on the left using a syringe equipped with a 26-gauge needle via a self-sealing mat and the plate was incubated at 37 °C overnight. On the next morning, the mat was carefully removed, MCC solution from wells on the right was transferred into a clear polystyrene 96-well plate and read at 366 nm using a Spectramax M5 plate reader (Molecular Devices). HCN concentrations were calculated against a standard curve, and the absolute concentrations measured were typically in the range of 30–100 nM. Data were normalized to total wet weight of the tissues.

### Confocal microscopy

#### Live cell imaging

HepG2 cells were seeded on poly-l-ornithine-coated glass-bottom microscopy chamber slides at a density 300,000 cells per well and incubated overnight. Human primary hepatocytes were seeded (in plating medium) on collagen-coated glass-bottom microscopy chamber slides at a density 150,000 cells per well and incubated for 6 h. Human primary hepatocytes (in maintenance medium) and HepG2 cells were treated with 10 mM glycine for 24 h and 10 µM THC for 3 h. Before the experiment, cells were incubated for 1 h with 10 µM of the fluorescence HCN probe CSP, a spiropyrane derivative of cyanobiphenyl^14^, which undergoes a turn-on fluorescence in the presence of HCN (excitation 405 nm, emission 495 nm) or the fluorescence HCN/HOCl probe Chemosensor P (2-amino-3-((5-(benzothiazol-2-yl)-2-hydroxybenzylidene)amino) maleonitrile)^15^, which undergoes a sharp turn-on fluorescence in the presence of HCN (excitation 405 nm/emission 584–620 nm) or in the presence of HOCl (excitation 405 nm/emission 450–550 nm). For colocalization experiments, cells were also incubated together with cell-permeant dyes (50 nM LysoTrackerGreen, Thermo Fisher Scientific, L7526; 10 µM calcein, AM, Thermo Fisher Scientific, C3100; 1 µM CellMask Green Actin Tracking Stain, Thermo Fisher Scientific, A57247; or 200 nM MitoTracker Deep Red FM, Thermo Fisher Scientific, M22426) for 30 min at 37 °C and 5% CO_2_. At the end of the incubation, cells were washed three times and visualized using confocal microscope Leica SP5 or Leica 8 Stellaris Falcon using a ×40 oil-immersion APO Plan objective. The following excitation and emission spectra were used: LysoTrackerGreen (excitation 488 nm/emission 517 nm), calcein, AM (excitation 488 nm/emission 517 nm), CellMask Plasma Membrane Stain (excitation 488 nm/emission 535 nm) and MitoTrackerDeepRed (excitation 644 nm/emission 665 nm). The parameters of acquisition were: image format of 1,024 × 1,024 pixels, 200 Hz scan speed. Cell fluorescence was measured using ImageJ with the CTCF formula where CTCF is defined as: integrated density − (area of selected cell × mean fluorescence of background reading).

#### MPO localization and subcellular organelles

HepG2 cells were seeded on poly-l-ornithine-coated cover glass in a 12-well plate at a density 100,000 cells per well and incubated with the appropriate medium until 40–60% confluence was reached. Cells were loaded with 500 nM ER tracker green for 30 min. Cells were then washed twice with pre-warmed 0.1 M Tris-buffered saline (TBS), fixed with pre-warmed 4% paraformaldehyde solution for 2 min at 37 °C and incubated in blocking buffer (0.1 M TBS containing 10% donkey serum) for 60 min at room temperature. Primary anti-MPO (rabbit, 1:10,000 dilution, Sigma-Aldrich, HPA021147) was added and incubated overnight at 4 °C. The following day, cells were washed three times with TBS and incubated with the appropriate secondary antibody (goat anti-rabbit IgG (H+L) Highly Cross-Adsorbed Secondary Antibody Alexa Fluor Plus 647; 1:1,000 dilution), added for 1 h at room temperature. DAPI (Molecular Probes, 5 µg ml^−1^, Thermo Fisher Scientific, D1306) was added for the last 5 min. Cells were then washed three times and covered with a coverslip using Prolong Gold antifade reagent (Thermo Fisher Scientific, P36930) and visualized using a Leica SP5 or Leica 8 STELLARIS Falcon at ×63 magnification. ERtracker and MPO were visualized at an excitation and emission of 504 nm and 511 nm and 647 nm and 665 nm, respectively.

For experiments with MitoTracker Deep Red, cells were loaded with 200 nM MitoTracker Deep Red FM and fixed with 4% paraformaldehyde for 15 min at 37 °C and incubated in blocking buffer (0.1 M TBS containing 10% donkey serum) for 60 min at room temperature. Primary anti-MPO (rabbit, 1:10,000 dilution, Sigma-Aldrich, HPA021147) was added and incubated overnight at 4 °C. The following day, cells were washed three times with TBS and incubated with the appropriate secondary antibody (goat anti-rabbit IgG (H + L) Highly Cross-Adsorbed Secondary Antibody Alexa Fluor Plus 568, 1:1,000 dilution; anti-mouse Alexa Fluor 568, Thermo Fisher Scientific, A-11004), added for 1 h at room temperature. DAPI (Molecular Probes, 5 µg ml^−1^, Thermo Fisher Scientific, D1306) was added for the last 5 min. Cells were then washed three times and covered with a coverslip using Prolong Gold antifade reagent (Thermo Fisher Scientific, P36930) and visualized using a Leica 8 STELLARIS Falcon at ×63 magnification. MitoTracker and MPO were visualized at an excitation and emission of 647 nm and 665 nm and 568 nm and 603 nm, respectively.

### Proteomics and detection of *S*-cyanylation by LC–MS/MS

#### Mouse liver tissue treated with 10 mM glycine or vehicle

Mouse liver samples (50 mg each) were obtained frozen at −80 °C. Livers were cut in half using a scalpel and forceps and weighed on a scale. Tissues were added to a Potter homogenizer with 2 ml of PBS (pH 7.4) and homogenized until completely broken and mixed with PBS. Then 1 ml of the sample was transferred to a 5-ml microcentrifuge tube to which 1 ml of 20 mM buffered glycine (Sigma-Aldrich) was added. Into the other half of the sample, 1 ml of PBS was added. In each sample, protease inhibitor was added to a volume of 1%. Samples were placed in a six-well plate and incubated (37 °C, 5% CO_2_) for 8 h. Samples were transferred to 5-ml microcentrifuge tubes and 1 ml of 2× HEN was added to each (100 mM HEPES, 2 mM EDTA, 0.2 mM neocuproine, 2% IGEPAL, 4% SDS, pH 7.4) with 2% protease inhibitor and 40 mM iodoacetamide. Samples were lysed using a handheld homogenizer and precipitated using the methanol/chloroform method. Pellets were left to dry overnight. Dry samples were resuspended using 50 µl 50 mM ABC, 6 M guanidine HCl. Next, 20 µg of protein was diluted 20 times with 50 mM ABC (no guanidine) and trypsin was added with a 1:20 trypsin-to-sample ratio (Promega, V5117). Proteins were digested overnight at 37 °C. The desalting was performed on Oasis HLB 1-cc Vac Cartridges, 30 mg sorbent (Waters, WAT094225). Columns were washed with the elution solvent 60% acetonitrile in 0.1% trifluoroacetic acid (TFA) and then twice with 0.1% TFA. Samples were then loaded on the column by gravity flow. Cartridges were washed once with 1 ml 0.1% TFA, followed by gravity flow elution two times with the elution solvent. Desalted samples were evaporated under vacuum until dryness. Dry samples were resuspended in 40 µl 0.1% TFA, and quality control was performed as described^69^ using an Ultimate 3000 Nano ultra high-pressure chromatography (UPLC) system with a PepSwift Monolithic Trap 200 µm × 5 mm (Thermo Fisher Scientific). Peptides were analysed on high-resolution LC–MS/MS using an Ultimate 3000 Nano UPLC system (Thermo Fisher Scientific) coupled to a timsTOF Pro (Bruker) equipped with a CaptiveSpray source. Peptide separation was carried out with an Acclaim PepMap 100 C18 column (Thermo Fisher Scientific) using a 150-min linear gradient from 3% to 42% of B (84% acetonitrile, 0.1% formic acid) at a flow rate of 250 nl min^−1^. Data were evaluated with PEAKS ONLINE software (version 12) using 20 ppm for precursor mass tolerance, 0.05 Da for fragment mass tolerance, specific tryptic digest, and a maximum of three missed cleavages. Carbamidomethylation (+57.021464 Da) on C, N-term acetylation (+42.010565 Da), methionine oxidation (+15.994915 Da) and on cyanylated peptides only Cyano PTM (+24.995249 Da) were added as variable modifications. Peptide spectrum match (PSM) and proteins were filtered at an FDR of 1%.

For the experiments with heavy glycine (^13^C,^15^N-labelled, Sigma-Aldrich), lysis of liver samples was performed as desribed above and one half of the sample was treated with heavy glycine instead of light. Once tryptic digested peptides were generated, as described, we performed Zn^2+^-catalysed click chemistry. Briefly, dried peptides were resuspended in 50 mM HEPES (pH 7.4) then isopropanol was added to 10% final concentration, samples were vortexed for 1 min, followed by addition of NaN_3_ (50 µM final) and ZnCl_2_ (100 µM final) and incubated for 2 h at 37 °C. Samples were then desalted, dried and resuspended in 0.1% TFA. The remaining steps were performed as described above. Data were evaluated with PEAKS ONLINE software using 20 ppm for precursor mass tolerance, 0.05 Da for fragment mass tolerance, specific tryptic digest and a maximum of three missed cleavages. Carbamidomethylation (+57.021464 Da) of cysteines, N-term acetylation (+42.010565 Da) and methionine oxidation (+15.994915 Da) were used as variable modifications. Light and heavy tetrazole modifications on cysteine were also added as variable modifications (+68.01175 and +70.01214, respectively). PSM and proteins were filtered at an FDR of 1%.

#### HepG2 cells treated with 10 mM glycine and serine/glycine-free medium

HepG2 cells (500,000 per well) were seeded in six-well plates and incubated overnight at 37 °C and 5% CO_2_. The day after cells were treated with 10 mM glycine, −Ser/Gly medium and control and further incubated for 24 h. After the incubation, cells were washed in ice-cold PBS and lysed in lysis buffer (CelLytic M, Sigma-Aldrich, C2978) supplemented with 1% protease inhibitor and 20 mM iodoacetamide. Samples were incubated at 37 °C for 1 h, 450 rpm. Samples were centrifuged for 10 min at maximum speed (20,000 rcf) at 4 °C, the supernatant was taken and proteins were precipitated with the methanol/chloroform method. Proteins were pelleted using the methanol/chloroform precipitation protocol. Samples were resuspended in 50 mM ABC 6 M guanidine HCl to a concentration of 6 mg ml^−1^. Their protein concentration was determined using DC protein assay (Lowry method). Around 20 µg of protein was diluted 20 times with 50 mM ABC (no guanidine) and trypsin was added with a 1:20 trypsin-to-sample ratio (Promega, V5117). Proteins were digested overnight at 37 °C. Desalting, peptide quality checking and MS run and data analysis were performed as described for liver samples.

#### HepG2 cells treated with HCN-releasing agents

HepG2 cells (500,000 per well) were seeded in six-well plates and incubated overnight at 37 °C and 5% CO_2_. The day after cells were treated with 30 µM amygdalin, 100 µM linamarin, 100 µM mandelonitrile, 1 µM KCN or 10 nM KCN and further incubated for 24 h. After the incubation, cells were washed in ice-cold PBS and lysed in HEN lysis buffer (50 mM HEPES, 1 mM EDTA, 0.1 mM neocuproine, 1% IGEPAL, 2% SDS, pH 7.4) supplemented with 1% protease inhibitor and 20 mM iodoacetamide using syringes. Samples were then left to incubate in the dark at 37 °C for 1.5 h. Samples were centrifuged for 15 min at maximum speed (20,000 rcf) at 4 °C, the supernatant was taken and proteins were precipitated with the methanol/chloroform method. Pellets were resuspended in 120 µl of 50 mM ABC 1 M guanidine HCl. Around 20 µg of protein was diluted ten times with 50 mM ABC (no guanidine) and trypsin was added with a 1:20 trypsin-to-sample ratio (Promega, V5117). Proteins were digested overnight at 37 °C. Desalting, peptide quality checking and MS run and data analysis were performed as described for liver samples.

#### Cyanylation of GAPDH or GPDH

GAPDH (0.14 mg ml^−1^ in PBS buffer) was treated with 10 µM KCN, 10 µM H_2_O_2_ and both KCN and H_2_O_2_ for 30 min at room temperature. GPDH (0.14 mg ml^−1^ in PBS buffer) was treated with 30 µM KCN, 30 µM H_2_O_2_ and both KCN and H_2_O_2_ for 30 min at room temperature. Around 1 µg of protein was diluted five times with 50 mM ABC and trypsin was added with a 1:20 trypsin-to-sample ratio (Promega, V5117). Proteins were digested for 24 h at 37 °C. The desalting was performed on Oasis HLB 1-cc Vac Cartridges, 30 mg sorbent (Waters, WAT094225). Columns were washed with the elution solvent 60% acetonitrile in 0.1% TFA and then twice with 0.1% TFA. Samples were then loaded on the column by gravity flow. Cartridges were washed once with 1 ml 0.1% TFA, followed by gravity flow elution two times with the elution solvent. Desalted samples were evaporated under vacuum until dryness. Dry samples were resuspended in 4 µl 0.1% TFA and quality control was performed using an Ultimate 3000 Nano UPLC system with a PepSwift Monolithic Trap 200 µm × 5 mm (Thermo Fisher Scientific). Peptides were analysed on high-resolution LC–MS/MS using an Ultimate 3000 Nano UPLC system (Thermo Fisher Scientific) coupled to a timsTOF Pro (Bruker) equipped with a CaptiveSpray source. Peptide separation was carried out with an Acclaim PepMap 100 C18 column (Thermo Fisher Scientific) using a 120-min linear gradient from 3% to 35% of B (84% acetonitrile, 0.1% formic acid) at a flow rate of 250 nl min^−1^. Data were evaluated with PEAKS ONLINE software using 20 ppm for precursor mass tolerance, 0.5 Da for a fragment mass tolerance, specific tryptic digest, and a maximum of three missed cleavages. Carbamidomethylation (+57.021464 Da) on C, N-term acetylation (+42.010565 Da), methionine oxidation (+15.994915 Da) and on cyanylated peptides only Cyano PTM (+24.995249 Da) were added as variable modifications. PSM and proteins were filtered at an FDR of 1%.

### Detection of protein *S*-cyanylation by SDS–PAGE

This method is based on the principle that chemical cleavage of polypeptide’s backbone occurs after *S*-cyanylation of target cysteine residues under alkaline and denaturating conditions and has been previously used to detect *S*-cyanylation of various plasma proteins^30,70,71^. Briefly, 0.14 mg ml^−1^ GAPDH or GPDH in PBS in a 20 µl PCR tube was pretreated with 1 mM KCN or 0.325 mM H_2_O_2_ (used to induce oxidation of cysteine residues) or 0.325 mM diamide (used to induce oxidation of cysteine residues specifically to disulfide) at room temperature for 30 min under constant agitation (750 rpm). Afterwards, samples were treated with 1 mM KCN or 0.325 mM H_2_O_2_ or 0.325 mM diamide and further incubated at room temperature for 30 min under constant agitation (750 rpm). The reaction mixture was alkalinized by adding NaOH, yielding a final concentration of 120 mM, and resuspended in an equal volume of 2× Laemmli buffer supplemented with 5% β-mercaptoethanol, thus inducing protein denaturation. Samples were separated on SDS–PAGE followed by Coomassie staining.

### Hypoxia and hypoxia–reoxygenation experiments

HepG2 cells were seeded into a 96-well plate at 20,000 cells per well or into a six-well plate at 500,000 cells per well and incubated at 37 °C and 5% CO_2_ overnight. To monitor the effect of glycine and HCN scavengers, the day after seeding medium was replaced with −Ser/Gly medium or with −Ser/Gly medium supplemented with 0.4 mM glycine or with −Ser/Gly medium supplemented with 10 mM glycine. Cells were moved to a hypoxic chamber (InvivO2 400, Baker Ruskinn) at 0.2% O_2_ and 5% CO_2_ and subsequently incubated for 48 h. For the last 3 h, a group of cells was treated with HCN scavengers (10 µM THC or CoE); control cells received vehicle. To monitor the effect of HCN and HCN releasers, the day after seeding the medium was replaced with medium supplemented with 10 nM KCN or with HCN releasers amygdalin (30 µM), linamarin (100 µM) or mandelonitrile (100 µM), and cells were incubated for 48 h under hypoxic conditions (0.2% O_2_). In all cases, reoxygenation was also tested by incubating cells under normoxic conditions (5% CO_2_) for an additional 24 h.

#### Cell viability assessment

After treatments and incubation, 50 µl of each well’s supernatant was transferred to another plate to test LDH activity, an indicator of cell necrosis. The LDH assay was performed using the Pierce LDH Cytotoxicity Detection Kit Plus (Roche, 04744926001). Briefly, 50 µl per well of LDH reaction mixture were added to the supernatants. The plate was incubated for 30 min at room temperature protected from light and the reaction was stopped with 25 µl per well of Stop Solution. The plate was mixed for 60 s using an orbital shaker before measuring the absorbance at 490 nm and 680 nm (background) with a Tecan Infinite 200 Pro reader.

#### Sample preparation for western blotting

After treatments and incubation, cells seeded in the six-well plate were washed with 500 µl per well PBS and, to preserve the stability of HIF-1α, cells were lysed with 200 µl LDS sample buffer (1×; Invitrogen, 2134101) supplemented with reducing agent (1×; Invitrogen, 199821). Samples were collected by scraping the wells and sonicated for 10 min (ten cycles of 30 s sonication/30 s stop at room temperature). Proteins were denatured by incubation at 95 °C for 10 min and were immediately loaded in 4–12% Bis-Tris Plus Gels (Invitrogen) and ran at a constant voltage of 120 V (see below).

### Western blot analysis

Briefly, 20 μg of total protein (from whole-cell extracts or isolated lysosomes) was separated on 4–12% Bis-Tris Plus Gels (Invitrogen) and then transferred onto a polyvinylidene difluoride membrane by a dry transfer using the iBlot 2 system (Invitrogen). After blocking with 5% non-fat milk in TBS-Tween20 (TBS-T) for 1 h, membranes were incubated with specific antibodies overnight at 4 °C. The following primary antibodies and dilutions were used: anti-HIF-1α mouse monoclonal antibody (BD Transduction Laboratories, 610958, 1:1,000 dilution), rabbit recombinant monoclonal LAMP1 antibody (Abcam, 225762, 1:1,000 dilution), anti-GAPDH rabbit polyclonal antibody (Sigma-Aldrich, ABS16, 1:1,000 dilution), anti-MPO rabbit monoclonal antibody (E1E7I; Cell Signaling Technology, 14569, 1:1,000 dilution), anti-CAT monoclonal antibody (D4P7B; Cell Signaling Technology, 12980, 1:1,000 dilution), anti-myc tag monoclonal antibody (2278; Cell Signaling Technology, 1:1,000 dilution), anti-β-actin mouse monoclonal antibody (AC-15; Sigma-Aldrich, 1:1,000 dilution), anti-PXDN rabbit polyclonal (Sigma-Aldrich, 1675, 1:1,000 dilution) and anti-TST rabbit polyclonal antibody (Abcam, 231248, 1:1,000 dilution). Rabbit polyclonal anti-MGST1 (114551), anti-GSTA1 (108012), anti-GSTA2 (55651) and anti-PRDX3 (112004) antibody were purchased from GeneTex and rabbit polyclonal anti-PRDX6 (64329) antibody (Cell Signaling) was used at a 1:1,000 dilution. After incubation, the membranes were briefly washed three times with TBS and incubated for 1 h at room temperature with secondary antibodies anti-rabbit IgG or anti-mouse IgG, HRP-linked antibody (Cell Signaling, 7076) diluted at 1:3,000 in TBS-T/5% milk. Membranes were washed twice with TBS-T and once with TBS, and the detection was performed using Radiance plus femtogram HRP substrate (Azure Biosystems, AC2103). Chemiluminescence was captured using the Azure Imaging System 300 (Azure Biosystems).

### Bioenergetic analysis in HepG2 cells

The Seahorse XFe24 flux analyzer (Agilent Technologies) was used^72^ to measure various bioenergetic parameters of HepG2 cells or dermal fibroblasts from Detroit551, G;00880, GM00747 and GM10360. HepG2 cells were seeded at a density of 20,000 cells per well on Agilent Seahorse XF24 cell culture microplates. The next day, the medium was replaced with fresh medium supplemented with 10 mM glycine or −Ser/Gly medium and further incubated for 24 h. To reduce HCN levels, cells were treated with HCN scavengers THC and CoE (10 µM) and incubated at 37 °C and 5% CO_2_ for 3 h. NKH or healthy control skin fibroblasts were incubated with vehicle or hydroxychloroquine (30 µM) for 72 h followed by seeding at a density of 5,000 cells per well on Agilent Seahorse XF24 cell culture microplates for bioenergetic measurement.

#### Mito stress test

For analysis of mitochondrial respiration, the Cell Mito Stress assay kit (Agilent, 103015-100) was used. Cells were washed twice with DMEM (pH 7.4) supplemented with 2 mM l-glutamine (Corning, 25-015-CL), 1 mM sodium pyruvate (Cytiva, SH30239.01) and 10 mM glucose (Sigma-Aldrich, G8644). The microplate was then incubated in a CO_2_-free incubator at 37 °C for 1 h, to allow temperature and pH equilibration, as recommended by the manufacturer. The assay consisted of two measurements of basal values of OCR, followed by the injection of 1 μM oligomycin, used to evaluate ATP generation rate. Subsequently, the mitochondrial oxidative phosphorylation uncoupler FCCP (0.8 μM for HepG2, 2 μM for dermal fibroblasts) was used to estimate maximal mitochondrial respiratory capacity. Eventually, 0.5 μM of rotenone and antimycin A were injected to inhibit the electron flux through the complex I and III, respectively, aiming to detect the extra-mitochondrial OCR.

#### Long-chain fatty acid oxidation stress test

For the analysis of the fatty acid oxidation pathway, the Long Chain Fatty Acids Oxidation Kit (Agilent, 103672-100) was used. Cells were washed twice with DMEM (pH 7.4) supplemented with 2 mM glucose (Sigma-Aldrich) and 0.5 mM l-carnitine. After 1 h incubation at 37 °C in CO_2_-free conditions, just before starting the assay, each well received 87.5 µl of 1 mM XF palmitate-BSA FAO substrate (Agilent, 102720-100) and etomoxir (4 µM) to reach a final volume of 500 µl per well. Measurements of basal values of OCR were followed by injection of 1 μM oligomycin, followed by FCCP (0.8 μM). Subsequently, 0.5 μM of rotenone and antimycin A was injected to inhibit the electron flux through the complex I and III, respectively, aiming to detect extra-mitochondrial OCR. Data were analysed with Wave (v.2.6; Agilent Technologies) and graphed with Prism 8 (GraphPad Software).

### Cell proliferation and viability

HepG2 cells were seeded in a sterile transparent 96-well plate at 5,000 cells per well in 100 µl of complete culture medium and incubated overnight at 37 °C and 5% CO_2_. Cell proliferation was monitored for 72 h using vis IncuCyte Live Cell Analysis^73^ (×20 objective; Essen Bioscience). Cell confluence was recorded hourly by phase contrast scanning for 72 h at 37 °C and 5% CO_2_ and calculated from the microscopy images. Data were analysed using the IncuCyte ZOOM v.2018A software.

Skin fibroblasts were seeded at a density of 5,000 cells per well in a sterile transparent 96-well plate and incubated for 72 h. Every 24 h, culture medium was replaced with fresh medium containing vehicle or 10 µM hydroxychloroquine. End point cell proliferation was assayed with an ELISA BrdU kit (Merck, 11296736001). Briefly, cells were incubated with BrdU labelling solution for 2 h at 37 °C and 5% CO_2_. After removing the culture medium, DNA denaturation and fixation were performed, followed by incubation with anti-BrdU. Subsequently, the colorimetric substrate reaction was measured. Absorbance was measured at 450 nm, with 690 nm as the reference wavelength, using a Tecan Infinite M200 Pro plate reader. To assess cell viability, following various treatments (as described above), skin fibroblasts were further incubated for 1 h with 0.5 mg ml^−1^ MTT. The converted formazan dye was dissolved in 100 µl DMSO and the absorbance was measured at 570 nm and 690 nm (reference wavelength) with the Tecan Infinite 200 Pro plate reader.

### Measurement of intracellular glycine content

Glycine concentration in homogenates of HepG2 cells or fibroblasts (control and NKH) was quantified by a fluorometric Glycine Assay Kit (Abcam, ab211100).

### GAPDH activity assay

The GAPDH Activity Assay Kit (Sigma-Aldrich, MAK277-1KT) was used to measure the enzyme activity of GAPDH after S-cyanylation. Before all experiments, GAPDH was incubated with dithiothreitol (DTT) at 20 molar excess to reduce all exposed sulfhydryl groups of cysteine residues. Excess of DTT was removed by using a PD10 desalting column pre-equilibrated in 50 mM Tris-HCl, pH 7.4. The reaction mixture, in the assay buffer provided with the kit, contained 0.55 µM GAPDH and 10 µM H_2_O_2_, 10 µM KCN or their combination, in an assay volume of 50 µl. The 96-well plate was sealed with transparent ELISA film and incubated for 30 min at room temperature. Enzymatic activity was triggered by the addition of a mixture containing substrates and developer and was monitored at 450 nm, at 37 °C for 30 min using the Tecan Infinite 200 Pro reader.

### GPDH activity assay

Cytosolic GPDH (Merck, 10127752001) was used to test GPDH enzymatic activity after *S*-cyanylation. Before all experiments, GPDH was incubated with DTT at 20 molar excess to reduce all exposed sulfhydryl groups of cysteine residues. Excess DTT was removed by using a PD10 desalting column pre-equilibrated in 50 mM Tris-HCl, pH 7.4. The reaction mixture in 50 mM Tris-HCl pH 7.4 supplemented with 1 mM EDTA contained 0.05 µM GPDH, and 10 µM H_2_O_2_, 10 µM KCN or a combination of them, in a volume of 50 µl. The 96-well plate was sealed with transparent ELISA film and incubated for 30 min at room temperature. Enzymatic activity was triggered by adding 500 µM NADH followed by 1.5 mM dihydroxyacetone phosphate and the oxidation of NADH to NAD^+^ was followed by monitoring the decrease in absorbance at 340 nm over time, at 37 °C using the Tecan Infinite 200 Pro reader.

### Metabolomics analysis

HepG2 cells were seeded into a sterile 10-cm dish at 2,000,000 cells per dish and incubated at 37 °C and 5% CO_2_. The day after, medium was replaced with the fresh medium supplemented with 10 mM glycine and further incubated for 24 h. Cells were washed twice with 10 ml of PBS and snap frozen. Metabolites were quantified by LC–MS at the Metabolomics Unit of the University of Lausanne (Switzerland).

### RNA-seq

HepG2 cells were seeded into six-well plates at 500,000 cells per well and incubated at 37 °C and 5% CO_2_. The next day, the medium was replaced with fresh medium supplemented with 10 mM glycine and further incubated for an additional 24 h and harvested for analysis. For the comparison of TST silencing or TST overexpression, cells (wild-type control, TST-OE and shTST HepG2) were incubated in normal cell culture medium containing 400 µM glycine for 24 h, washed twice and RNA extraction and RNA extraction was performed using the NucleoSpin RNA plus kit (Macherey-Nagel, 740984.250). RNA concentration and purity were determined using a Nanodrop 2000 spectrophotometer (Thermo Scientific) by measuring the absorbance at 260/280 nm. Samples were processed and analysed using next-generation sequencing at Azenta Life Sciences. Fast GSEA was performed on the complete (normalized) count data using the hallmark gene sets^74^ using the GSEA software (version 4.3.2., University of California, San Diego).

### Statistical analysis

Unless otherwise stated, data are presented as mean values ± s.e.m. of several independent experiments where an independent experiment is defined as an experiment performed on a different experimental day, representing a biological replicate (as opposed to technical replicates). Similarly, for the MS measurements, *n* refers to the number of biological replicates. No statistical methods were used to predetermine sample sizes, but our sample sizes are similar to those reported in previous publications^32,56,59,60,72^. Data distribution was assumed to be normal, but this was not formally tested. In the animal experiments, *n* represents the number of animals used per group; animals were randomly assigned to the experimental groups. Whenever it was logistically possible, data analysis was performed blind to the conditions of the experiments. For instance, in the animal experiments, the investigators quantifying infarct size or measuring organ injury markers were unaware of the designation of the samples. Likewise, the investigator measuring blood cyanide levels was not aware of the designation of the blood samples. Metabolomic analysis, RNA-seq, proteomics studies and protein cyanylation analysis were also conducted in a fully blinded manner. No animals or data points were excluded. Statistical comparison of two groups was performed using a two-sided Student’s *t*-test. In the cell-based and animal studies, statistical analysis of more than two groups performed by two-way ANOVA followed by a post hoc Bonferroni’s test to identify significant differences between two groups. These analyses were performed using GraphPad Prism 8.0. For the analysis of the MS data, all peptides were normalized using the EigenMS R package^75,76^, which aims to preserve the original differences between treatment groups while removing the bias imposed by the datasets themselves. The cyanylated peptides are then extracted from the list and normalized to the corresponding protein levels. Statistical tests were performed using Perseus^77^. Only peptides present in at least three of five biological replicates were taken in consideration. For the RNA-seq experiments, fast GSEA was performed on the complete (normalized) count data using the hallmark gene sets using the GSEA_v.4.3.2 software. For GO analysis, significant GO terms passed the Benjamini-adjusted *P*-value threshold of 0.01.

### Reporting summary

Further information on research design is available in the Nature Portfolio Reporting Summary linked to this article.

## Supplementary information

Supplementary Information Supplementary Figs. 1–4 and Tables 3 and 4.

Reporting Summary

Supplementary Table 1 Upregulated genes of the glycolytic pathway, oxidative phosphorylation pathway, fatty acid metabolism and oxidative stress pathway detected by RNA-seq analysis in HepG2 cells in response to incubation with 10 mM glycine for 24 h. This table displays results from RNA-seq analysis of the glycolytic pathway, oxidative phosphorylation pathway, fatty acid metabolism and oxidative stress pathway after a 24-h glycine treatment, including the ranking of genes based on their enrichment within the pathway. Shown for each gene are the Rank in Gene List (relative position based on enrichment), Rank Metric Score (indicating the strength and direction of association with glycine treatment), Running Enrichment Score (ES) (reflecting cumulative enrichment at each gene rank) and Core Enrichment status (genes contributing most significantly to the pathway enrichment). The increased enrichment scores highlight the activation of glycolytic genes after glycine treatment, reflecting metabolic adaptation in response to glycine exposure. Data are based on three independent replicates.

Supplementary Table 2 Upregulated and downregulated genes detected by RNA-seq analysis after TST silencing in HepG2 cells.

## Source data

Source Data Fig. 1 Statistical source data.

Source Data Fig. 1 Unmodified confocal images for Fig. 1g.

Source Data Fig. 2 Statistical source data.

Source Data Fig. 2 Electron microscopy images for Fig. 2c.

Source Data Fig. 2 Unprocessed western blots for Fig. 2g,n.

Source Data Fig. 3 Statistical source data.

Source Data Fig. 3 Unprocessed western blots for Fig. 3b,c,e.

Source Data Fig. 4 Statistical source data.

Source Data Fig. 4 Unprocessed two-dimensional SDS–PAGE for Fig. 4k,l.

Source Data Fig. 5 Statistical source data.

Source Data Fig. 6 Statistical source data.

Source Data Fig. 7 Statistical source data.

Source Data Fig. 7 Unprocessed western blots for Fig. 7b.

Source Data Fig. 7 Unmodified images for Fig. 7i.

Source Data Fig. 8 Statistical source data.

Source Data Fig. 8 Confocal images for Fig. 8a,b.

Source Data Extended Data Fig. 1 Statistical source data.

Source Data Extended Data Fig. 2 Statistical source data.

Source Data Extended Data Fig. 3 Statistical source data.

Source Data Extended Data Fig. 3 Unmodified confocal images for Extended Data Fig. 3b,c,d.

Source Data Extended Data Fig. 4 Statistical source data.

Source Data Extended Data Fig. 4 Unprocessed western blots for Extended Data Fig. 4a,b.

Source Data Extended Data Fig. 5 Statistical source data for Extended Data Fig. 5e. The statistical source data for Extended Data Fig.5a,b,c are identical to the source data for Fig. 4a (see above).

Source Data Extended Data Fig. 6 Statistical source data.

Source Data Extended Data Fig. 7 Statistical source data.

Source Data Extended Data Fig. 8 Statistical source data.

## Data Availability

All raw data were uploaded to various data depositories. All original data and images and western blots are deposited in Zenodo via 10.5281/zenodo.14610115 (ref. ^78^). RNA-seq data were deposited to the Gene Expression Omnibus under accession numbers GSE286105 and GSE286106. The mass spectrometry proteomics data are deposited in the ProteomeXchange Consortium under accession numbers PXD046184, PXD046065 and PXD046323. The oligonucleotide sequences used are listed in Supplementary Table 4. Source data are provided with this paper.
