# Supplementary material for: Regulation of mammalian cellular metabolism by endogenous cyanide production
Source: Nat Metab. 2025 Mar 3;7(3):531–55. doi: 10.1038/s42255-025-01225-w (PMC11946912; doi:10.1038/s42255-025-01225-w)
Supplement: Supplementary file 1 — Supplementary Figs. 1–4 and Tables 3 and 4. [file 42255_2025_1225_MOESM1_ESM.pdf]

nature metabolism

Supplementary information https://doi.org/10.1038/s42255-025-01225-w

Regulation of mammalian cellular metabolism by endogenous cyanide production

In the format provided by the authors and unedited

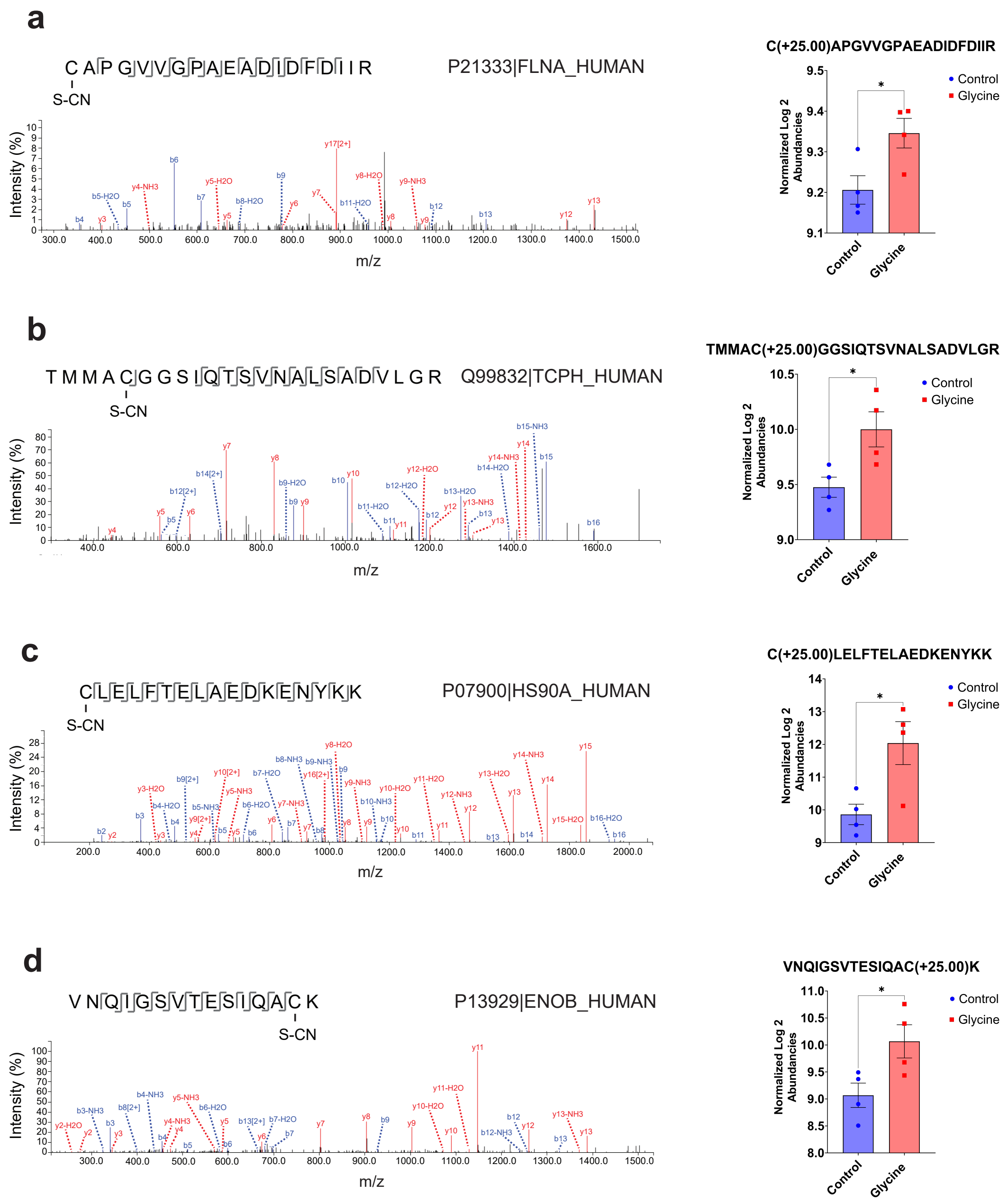

Supplementary Figure 1. Representative MS/MS fragmentation of cyanylated peptides from HepG2 proteins found to have increased cyanylation upon treatment with 10 mM Gly and corresponding quantifications. (a) C205 of filamin A (UniProt accession: P21333); (b) C326 of T-complex protein 1 subunit eta (Uniprot accession: Q99832); (c) C420 of heat shock protein HSP 90-alpha (UniProt accession: P07900); (d) C357 of beta enolase (UniProt accession: P13929).

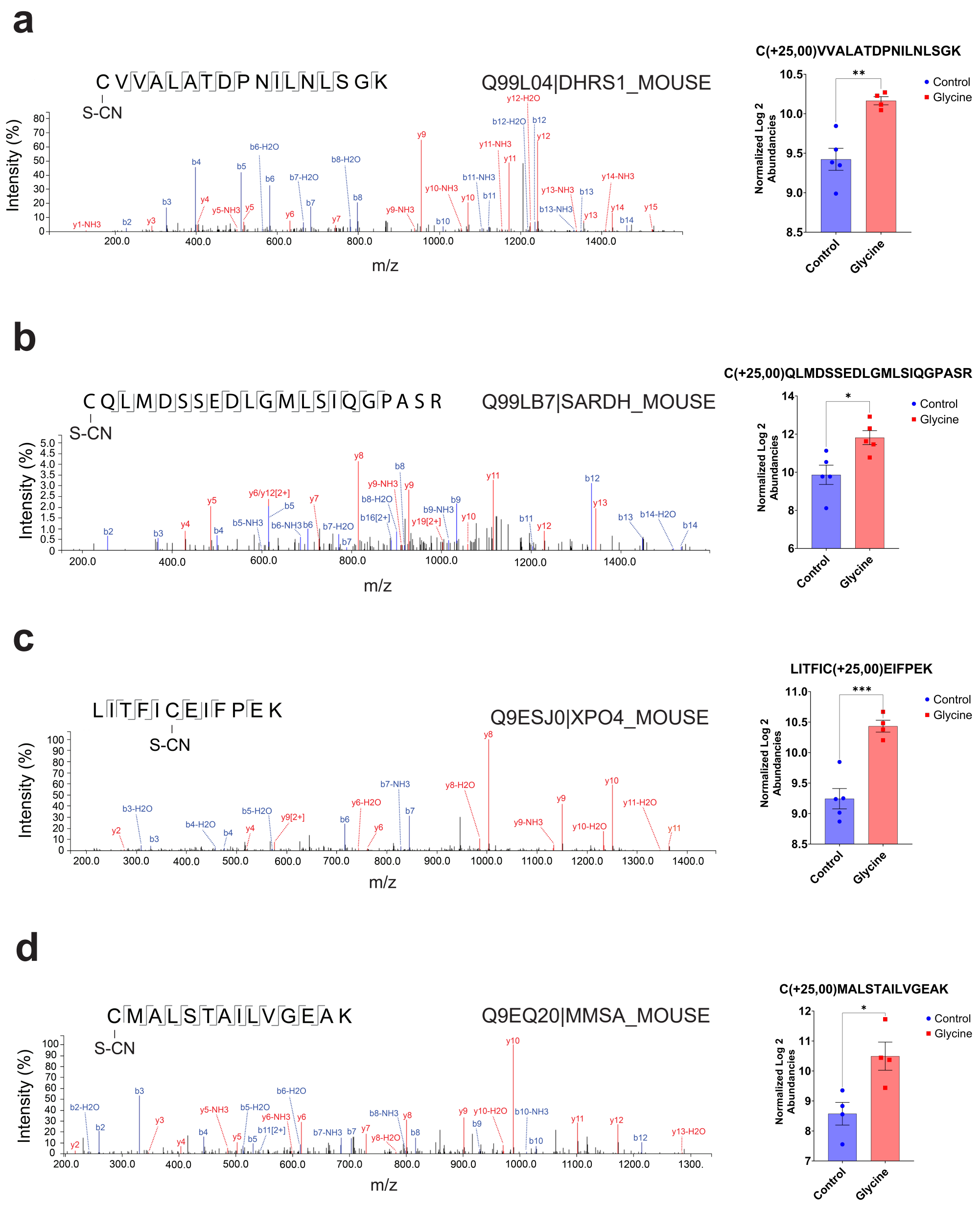

Supplementary Figure 2. Representative MS/MS fragmentation of cyanylated peptides from mouse liver proteins found to have increased cyanylation upon treatment with 10 mM Gly and corresponding quantifications. (a) C235 of dehydrogenase/reductase SDR family member 1 (UniProt accession: Q99L04); (b) C672 of sarcosine dehydrogenase, mitochondrial (UniProt accession: Q99LB7); (c) C988 of exportin 4 (UniProt accession: Q9ESJ0); (d) C317 of methylmalonate-semialdehyde/malonate-semialdehyde dehydrogenase [acylating], mitochondrial (UniProt accession: Q9EQ20)

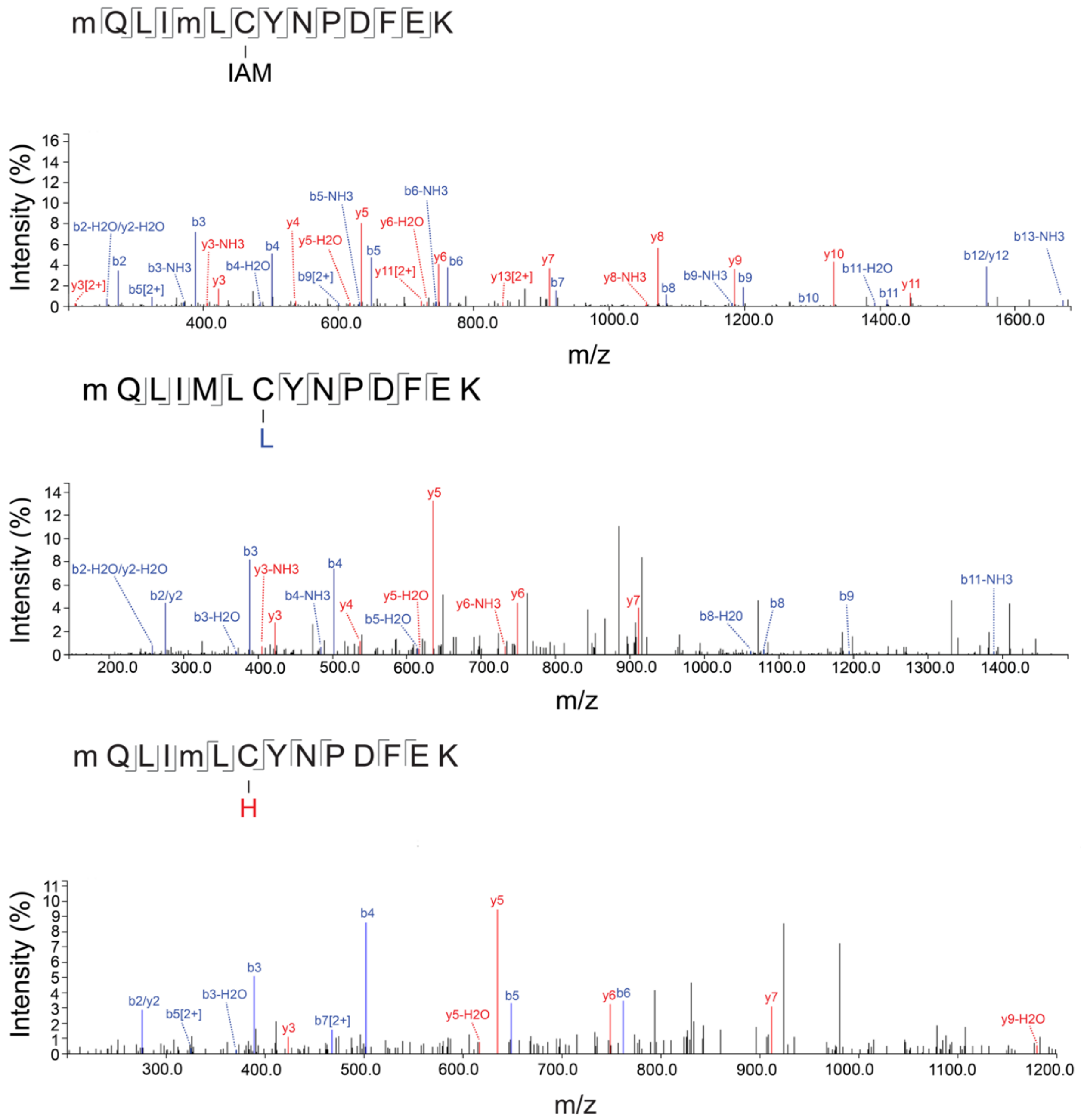

Supplementary Figure 3. Annotated MS/MS spectra of peptide from Glutathione S-transferase Mu 1 (UniProt accession: P10649) with C115 site either as carbamidomethylated (IAM) or containing light (blue L) or heavy (red H) tetrazole. Methionine oxidation is annotated with small letter “m”.

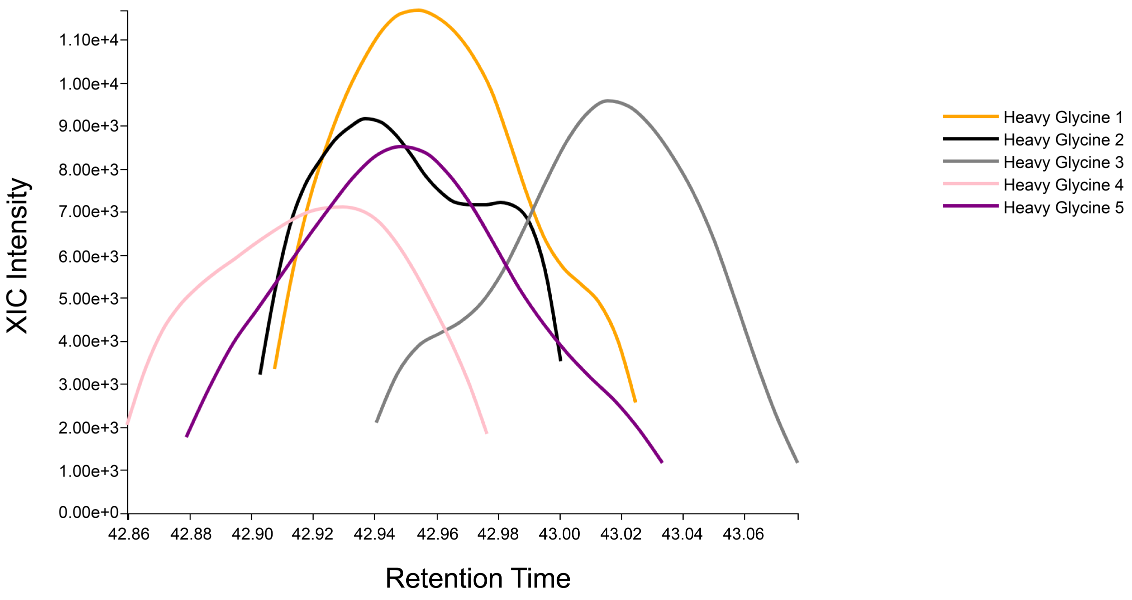

Supplementary Figure 4. Extracted ion chromatograms of peptide shown in Figure 3h.

Data presented as bar graphs in Supplemental Fig. 1a-d.

Fig 1a C(+25,00)APGVVGPAEADIDFDIIR	
Control	Glycine
9.167027473	9.397246361
9.199999809	9.341644287
9.150506973	9.244131088
9.306610107	9.400037766

Fig 1b TMMAC(+25,00)GGSIQTSVNALSADVLGR	
Control	Glycine
9.386567116	9.788232803
9.680280685	10.35743141
9.565585136	9.681835175
9.269429207	10.17213631

Fig 1c C(+25,00)LELFTELAEDKENYKK	
Control	Glycine
9.221037865	12.35801125
10.02364922	12.60267448
9.544195175	10.12223148
10.65752411	13.07194996

Fig 1d VNQIGSVTESIQAC(+25,00)K	
Control	Glycine
8.904956818	9.678217888
9.492713928	10.75815964
8.506340981	9.435761452
9.367603302	10.39586163

Data presented as bar graphs in Supplenental Fig. 2a-d.

Fig 2a C(+25,00)VVALATDPNILNLSGK	
Control	Glycine
9.348651886	10.11612892
9.384705544	nd
9.845586777	10.04615021
8.989019394	10.22787762
9.546496391	10.26991844

Fig 2b C(+25,00)QLMDSSEDLGMLSIQGPASR	
Control	Glycine
8.112476349	12.91721249
9.772930145	12.29935265
11.12843037	10.77260876
9.775575638	11.46232605
10.53450871	11.62384224

Fig 2c LITFIC(+25,00)EIFPEK	
Control	Glycine
9.015304565	10.4830904
9.238596916	nd
8.86942482	10.66940784
9.251031876	10.20317841
9.846568108	10.3786068

Fig 2d C(+25,00)MALSTAILVGEAK	
Control	Glycine
7.554414272	11.72561264
8.55654335	nd
nd	10.37265873
9.353498459	10.44212532
8.833928108	9.439948082

Supplementary Table 3. Comparison of the biological production and cellular action of the gasotransmitters nitric oxide (NO), carbon monoxide (CO), hydrogen sulfide (H_2S) and hydrogen cyanide (HCN) in mammalian cells.

	NO	*CO*	*H_2S*	*HCN*
Chemical properties	• Diffusible and labile gas; free radical	• Diffusible gas	• Diffusible and labile gas	• Diffusible and labile gas
Enzymatic and non-enzymatic sources	• iNOS, eNOS, nNOS	• HO-1 and HO-2	• CBS, CSE, 3-MST	• Various peroxidases such as MPO and PXDN<br>• Non-enzymatic generation
Biochemical reactions	• Conversion of L-arginine and O_2 into NO and citrulline, using NADPH	• Conversion of heme and O_2 into CO and biliverdin, using NADPH	• L-cysteine and homocysteine (CBS and CSE)<br>• 3-mercaptopyruvate; generation of a persulfide which decomposes into R-SH and H_2S	• pH-dependent conversion of HOCl and glycine into di-chloro-glycine which decomposes into HCl, CO_2 and HCN
Catabolism	• Reactions with globins	-	• SQR, persulfide dioxygenase and thiosulfate sulfurtransferase	• Thiosulfate sulfurtransferase (TST)<br>• Reactions with globins
Biological half-life, elimination	• Seconds<br>• Elimination via urine (nitrite & nitrate) and via exhaled air	• Hours<br>• Elimination via exhaled air	• Seconds to minutes<br>• Elimination via the urine and exhaled air	• Minutes to hours (based on toxicological studies)<br>• Elimination via urine as thiocyanate and via exhaled air
Signaling pathways	• Activation of guanylate cyclase<br>• Opening of K_{ATP} channels<br>• Post-translational protein modification via formation of S-nitrosothiols<br>• Interaction with protein metal centers	• Activation of guanylate cyclase (with lower affinity)<br>• Opening of K_{ATP} channels<br>• Interacts with protein metal centers	• Opening of K_{ATP} channels<br>• Post-translational protein modification via persulfidation<br>• Interaction with protein metal centers<br>• Inhibition of phosphodiesterase	• Activation of NMDA receptors<br>• Stimulation of calcium mobilization<br>• Post-translational protein modification via S-cyanylation<br>• Interaction with protein metal centers
Effect on bioenergetics	• Inhibitory via inhibition of cytochrome *c* oxidase activity, leading to inhibition of mitochondrial electron transport<br>• Inhibitory; after conversion to peroxynitrite	• Inhibitory via inhibition cytochrome *c* oxidase activity, leading to inhibition of mitochondrial electron transport	• Stimulatory at lower concentrations, via direct electron donation to the mitochondrial electron transport chain.<br>• Inhibitory at higher concentrations via inhibition cytochrome *c* oxidase	• Stimulatory at lower concentrations via stimulation of cytochrome *c* oxidase activity<br>• Inhibitory at higher concentrations via inhibition of cytochrome *c* oxidase activity
Cytoprotective effects	• Low concentrations (µM) yield antioxidant effects partly via activation of Nrf2<br>• Bell-shaped dose-response	• Low concentrations (µM) yield antioxidant effects partly via activation of Nrf2<br>• Bell-shaped dose-response	• Low concentrations (µM) produce antioxidant effects partly via activation of Nrf2<br>• Bell-shaped dose-response	• Very low concentrations (nM to low µM) yield cytoprotective effects<br>• Bell-shaped dose-response

Supplementary Table 4. Oligonucleotide sequences used.

<u>ShTST:</u>

Rhodanese shRNA Plasmid (h): sc-36418-SH

<u>Codon optimized PXDN with Myc tag in the N-Terminus:</u>

**ATGGAGCAGAAGCTGATCTCAGAGGAGGACCTGATGGCCAAGCGGAGCAGAGGCCCTGGCA
GACGGTGTCTGCTGGCTCTGGTGCTGTTCTGCGCTTGGGGAACCCTAGCCGTGGTAGCCCAA
AAACCTGGCGCTGGTTGTCCTAGCAGATGCCTGTGCTTCAGAACCACCGTCAGATGCATGCAC
TTGCTGCTGGAAGCCGTGCCCGCCGTGGCTCCTCAGACCAGCATCCTGGATCTCAGATTCAAT
AGAATTCGGGAAATCCAGCCAGGCGCCTTTAGGCGGCTGCGAAATCTTAACACCCTGCTGCT
GAACAACAACCAGATCAAAAGAATCCCTAGCGGTGCGTTTGAGGATCTGGAGAATCTGAAATA
CCTGTACTTGCACTTCAACCAGATCGAGACACTGGACCCCGACAGCTTCCAGCATCTGCCTAA
GCTGGAGAGACTCTTCCTGCACAACAACAGAATTACGCACCTCGTGCCTGGCACCTTTAACCA
CCTGGAATCTATGAAGCGGCTCCGGCTGGACTCTAACACACTGCACTGTGACTGCGAGATCCT
GTGGCTGGCCGACCTGCTGAAAACATACGCCGAGTCTGGAAATGCCCAGGCTGCTGCTATCT
GCGAATACCCTAGAAGAATCCAGGGCAGATCCGTGGCCACCATCACGCCTGAGGAACTGAATT
GCGAGAGACCTAGAATCACAAGCGAGCCTCAGGATGCAGACGTGACTAGCGGCAATACCGTG
TACTTCACCTGCAGAGCCGAGGGCAATCCTAAGCCTGAAATCATCTGGCTGAGAAACAACAAC
GAGCTGTCAATGAAGACTGACAGCAGACTGAACCTGCTGGACGACGGCACCCTCATGATCCA
GAATACACAGGAGACAGATCAAGGCATCTACCAGTGCATGGCCAAGAACGTGGCCGGCGAAG
TGAAAACCCAGGAGGTCACACTGAGATACTTCGGCAGCCCTGCCAGACCCACCTTCGTGATC
CAGCCCCAAAATACTGAAGTCCTGGTGGGCGAAAGCGTCACACTGGAGTGTTCCGCCACCGG
CCATCCCCCTCCTAGAATCTCCTGGACCCGAGGCGACAGGACCCCTCTCCCAGTGGACCCCA
GAGTGAACATCACCCCTAGCGGAGGACTGTACATCCAGAACGTGGTGCAAGGCGATTCTGGC
GAGTACGCCTGCAGCGCTACAAACAACATCGACTCCGTGCACGCCACAGCCTTTATCATCGTG
CAGGCCCTGCCTCAGTTCACAGTGACCCCACAGGATAGGGTGGTGATCGAGGGCCAGACAGT
GGATTTCCAGTGTGAGGCCAAGGGCAACCCGCCTCCTGTGATCGCCTGGACCAAGGGCGGAA
GCCAGCTCAGCGTCGACCGGCGCCACCTGGTGCTGAGCAGCGGCACACTGCGTATCAGTGGC
GTAGCTCTGCACGACCAGGGCCAATACGAGTGCCAGGCCGTGAACATCATCGGCTCTCAGAA
GGTGGTGGCCCACCTGACCGTGCAGCCTCGCGTCACACCAGTGTTCGCCAGCATCCCATCCG
ACACCACTGTTGAAGTCGGCGCCAACGTCCAGCTGCCTTGCAGCAGCCAGGGCGAACCCGA
GCCTGCCATCACATGGAACAAGGACGGGGTGCAGGTGACCGAAAGCGGCAAGTTCCACATCA
GCCCAGAGGGTTTCCTGACCATCAACGACGTAGGACCTGCCGACGCCGGCCGGTATGAATGT
GTGGCCAGAAACACCATCGGCAGCGCTAGCGTGAGCATGGTCCTGAGCGTGAACGTGCCCGA
TGTGTCTAGAAATGGCGACCCTTTCGTGGCCACCAGCATAGTGGAAGCCATCGCAACAGTGG
ACCGGGCCATCAACAGCACACGGACCCATCTGTTCGACAGCCGGCCTCGGTCGCCCAACGAC
CTGCTGGCCCTGTTCCGGTACCCCAGAGACCCTTACACCGTGGAACAAGCCCGGGCCGGCGA
AATCTTCGAGCGGACCCTGCAACTTATCCAGGAGCACGTGCAACACGGCCTGATGGTGGATC
TGAACGGCACCAGCTACCACTACAACGACCTGGTCTCCCCCCAGTACCTGAACCTGATCGCCA
ATCTAAGCGGCTGCACCGCCCACAGAAGAGTGAACAACTGCTCAGATATGTGCTTCCACCAGA
AGTACAGAACACACGATGGCACCTGTAACAATCTGCAGCACCCCATGTGGGGCGCCTCTCTG
ACAGCCTTCGAGCGACTGCTGAAGAGCGTGTATGAGAACGGCTTCAACACCCCTAGAGGCAT
CAACCCTCACCGGCTGTACAACGGCCACGCCCTCCCTATGCCTAGACTGGTCAGCACAACCCT
GATCGGAACCGAGACAGTGACCCCCGATGAGCAGTTCACACACATGCTGATGCAGTGGGGCC
AGTTTCTGGATCATGACCTGGACAGCACAGTGGTGGCTCTGTCTCAAGCCAGATTCAGCGAC**

GGACAGCATTGTAGCAACGTGTGCAGCAATGACCCTCCTTGTTTTCTGTGATGATTCCTCCT
AACGATTCCCGGGCTAGATCTGGGGCCAGATGCATGTTCTTCGTGCGGTCCAGCCCTGTGTGT
GGCAGCGGCATGACCTCTCTGCTGATGAACAGCGTTTACCCCAGAGAACAGATCAACCAACT
GACCTCTTATATCGACGCCTCTAACGTTTACGGCAGCACTGAACACGAGGCTCGGAGCATTCG
GGACCTGGCCAGCCACAGAGGGCTGCTCAGACAGGGAATCGTTCAGAGGTCCGGCAAACCC
CTGCTACCCTTCGCCACTGGCCCTCCTACAGAATGCATGAGAGACGAGAACGAAAGCCCTATC
CCCTGCTTTCTGGCTGGCGACCACAGAGCCAACGAGCAGCTGGGCCTGACATCTATGCACAC
CCTGTGGTTCCGGGAACACAATAGAATCGCCACCGAGCTGCTGAAGCTGAACCCCCACTGGG
ACGGCGATACCATCTACTACGAGACCAGAAAGATCGTGGGCGCCGAGATCCAGCACATCACCT
ACCAGCACTGGCTGCCTAAGATCCTGGGCGAGGTGGGAATGAGAACCCTGGGCGAGTACCAC
GGCTACGACCCCGGAATCAACGCCGGAATCTTTAACGCCTTCGCCACAGCCGCTTTCCGGTTT
GGCCACACCCTAGTGAACCCTCTGCTGTACAGACTAGACGAGAACTTCCAGCCAATCGCCCAA
GACCACCTGCCTCTGCACAAGGCCTTTTTCAGTCCTTTTAGAATCGTGAATGAAGGTGGCATC
GACCCCCTGCTGAGAGGACTGTTCGGCGTGGCCGGCAAGATGAGAGTGCCGTCACAGCTCCT
GAACACCGAACTGACCGAACGGCTGTTTAGCATGGCGCACACCGTGGCCCTGGACCTCGCCG
CCATCAATATCCAACGGGGCAGAGATCACGGCATCCCCCCCTACCACGACTACCGAGTCTACT
GCAACCTGAGCGCCGCCCACACCTTCGAGGACCTGAAGAACGAAATCAAGAATCCTGAGATC
AGAGAGAAGCTGAAAAGGCTGTACGGCAGCACACTGAACATCGACCTGTTCCCAGCCCTTGT
GGTGGAGGACCTGGTGCCTGGTAGCAGACTGGGCCCCACCCTGATGTGCCTGCTGTCCACCC
AGTTTAAGAGACTGCGCGACGGCGATAGACTGTGGTATGAAAATCCCGGAGTTTTCTCACCTG
CCCAGCTCACTCAGATCAAGCAGACCAGCCTGGCCAGAATTCTGTGCGACAACGCCGATAAC
ATCACCCGGGTGCAGTCTGATGTGTTCCGGGTGGCCGAATTCCCCCACGGATACGGAAGCTG
CGACGAGATCCCACGGGTGGATTTAAGAGTCTGGCAGGACTGCTGTGAGGACTGCAGAACCA
GAGGCCAGTTCAACGCCTTCAGCTACCACTTCAGAGGTCGAAGATCCCTGGAGTTCTCTTATC
AGGAGGATAAGCCCACCAAGAAAACAAGACCCCGGAAGATCCCTAGCGTGGGCCGGCAGGG
AGAACATCTTAGCAACTCTACCAGCGCTTTCAGCACGCGGAGTGATGCCTCTGGCACCAACGA
CTTCAGGGAGTTCGTGCTGGAGATGCAGAAGACAATCACCGACCTCCGAACACAGATCAAGA
AACTGGAGTCCCGGCTGAGCACCACCGAGTGCGTGGACGCCGGCGGCGAAAGCCACGCCAA
CAACACCAAGTGGAAGAAGGACGCTTGCACAATTTGCGAGTGTAAAGACGGTCAGGTTACAT
GCTTTGTGGAAGCTTGCCCTCCGGCCACATGCGCCGTGCCAGTGAATATCCCTGGCGCCTGTT
GCCCTGTGTGTCTGCAAAAAAGAGCGGAAGAGAAGCCT

Codon optimized MPO with Myc tag in the N-Terminus:

ATGGAGCAGAAGCTGATCTCAGAGGAGGACCTGATGGGGGTGCCTTTCTTTAGCAGTCTGAG
ATGTATGGTGGACCTCGGACCTTGTTGGGCCGGTGGTTTGACTGCTGAAATGAAACTGCTGTT
GGCACTCGCAGGACTGCTGGCTATCCTCGCTACGCCTCAGCCATCCGAAGGTGCTGCTCCCG
CGGTCCTGGGCGAGGTAGACACGTCTCTTGTCCTTAGCTCTATGGAAGAGGCCAAGCAGCTG
GTAGACAAAGCCTACAAGGAGAGACGCGAGTCCATAAAACAAAGGCTCCGGTCTGGCTCAGC
TTCTCCAATGGAACTCTTGTCATATTTCAAACAGCCCGTGGCGGCCACAAGAACTGCAGTCCG
CGCAGCAGACTACCTGCATGTTGCTCTTGATCTGCTCGAAAGGAAATTGAGAAGCCTTTGGCG
GAGGCCCTTCAATGTAACGGACGTACTCACGCCAGCTCAACTTAACGTGTTGTCCAAAAGCAG
CGGGTGCGCCTATCAGGATGTCGGTGTAACTTGTCCCGAGCAGGACAAATATAGGACGATCAC
AGGAATGTGCAACAACCGGCGGTCTCCTACGCTGGGCGCTTCAAATAGGGCATTCGTGCGAT
GGTTGCCTGCCGAGTACGAGGATGGATTCAGTTTGCCGTATGGATGGACTCCGGGAGTGAAA
CGGAATGGGTTCCCGGTAGCGTTGGCAAGAGCCGTATCTAATGAAATTGTCCGCTTTCCCACC
GACCAGCTTACACCTGATCAGGAGCGGTCACTGATGTTCATGCAGTGGGGGCAGTTGTTGGA
TCATGACCTTGATTTCACCCCAGAGCCTGCCGCAAGGGCGTCCTTCGTTACGGGAGTGAATTG
CGAAACAAGTTGTGTCCAGCAACCGCCATGCTTCCCTCTGAAGATCCCCCCAAACGATCCTAG

GATCAAAAATCAAGCCGACTGTATACCGTTTTTCCGATCTTGTCCGGCGTGTCCGGGCAGCAACATAACTATTCGCAATCAAATAAATGCTCTCACTAGCTTCGTTGATGCTTCCATGGTATATGGCTCAGAGGAACCACTTGCACGGAACTTGAGAAACATGAGCAACCAACTGGGTTTGCTGGCCGTCAACCAACGATTTCAGGACAACGGGCGGGCGCTCCTCCCCTTCGACAATCTGCATGACGACCCTTGTCTGCTGACTAACAGGTCAGCCAGGATTCCATGTTTCTCGCGGGTGATACTCGGTCCTCTGAGATGCCCGAGCTGACCTCAATGCATACTCTTCTTCTTCGCGAACATAACAGATTGGCTACAGAGCTGAAATCACTCAATCCTCGATGGGACGGTGAACGATTGTATCAGGAAGCCAGAAAAATTGTTGGTGCGATGGTACAGATCATCACTTATCGCGATTACCTTCCTCTCGTCTTGGGACCCACTGCCATGCGAAAATACCTGCCCACTTATCGATCTTATAATGATAGCGTAGATCCAAGGATTGCGAATGTTTTCACCAATGCATTCCGCTACGGGCATACTCTGATTCAACCGTTTATGTTCAGACTCGACAATAGATACCAGCCTATGGAGCCTAACCCCCGAGTTCCTTTGTCACGAGTCTTTTTTGCGTCCTGGCGAGTAGTACTGGAAGGCGGTATAGACCCTATTCTTAGAGGTTTGATGGCTACCCCTGCGAAATTGAATAGGCAGAATCAGATCGCTGTTGACGAGATAAGAGAGCGGCTCTTCGAGCAGGTTATGCGAATTGGCCTCGATCTTCCCGCGCTGAACATGCAGCGATCCCGAGACCACGGTCTTCCGGGGTACAATGCCTGGCGACGCTTCTGCGGATTGCCGCAGCCTGAAACAGTCGGGCAGCTCGGTACAGTTCTTCGCAACCTGAAGTTGGCCCGAAAATTGATGGAGCAATATGGTACACCAAACAACATAGATATATGGATGGGAGGGGTTAGCGAGCCTCTCAAAAGGAAAGGTCGGGTTGGCCATTGCTGGCTTGCATCATTGGCACCCAATTCAGGAAACTCCGAGACGGTGACCGATTCTGGTGGGAAAATGAAGGAGTGTTCAGCATGCAACAGCGACAAGCTCTCGCGCAGATATCTCTTCCTAGGATCATATGCGATAACACGGGTATAACGACTGTCAGTAAGAATAACATATTTATGTCCAATTCCTATCCCCGGGACTTTGTCAATTGTTCTACCCTCCCTGCCCTTAACCTCGCAAGCTGGAGGGAGGCCTCT

<u>Codon optimized Cyanide dihydratase with Myc tag in the N-Terminus:</u>

ATGGAGCAGAAGCTGATCTCAGAGGAGGACCTGATGACCAGCATCTACCCCAAGTTTAGAGCCGCAGCTGTGCAGGCCGCCCCTATCTACCTGAATCTGGAAGCTTCTGTGGAAAAGAGCTGCGAGCTGATCGATGAGGCCGCTTCTAATGGCGCCAAGCTGGTGGCCTTTCCAGAAGCCTTCCTGCCTGGCTACCCTTGGTTCGCCTTCATCGGCCATCCTGAGTACACCAGAAAATTCTACCACGAGCTGTATAAGAACGCCGTGGAAATCCCCAGCTTGGCTATCCAAAAGATCTCCGAGGCCGCTAAGCGGAACGAGACATACGTGTGCATCAGCTGTAGCGAGAAGGACGGCGGCAGCCTCTACCTGGCCCAGCTGTGGTTCAATCCTAACGGAGATCTGATCGGAAAGCACAGAAAGATGCGGGCCTCCGTGGCTGAGAGACTGATCTGGGGCGATGGAAGCGGCAGCATGATGCCAGTCTTTCAGACAGAGATCGGCAACCTGGGCGGCCTGATGTGCTGGGAGCACCAGGTGCCTCTGGACCTGATGGCCATGAACGCCCAAAACGAGCAGGTCCACGTGGCCAGCTGGCCTGGATATTTCGACGACGAGATTAGCTCTAGGTACTACGCCATCGCCACACAGACCTTCGTGCTGATGACATCTAGCATCTATACCGAGGAAATGAAAGAAATGATCTGCCTGACCCAGGAGCAGCGGGACTACTTCGAGACATTCAAGTCCGGCCACACCTGTATCTACGGCCCCGATGGCGAGCCCATCAGCGACATGGTGCCCGCCGAGACCGAGGGCATTGCCTACGCCGAAATCGACGTTGAAAGAGTGATCGACTACAAGTACTACATCGATCCTGCCGGCCACTACAGCAACCAGAGCCTGAGCATGAACTTCAACCAGCAACCTACCCCTGTGGTGAAGCACCTGAACCACCAGAAAAACGAAGTGTTCACCTACGAGGACATCCAGTACCAGCACGGCATCCTGGAAGAAAAAGTG

<u>Codon optimized TST with Myc tag in N-Terminus:</u>

ATGGAGCAGAAGCTGATCTCAGAGGAGGACCTGATGGTGCACCAGGTGCTGTACAGAGCCCTGGTCAGCACCAAGTGGCTGGCTGAAAGCATCAGAACCGGCAAACTGGGACCTGGCCTGAGAGTGCTGGACGCCTCTTGGTACAGCCCTGGCACCCGGGAAGCCAGAAAGGAATACCTGGAAAGACACGTTCCTGGTGCTAGCTTCTTCGACATCGAGGAATGCCGGGACACCGCCAGCCCCTACGAGATGATGCTGCCTAGCGAGGCCGGATTTGCCGAGTACGTGGGCAGACTGGGCATCAGCAACCACACACACGTGGTCGTGTATGACGGCGAGCACCTGGGATCTTTTTACGCCCCTAGAGTGTG

**GTGGATGTTCAGAGTGTTCGGCCATAGAACAGTGTCCGTGCTGAACGGCGGATTTCGGAACT
GGCTGAAAGAGGGCCACCCCGTGACCAGCGAGCCATCTAGACCTGAGCCAGCTGTTTTCAAG
GCAACCCTGGATAGAAGCCTGCTGAAGACCTACGAGCAGGTGCTGGAAAATCTGGAATCTAA
GCGGTTCCAGCTGGTGGATAGCCGGAGCCAGGGCAGGTTCCTGGGAACCGAGCCTGAACCC
GACGCCGTGGGCCTGGACAGCGGCCACATCCGGGGCGCCGTGAACATGCCTTTCATGGACTT
CCTGACAGAAGATGGCTTCGAGAAGGGCCCTGAGGAACTGAGAGCCCTCTTCCAAACAAAGA
AGGTGGACCTGAGCCAGCCTCTGATCGCCACCTGCAGAAAGGGCGTGACAGCTTGTCACGTG
GCCCTCGCCGCCTACCTGTGCGGCAAGCCTGATGTGGCCGTGTACGACGGCTCCTGGTCCGA
GTGGTTCCGGAGAGCCCCCCCGAGAGCCGCGTGTCCCAGGGCAAGAGCGAGAAAGCC**
