# Supplementary material for: Regulation of mammalian cellular metabolism by endogenous cyanide production
Source: Nat Metab. 2025 Mar 3;7(3):531–55. doi: 10.1038/s42255-025-01225-w (PMC11946912; doi:10.1038/s42255-025-01225-w)
Supplement: Supplementary file 2 — Reporting Summary [file 42255_2025_1225_MOESM2_ESM.pdf]

nature portfolio

Corresponding author(s): Szabo, Filipovic

Last updated by author(s): Jan 7, 2025

Reporting Summary

Nature Portfolio wishes to improve the reproducibility of the work that we publish. This form provides structure for consistency and transparency in reporting. For further information on Nature Portfolio policies, see our Editorial Policies and the Editorial Policy Checklist.

Statistics

For all statistical analyses, confirm that the following items are present in the figure legend, table legend, main text, or Methods section.

n/a	Confirmed	
☐	☒	The exact sample size (*n*) for each experimental group/condition, given as a discrete number and unit of measurement
☐	☒	A statement on whether measurements were taken from distinct samples or whether the same sample was measured repeatedly
☐	☒	The statistical test(s) used AND whether they are one- or two-sided<br>*Only common tests should be described solely by name; describe more complex techniques in the Methods section.*
☐	☒	A description of all covariates tested
☐	☒	A description of any assumptions or corrections, such as tests of normality and adjustment for multiple comparisons
☐	☒	A full description of the statistical parameters including central tendency (e.g. means) or other basic estimates (e.g. regression coefficient) AND variation (e.g. standard deviation) or associated estimates of uncertainty (e.g. confidence intervals)
☐	☒	For null hypothesis testing, the test statistic (e.g. *F*, *t*, *r*) with confidence intervals, effect sizes, degrees of freedom and *P* value noted<br>*Give P values as exact values whenever suitable.*
☒	☐	For Bayesian analysis, information on the choice of priors and Markov chain Monte Carlo settings
☒	☐	For hierarchical and complex designs, identification of the appropriate level for tests and full reporting of outcomes
☒	☐	Estimates of effect sizes (e.g. Cohen's *d*, Pearson's *r*), indicating how they were calculated

Our web collection on statistics for biologists contains articles on many of the points above.

Software and code

Policy information about availability of computer code

Data collection	HCN generated by cells or tissues was measured using a CN- selective electrode (Lazar Research Labs, Inc., LIS-146CNCM-XS micro ion connected to a digital millivolt meter.<br>CN-NDA-taurine complex concentrations were analyzed using a Shimadzu HPLC (LC20AD, Shimadzu Corp., Kyoto, Japan) followed by Sciex 5500 Q-trap MS/MS. Separation was achieved by reversed-phase chromatography using a ZORBAX RRHT Eclipse Plus C18 column.<br>Confocal images were obtained by a Leica SP5 or Leica 8 STELLARIS Falcon system at 63x magnification.<br>Histological images were collected by using a Leica DFC310 FX Digital Color Camera through a Nikon SMZ800 stereoscope.<br>Biochemical assays were typically run on an Infinite 200 Pro reader (Tecan).<br>Cyanylated peptides were analyzed on high-resolution LC-MS/MS using an Ultimate 3000 Nano Ultra High-Pressure Chromatography (UPLC) system (Thermo Fisher Scientific) coupled to a timsTOF Pro (Bruker) equipped with a CaptiveSpray source. Peptide separation was carried out with an Acclaim™ PepMap™ 100 C18 column.<br>Metabolomics data were obtained by employing high-end liquid chromatography-high resolution mass spectrometry (UHPLC-HRMS) and tandem mass spectrometry (MS/MS) systems.<br>Electron microscopy images were obtained with a CM100 transmission electron microscope (Philips, Eindhoven, The Netherlands).<br>Cell proliferation data were collected via the IncuCyte Live Cell Analysis device.<br>The Seahorse XFe24 flux analyzer (Agilent Technologies, Santa Clara, CA, USA) was used to estimate cellular bioenergetics of HepG2 cells.<br>Chemiluminescence was detected with the Azure Imaging System 300 (Azure Biosystems, Dublin, CA, USA).
Data analysis	Mass spectrometry data were evaluated with PEAKS ONLINE software (Version 12) using 20 ppm for precursor mass tolerance, 0.5 Da for fragment mass tolerance, specific tryptic digest, and a maximum of 3 missed cleavages.<br>The Extracellular Flux Analysis data were analyzed with Wave (version 2.6; Agilent Technologies).

For the RNA-seq experiments, fast gene set enrichment analysis (fGSEA) was performed on the complete (normalized) count data using the hallmark gene sets using the GSEA_v.4.3.2 software. For Gene Ontology analysis, significant GO terms passed the Benjamini adjusted p-value threshold of 0.01.
For the blood pressure measurements and analysis in the hemorrhagic shock study, LabChart 8.1.30 was used.
Statistical analysis was performed with Graphpad Prism 8.0. Student's t-test was used to identify significant differences between two experimental groups. For experiments with 3 or more groups, two-way ANOVA followed by post-hoc Bonferroni's multiple-comparison test was used; p<0.05 was considered statistically significant.
Schematics were created with Biorender.com

For manuscripts utilizing custom algorithms or software that are central to the research but not yet described in published literature, software must be made available to editors and reviewers. We strongly encourage code deposition in a community repository (e.g. GitHub). See the Nature Portfolio guidelines for submitting code & software for further information.

Data

Policy information about availability of data

All manuscripts must include a data availability statement. This statement should provide the following information, where applicable:

- Accession codes, unique identifiers, or web links for publicly available datasets
- A description of any restrictions on data availability
- For clinical datasets or third party data, please ensure that the statement adheres to our policy

All data that form the basis of the figures of the paper have been uploaded into the Zenodo database and are accessible at the following link: https://doi.org/10.5281/zenodo.14610115. The RNAseq and the proteomics data were uploaded into separate databases and the access numbers are provided in the paper.

Research involving human participants, their data, or biological material

Policy information about studies with human participants or human data. See also policy information about sex, gender (identity/presentation), and sexual orientation and race, ethnicity and racism.

Reporting on sex and gender	No human studies were conducted in this study.
Reporting on race, ethnicity, or other socially relevant groupings	N/A
Population characteristics	N/A
Recruitment	N/A
Ethics oversight	N/A

Note that full information on the approval of the study protocol must also be provided in the manuscript.

Field-specific reporting

Please select the one below that is the best fit for your research. If you are not sure, read the appropriate sections before making your selection.

☒ Life sciences ☐ Behavioural & social sciences ☐ Ecological, evolutionary & environmental sciences

For a reference copy of the document with all sections, see nature.com/documents/nr-reporting-summary-flat.pdf

Life sciences study design

All studies must disclose on these points even when the disclosure is negative.

Sample size	This is consistent with previous studies and accounts for biological variability between distinct samples from cell lines and it is commonly used in the literature in the fields of biochemistry and cell biology. Unless stated otherwise, the n numbers reported in the legend represent the number of experimental days and not the number of wells or replicates run in parallel on the same day. Typically, each experiment was conducted on at least n=3 technical replicates (e.g. parallel wells to study cell proliferation); these values were averaged and taken as an n=1 for the given experimental day. Vehicle treated controls and samples treated with various pharmacological agents were run in parallel on the same plate or within the same assay on each experimental day. No statistical methods were used to pre-determine sample sizes, but our sample sizes are similar to those reported in previous publications. We typically used at least 5 biological or biochemical replicates for each experiment unless stated otherwise.
Data exclusions	No data were excluded.
Replication	All experimental data were reliably reproduced in multiple independent experiments as indicated in the figure legends. The fundamental finding of the paper (CN generation from liver tissue and from isolated lysosomes and stimulating effect of glycine on CN generation) were replicated using several different methods, in several different laboratories. For example, CN generation was measured using an electrode

method (one group of investigators at UniFR in Switzerland), by several, structurally different CN-detecting fluorescent probes (another group of investigators, working in a different laboratory at UniFR Switzerland), a method based on CN release and capture by a CN scavenger (another group of investigators, working in a different laboratory at UniFR Switzerland and a derivatization mass spec method (another group of investigators, working at SDSU, USA). The phenomenon of CN generation and the stimulating effect of glycine was also replicated in vivo; these studies involved collection of mouse blood samples (University of Krakow, Poland) and analysis by another group of investigators, working at SDSU, USA. The cytoprotective effect of the CN releaser amygdalin in ischemia-reperfusion was replicated by 3 different methods: a cell-based method which utilized several structurally different CN donors as well as low concentrations of exogenous KCN (conducted by a group of investigators at UniFR in Switzerland), a model of myocardial ischemia-reperfusion in mice (conducted by a group of investigators at Athens University in Greece) and a model of whole-body ischemia-reperfusion, i.e. hemorrhagic shock in mice (conducted by a group of investigators at Columbia University in USA). The basal cyanylation of various proteins and the effect of glycine administration on protein cyanylation was demonstrated at the Leibniz Institute of Analytical Sciences, Germany, first using liver tissue, and this was replicated in HepG2 cell homogenates. Protein cyanylation was also independently replicated using a gel-based method (an independent group of investigators, working in a different laboratory at UniFR Switzerland). The effect of natural CN donors on increasing protein cyanylation was also replicated by using several different, chemically distinct molecules (Leibniz Institute of Analytical Sciences, Germany). The effect of cyanide scavenging on cellular bioenergetics and proliferation was replicated using several structurally different cyanide scavengers as well as by another approach, which involved the forced overexpression of CN-catabolizing enzymes in our cells (two different enzymes, one bacterial and one mammalian). The presence of myeloperoxidase in lysosomes was demonstrated by Western blotting analysis and the findings were subsequently replicated using confocal microscopy. The functional role of peroxidases in the process of mammalian generation was first shown by a broad-spectrum peroxidase inhibitor, and replicated using a myeloperoxidase inhibitor and by a genetic approach (comparison of CN generation in tissues from WT vs. MPO-/- or PDXN+/- mice). The stimulating effect of glycine on cellular bioenergetics and metabolism was first demonstrated by a group working in a laboratory at UniFR Switzerland using the Extracellular Flux Analysis method and the findings were independently confirmed by a subcontracted core laboratory which performed an untargeted metabolomic analysis.

Randomization

For the cellular and biochemical studies, random allocation was used. Similarly, in the in vivo studies, mice were randomly allocated into control and treated groups. All mice were sex- and age-matched and on identical genetic background.

Blinding

Whenever possible, samples were analyzed in a blinded matter. For example, mouse blood samples were collected from mice by the group in Poland, with a blinded code to SDSU in USA, where and CN levels were measured by an investigator who was unaware of the designation of the samples. The assessment of infarct size and the measurement of organ damage markers in the hemorrhagic shock study was conducted in such a way that the investigator conducting the analysis was unaware of the designation of the samples with respect to the various experimental groups. Whenever possible, treatment of cells and tissues and analysis of various outcome measures (e.g. cyanide measurement using the electrode method) was conducted by investigators who were unaware of the designation of the samples with respect to the various experimental groups. In the cell-based and biochemical assays, control and treated samples were always included in each experimental run on each experimental day and experiments were repeated several times on different experimental days, using newly seeded cells. Metabolomic analysis, RNAseq, proteomics studies and protein cyanylation analysis were conducted in a fully blinded manner.

Reporting for specific materials, systems and methods

We require information from authors about some types of materials, experimental systems and methods used in many studies. Here, indicate whether each material, system or method listed is relevant to your study. If you are not sure if a list item applies to your research, read the appropriate section before selecting a response.

Materials & experimental systems

n/a	Involved in the study
☐	☒ Antibodies
☐	☒ Eukaryotic cell lines
☒	☐ Palaeontology and archaeology
☐	☒ Animals and other organisms
☒	☐ Clinical data
☒	☐ Dual use research of concern
☒	☐ Plants

Methods

n/a	Involved in the study
☒	☐ ChIP-seq
☒	☐ Flow cytometry
☒	☐ MRI-based neuroimaging

Antibodies

Antibodies used

Primary antibodies:
1) Rabbit polyclonal anti-MPO antibody, Sigma Aldrich, Cat. No. HPA021147, Lot. No. A95491
2) Purified mouse anti-HIF-1α monoclonal antibody, BD Transduction Laboratories, Cat. No. 610958, Clone name: 54/HIF-1α (RUO), Lot. No. 7285963
3) Anti-Nrf2 rabbit polyclonal antibody, Abcam, Cat. No. ab92946, Lot. No. GR3418792-2
4) Rabbit recombinant monoclonal LAMP1 antibody, Abcam, Cat. No. ab225762, Clone name: EPR21026, Lot. No. GR3384117-4
5) Anti-GAPDH rabbit polyclonal antibody, Sigma-Aldrich, Cat. No. ABS16, Lot. No. 3790332
6) Anti-MPO rabbit monoclonal antibody, Cell Signaling Technology, Cat. No. 14569, Clone name: E1E7I, Lot. No. 1
7) Anti-catalase monoclonal antibody, Cell Signaling Technology, Cat. No. 12980, Clone name: D4P7B, Lot. No. 3
8) Mouse anti-myc tag monoclonal antibody, Cell Signaling Technology, Cat. No. 2276, Clone name: 9B11, Lot. No. 24
9) Anti-β-actin mouse monoclonal antibody, Sigma-Aldrich, Cat. No. A1978, Clone name: AC-15, Lot. No. 165998
10) Anti-PXDN rabbit polyclonal antibody, Sigma-Aldrich, Cat. No. ABS1675, Lot. No. 3761823
11) Anti-TST rabbit polyclonal antibody, Abcam, Cat. No. ab231248, Lot. No. GR3436303-1
12) Anti-MGST1 rabbit polyclonal antibody, GeneTex, Cat. No. GTX114551, Lot. No. 42109
13) Anti-GSTA1 rabbit polyclonal antibody, GeneTex, Cat. No. GTX108012, Lot. No. 39855

14) Anti-GSTA2 rabbit polyclonal antibody, GeneTex, Cat. No. GTX55651, Lot. No. 822203931
15) Anti-PRDX3 rabbit polyclonal antibody, GeneTex, Cat. No. GTX112004, Lot. No. 40093
16) Anti-PRDX6 rabbit polyclonal antibody, Cell Signaling Technology, Cat. No. 64329, Clone name: D9J9H Lot No. 1

Secondary antibodies:
1) Goat anti-rabbit IgG Highly Cross-Adsorbed polyclonal antibody, Alexa Fluor Plus 568, Thermo Fisher Scientific, Cat. No. A-11011, Lot. No. 2277758
2) Anti-mouse IgG, HRP-linked antibody, Cell Signaling, Cat. No. 7076, Lot. No. 38

Validation

Only commercially available antibodies, from major vendors, were used in this project. These antibodies have been validated by the suppliers, as listed below and have also been used by multiple independent laboratories (see the number of publications for each antibody below).
All proteins detected by antibodies used in this study showed the expected bands at the expected molecular weight.
The MPO antibody is additionally validated by the fact that in the tissues of the MPO-/- mice, the MPO band was completely absent, while in the wild-type mice a clear band was detected at the expected molecular weight.
Likewise, in the peroxidasin +/- mice, the peroxidasin showed the expected reduction at the expected molecular weight.
All antibodies used in this study were tested by the manufacturer.

Primary antibodies:
1) Rabbit anti-MPO antibody (Sigma Aldrich, Cat. No. HPA021147) can be found in 9 literature citations. The manufacturer also provides the antibody validation data: https://www.sigmaaldrich.com/CH/en/technical-documents/technical-article/protein-biology/immunohistochemistry/antibody-enhanced-validation
2) Purified mouse anti-human HIF-1α monoclonal antibody (BD Transduction Laboratories, Cat. No. 610958) can be found in 7 literature citations. The manufacturer also provides the antibody data sheet: https://www.bdbiosciences.com/en-ch/products/reagents/microscopy-imaging-reagents/immunofluorescence-reagents/purified-mouse-anti-human-hif-1.610958
3) Anti-Nrf2 rabbit polyclonal antibody (Abcam, Cat. No. ab92946 – discontinued now) can be found in 60 literature citations. The manufacturer provides the archived antibody data sheet: https://www.abcam.com/products/primary-antibodies/nrf2-antibody-ab92946.html
4) Recombinant anti-LAMP1 antibody rabbit monoclonal antibody (Abcam, Cat. No. ab225762) has been put onto the market very recently, and, so far, can only be found in 1 literature citation (Mol Neurobiol. 60: 3741, 2023). The manufacturer also provides the antibody data sheet: https://www.abcam.com/products/primary-antibodies/lamp1-antibody-epr21026-bsa-and-azide-free-ab225762.html
5) Anti-GAPDH rabbit polyclonal antibody (Sigma-Aldrich, Cat. No. ABS16) can be found in 182 literature citations. The manufacturer also provides the antibody data sheet: https://www.sigmaaldrich.com/CH/en/product/mm/abs16
6) Anti-myeloperoxidase rabbit monoclonal antibody (Cell Signaling Technology, Cat. No. 14569, Clone name: E1E7I) can be found in 14 literature citations. The manufacturer also provides the antibody data sheet: https://www.cellsignal.com/products/primary-antibodies/myeloperoxidase-e1e7i-xp-rabbit-mab/14569
7) Anti-catalase antibody (Cell Signaling Technology, Cat. No. 12980, Clone name: D4P7B) can be found in 72 literature citations. The manufacturer also provides the antibody data sheet: https://www.cellsignal.com/products/primary-antibodies/catalase-d4p7b-xp-rabbit-mab/12980
8) Anti-myc tag antibody (Cell Signaling Technology, Cat. No. 2276) can be found in 1976 literature citations. The manufacturer also provides the antibody data sheet: https://www.cellsignal.com/products/primary-antibodies/myc-tag-9b11-mouse-mab/2276
9) Anti-β-actin mouse monoclonal antibody (Sigma-Aldrich, Cat. No. A1978) can be found in 3786 literature citations. The manufacturer also provides the antibody data sheet: https://www.sigmaaldrich.com/CH/en/product/sigma/a1978
10) Anti-PXDN rabbit polyclonal antibody (Sigma-Aldrich, Cat. No. ABS1675) can be found in 4 literature citations. The manufacturer also provides the antibody data sheet: https://www.sigmaaldrich.com/CH/en/product/mm/abs1675
11) Anti-TST rabbit polyclonal antibody (Abcam, Cat. No. ab231248) can be found in 4 citations. The manufacturer also provides the antibody data sheet: https://www.abcam.com/products/primary-antibodies/tst-antibody-ab231248.html
12) Anti-MGST1 rabbit polyclonal antibody (GeneTex, Cat. No. GTX114551). The manufacturer provides the antibody data sheet: https://www.genetex.com/PDF/Download?catno=GTX114551
13) Anti-GSTA1 rabbit polyclonal antibody (GeneTex, Cat. No. GTX108012). The manufacturer provides the antibody data sheet: https://www.genetex.com/PDF/Download?catno=GTX108012
14) anti-GSTA2 rabbit polyclonal antibody (GeneTex, Cat. No. GTX55651). The manufacturer provides the antibody data sheet: https://www.genetex.com/PDF/Download?catno=GTX55651
15) Anti-PRDX3 rabbit polyclonal antibody (GeneTex, Cat. No. GTX112004) can be found in 1 literature citation (listed on the manufacturer site). The manufacturer also provides the antibody data sheet: https://www.genetex.com/PDF/Download?catno=GTX112004
16) Mouse anti-glutathione monoclonal antibody (Virogen, Cat. No. 101-A) can be found in 50 literature citations. The manufacturer also provides the antibody data sheet: https://www.virogen.com/media/101A.pdf

Secondary antibodies:
1) Goat anti-rabbit IgG highly cross-adsorbed antibody, Alexa Fluor 568, (Thermo Fisher Scientific, Cat. No. A-11011) can be found in 2703 literature citations. The manufacturer also provides antibody testing data: https://www.thermofisher.com/antibody/product/Goat-anti-Rabbit-IgG-H-L-Cross-Adsorbed-Secondary-Antibody-Polyclonal/A-11011
2) Anti-mouse IgG, HRP-linked antibody (Cell Signaling, Cat. No. 7076) can be found in 8738 literature citations. The manufacturer also provides the antibody data sheet: https://www.cellsignal.com/products/secondary-antibodies/anti-mouse-igg-hrp-linked-antibody/7076

Eukaryotic cell lines

Policy information about cell lines and Sex and Gender in Research

Cell line source(s): HepG2 hepatocellular carcinoma cells were from ATCC (HB-8065)

Cell line source(s)	Hep3B cells were from ATCC (HB-8064)<br>HL-60 cells were from ATCC (CCL-240)<br>HUVECs were from ATCC (CRL-1730)<br>A549 cells were from ATCC (CCL-185)<br>HCT116 cells were from ATCC (CCL-247)<br>HT29 cells were from ATCC (HTB-38)<br>LoVo cells were from ATCC (CCL-229)<br>U937cells were from ATCC (CRL-1593.2)<br>Human dermal fibroblasts from healthy subjects (Detroit551) were from ATCC (CCL-110) U138-MG human glioblastoma cells were from ATCC (HTB-16)<br>Cryopreserved human primary hepatocytes (from a 48-year-old Caucasian male) were from AnaBios Corporation, San Diego, CA, USA)<br>Human skin fibroblasts from NKH patients GM00880 (from a 21-year-old Caucasian male), GM00747 (from a 1.5 years-old Caucasian female), GM10360 (from a 2-months-old Caucasian male), were obtained from Coriell Institute for Medical Research (Camden, NJ, USA)
Authentication	The identity of the cell lines used in this study was verified by the supplier, ATCC. In addition, the main cell line used in the current study, HepG2 has a characteristic morphology and growth characteristic (monolayers and small aggregates), which was consistently observed and is identical to the morphological and cytological pictures available in hundreds of publications for this commonly used cell line. Moreover, the HepG2 cells grown at the University of Fribourg and used for the current project have been re-authenticated by Microsynth AG (Balgach, Switzerland).
Mycoplasma contamination	All cell lines used in our laboratory are regularly tested for mycoplasma contamination. No Mycoplasma contamination has been detected.
Commonly misidentified lines<br>(See ICLAC register)	The current project did not utilize any commonly misidentified cell lines.

Animals and other research organisms

Policy information about studies involving animals; ARRIVE guidelines recommended for reporting animal research, and Sex and Gender in Research

Laboratory animals	C57Bl6J mice were used. For the myocardial infarction study and for the hemorrhagic shock study, only wild type mice were used. For the analysis of CN generation by liver tissue, livers obtained from wild-type mice were compared with livers obtained from myeloperoxidase knockout mice (Mpo−/−; strain: #004265) and peroxidasin heterozygous mice (Pxdn+/-; strain: #042166), both on C57BL/6J background, were purchased from Jackson Laboratories. Animals were housed in a light-controlled room with a 12 h light-dark cycle and had ad libitum access to food and water. All studies were performed on 12-18 week-old mice. The room temperature for mice was 20–24°C (68–75°F) and was kept as stable as possible.
Wild animals	No wild animals were used in the study.
Reporting on sex	Sex was considered as a variable and male and female groups of animals were compared for cyanide blood levels and tissue cyanide production. In the ischemia-reperfusion and hemorrhagic shock studies, male animals were used.
Field-collected samples	No field collected samples were used in the study.
Ethics oversight	Each laboratory involved with animal studies in the current project (University of Fribourg, University of Krakow, Columbia University, University of Athens) has conducted the work under approved animal licenses. All animal experiments were performed in accordance with all applicable animal regulations and legal regulations and also in full observance of the 3R rules to reduce the number of animals used to the smallest necessary number.

Note that full information on the approval of the study protocol must also be provided in the manuscript.

Plants

Seed stocks	n/a
Novel plant genotypes	n/a
Authentication	n/a
