# Supplementary figures and images for: Regulation of mammalian cellular metabolism by endogenous cyanide production (part 1 of 2)
Source: Nat Metab. 2025 Mar 3;7(3):531–55. doi: 10.1038/s42255-025-01225-w (PMC11946912; doi:10.1038/s42255-025-01225-w)

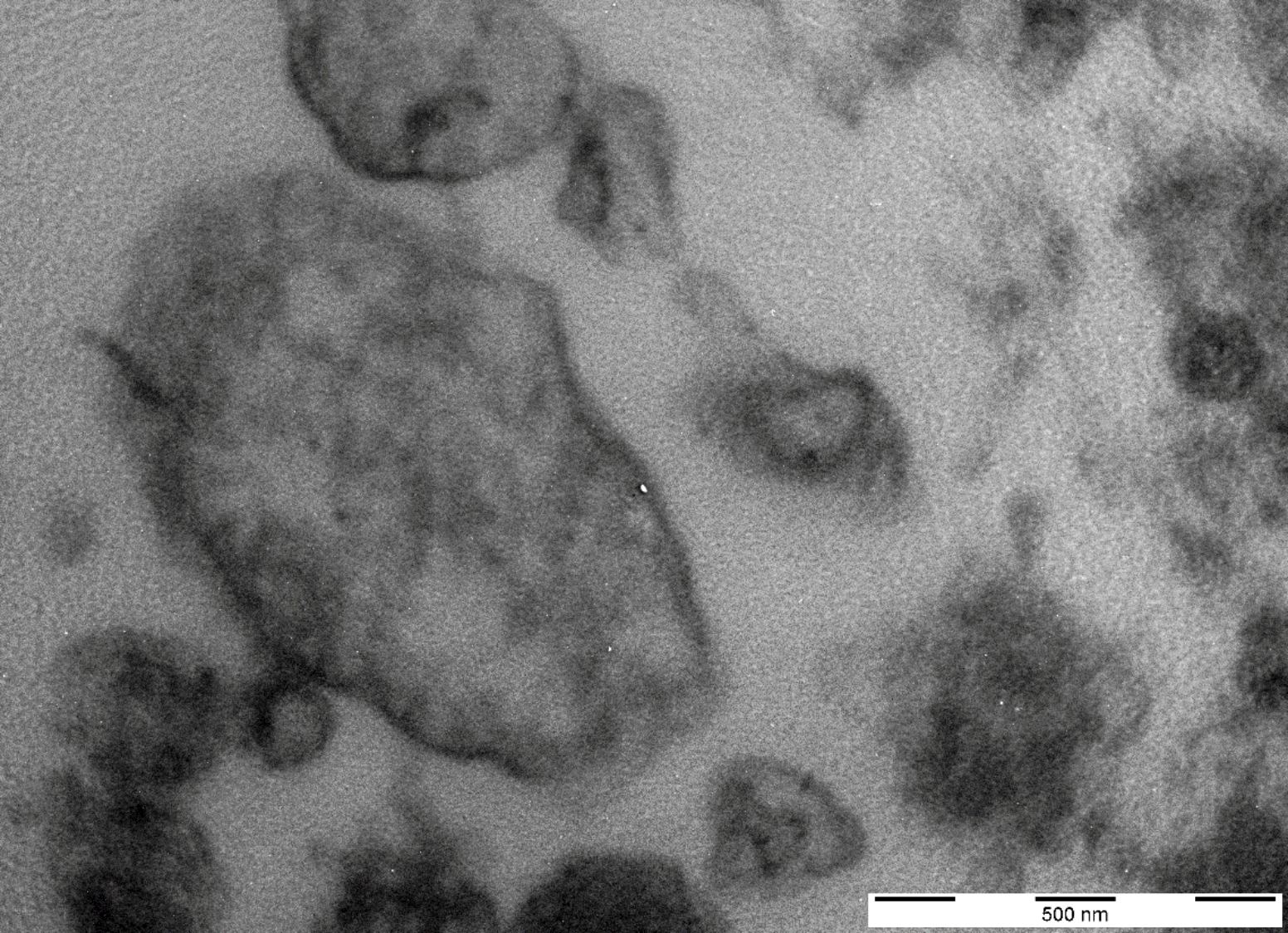

Supplement: Supplementary file 8 — Electron microscopy images for Fig. 2c. [file 42255_2025_1225_MOESM8_ESM.zip › Zuhra_Microscopy_Images_Fig2/Zuhra_EM_Main_Fig2_c/Fig2c_EM_image_lysosome_after_freeze_and_thaw.tif]

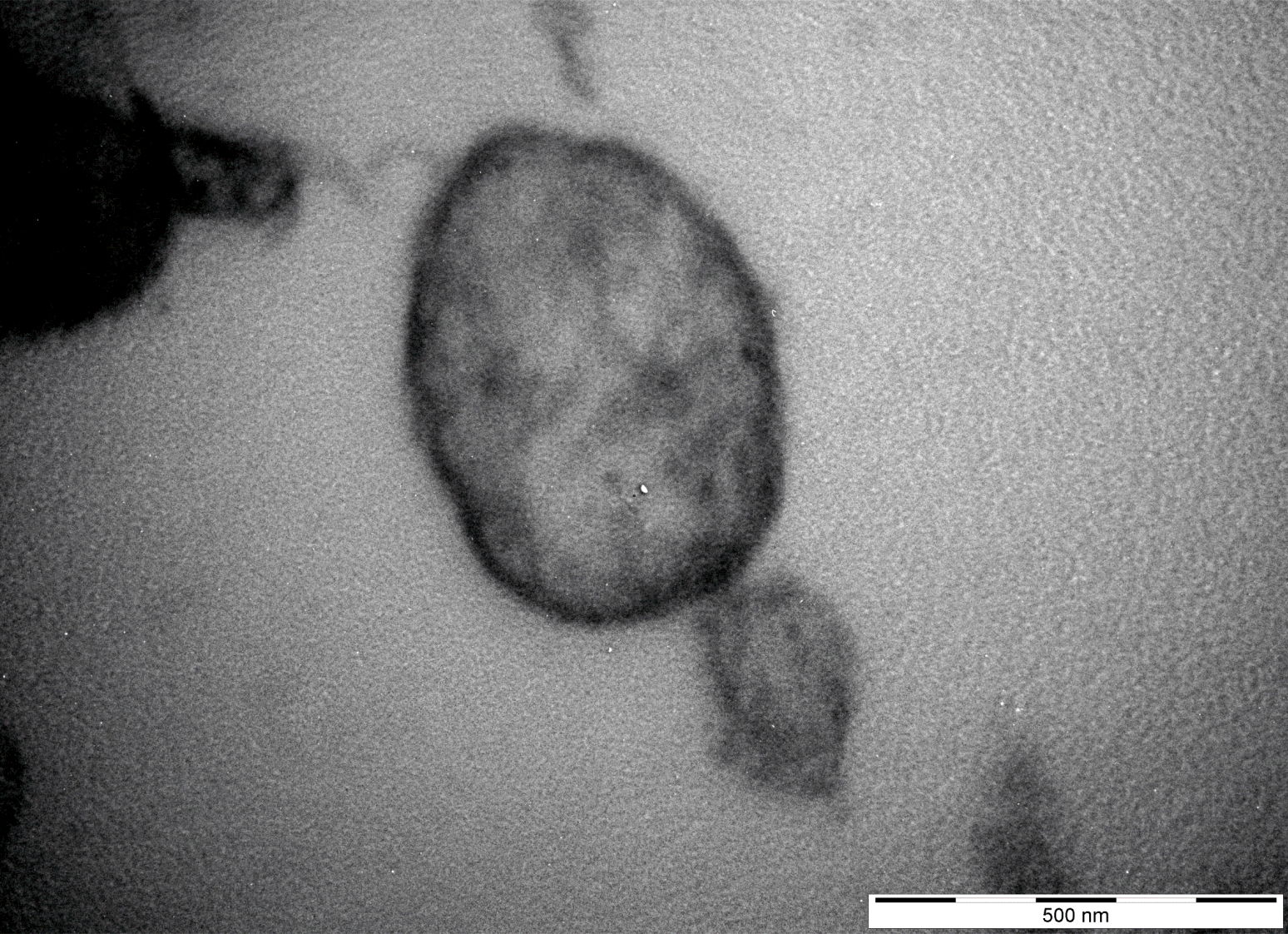

Supplement: Supplementary file 8 — Electron microscopy images for Fig. 2c. [file 42255_2025_1225_MOESM8_ESM.zip › Zuhra_Microscopy_Images_Fig2/Zuhra_EM_Main_Fig2_c/Fig2c_EM_image_lysosome_gentle_isolation.tif]

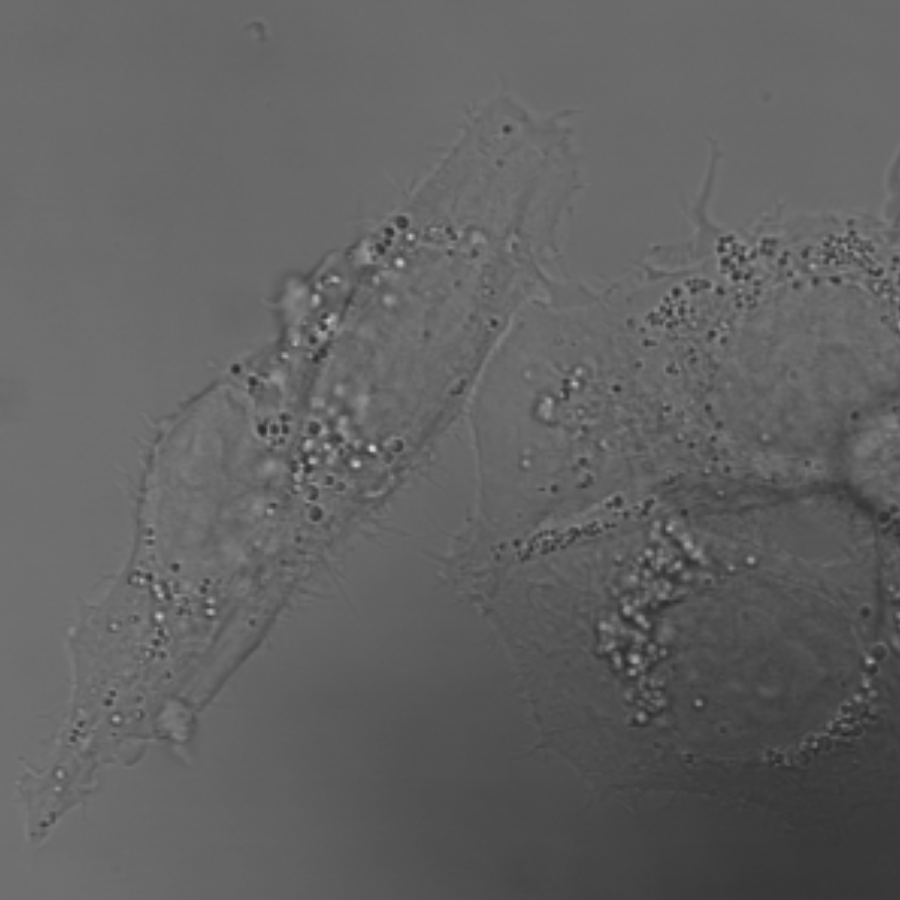

Supplement: Supplementary file 8 — Electron microscopy images for Fig. 2c. [file 42255_2025_1225_MOESM8_ESM.zip › Zuhra_Microscopy_Images_Fig2/Zuhra_Microscopy_Main_Fig2_a/Fig2a_HepG2_BF.tif]

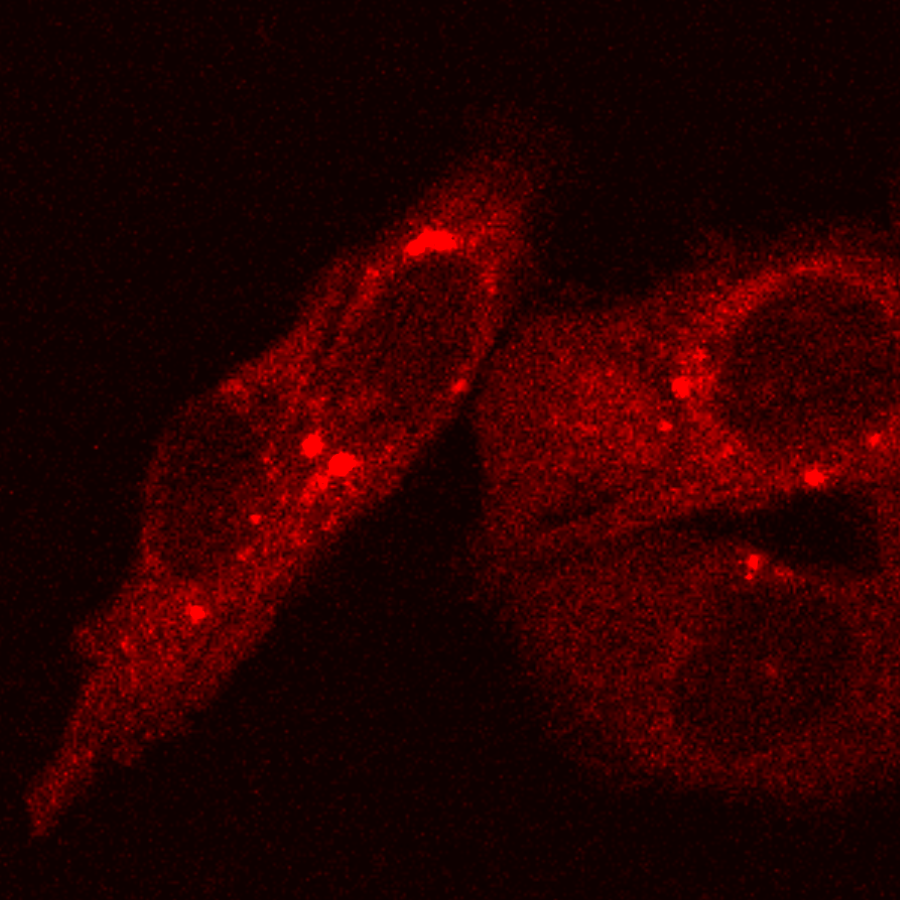

Supplement: Supplementary file 8 — Electron microscopy images for Fig. 2c. [file 42255_2025_1225_MOESM8_ESM.zip › Zuhra_Microscopy_Images_Fig2/Zuhra_Microscopy_Main_Fig2_a/Fig2a_HepG2_CyanideProbe(CP).tif]

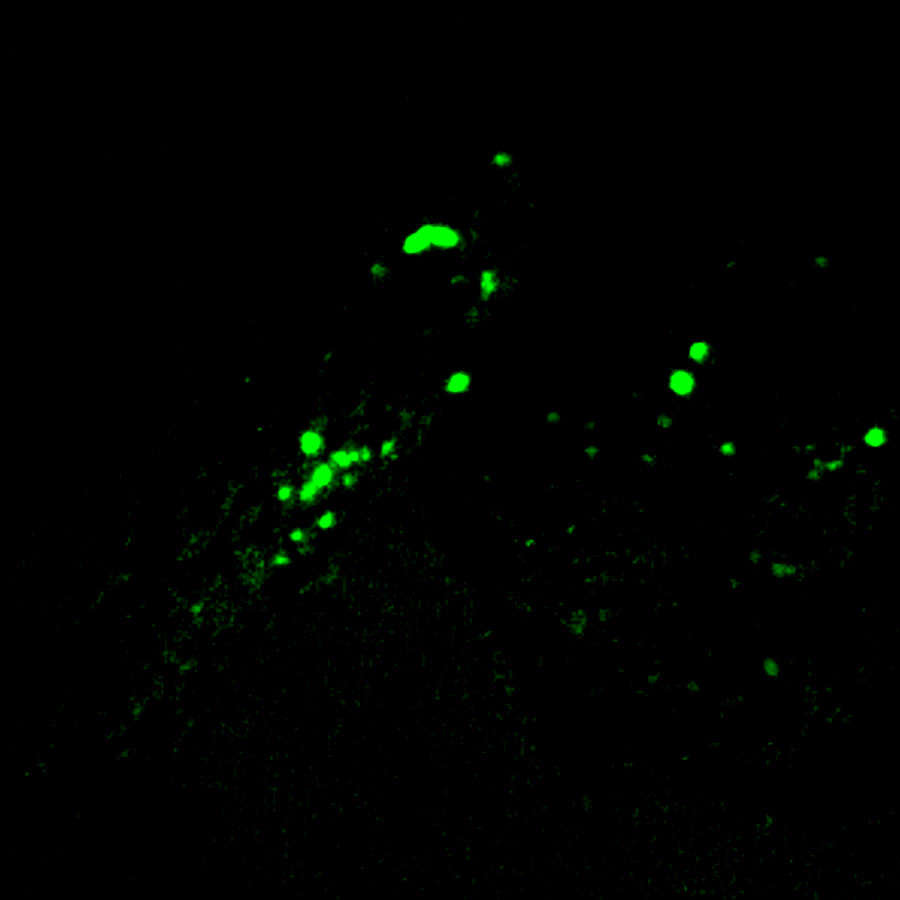

Supplement: Supplementary file 8 — Electron microscopy images for Fig. 2c. [file 42255_2025_1225_MOESM8_ESM.zip › Zuhra_Microscopy_Images_Fig2/Zuhra_Microscopy_Main_Fig2_a/Fig2a_HepG2_LysoTracker.tif]

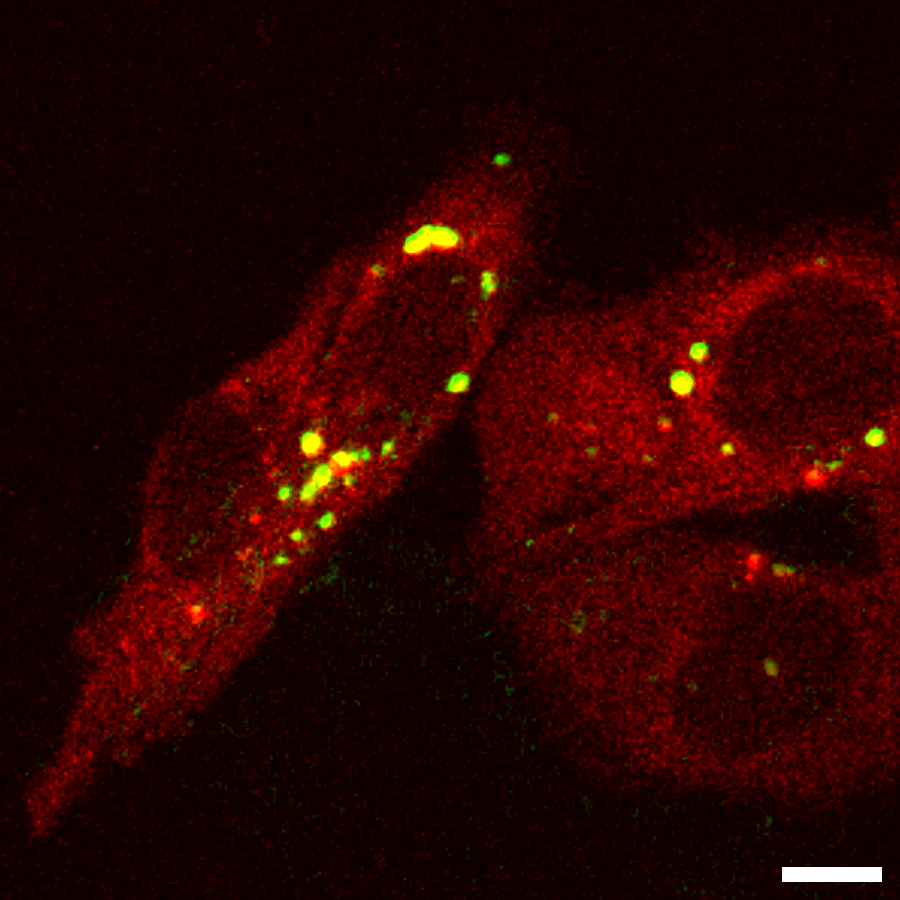

Supplement: Supplementary file 8 — Electron microscopy images for Fig. 2c. [file 42255_2025_1225_MOESM8_ESM.zip › Zuhra_Microscopy_Images_Fig2/Zuhra_Microscopy_Main_Fig2_a/Fig2a_HepG2_MERGE_LysoTracker-CP.tif]

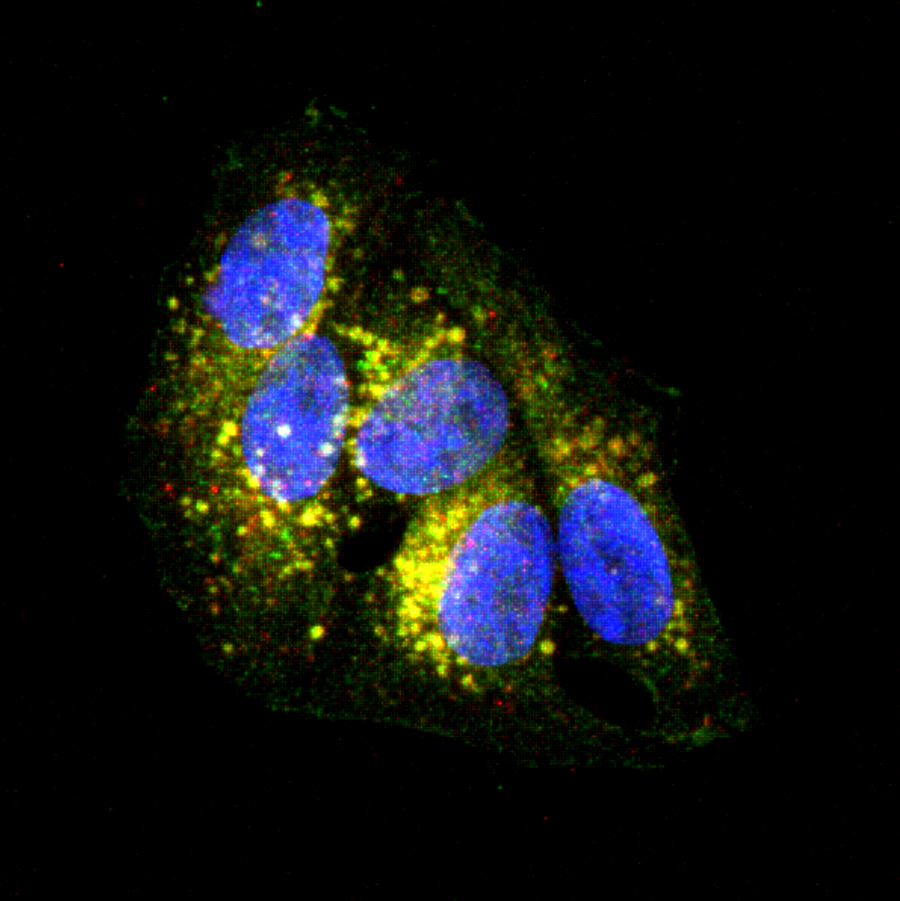

Supplement: Supplementary file 8 — Electron microscopy images for Fig. 2c. [file 42255_2025_1225_MOESM8_ESM.zip › Zuhra_Microscopy_Images_Fig2/Zuhra_Microscopy_Main_Fig2_h/Fig1hMPO-LAMP1-DAPI_merged.tif]

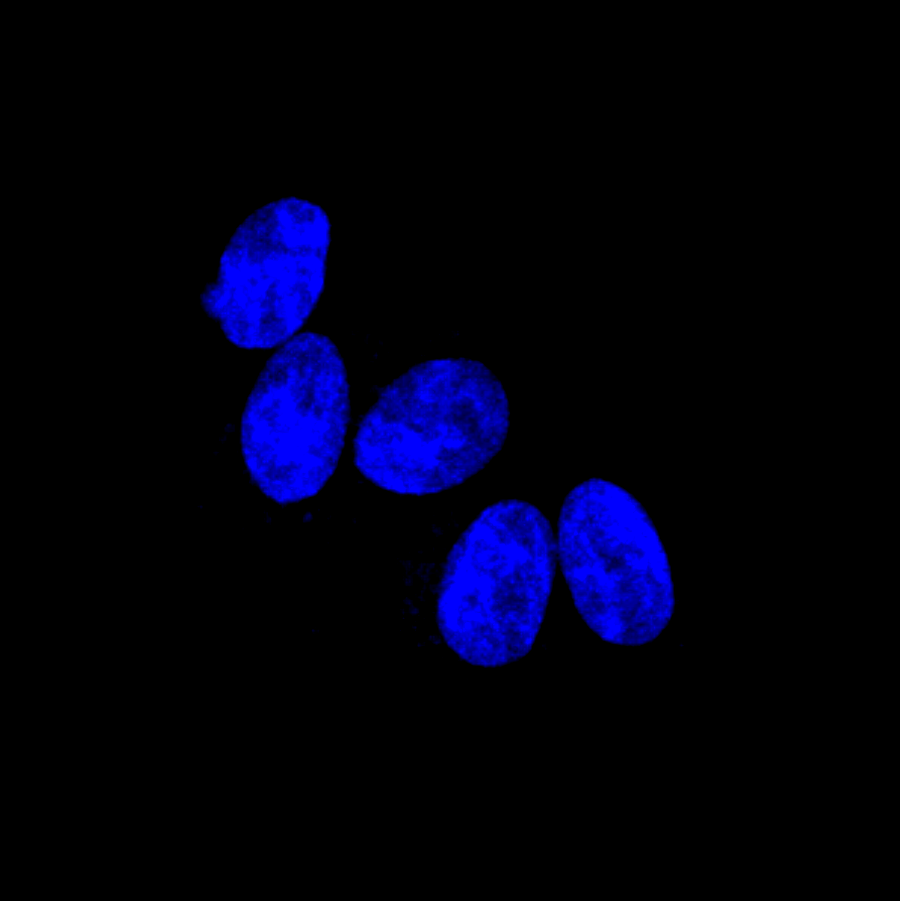

Supplement: Supplementary file 8 — Electron microscopy images for Fig. 2c. [file 42255_2025_1225_MOESM8_ESM.zip › Zuhra_Microscopy_Images_Fig2/Zuhra_Microscopy_Main_Fig2_h/Fig1h_Dapi.tif]

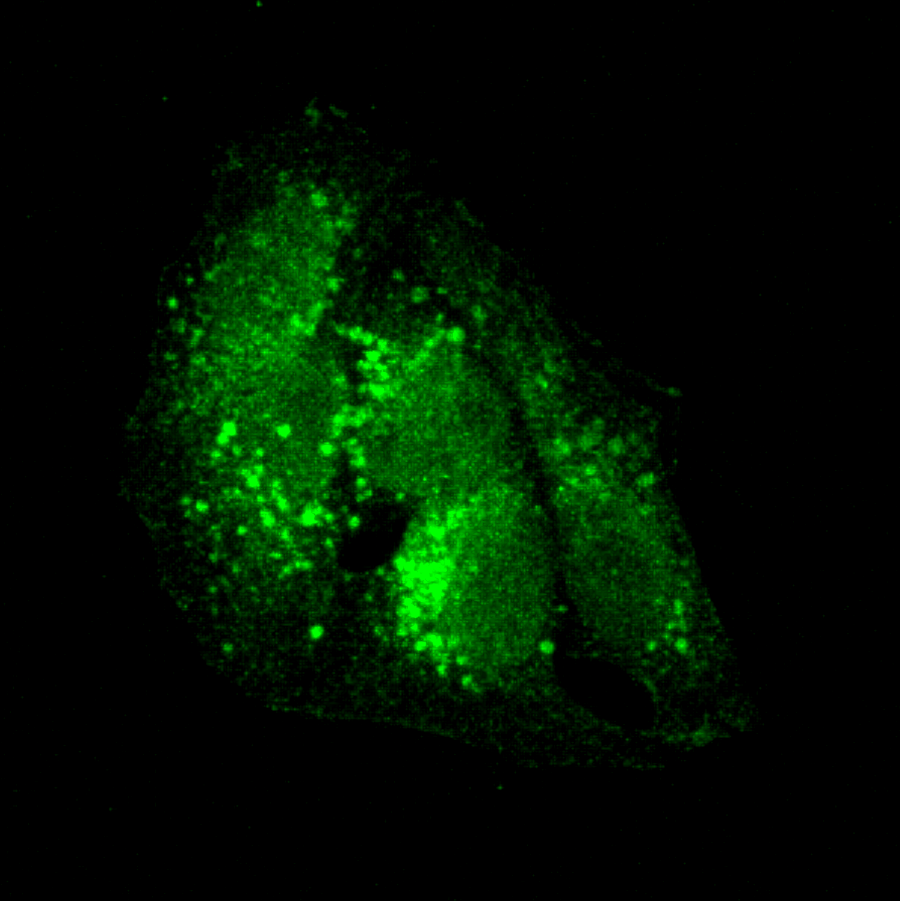

Supplement: Supplementary file 8 — Electron microscopy images for Fig. 2c. [file 42255_2025_1225_MOESM8_ESM.zip › Zuhra_Microscopy_Images_Fig2/Zuhra_Microscopy_Main_Fig2_h/Fig1h_LAMP1.tif]

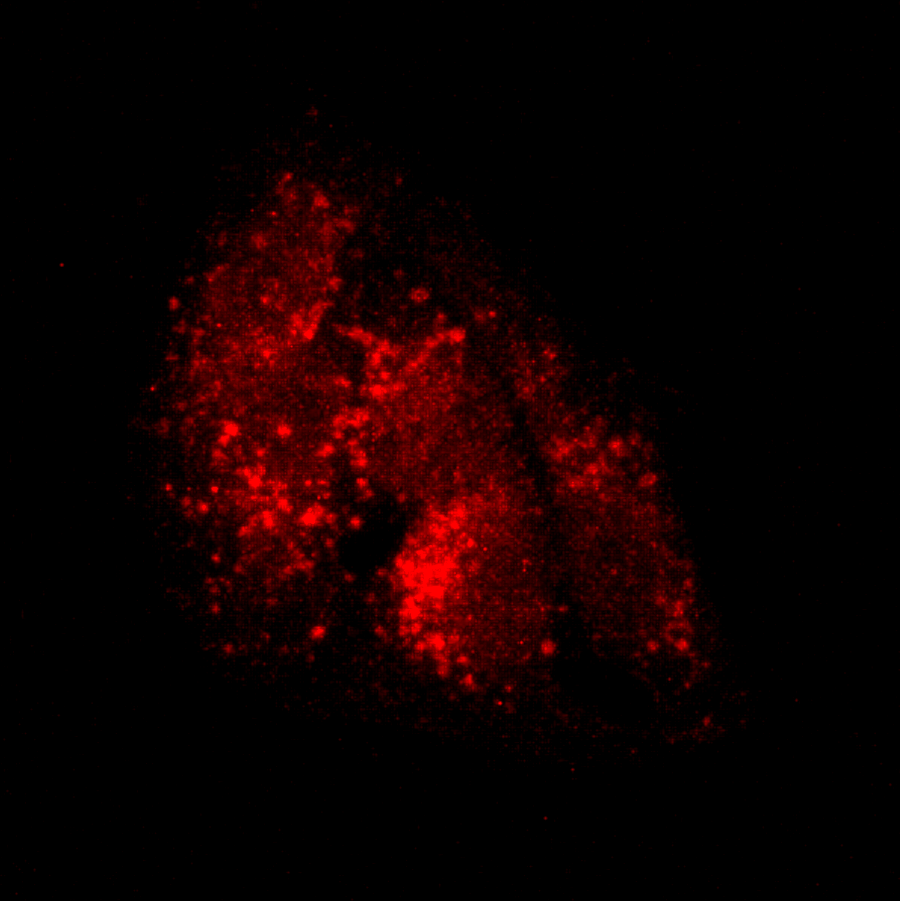

Supplement: Supplementary file 8 — Electron microscopy images for Fig. 2c. [file 42255_2025_1225_MOESM8_ESM.zip › Zuhra_Microscopy_Images_Fig2/Zuhra_Microscopy_Main_Fig2_h/Fig1h_MPO.tif]

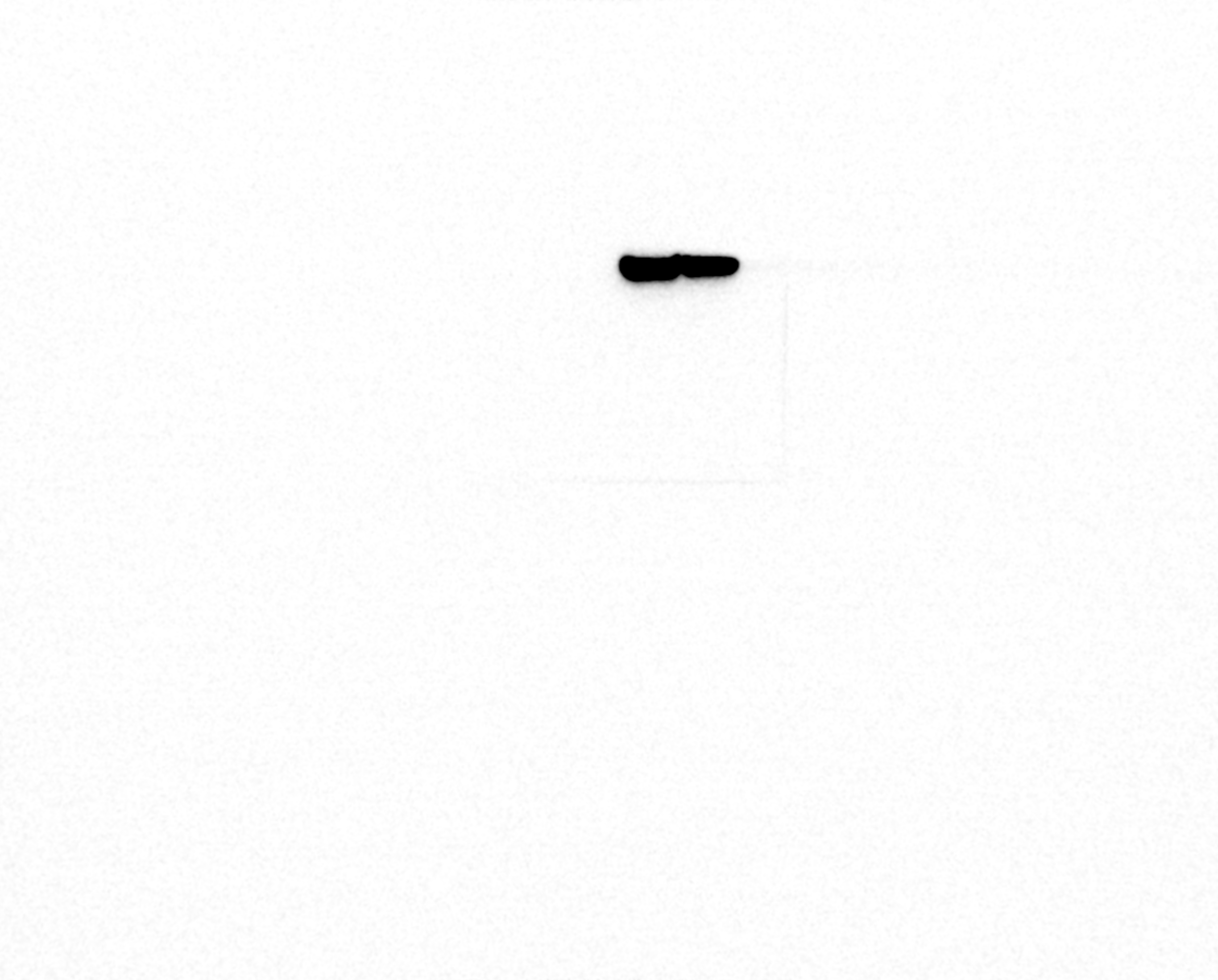

Supplement: Supplementary file 9 — Unprocessed western blots for Fig. 2g,n. [file 42255_2025_1225_MOESM9_ESM.zip › Zuhra_Unmodified_WesternBlot_Fig2/Zuhra_WesternBlot_Main_Fig2_g/MPO/Fig2g_MPO_Experiment1_actin.jpg]

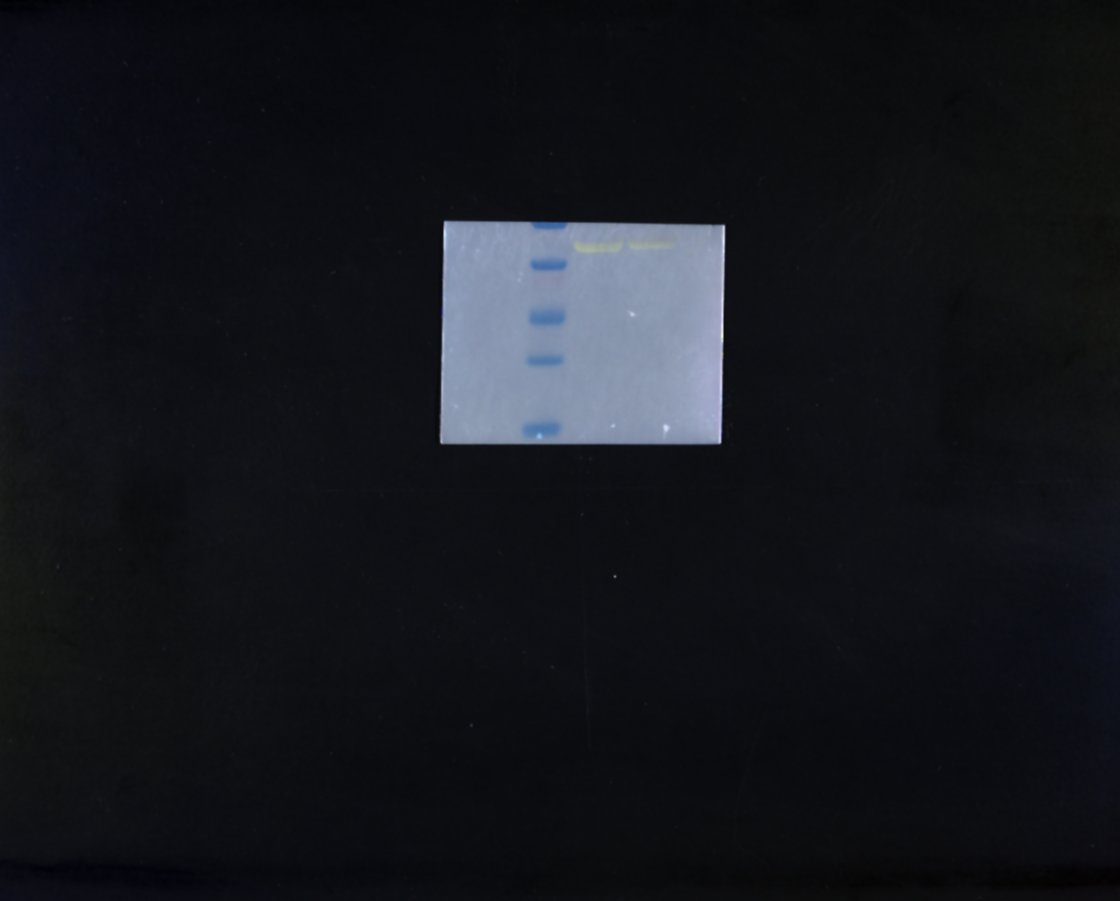

Supplement: Supplementary file 9 — Unprocessed western blots for Fig. 2g,n. [file 42255_2025_1225_MOESM9_ESM.zip › Zuhra_Unmodified_WesternBlot_Fig2/Zuhra_WesternBlot_Main_Fig2_g/MPO/Fig2g_MPO_Experiment1_actin_marker.jpg]

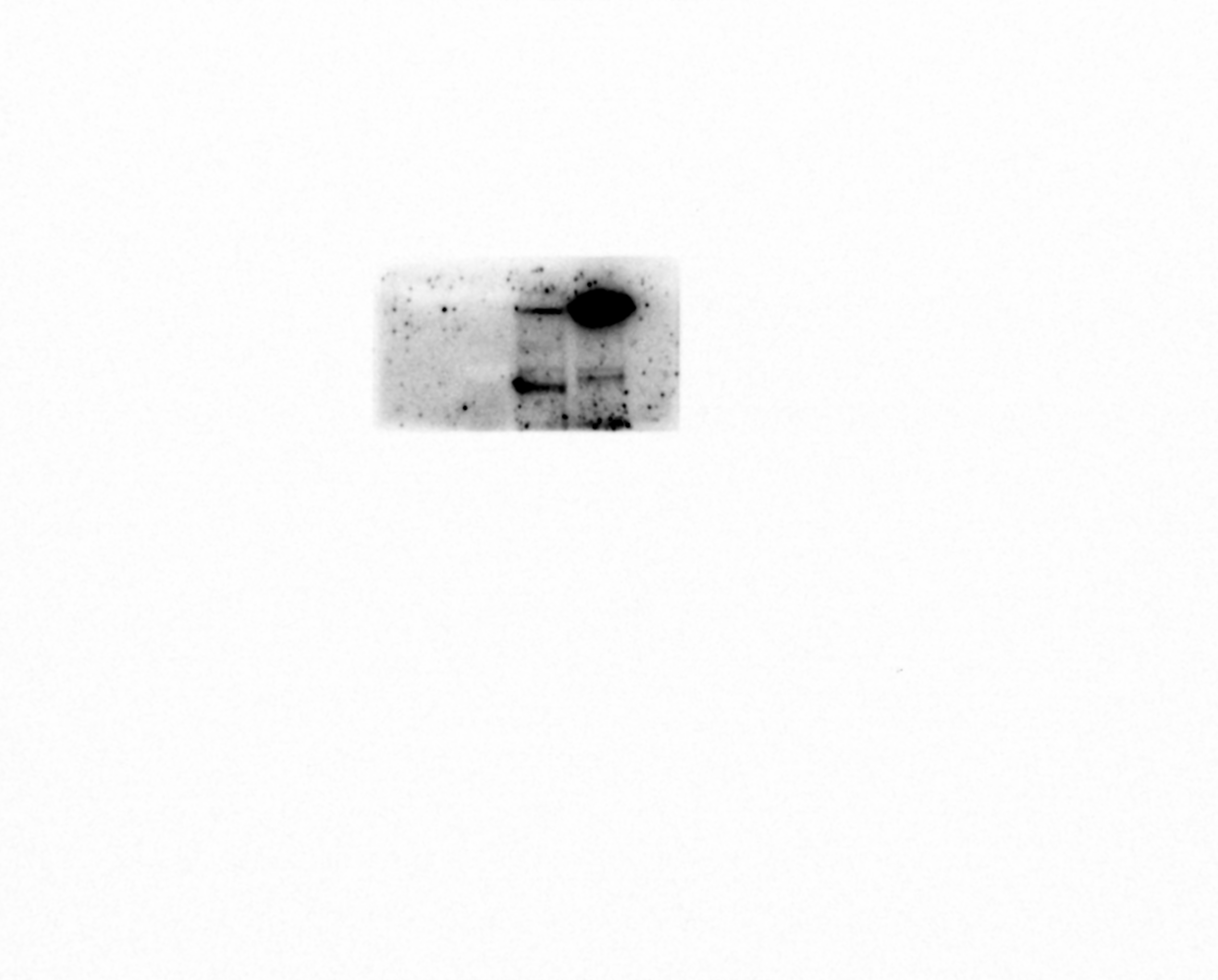

Supplement: Supplementary file 9 — Unprocessed western blots for Fig. 2g,n. [file 42255_2025_1225_MOESM9_ESM.zip › Zuhra_Unmodified_WesternBlot_Fig2/Zuhra_WesternBlot_Main_Fig2_g/MPO/Fig2g_MPO_Experiment1_MPO.jpg]

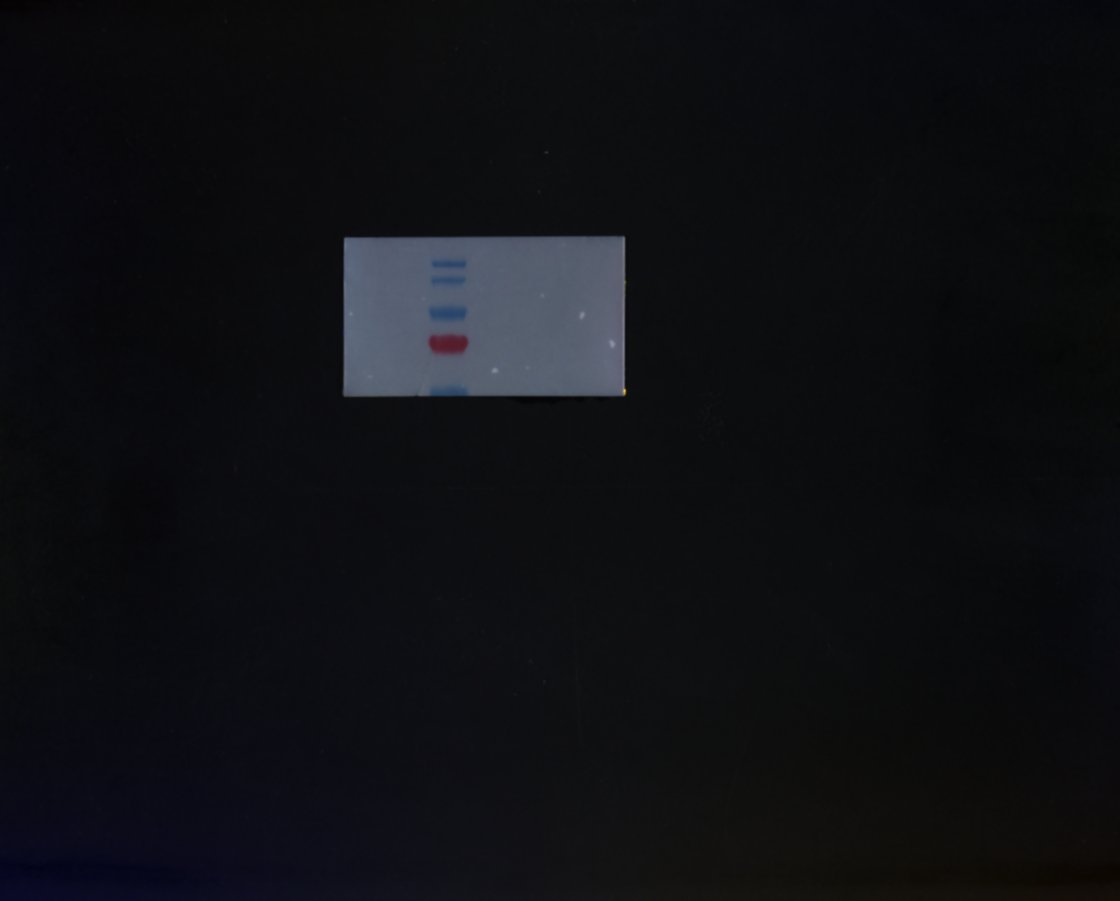

Supplement: Supplementary file 9 — Unprocessed western blots for Fig. 2g,n. [file 42255_2025_1225_MOESM9_ESM.zip › Zuhra_Unmodified_WesternBlot_Fig2/Zuhra_WesternBlot_Main_Fig2_g/MPO/Fig2g_MPO_Experiment1_MPO_marker.jpg]

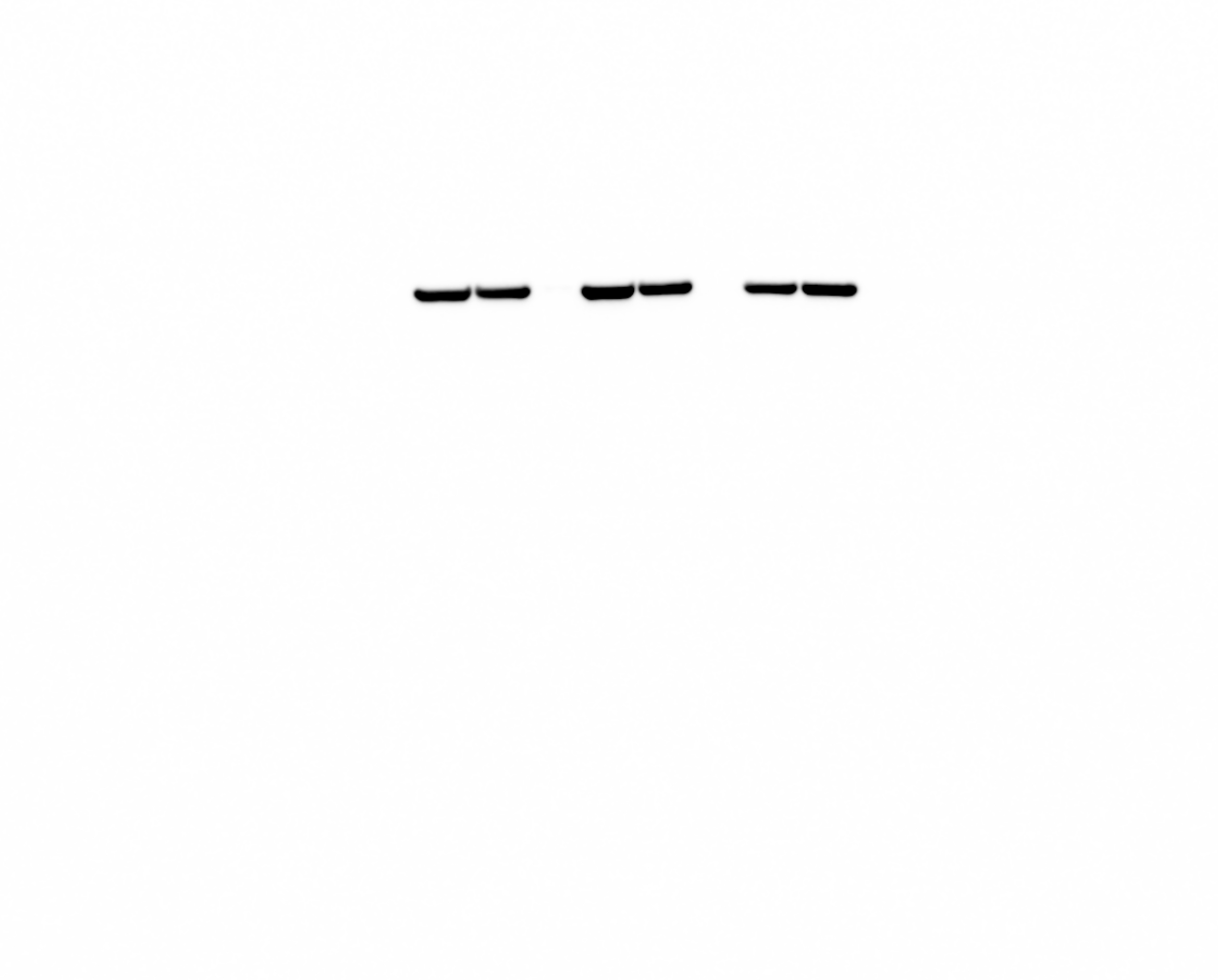

Supplement: Supplementary file 9 — Unprocessed western blots for Fig. 2g,n. [file 42255_2025_1225_MOESM9_ESM.zip › Zuhra_Unmodified_WesternBlot_Fig2/Zuhra_WesternBlot_Main_Fig2_g/MPO/Fig2g_MPO_Experiment2-3-4_actin.jpg]

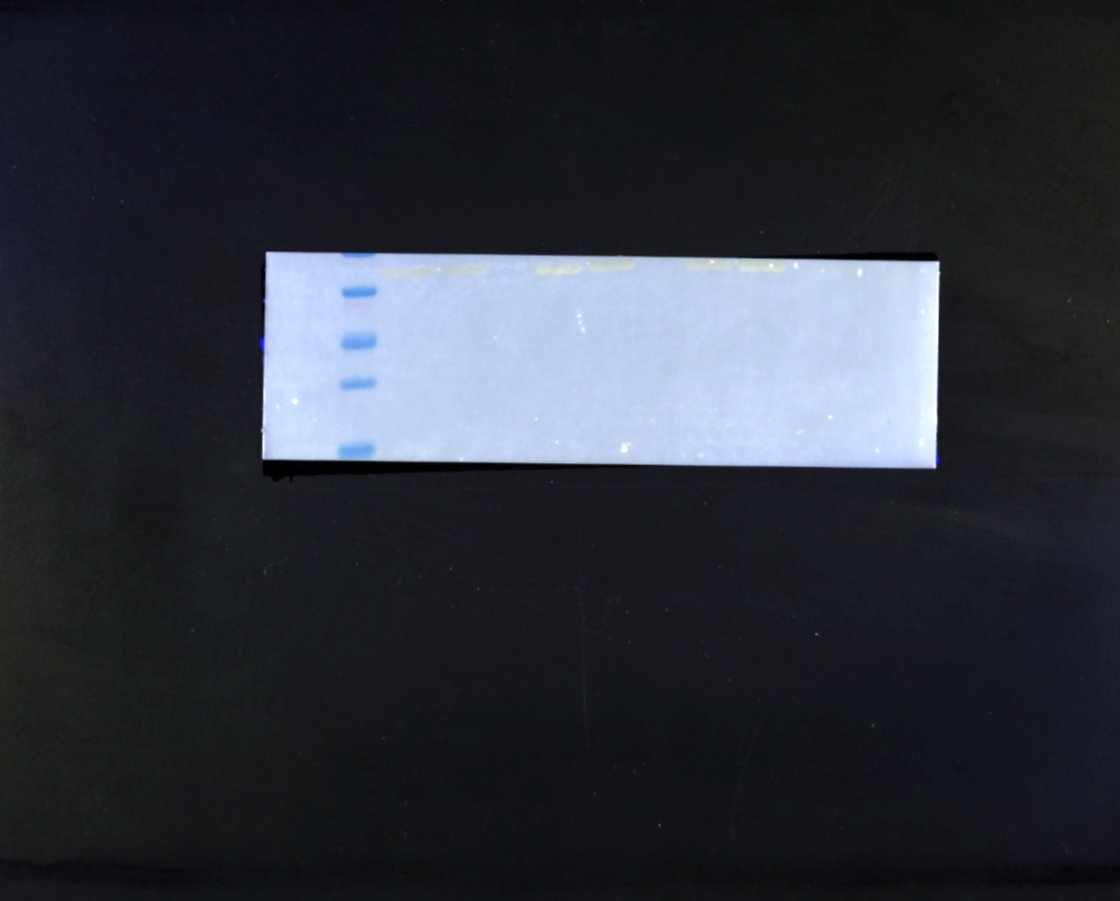

Supplement: Supplementary file 9 — Unprocessed western blots for Fig. 2g,n. [file 42255_2025_1225_MOESM9_ESM.zip › Zuhra_Unmodified_WesternBlot_Fig2/Zuhra_WesternBlot_Main_Fig2_g/MPO/Fig2g_MPO_Experiment2-3-4_actin_marker.jpg]

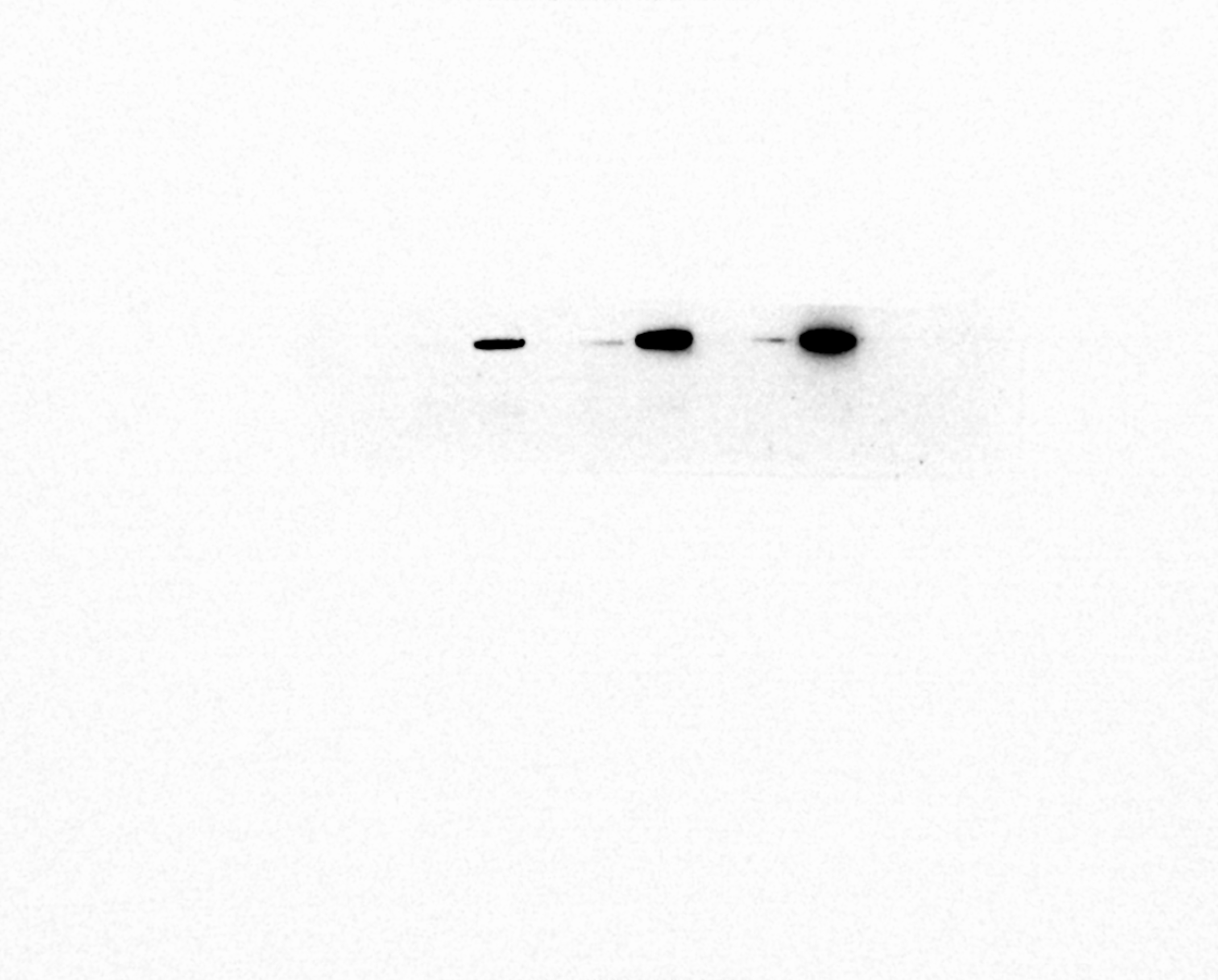

Supplement: Supplementary file 9 — Unprocessed western blots for Fig. 2g,n. [file 42255_2025_1225_MOESM9_ESM.zip › Zuhra_Unmodified_WesternBlot_Fig2/Zuhra_WesternBlot_Main_Fig2_g/MPO/Fig2g_MPO_Experiment2-3-4_MPO.jpg]

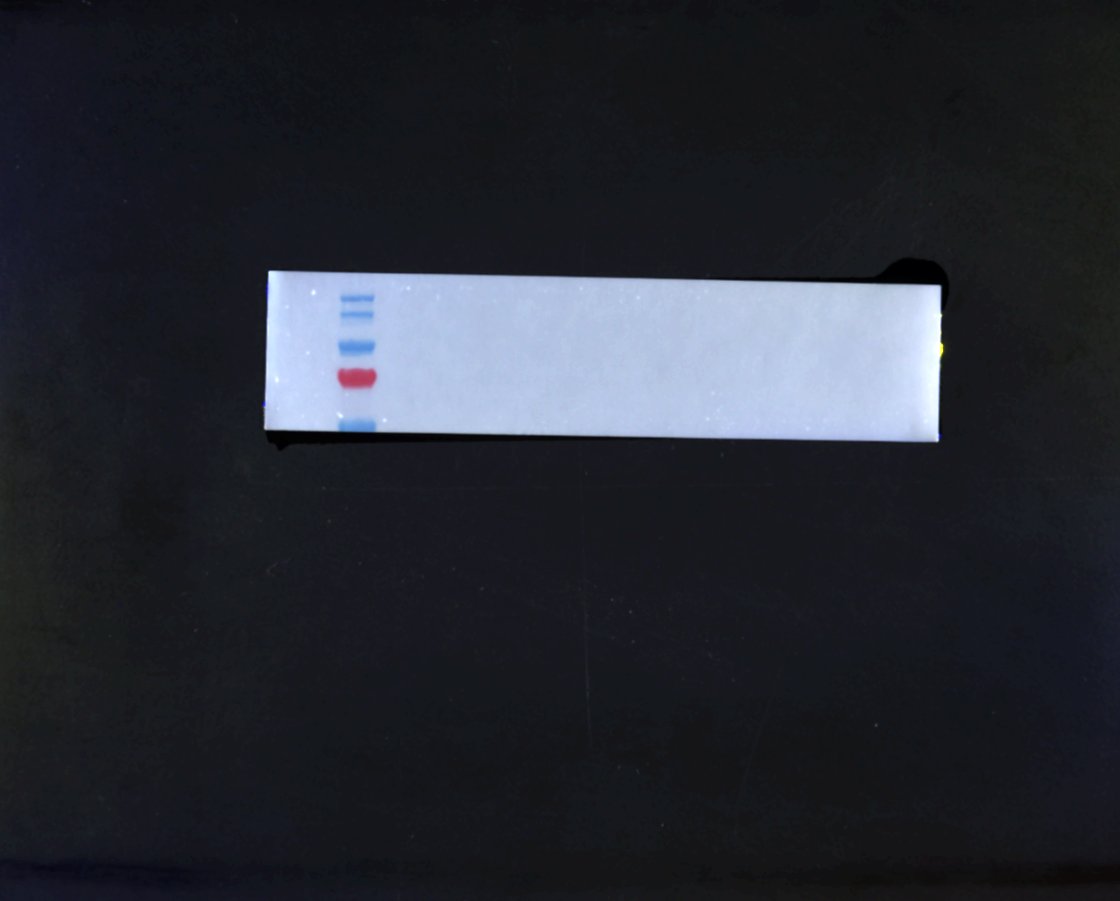

Supplement: Supplementary file 9 — Unprocessed western blots for Fig. 2g,n. [file 42255_2025_1225_MOESM9_ESM.zip › Zuhra_Unmodified_WesternBlot_Fig2/Zuhra_WesternBlot_Main_Fig2_g/MPO/Fig2g_MPO_Experiment2-3-4_MPO_marker.jpg]

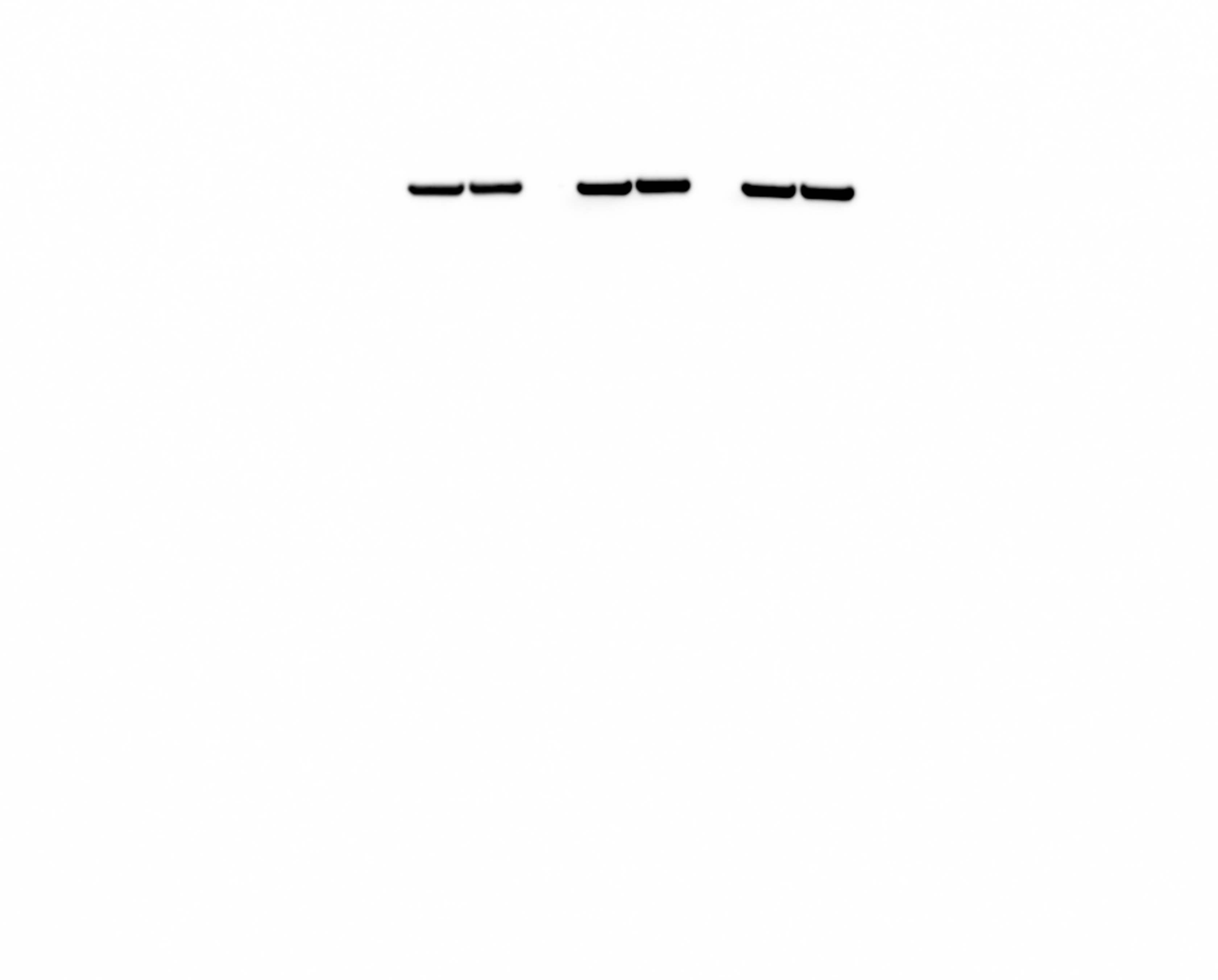

Supplement: Supplementary file 9 — Unprocessed western blots for Fig. 2g,n. [file 42255_2025_1225_MOESM9_ESM.zip › Zuhra_Unmodified_WesternBlot_Fig2/Zuhra_WesternBlot_Main_Fig2_g/MPO/Fig2g_MPO_Experiment5-6-7_actin.jpg]

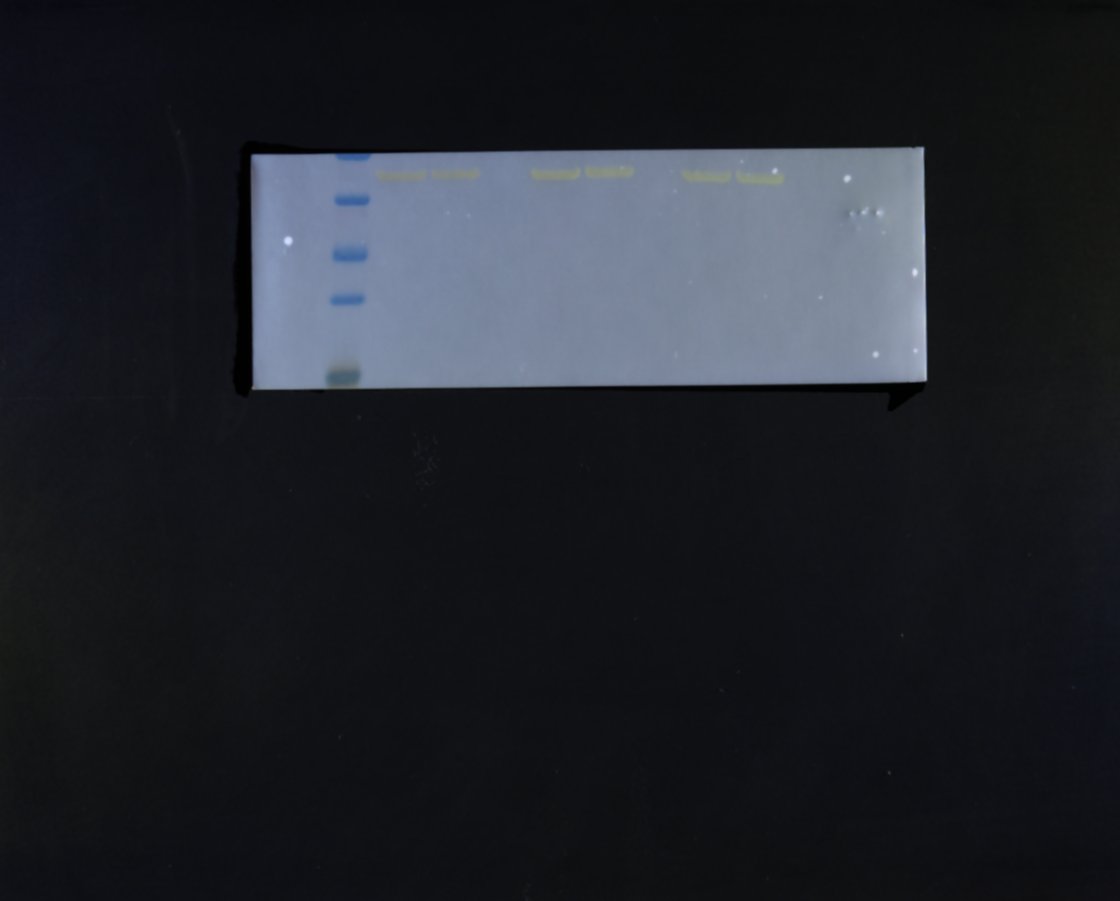

Supplement: Supplementary file 9 — Unprocessed western blots for Fig. 2g,n. [file 42255_2025_1225_MOESM9_ESM.zip › Zuhra_Unmodified_WesternBlot_Fig2/Zuhra_WesternBlot_Main_Fig2_g/MPO/Fig2g_MPO_Experiment5-6-7_actin_marker.jpg]

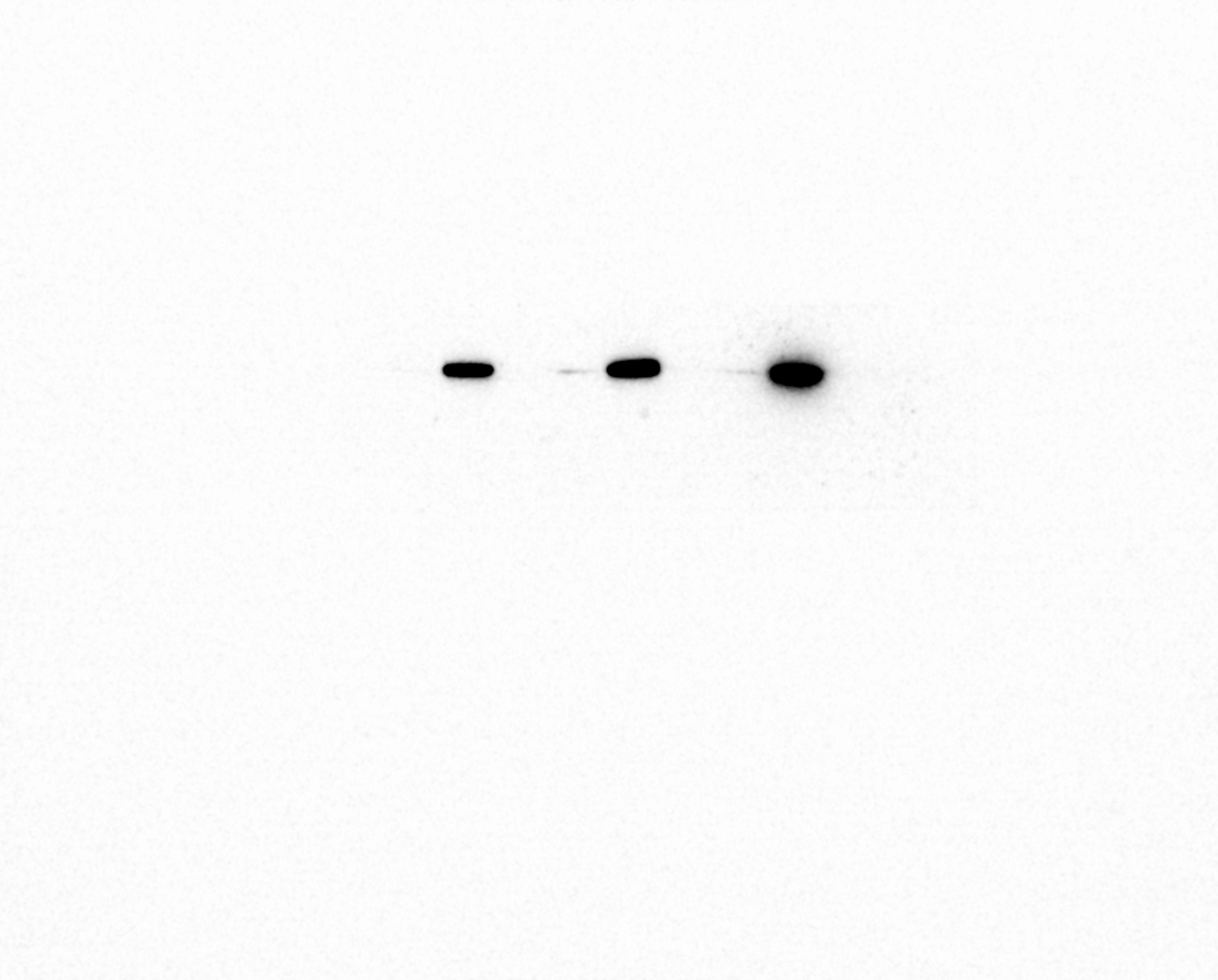

Supplement: Supplementary file 9 — Unprocessed western blots for Fig. 2g,n. [file 42255_2025_1225_MOESM9_ESM.zip › Zuhra_Unmodified_WesternBlot_Fig2/Zuhra_WesternBlot_Main_Fig2_g/MPO/Fig2g_MPO_Experiment5-6-7_MPO.jpg]

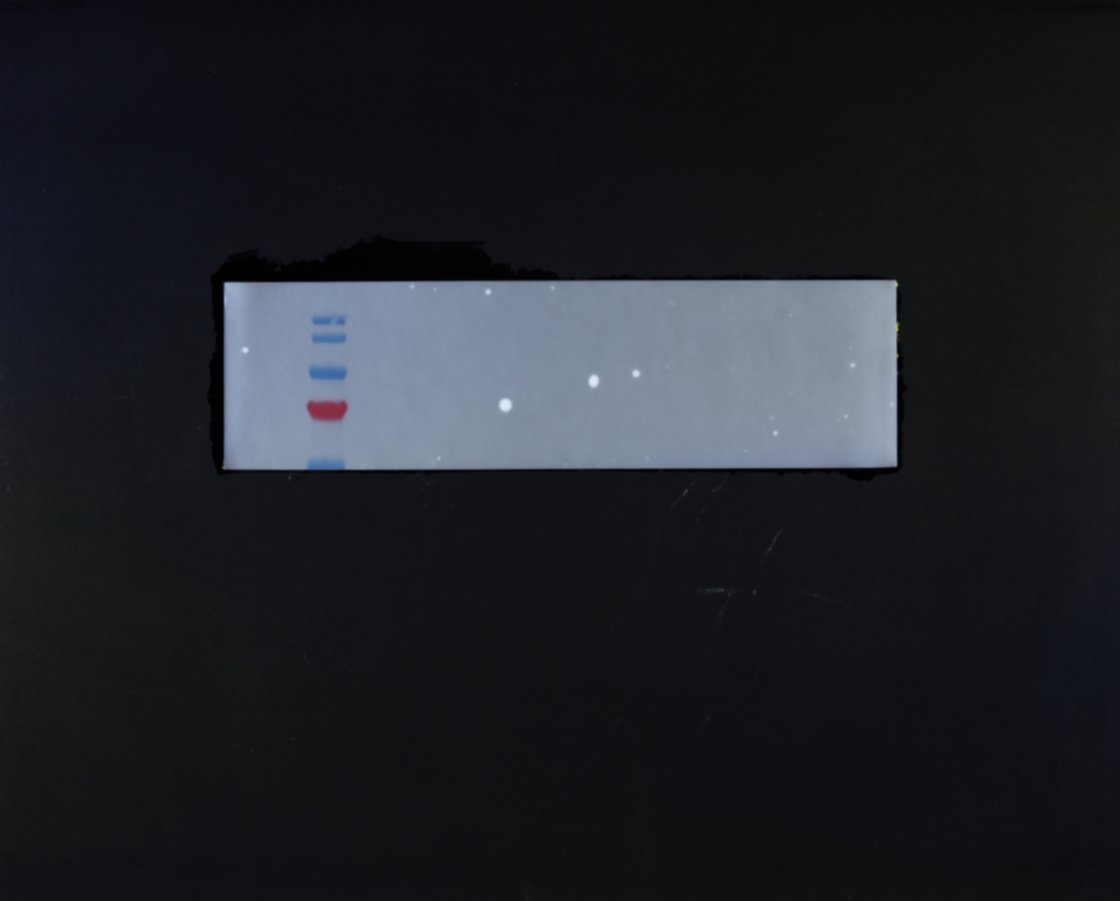

Supplement: Supplementary file 9 — Unprocessed western blots for Fig. 2g,n. [file 42255_2025_1225_MOESM9_ESM.zip › Zuhra_Unmodified_WesternBlot_Fig2/Zuhra_WesternBlot_Main_Fig2_g/MPO/Fig2g_MPO_Experiment5-6-7_MPO_marker.jpg]

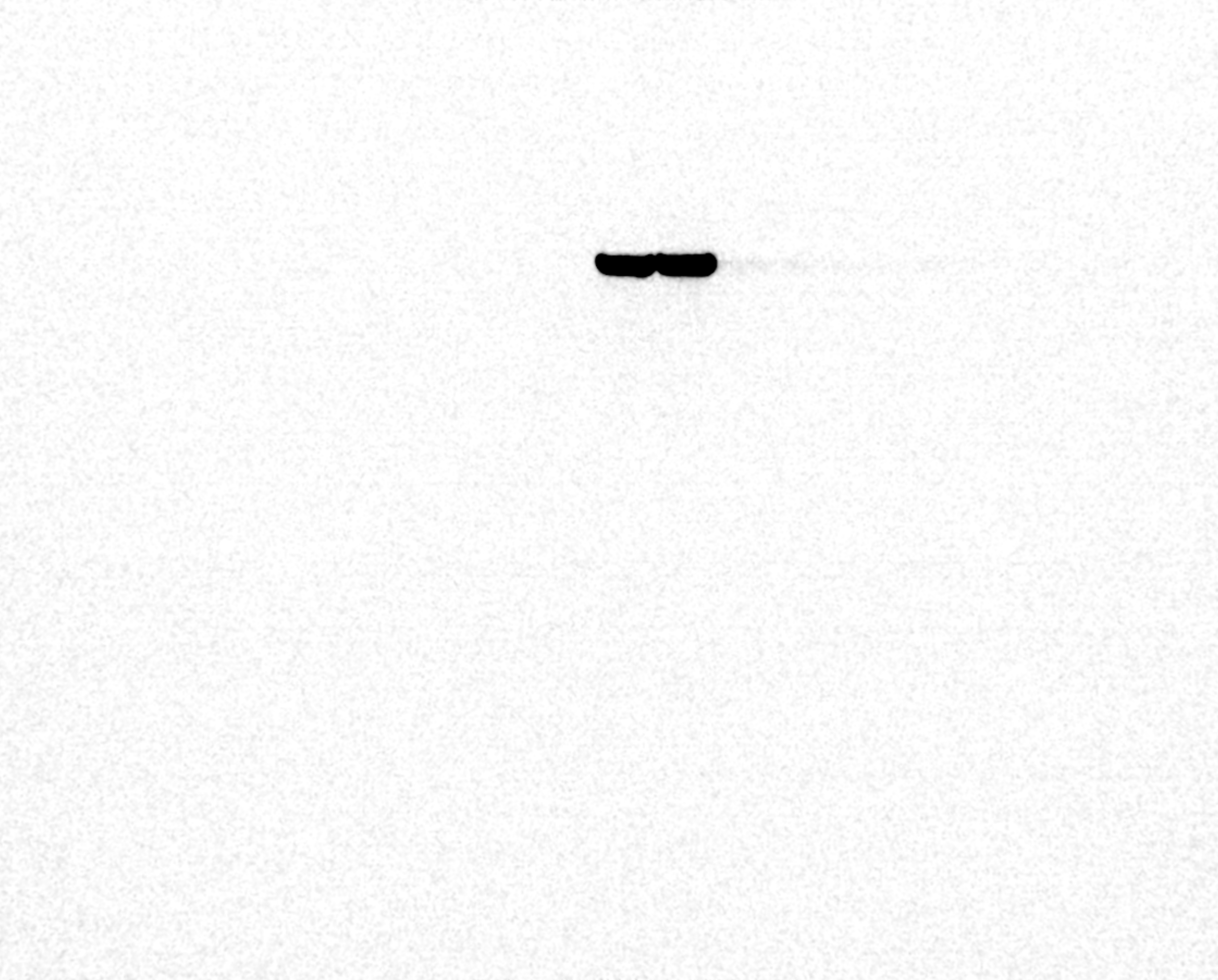

Supplement: Supplementary file 9 — Unprocessed western blots for Fig. 2g,n. [file 42255_2025_1225_MOESM9_ESM.zip › Zuhra_Unmodified_WesternBlot_Fig2/Zuhra_WesternBlot_Main_Fig2_g/PXDN/Fig2g_PXDN_Experiment1_actin.jpg]

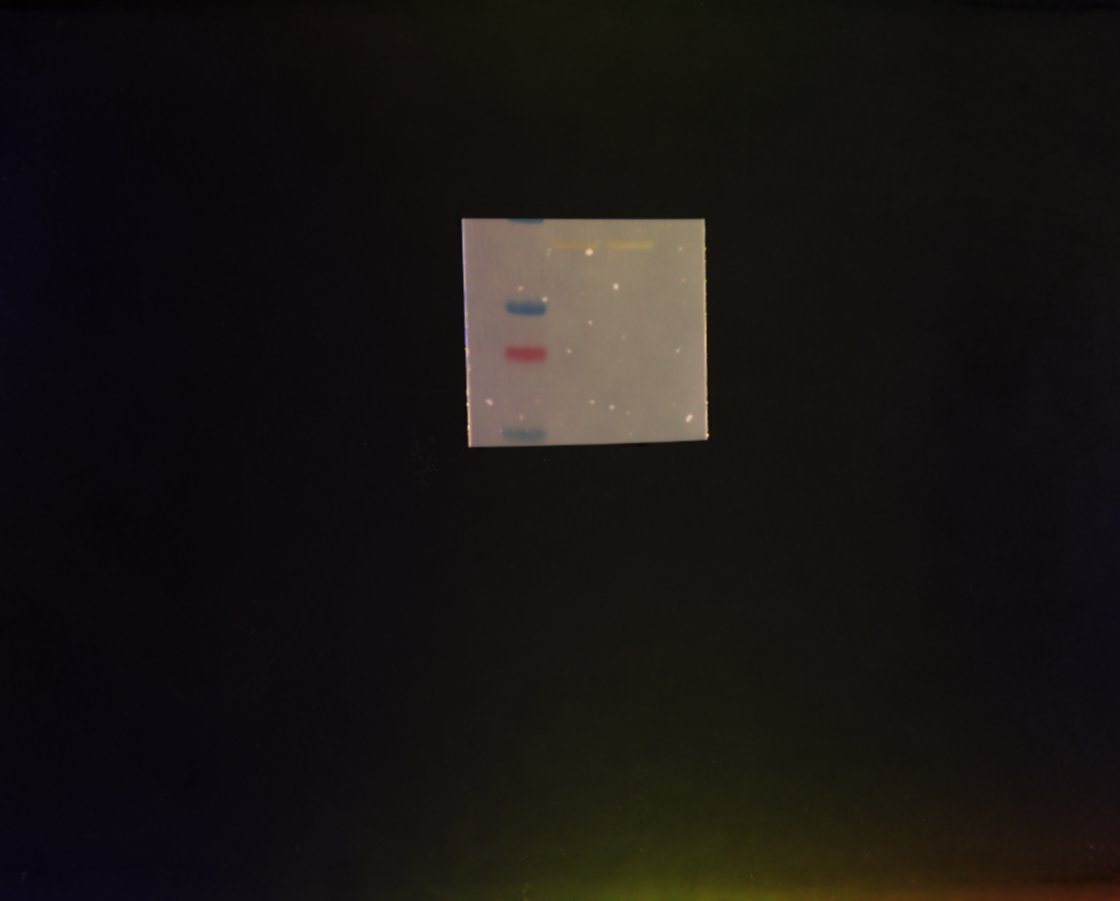

Supplement: Supplementary file 9 — Unprocessed western blots for Fig. 2g,n. [file 42255_2025_1225_MOESM9_ESM.zip › Zuhra_Unmodified_WesternBlot_Fig2/Zuhra_WesternBlot_Main_Fig2_g/PXDN/Fig2g_PXDN_Experiment1_actin_marker.jpg]

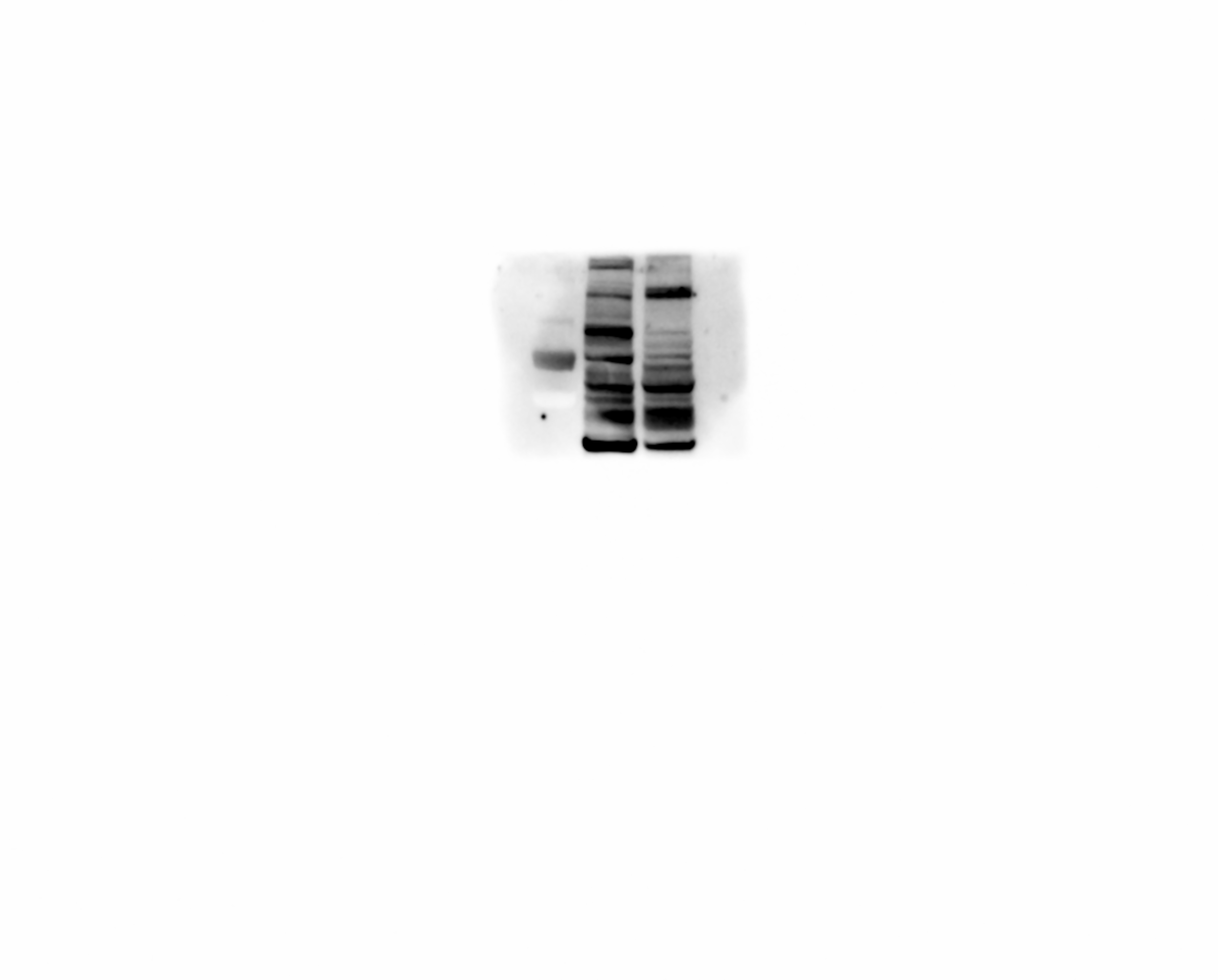

Supplement: Supplementary file 9 — Unprocessed western blots for Fig. 2g,n. [file 42255_2025_1225_MOESM9_ESM.zip › Zuhra_Unmodified_WesternBlot_Fig2/Zuhra_WesternBlot_Main_Fig2_g/PXDN/Fig2g_PXDN_Experiment1_PXDN.jpg]

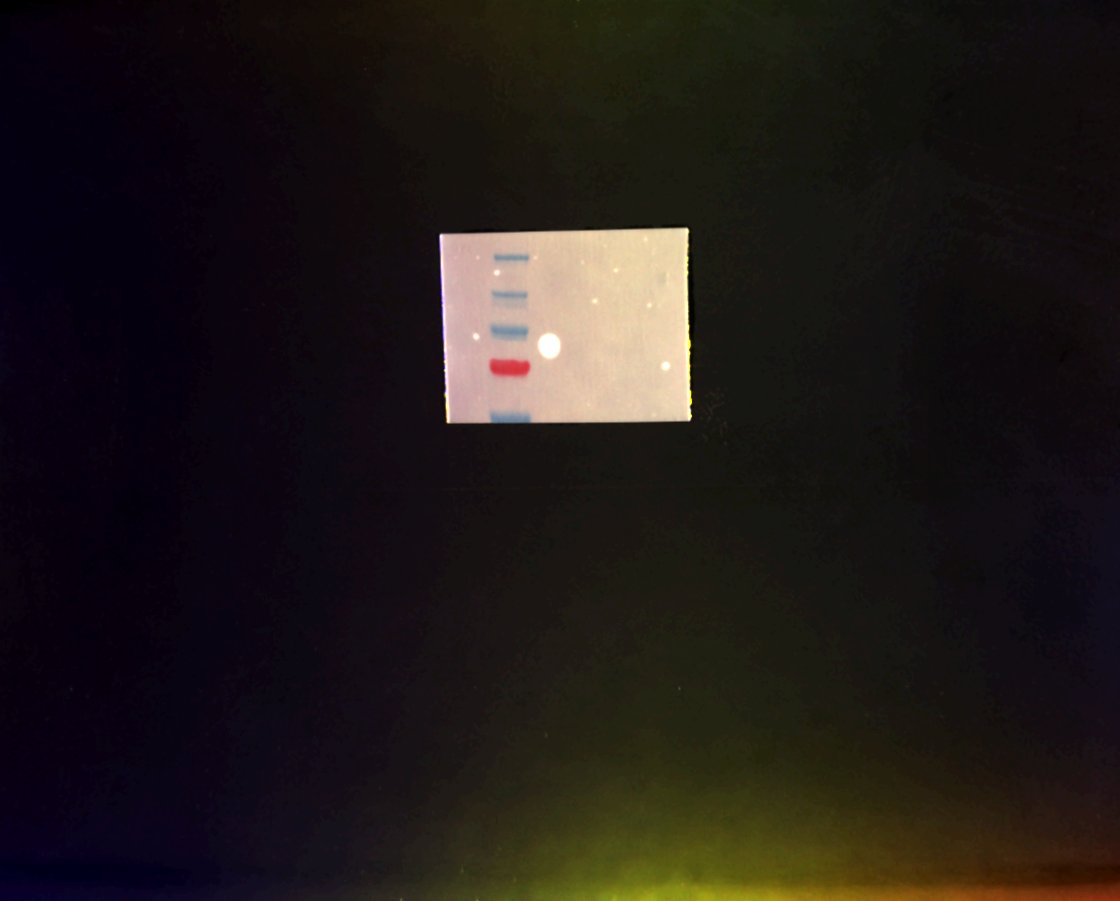

Supplement: Supplementary file 9 — Unprocessed western blots for Fig. 2g,n. [file 42255_2025_1225_MOESM9_ESM.zip › Zuhra_Unmodified_WesternBlot_Fig2/Zuhra_WesternBlot_Main_Fig2_g/PXDN/Fig2g_PXDN_Experiment1_PXDN_marker.jpg]

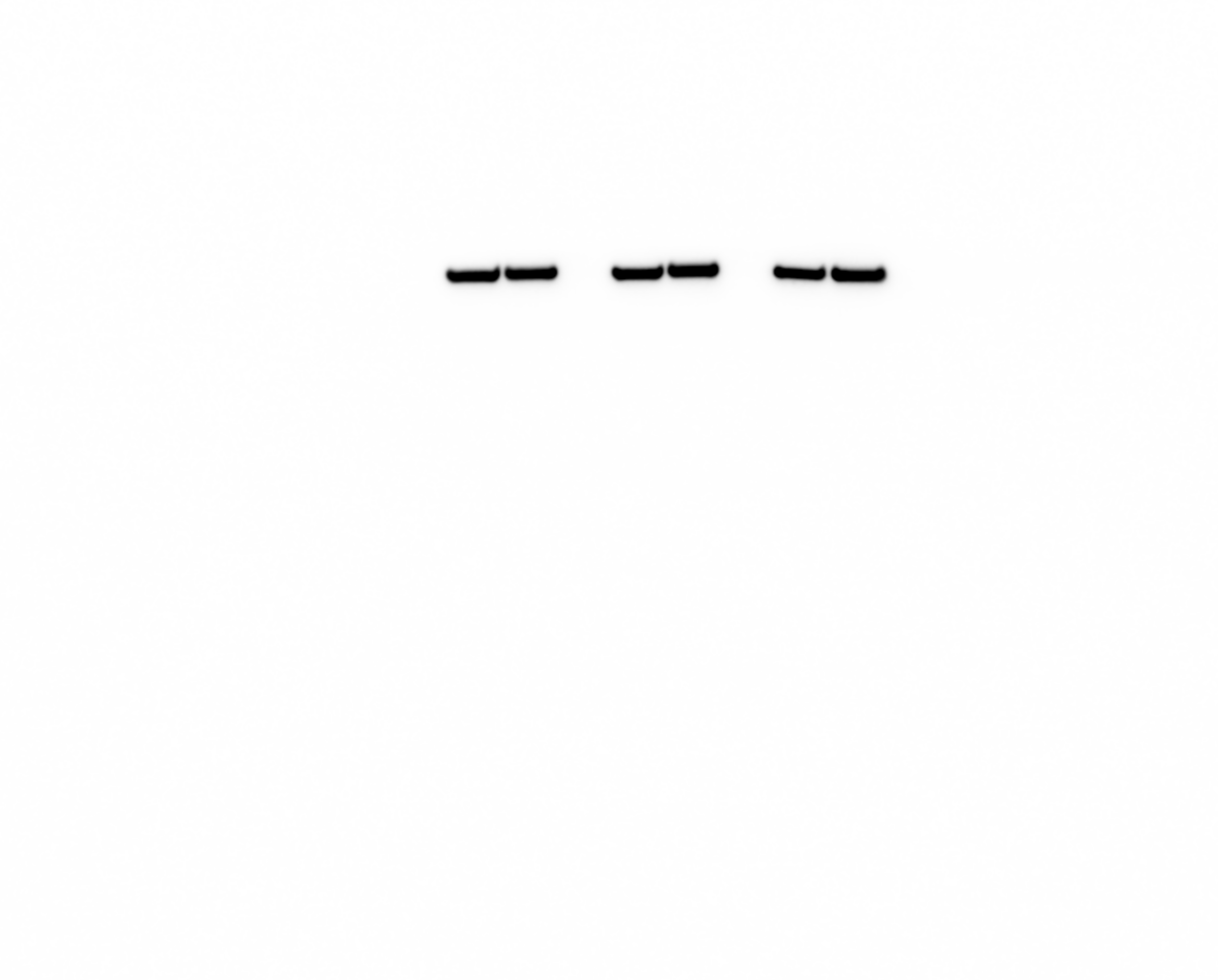

Supplement: Supplementary file 9 — Unprocessed western blots for Fig. 2g,n. [file 42255_2025_1225_MOESM9_ESM.zip › Zuhra_Unmodified_WesternBlot_Fig2/Zuhra_WesternBlot_Main_Fig2_g/PXDN/Fig2g_PXDN_Experiment2-3-4_actin.jpg]

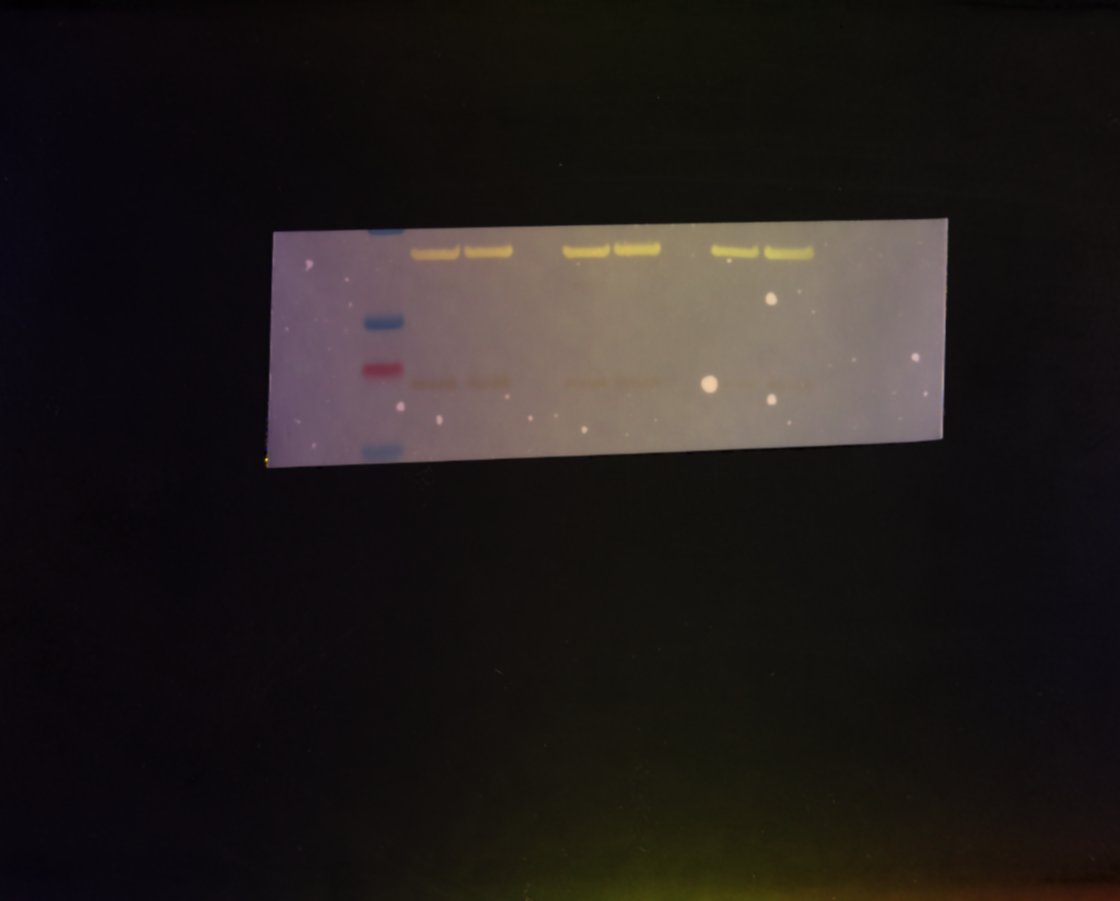

Supplement: Supplementary file 9 — Unprocessed western blots for Fig. 2g,n. [file 42255_2025_1225_MOESM9_ESM.zip › Zuhra_Unmodified_WesternBlot_Fig2/Zuhra_WesternBlot_Main_Fig2_g/PXDN/Fig2g_PXDN_Experiment2-3-4_actin_marker.jpg]

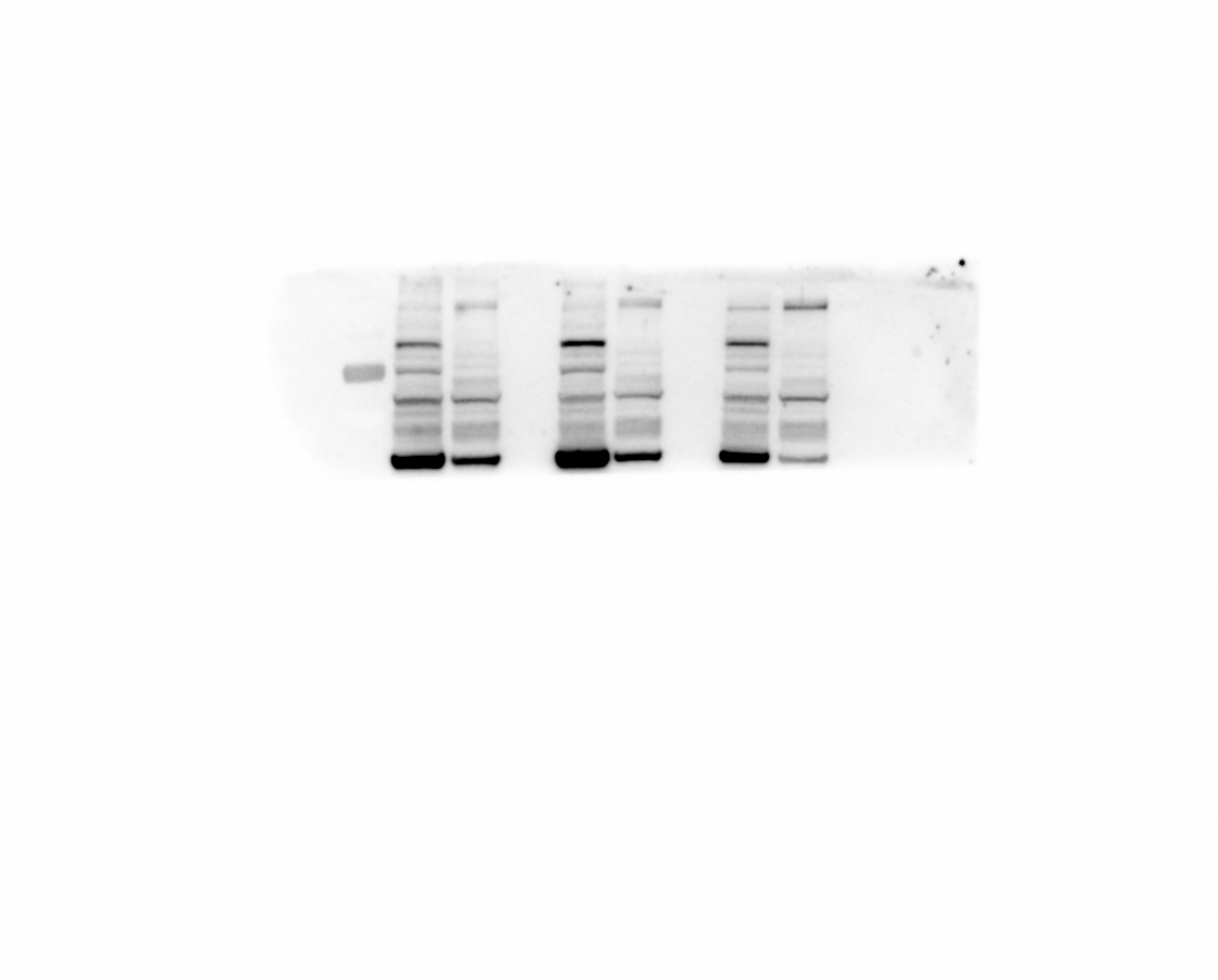

Supplement: Supplementary file 9 — Unprocessed western blots for Fig. 2g,n. [file 42255_2025_1225_MOESM9_ESM.zip › Zuhra_Unmodified_WesternBlot_Fig2/Zuhra_WesternBlot_Main_Fig2_g/PXDN/Fig2g_PXDN_Experiment2-3-4_PXDN.jpg]

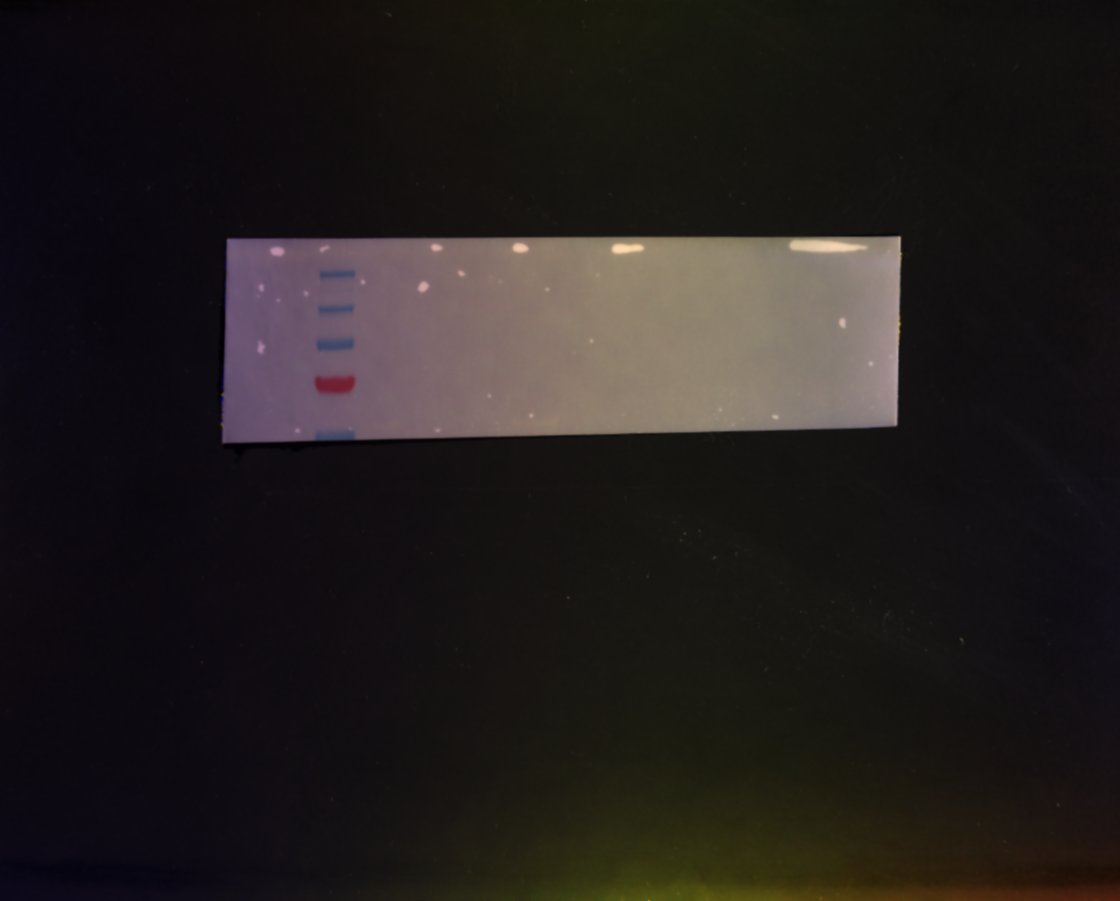

Supplement: Supplementary file 9 — Unprocessed western blots for Fig. 2g,n. [file 42255_2025_1225_MOESM9_ESM.zip › Zuhra_Unmodified_WesternBlot_Fig2/Zuhra_WesternBlot_Main_Fig2_g/PXDN/Fig2g_PXDN_Experiment2-3-4_PXDN_marker.jpg]

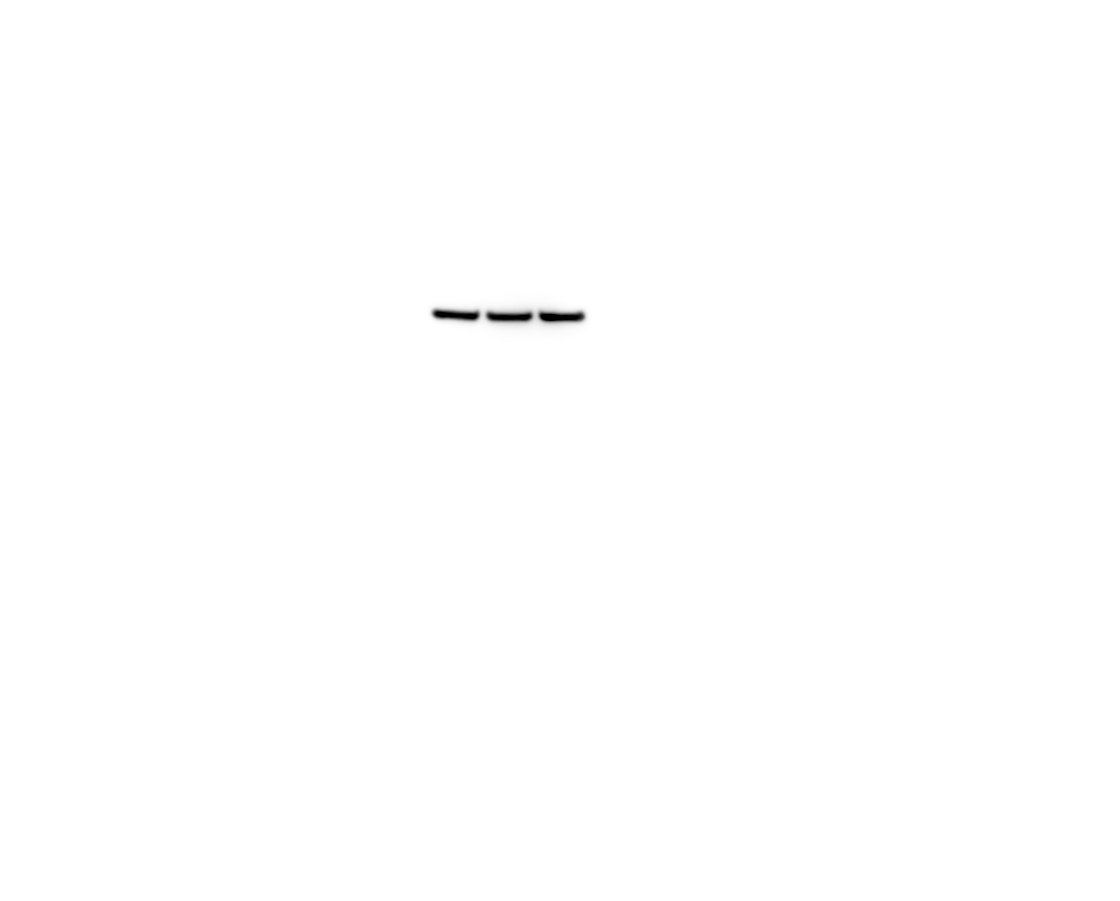

Supplement: Supplementary file 9 — Unprocessed western blots for Fig. 2g,n. [file 42255_2025_1225_MOESM9_ESM.zip › Zuhra_Unmodified_WesternBlot_Fig2/Zuhra_WesternBlot_Mani_Fig2_n/OE-TST/Fig2n_OE-TST_actin.jpg]

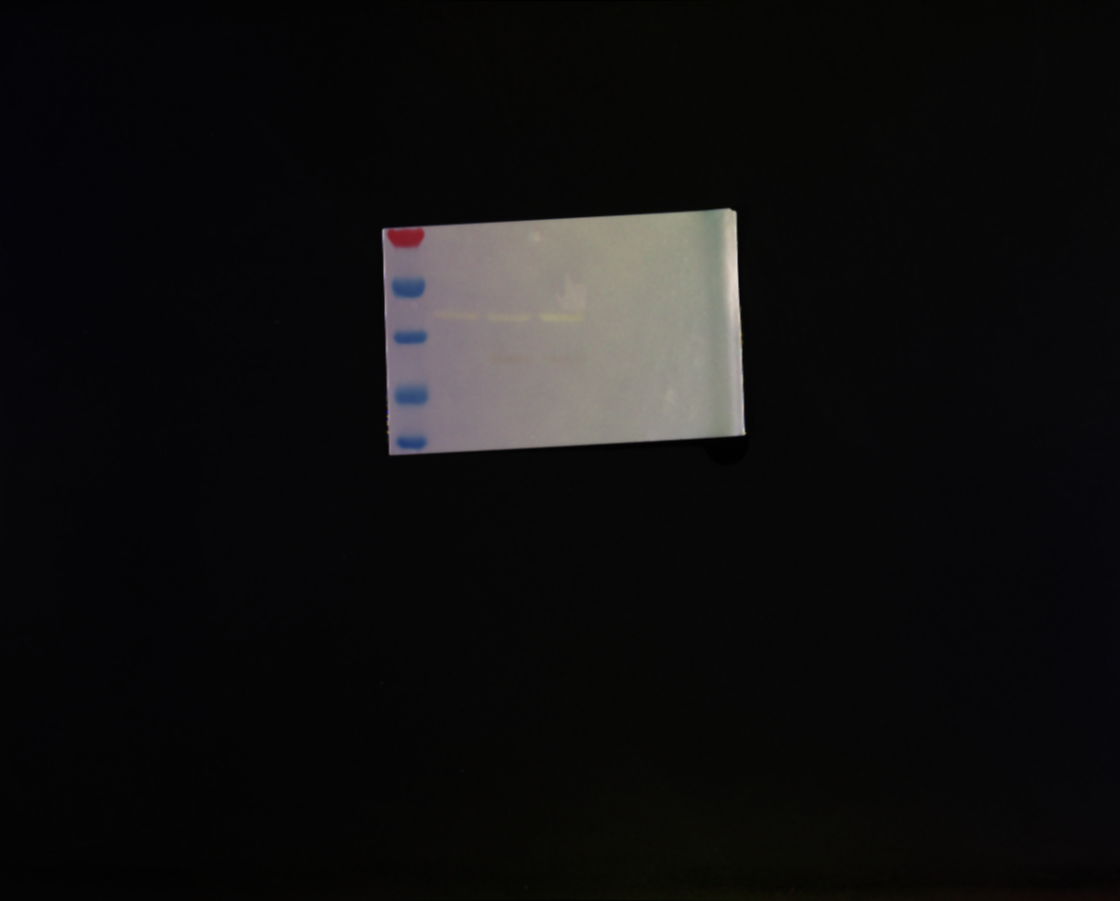

Supplement: Supplementary file 9 — Unprocessed western blots for Fig. 2g,n. [file 42255_2025_1225_MOESM9_ESM.zip › Zuhra_Unmodified_WesternBlot_Fig2/Zuhra_WesternBlot_Mani_Fig2_n/OE-TST/Fig2n_OE-TST_actin_marker.tif]

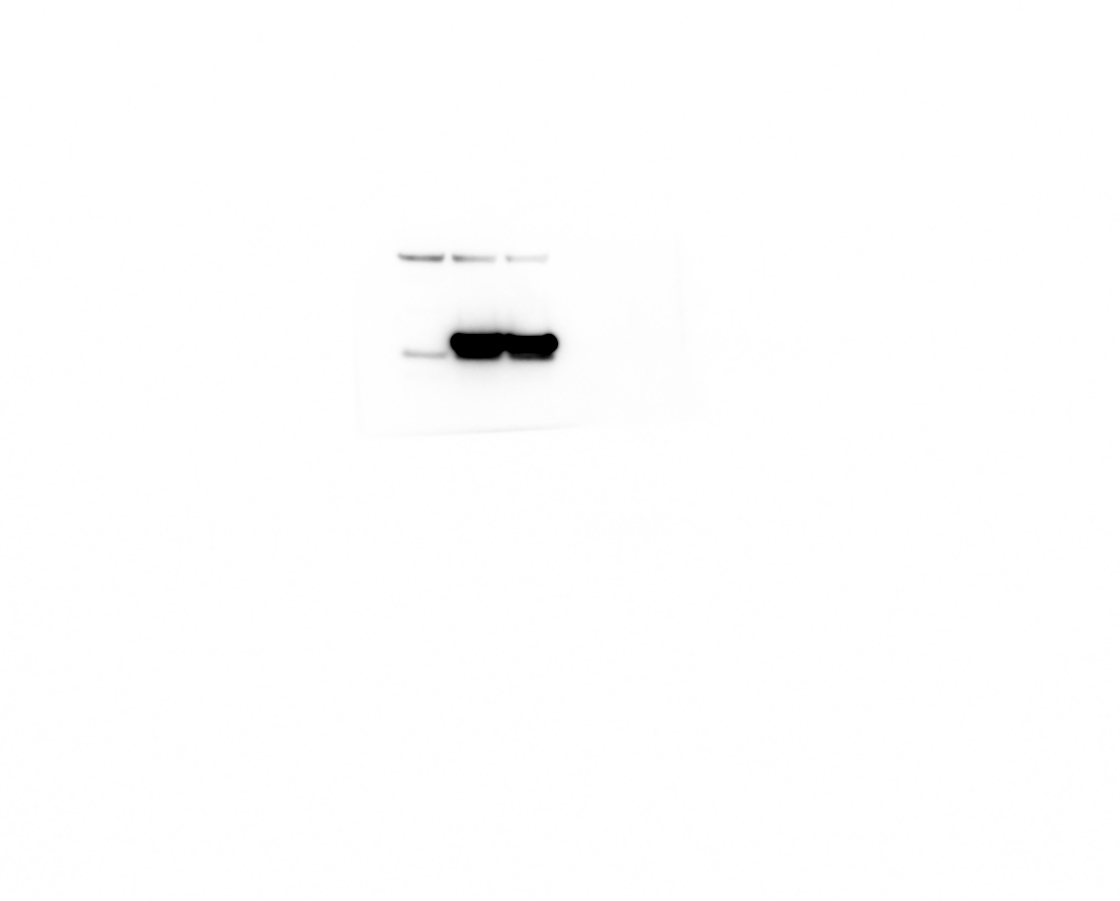

Supplement: Supplementary file 9 — Unprocessed western blots for Fig. 2g,n. [file 42255_2025_1225_MOESM9_ESM.zip › Zuhra_Unmodified_WesternBlot_Fig2/Zuhra_WesternBlot_Mani_Fig2_n/OE-TST/Fig2n_OE-TST_TST.jpg]

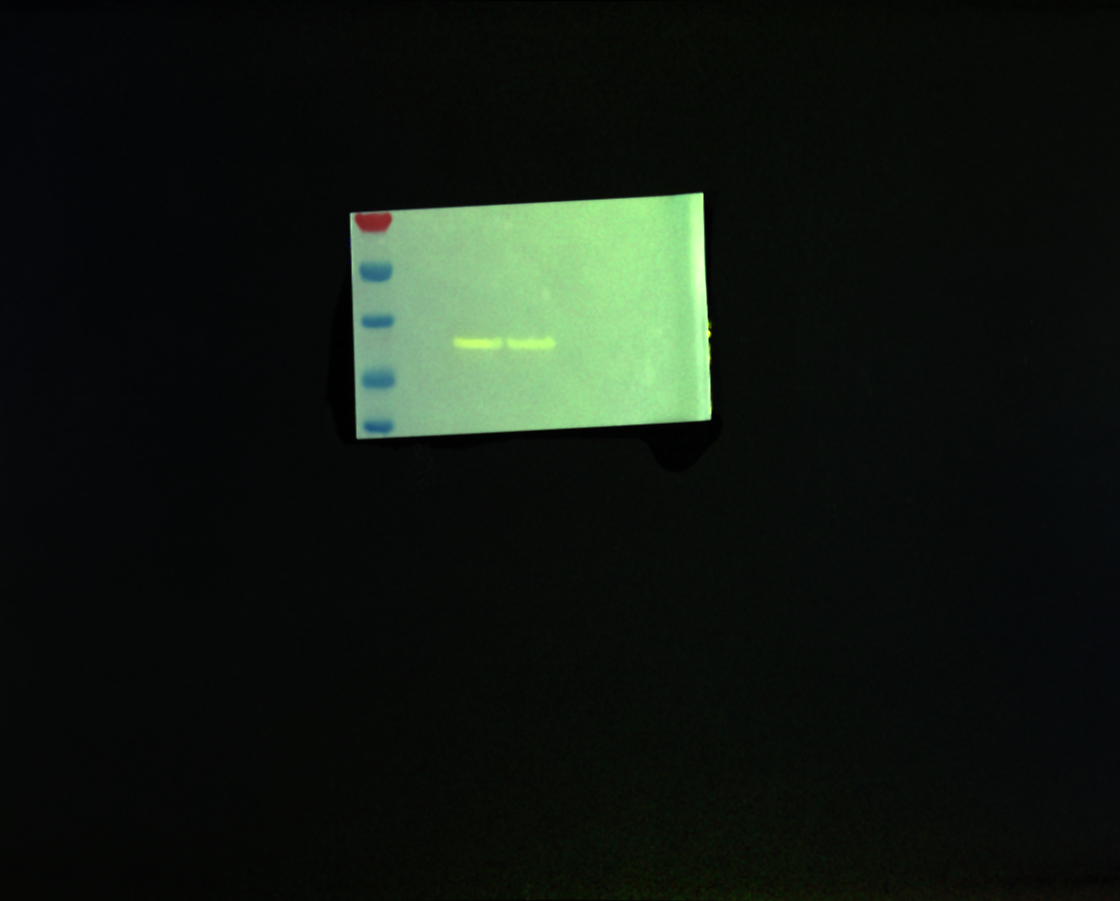

Supplement: Supplementary file 9 — Unprocessed western blots for Fig. 2g,n. [file 42255_2025_1225_MOESM9_ESM.zip › Zuhra_Unmodified_WesternBlot_Fig2/Zuhra_WesternBlot_Mani_Fig2_n/OE-TST/Fig2n_OE-TST_TST_marker.tif]

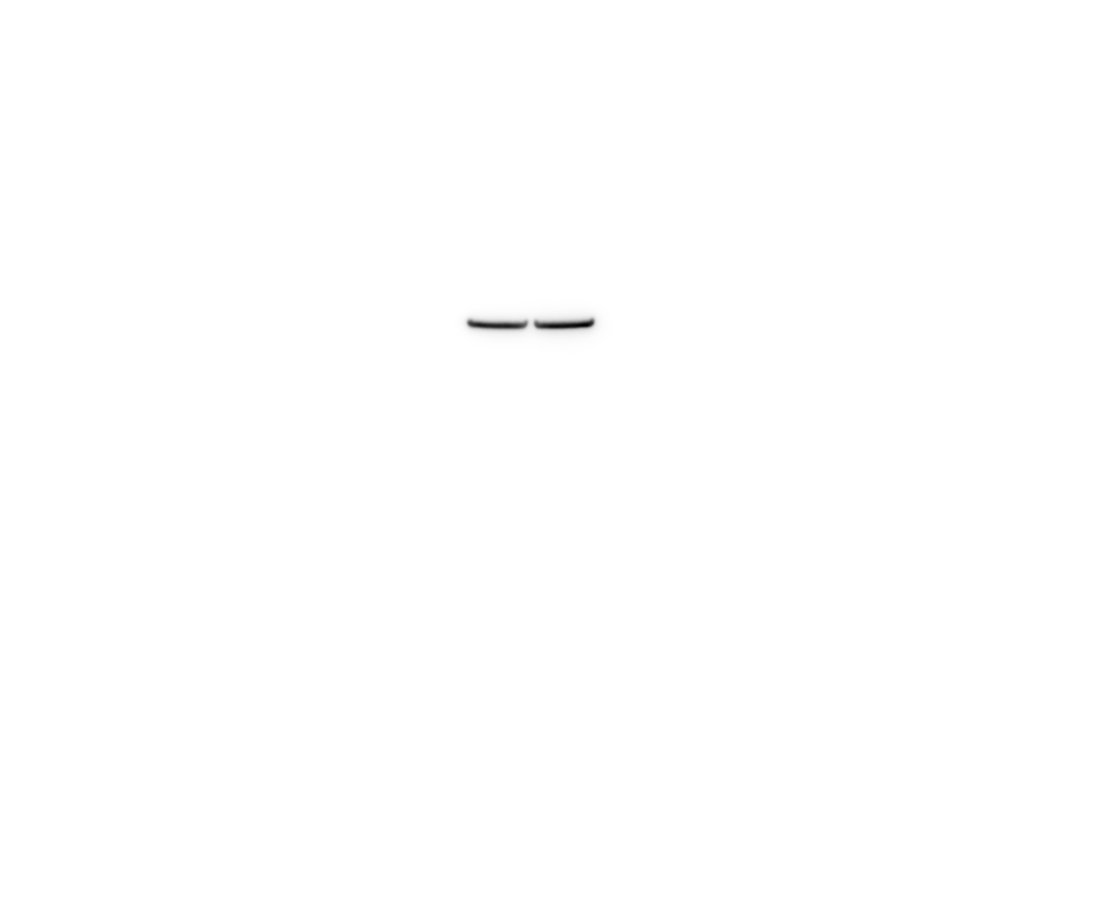

Supplement: Supplementary file 9 — Unprocessed western blots for Fig. 2g,n. [file 42255_2025_1225_MOESM9_ESM.zip › Zuhra_Unmodified_WesternBlot_Fig2/Zuhra_WesternBlot_Mani_Fig2_n/shTST/Fig2n_shTST_actin.jpg]

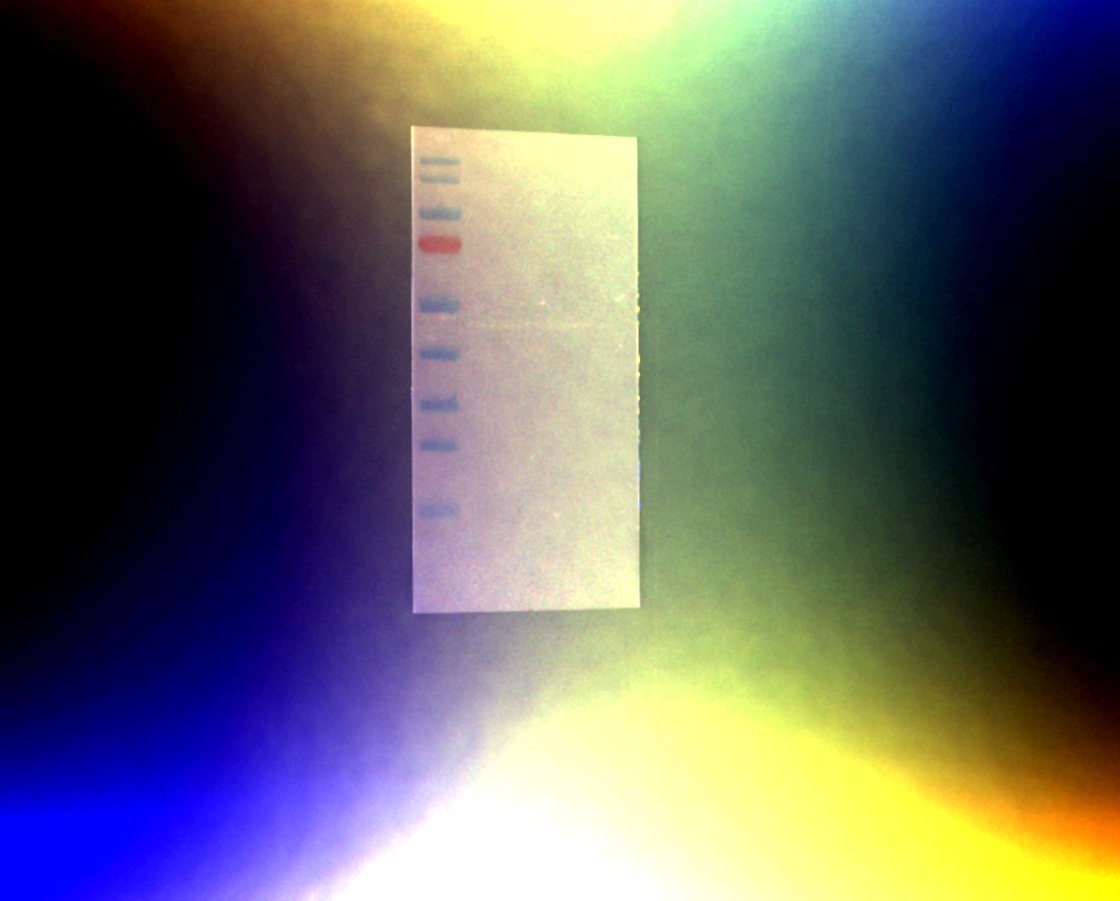

Supplement: Supplementary file 9 — Unprocessed western blots for Fig. 2g,n. [file 42255_2025_1225_MOESM9_ESM.zip › Zuhra_Unmodified_WesternBlot_Fig2/Zuhra_WesternBlot_Mani_Fig2_n/shTST/Fig2n_shTST_actin_marker.jpg]

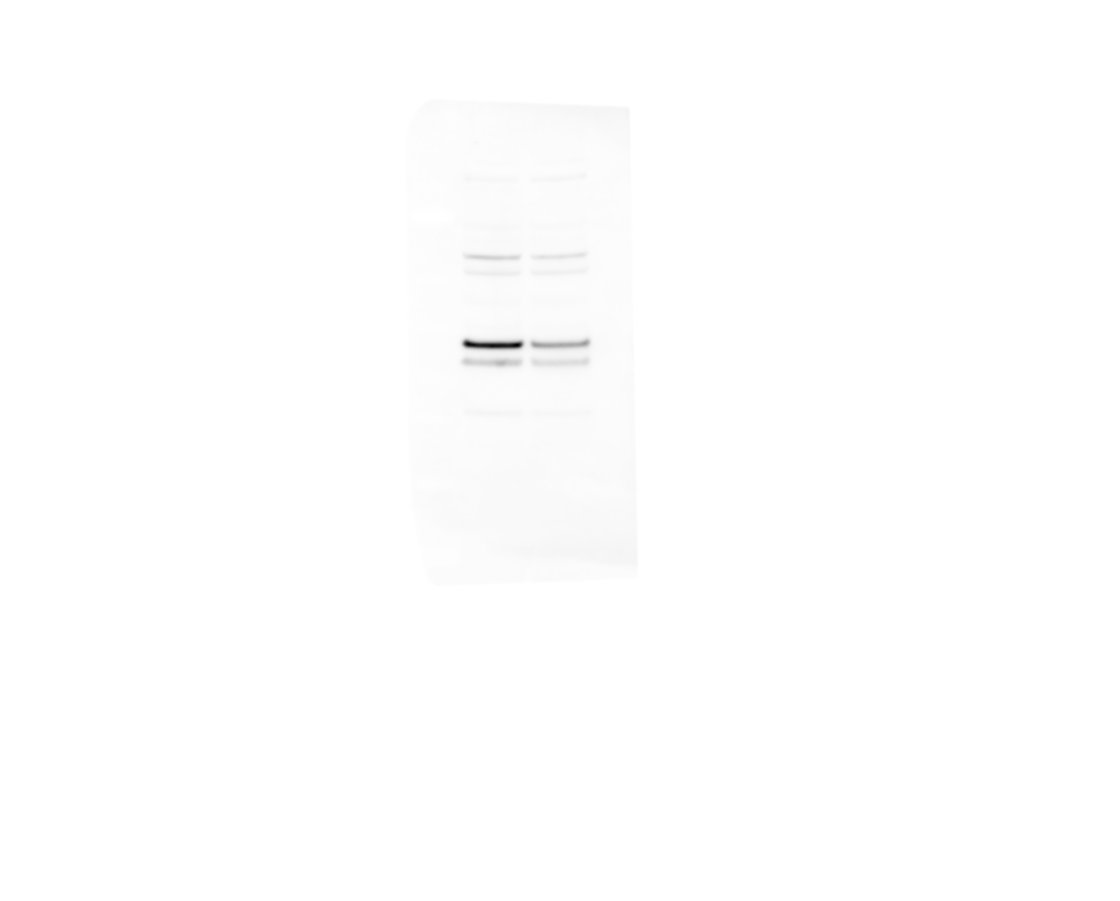

Supplement: Supplementary file 9 — Unprocessed western blots for Fig. 2g,n. [file 42255_2025_1225_MOESM9_ESM.zip › Zuhra_Unmodified_WesternBlot_Fig2/Zuhra_WesternBlot_Mani_Fig2_n/shTST/Fig2n_shTST_TST.jpg]

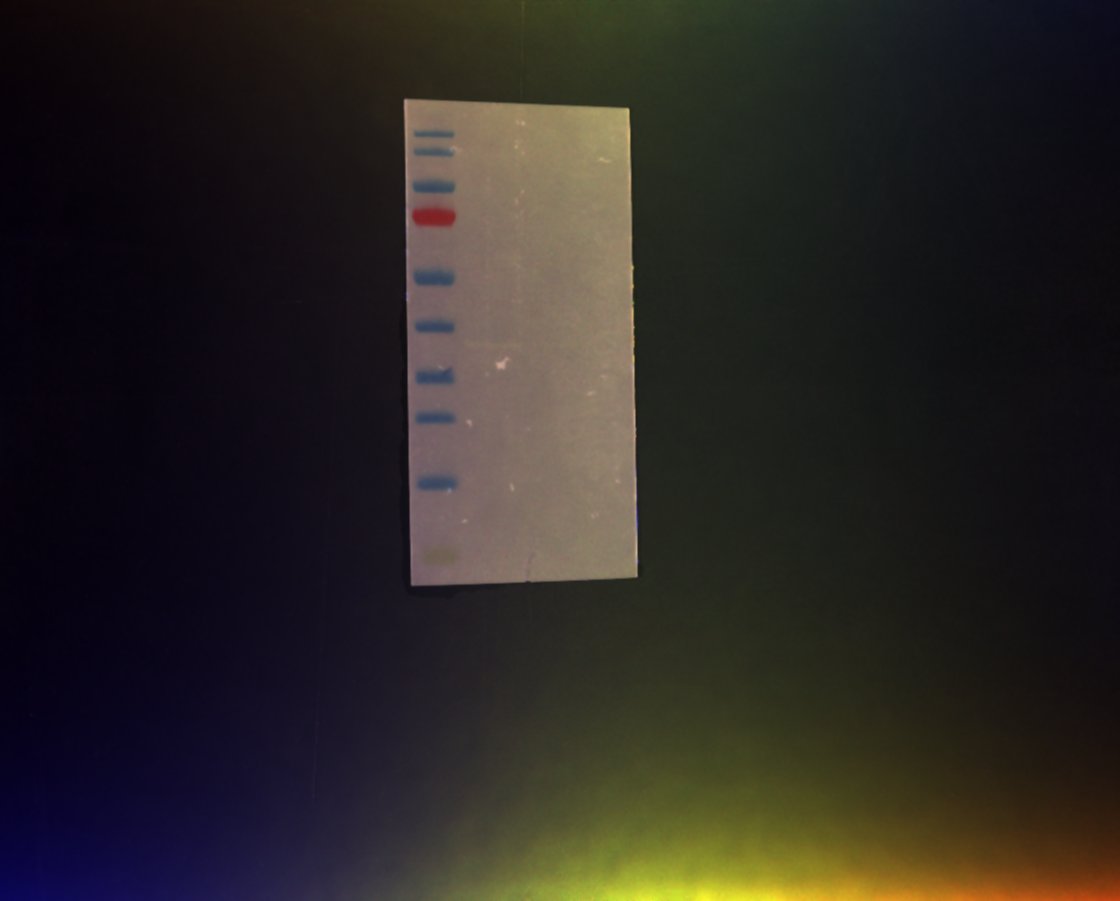

Supplement: Supplementary file 9 — Unprocessed western blots for Fig. 2g,n. [file 42255_2025_1225_MOESM9_ESM.zip › Zuhra_Unmodified_WesternBlot_Fig2/Zuhra_WesternBlot_Mani_Fig2_n/shTST/Fig2n_shTST_TST_marker.jpg]

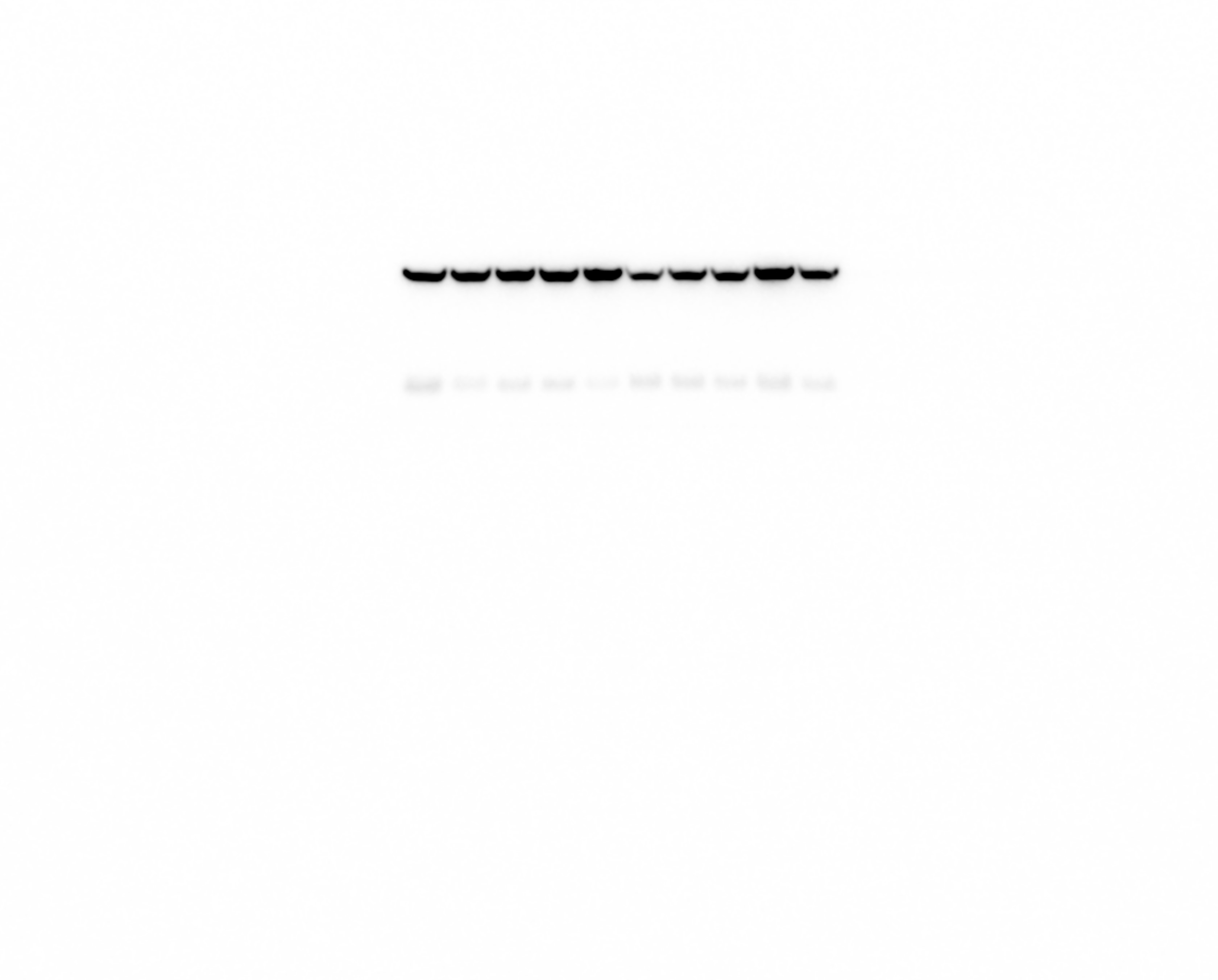

Supplement: Supplementary file 11 — Unprocessed western blots for Fig. 3b,c,e. [file 42255_2025_1225_MOESM11_ESM.zip › Zuhra_Unmodified_WesternBlot_Fig3/Zuhra_WesternBlot_Main_Fig3_b/actin.jpg]

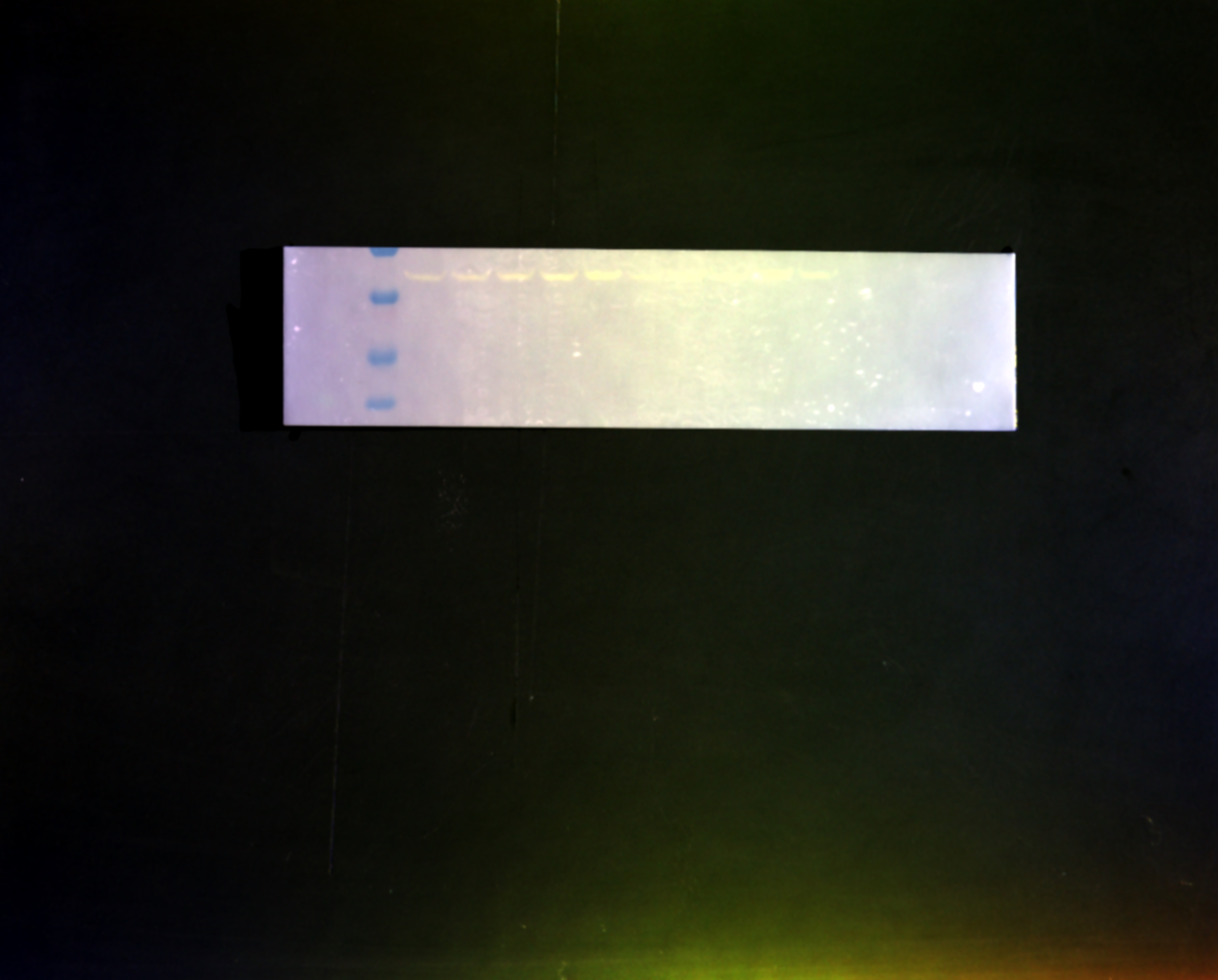

Supplement: Supplementary file 11 — Unprocessed western blots for Fig. 3b,c,e. [file 42255_2025_1225_MOESM11_ESM.zip › Zuhra_Unmodified_WesternBlot_Fig3/Zuhra_WesternBlot_Main_Fig3_b/actin_marker.jpg]

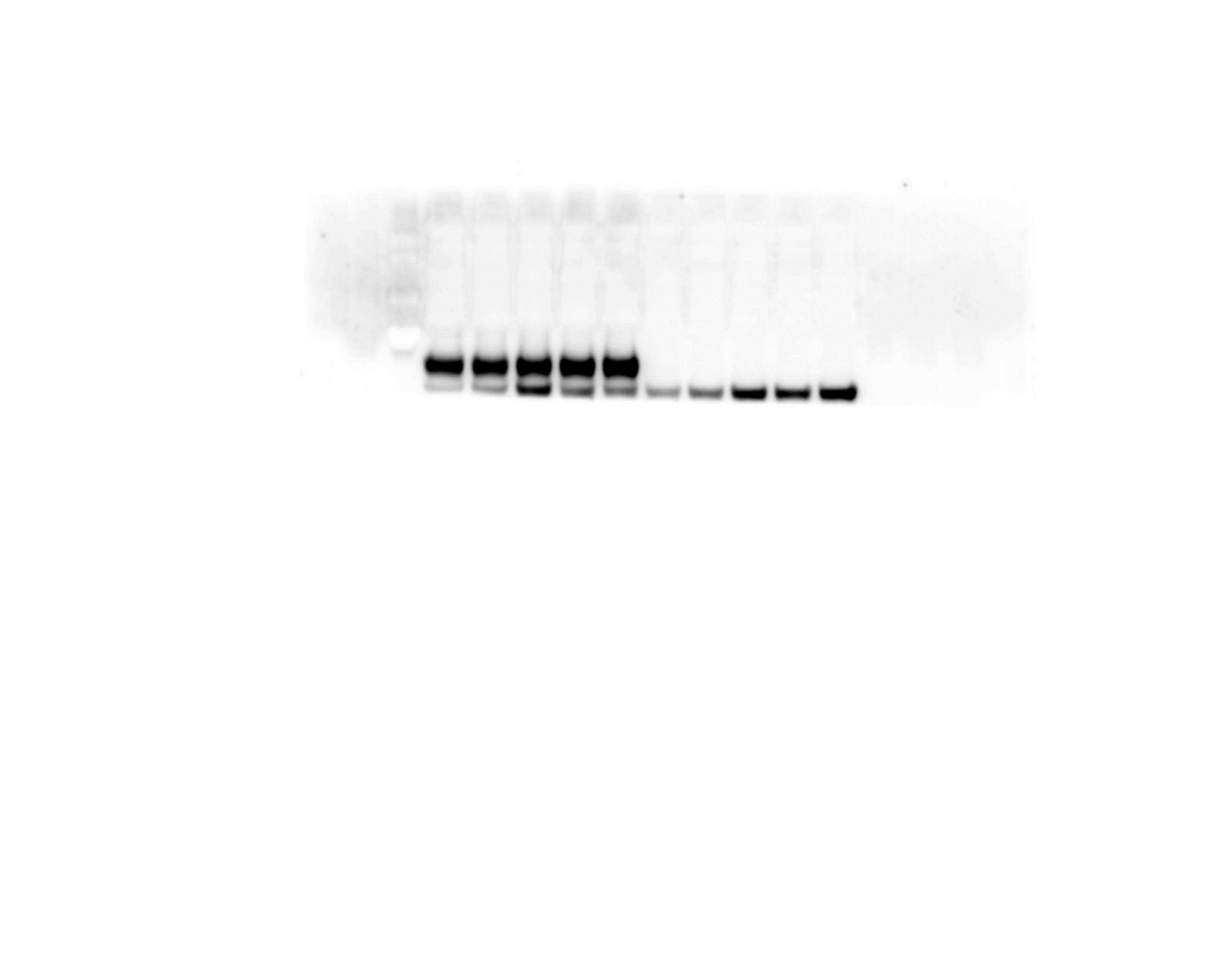

Supplement: Supplementary file 11 — Unprocessed western blots for Fig. 3b,c,e. [file 42255_2025_1225_MOESM11_ESM.zip › Zuhra_Unmodified_WesternBlot_Fig3/Zuhra_WesternBlot_Main_Fig3_b/MPO.jpg]

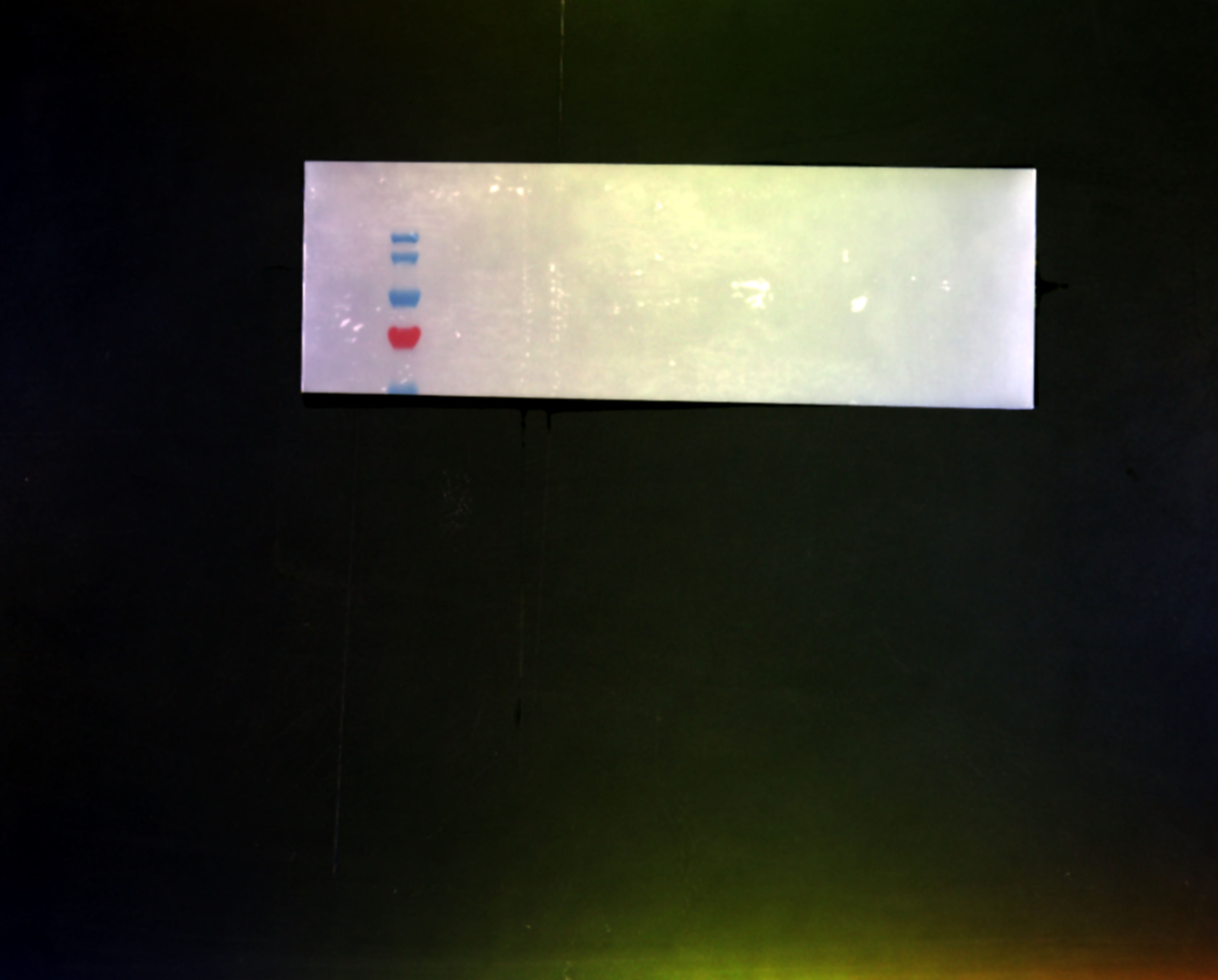

Supplement: Supplementary file 11 — Unprocessed western blots for Fig. 3b,c,e. [file 42255_2025_1225_MOESM11_ESM.zip › Zuhra_Unmodified_WesternBlot_Fig3/Zuhra_WesternBlot_Main_Fig3_b/MPO_marker.jpg]

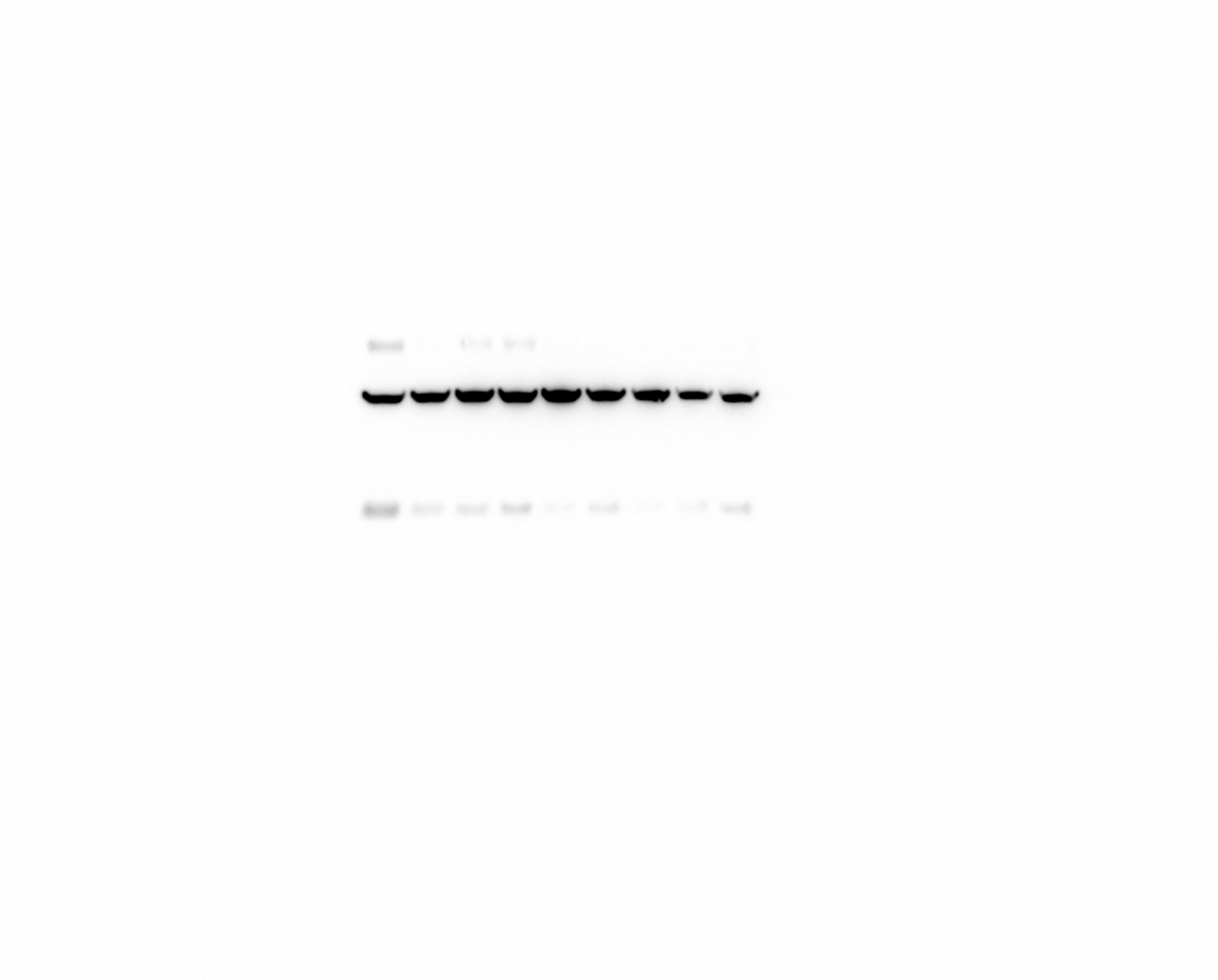

Supplement: Supplementary file 11 — Unprocessed western blots for Fig. 3b,c,e. [file 42255_2025_1225_MOESM11_ESM.zip › Zuhra_Unmodified_WesternBlot_Fig3/Zuhra_WesternBlot_Main_Fig3_c/actin.jpg]

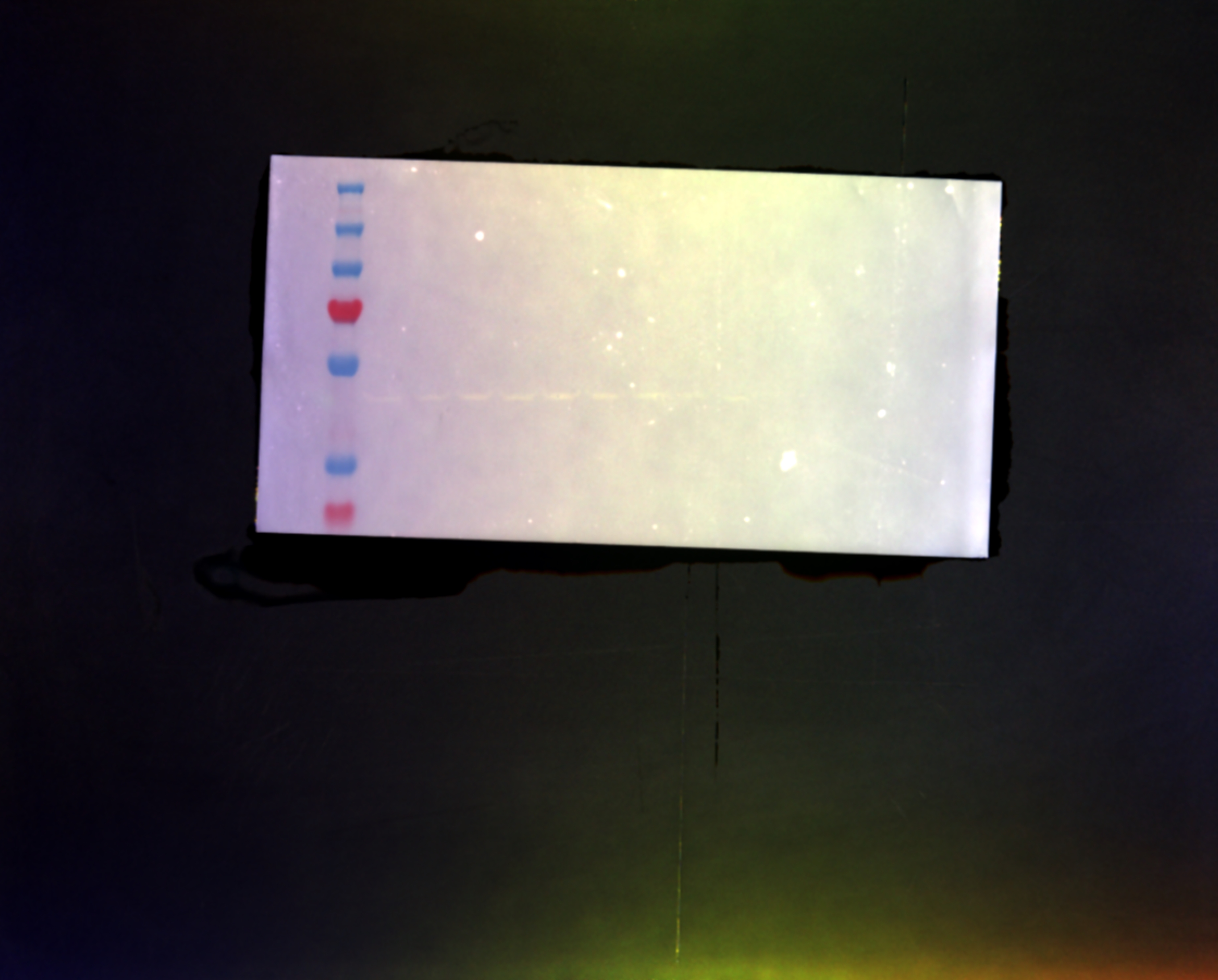

Supplement: Supplementary file 11 — Unprocessed western blots for Fig. 3b,c,e. [file 42255_2025_1225_MOESM11_ESM.zip › Zuhra_Unmodified_WesternBlot_Fig3/Zuhra_WesternBlot_Main_Fig3_c/actin_marker.jpg]

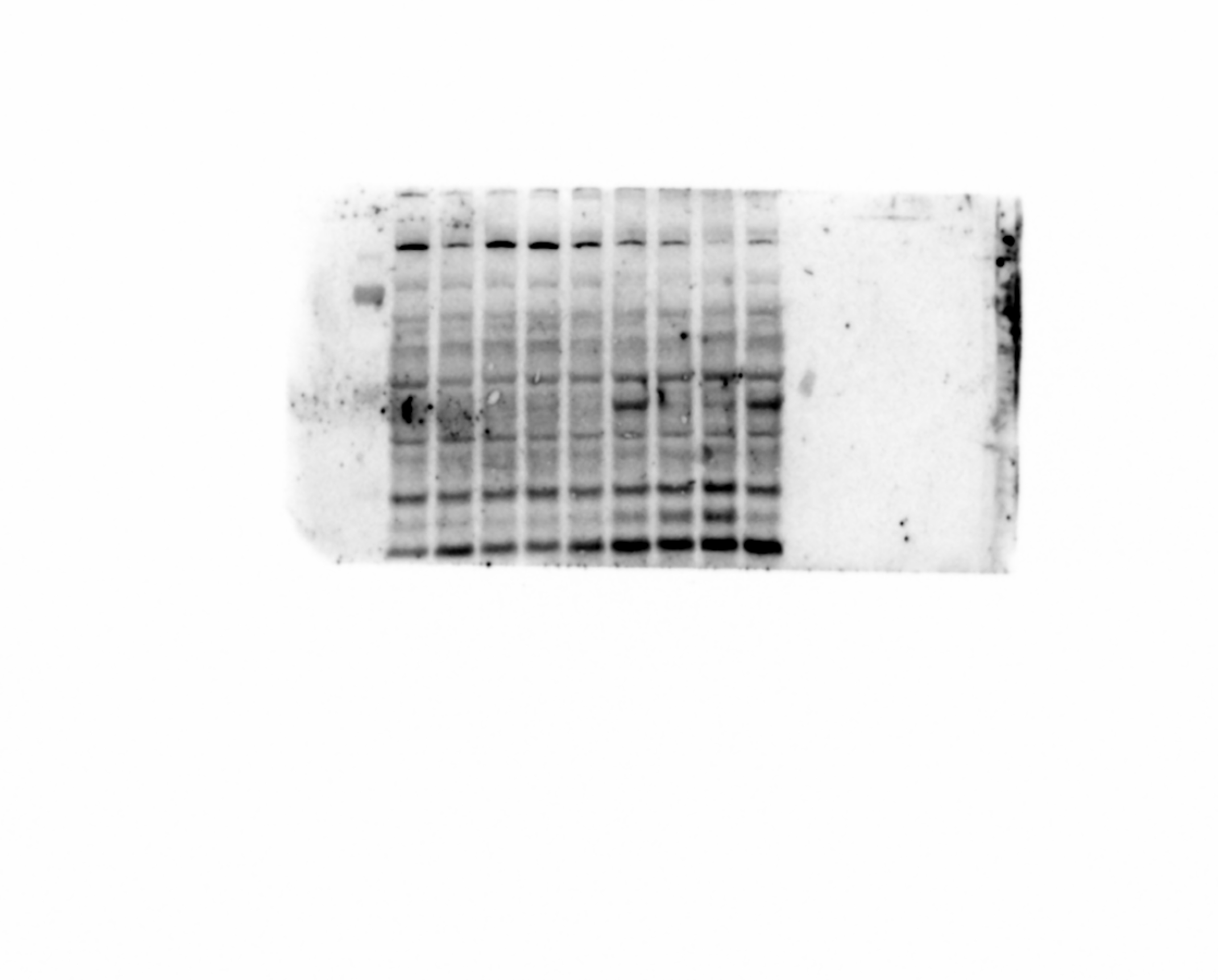

Supplement: Supplementary file 11 — Unprocessed western blots for Fig. 3b,c,e. [file 42255_2025_1225_MOESM11_ESM.zip › Zuhra_Unmodified_WesternBlot_Fig3/Zuhra_WesternBlot_Main_Fig3_c/PXDN.jpg]

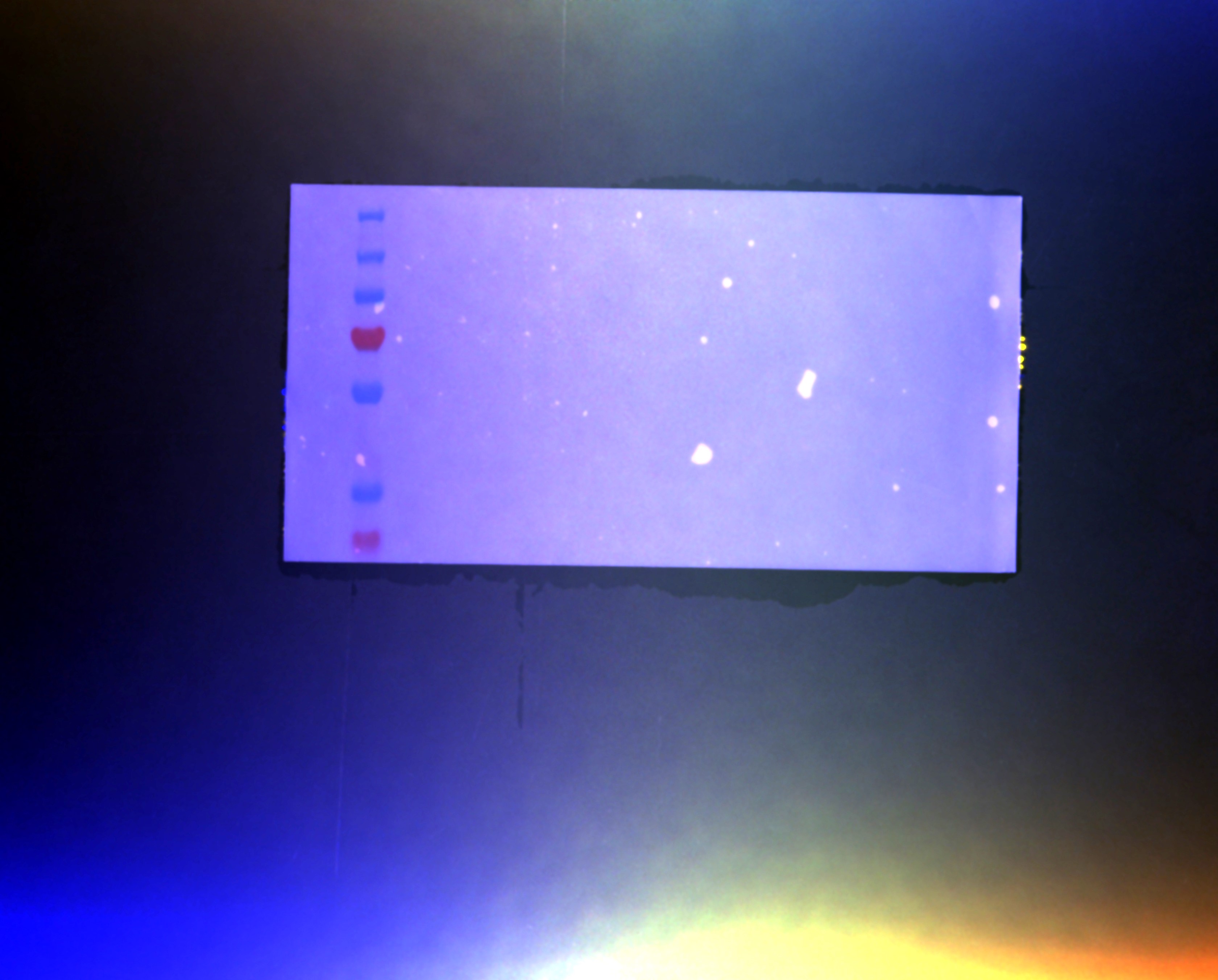

Supplement: Supplementary file 11 — Unprocessed western blots for Fig. 3b,c,e. [file 42255_2025_1225_MOESM11_ESM.zip › Zuhra_Unmodified_WesternBlot_Fig3/Zuhra_WesternBlot_Main_Fig3_c/PXDN_marker.jpg]

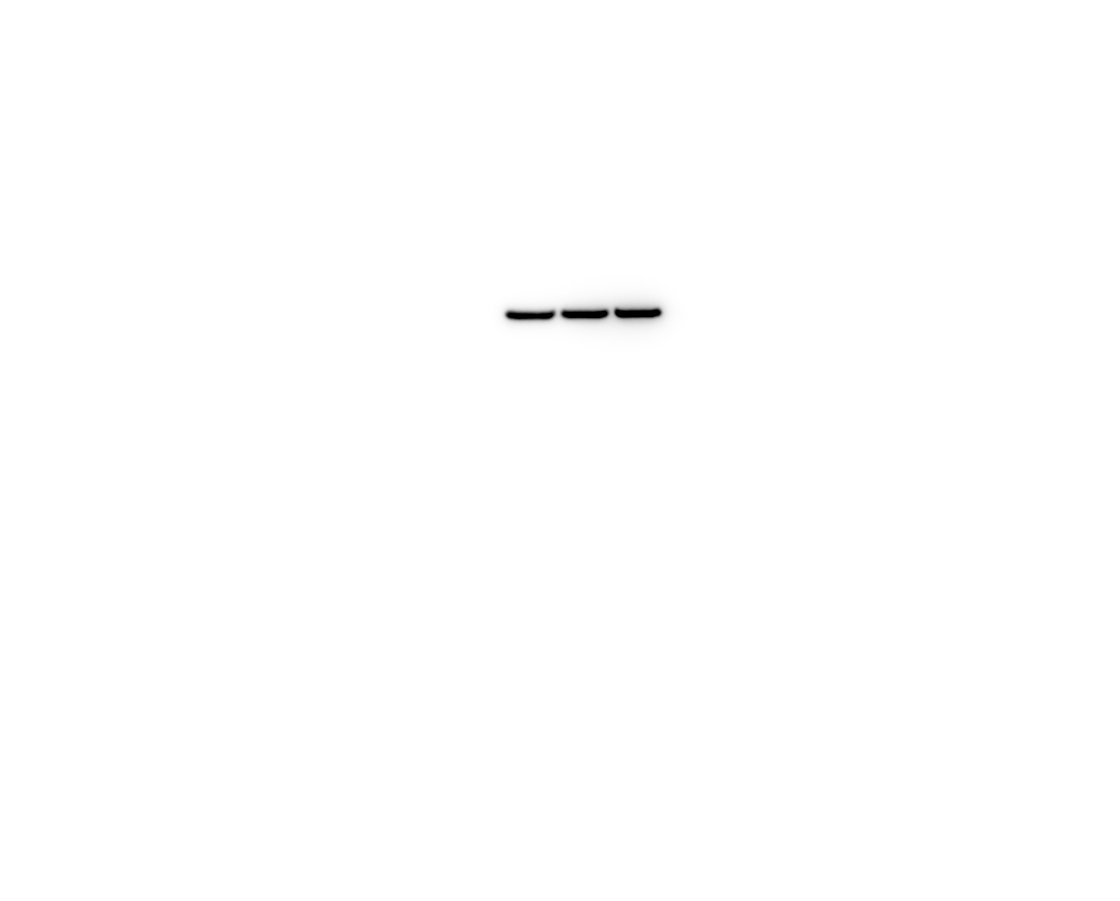

Supplement: Supplementary file 11 — Unprocessed western blots for Fig. 3b,c,e. [file 42255_2025_1225_MOESM11_ESM.zip › Zuhra_Unmodified_WesternBlot_Fig3/Zuhra_WesternBlot_Main_Fig3_e/actin.jpg]

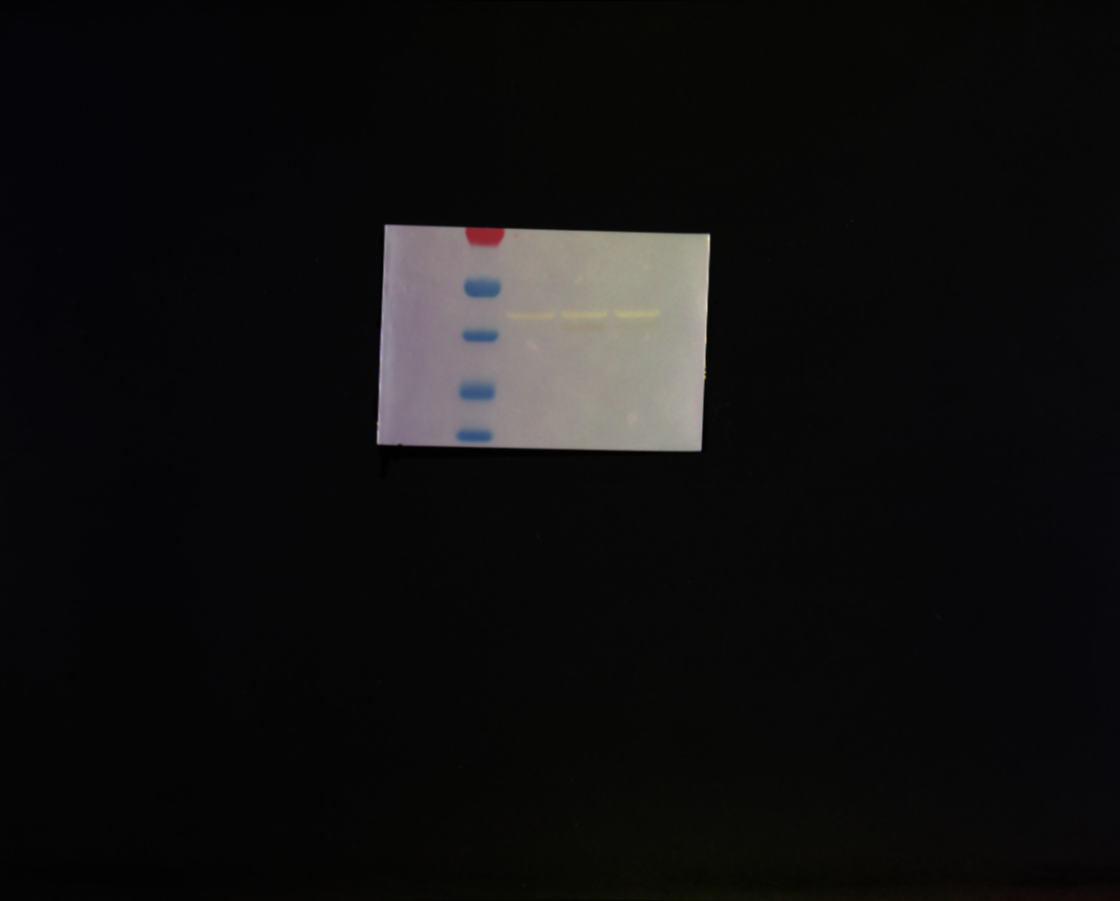

Supplement: Supplementary file 11 — Unprocessed western blots for Fig. 3b,c,e. [file 42255_2025_1225_MOESM11_ESM.zip › Zuhra_Unmodified_WesternBlot_Fig3/Zuhra_WesternBlot_Main_Fig3_e/actin_marker.tif]

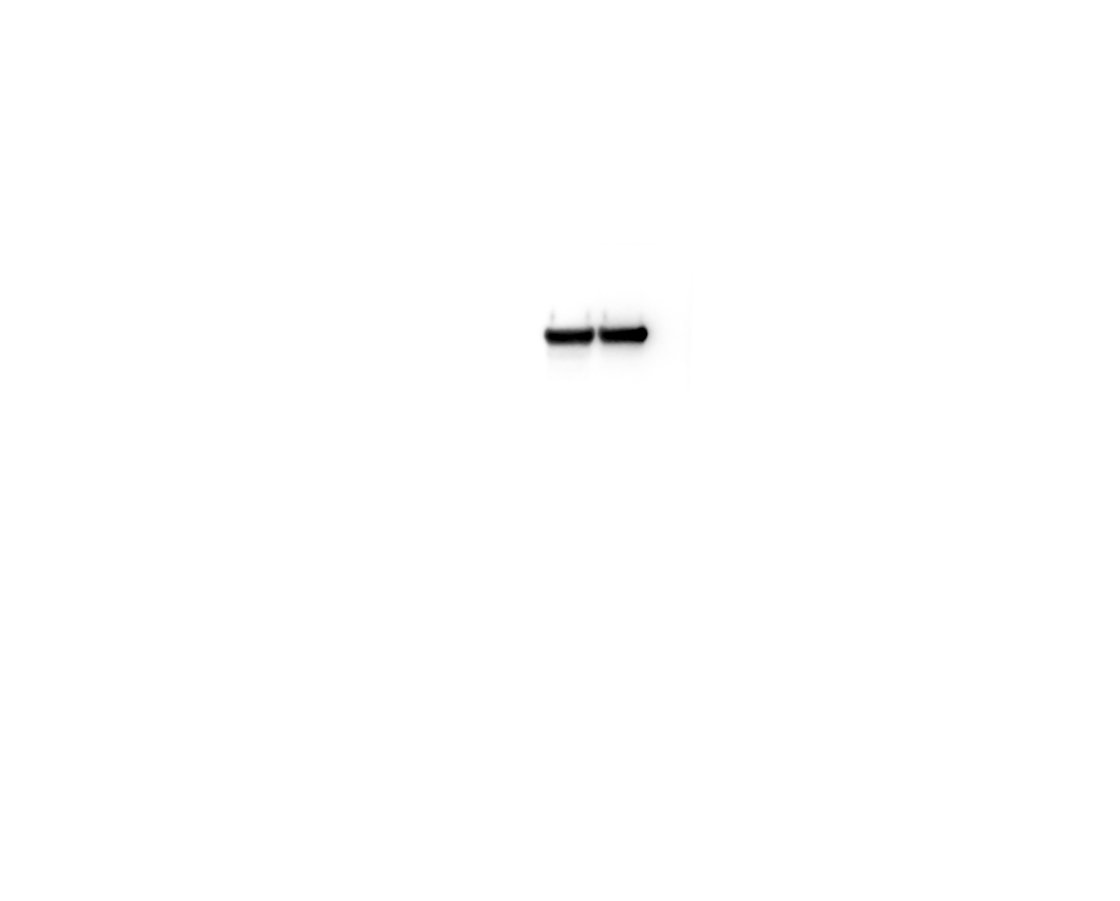

Supplement: Supplementary file 11 — Unprocessed western blots for Fig. 3b,c,e. [file 42255_2025_1225_MOESM11_ESM.zip › Zuhra_Unmodified_WesternBlot_Fig3/Zuhra_WesternBlot_Main_Fig3_e/CynD.jpg]

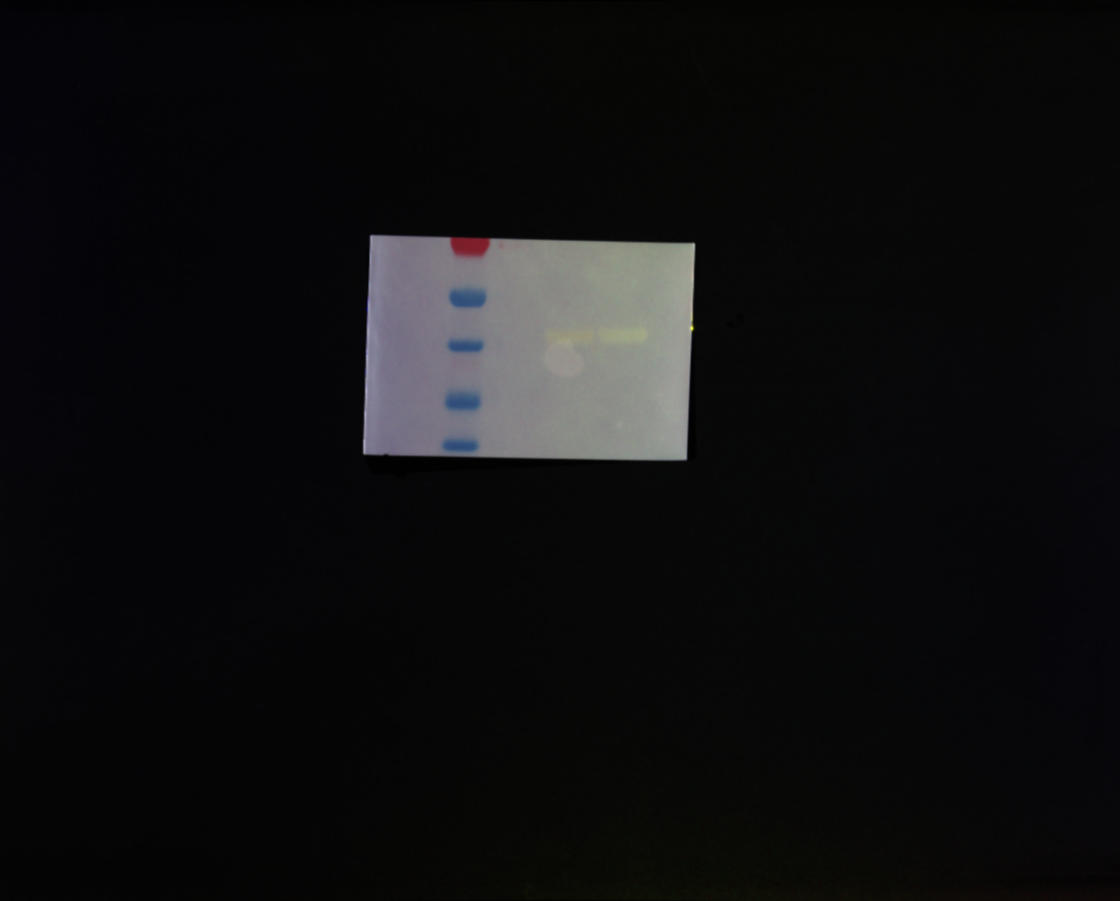

Supplement: Supplementary file 11 — Unprocessed western blots for Fig. 3b,c,e. [file 42255_2025_1225_MOESM11_ESM.zip › Zuhra_Unmodified_WesternBlot_Fig3/Zuhra_WesternBlot_Main_Fig3_e/CynD_marker.tif]

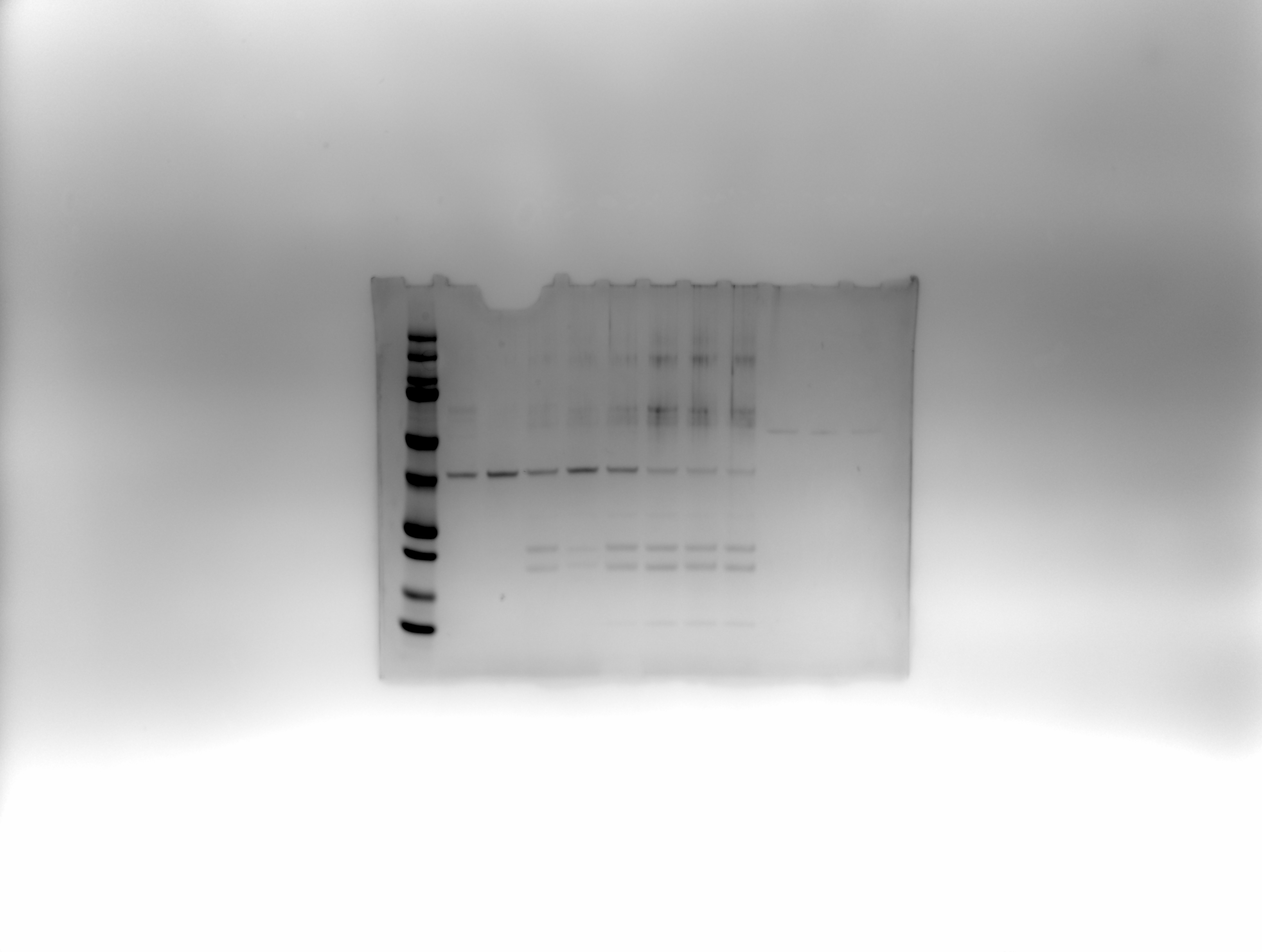

Supplement: Supplementary file 13 — Unprocessed two-dimensional SDS–PAGE for Fig. 4k,l. [file 42255_2025_1225_MOESM13_ESM.zip › Zuhra_Unmodified_Gels_Fig4/SDS-PAGE_GAPDH_Experiment1.jpg]

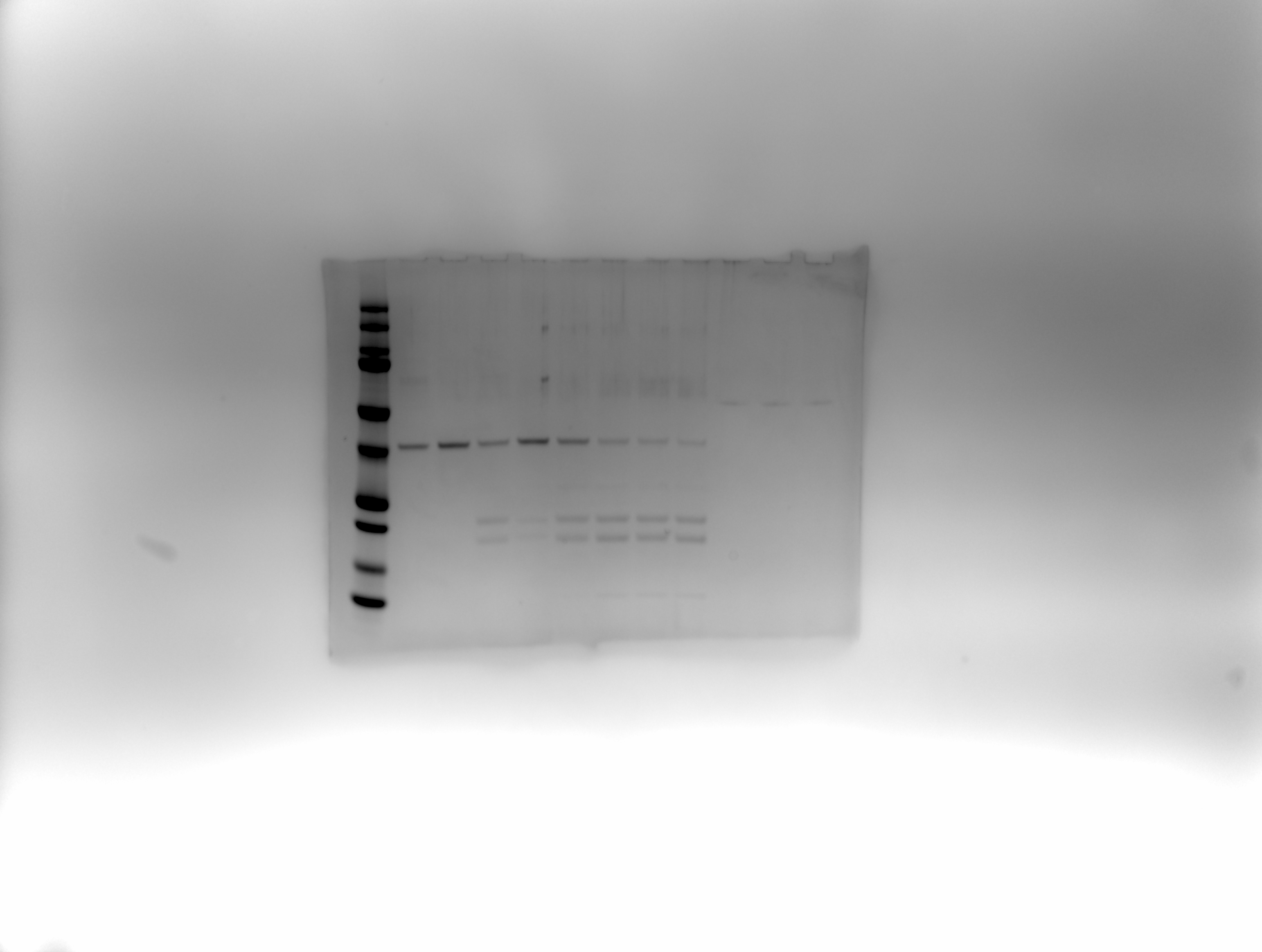

Supplement: Supplementary file 13 — Unprocessed two-dimensional SDS–PAGE for Fig. 4k,l. [file 42255_2025_1225_MOESM13_ESM.zip › Zuhra_Unmodified_Gels_Fig4/SDS-PAGE_GAPDH_Experiment2.jpg]

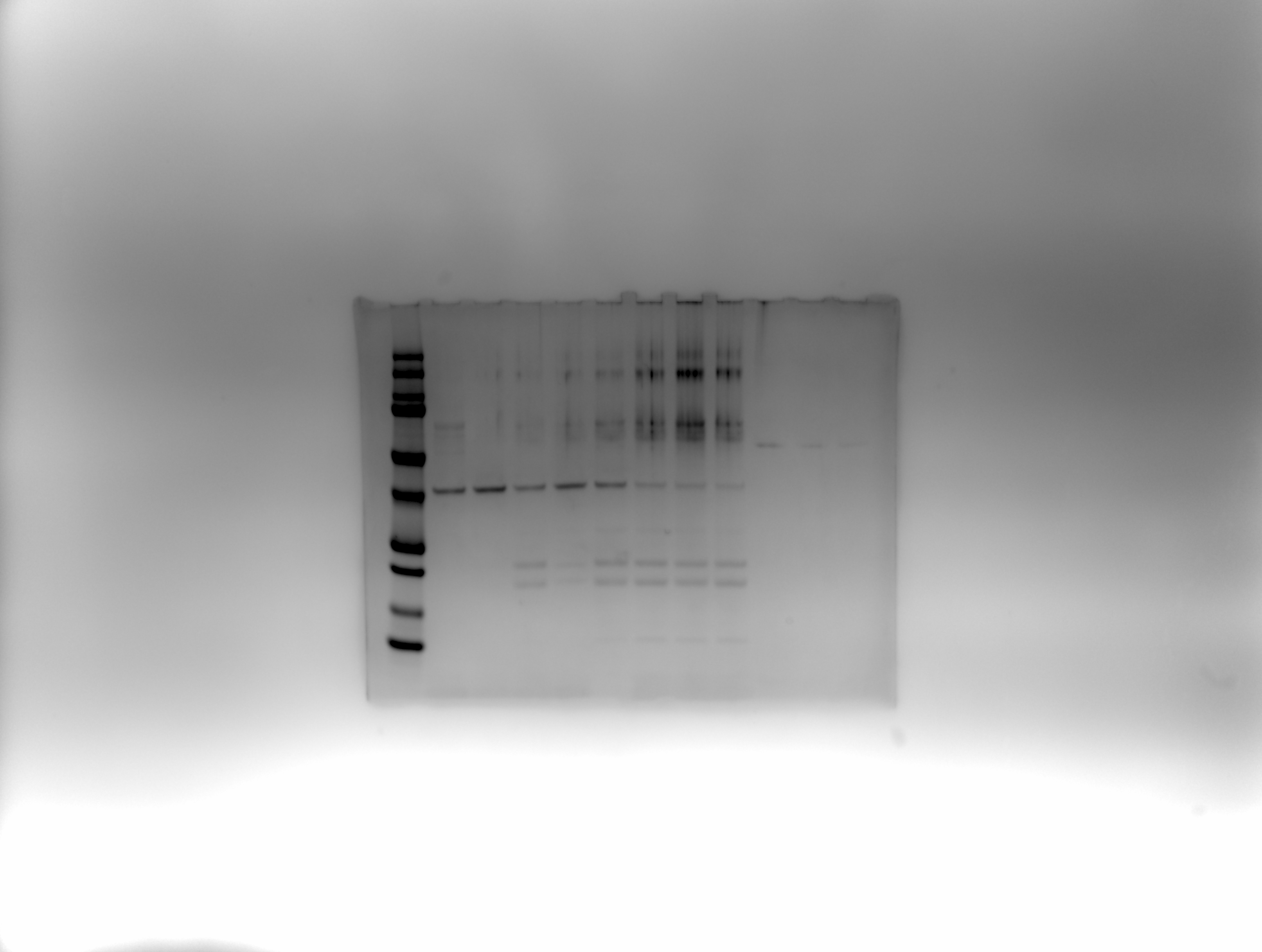

Supplement: Supplementary file 13 — Unprocessed two-dimensional SDS–PAGE for Fig. 4k,l. [file 42255_2025_1225_MOESM13_ESM.zip › Zuhra_Unmodified_Gels_Fig4/SDS-PAGE_GAPDH_Experiment3.jpg]

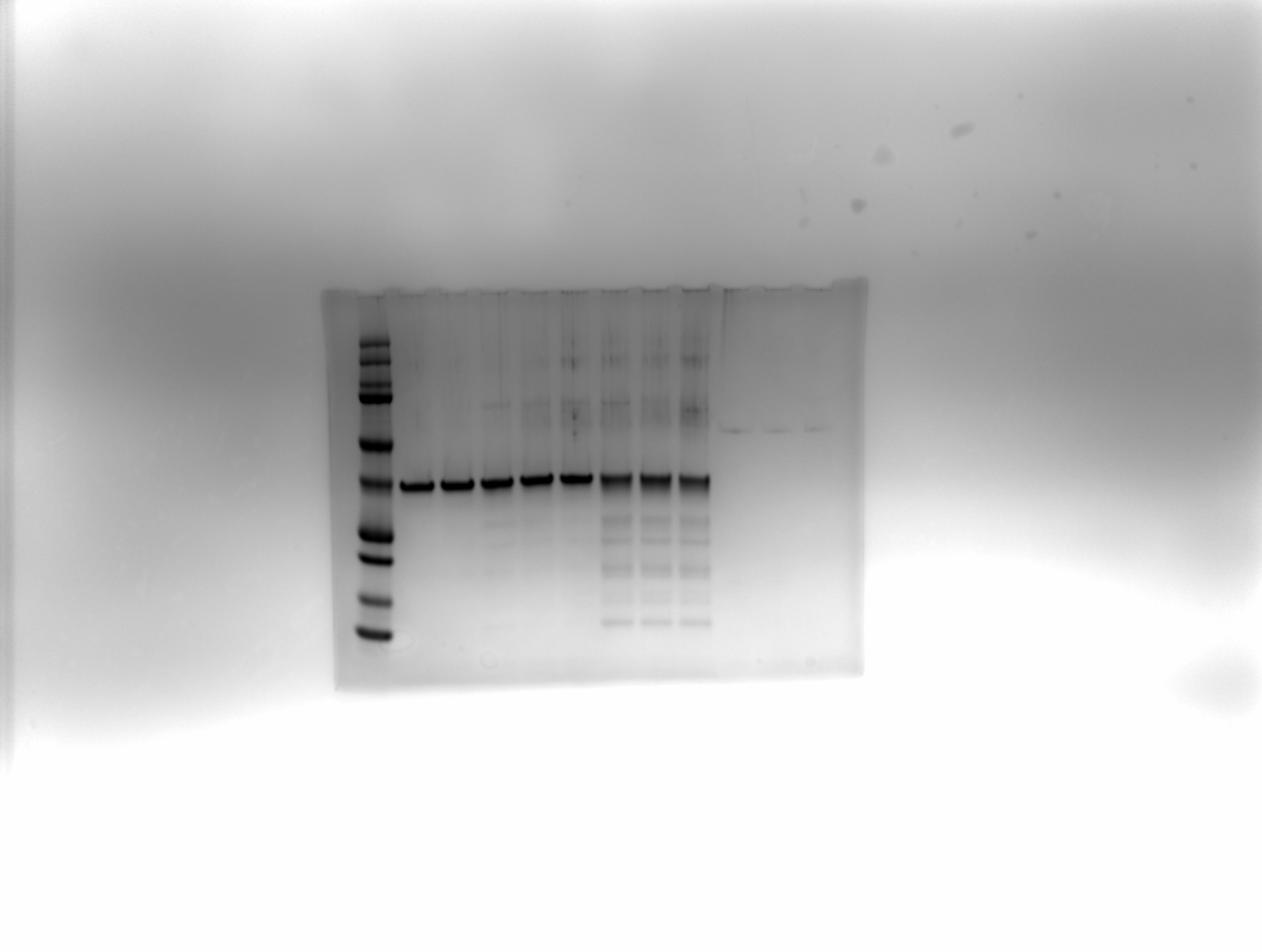

Supplement: Supplementary file 13 — Unprocessed two-dimensional SDS–PAGE for Fig. 4k,l. [file 42255_2025_1225_MOESM13_ESM.zip › Zuhra_Unmodified_Gels_Fig4/SDS-PAGE_GPDH_Experiment1.jpg]

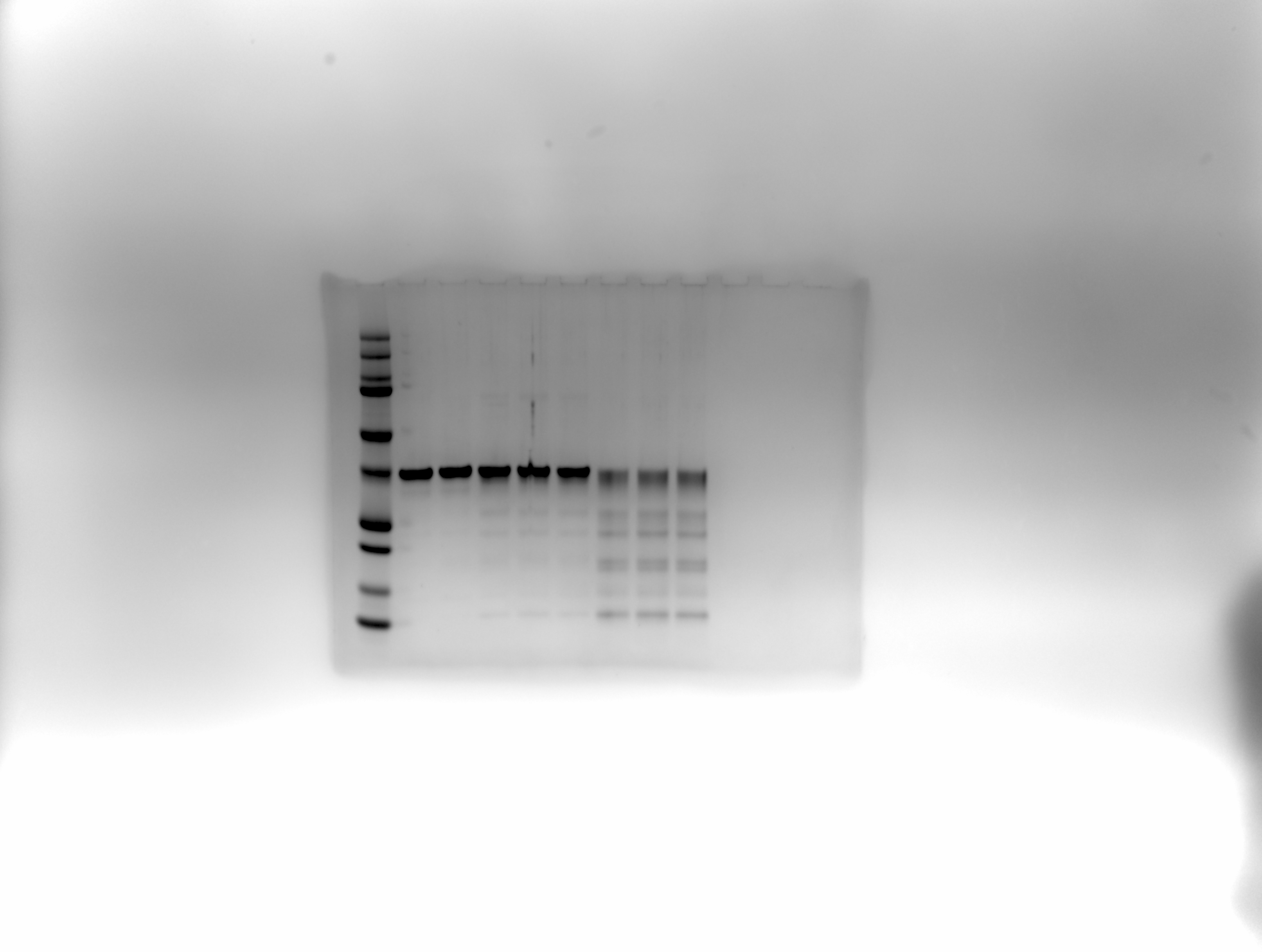

Supplement: Supplementary file 13 — Unprocessed two-dimensional SDS–PAGE for Fig. 4k,l. [file 42255_2025_1225_MOESM13_ESM.zip › Zuhra_Unmodified_Gels_Fig4/SDS-PAGE_GPDH_Experiment2.jpg]

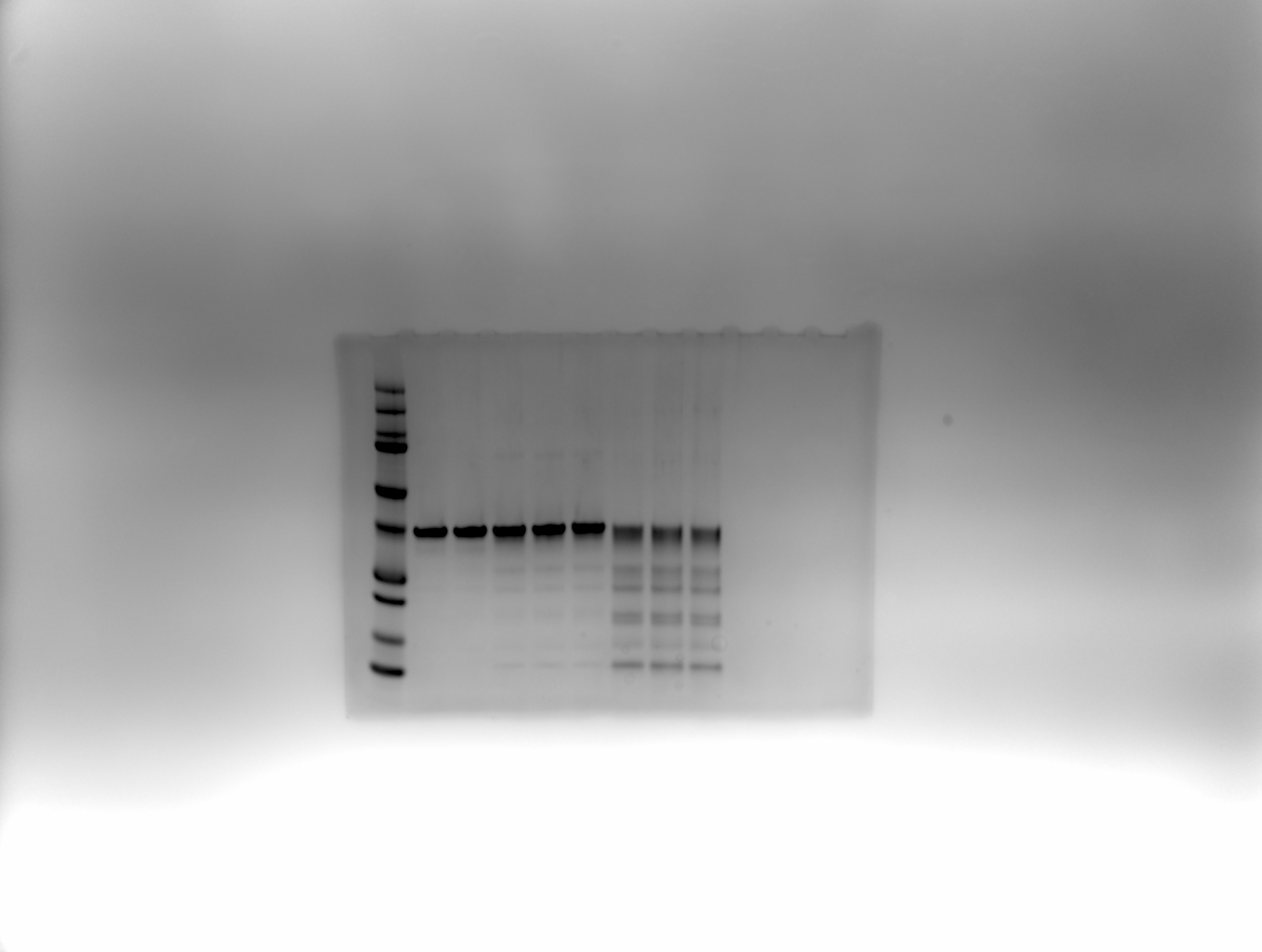

Supplement: Supplementary file 13 — Unprocessed two-dimensional SDS–PAGE for Fig. 4k,l. [file 42255_2025_1225_MOESM13_ESM.zip › Zuhra_Unmodified_Gels_Fig4/SDS-PAGE_GPDH_Experiment3.jpg]

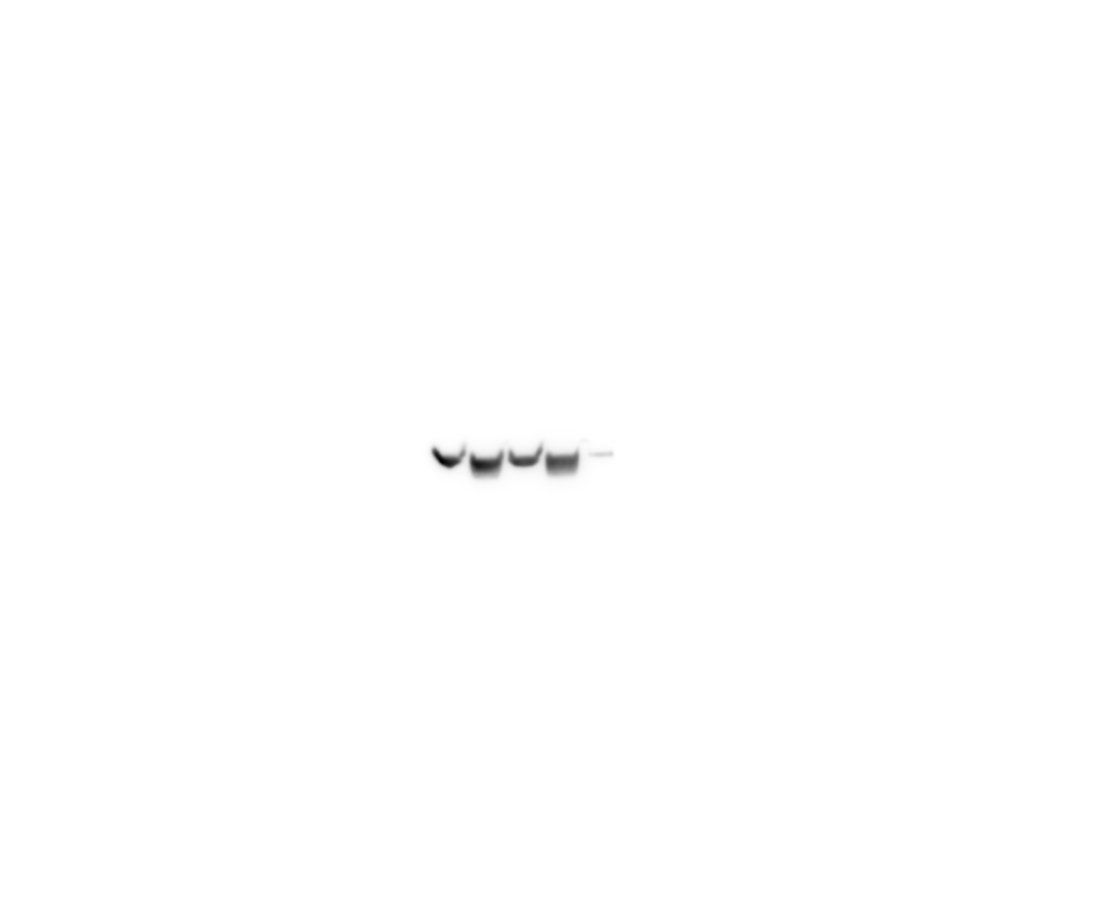

Supplement: Supplementary file 17 — Unprocessed western blots for Fig. 7b. [file 42255_2025_1225_MOESM17_ESM.zip › Zuhra_Unmodified_WesternBlot_Fig7/Experiment1_Hypoxia_actin.jpg]

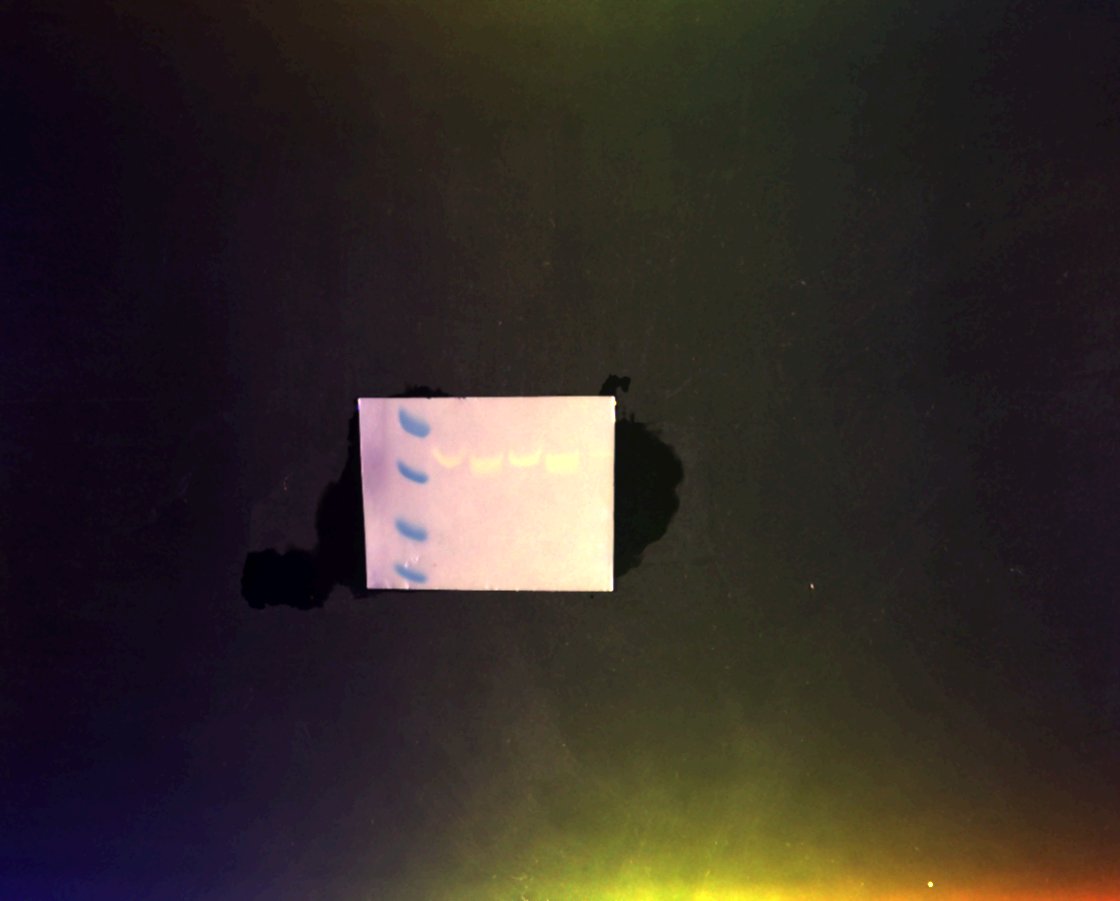

Supplement: Supplementary file 17 — Unprocessed western blots for Fig. 7b. [file 42255_2025_1225_MOESM17_ESM.zip › Zuhra_Unmodified_WesternBlot_Fig7/Experiment1_Hypoxia_actin_marker.jpg]

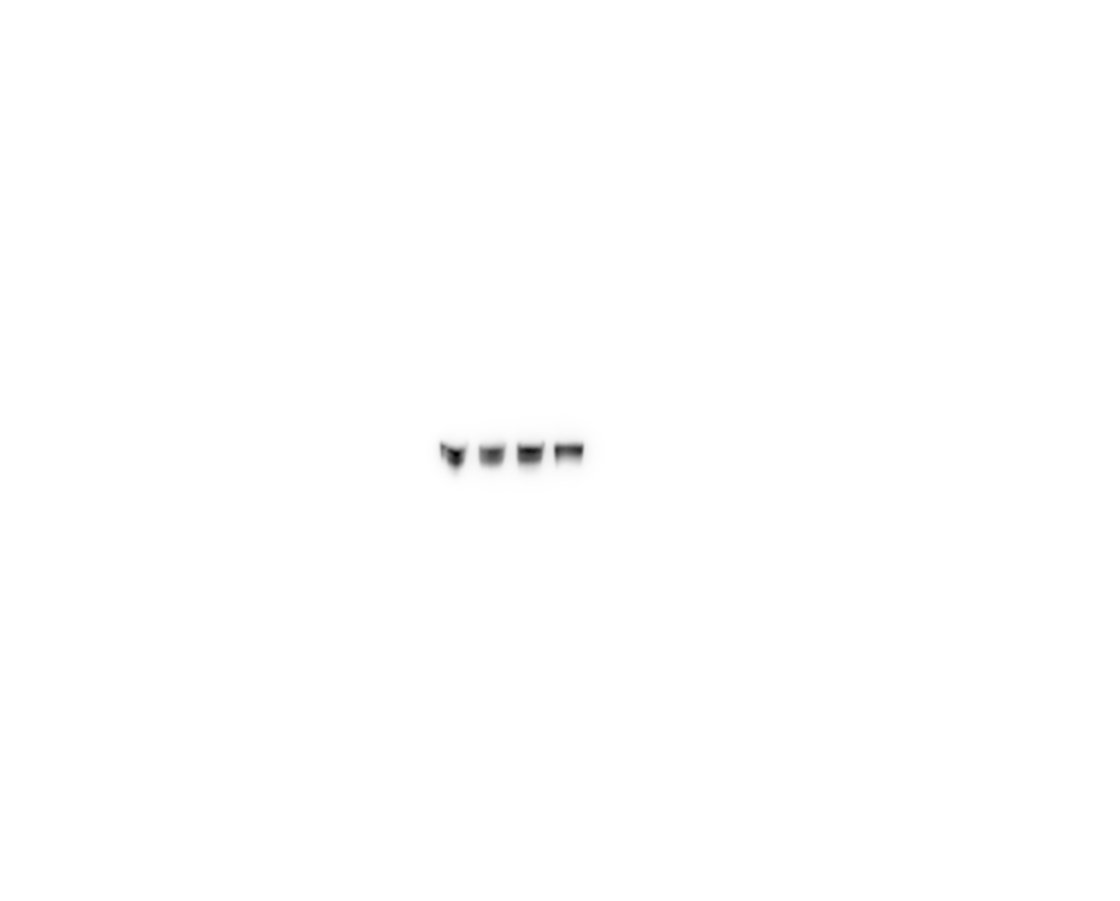

Supplement: Supplementary file 17 — Unprocessed western blots for Fig. 7b. [file 42255_2025_1225_MOESM17_ESM.zip › Zuhra_Unmodified_WesternBlot_Fig7/Experiment1_Hypoxia_Hif1a.jpg]

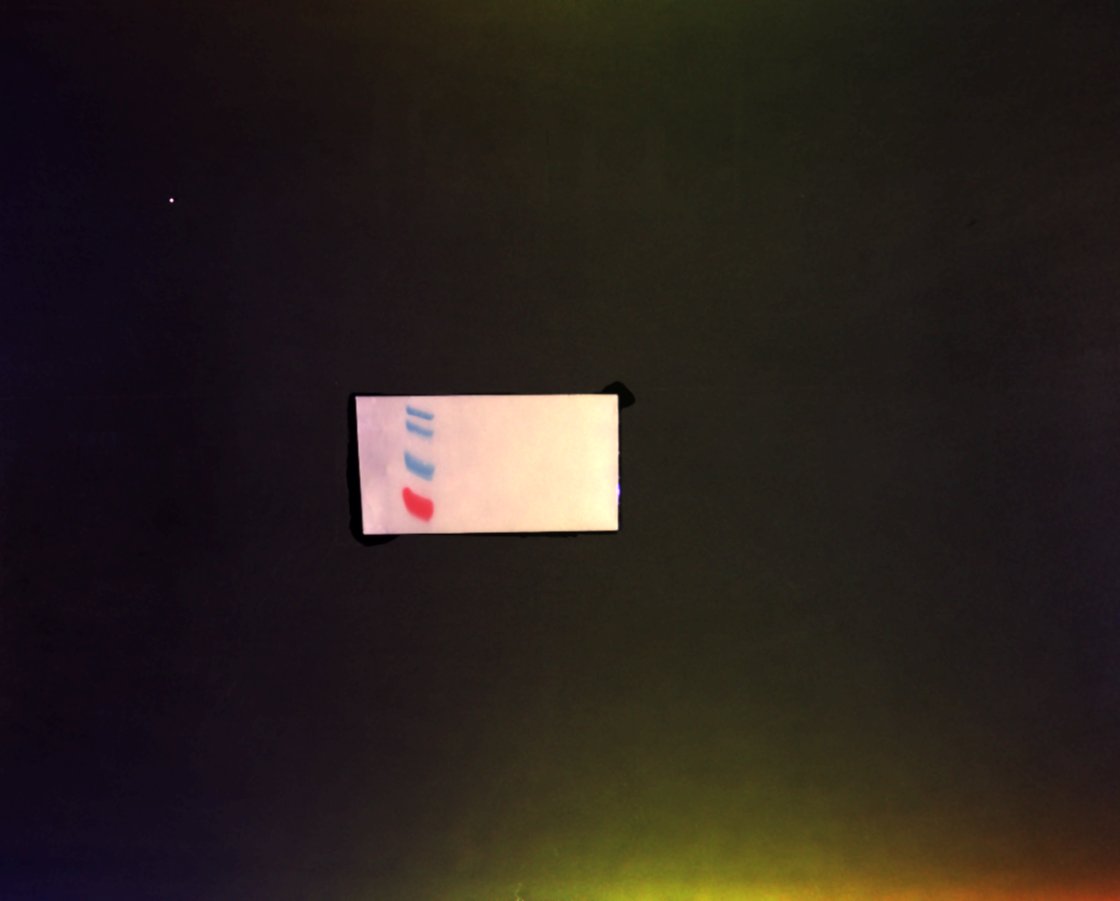

Supplement: Supplementary file 17 — Unprocessed western blots for Fig. 7b. [file 42255_2025_1225_MOESM17_ESM.zip › Zuhra_Unmodified_WesternBlot_Fig7/Experiment1_Hypoxia_Hif1a_marker.tif]

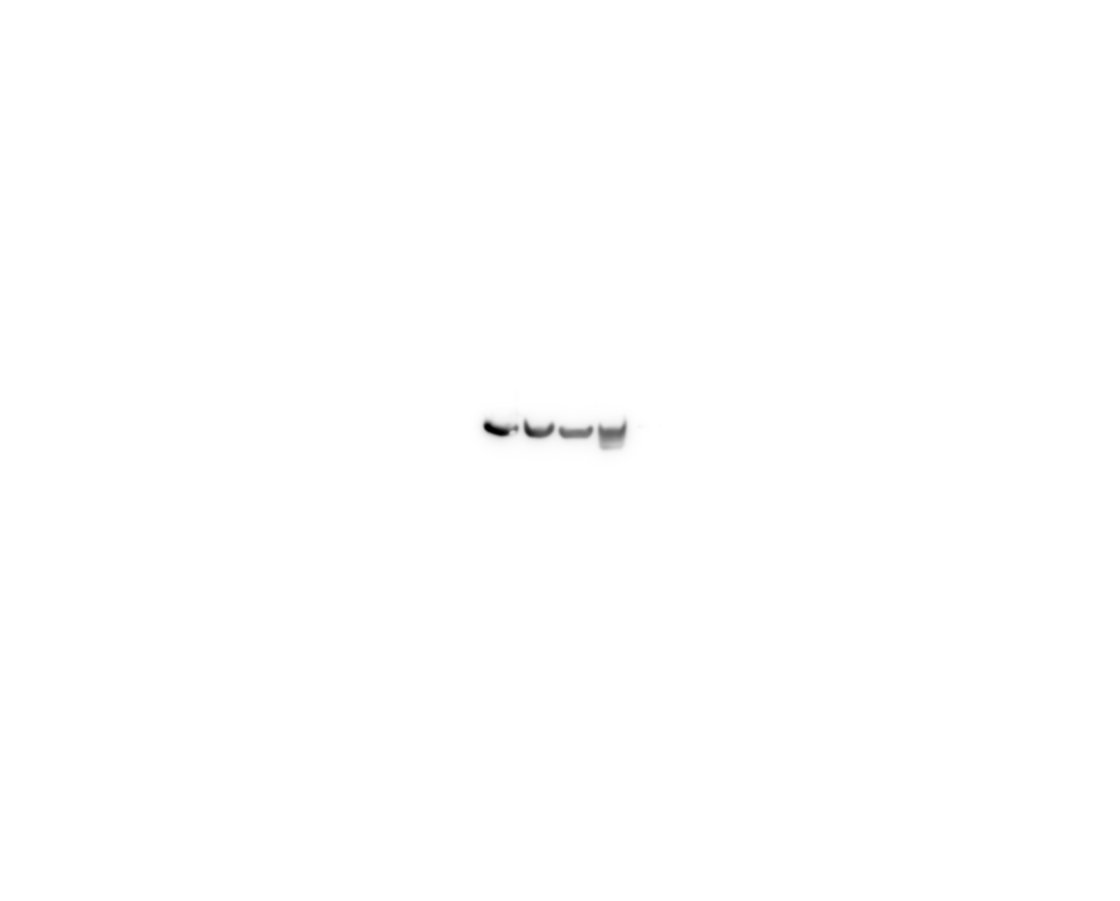

Supplement: Supplementary file 17 — Unprocessed western blots for Fig. 7b. [file 42255_2025_1225_MOESM17_ESM.zip › Zuhra_Unmodified_WesternBlot_Fig7/Experiment1_Normoxia_actin.jpg]

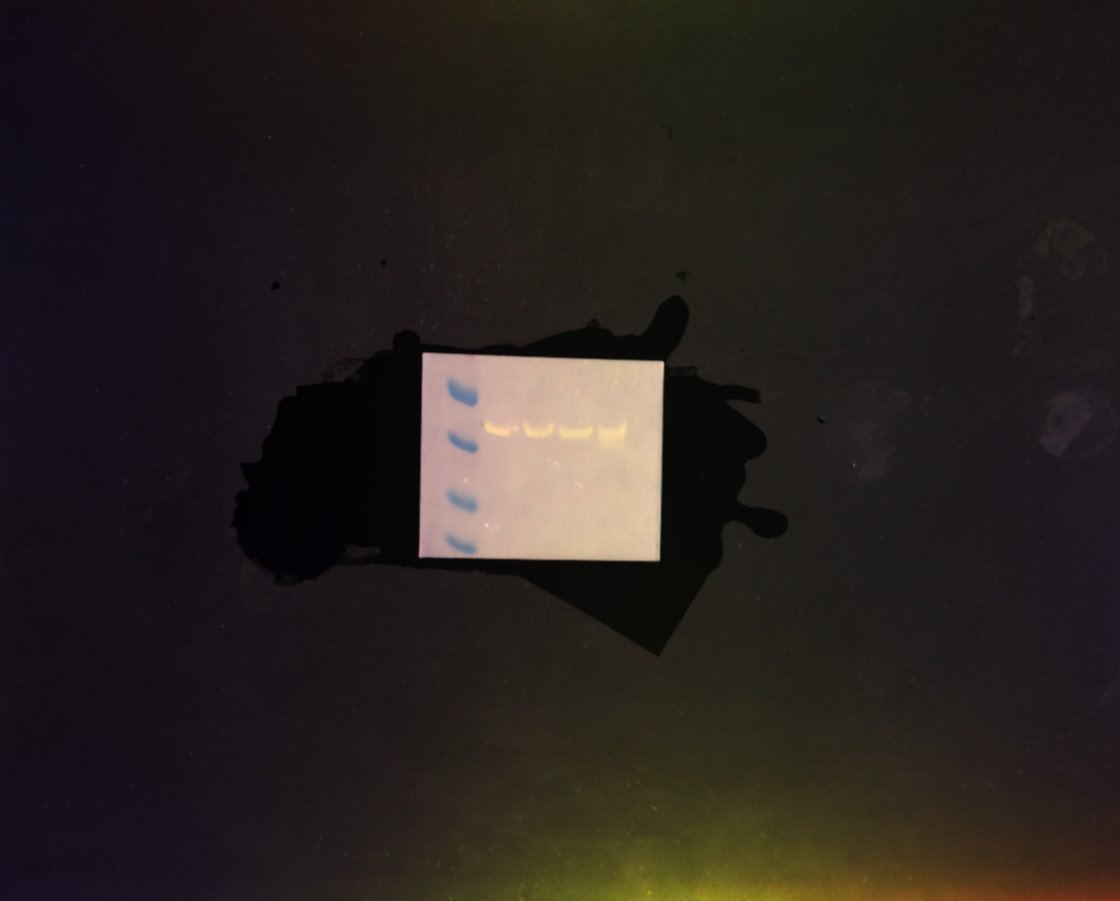

Supplement: Supplementary file 17 — Unprocessed western blots for Fig. 7b. [file 42255_2025_1225_MOESM17_ESM.zip › Zuhra_Unmodified_WesternBlot_Fig7/Experiment1_Normoxia_actin_marker.tif]

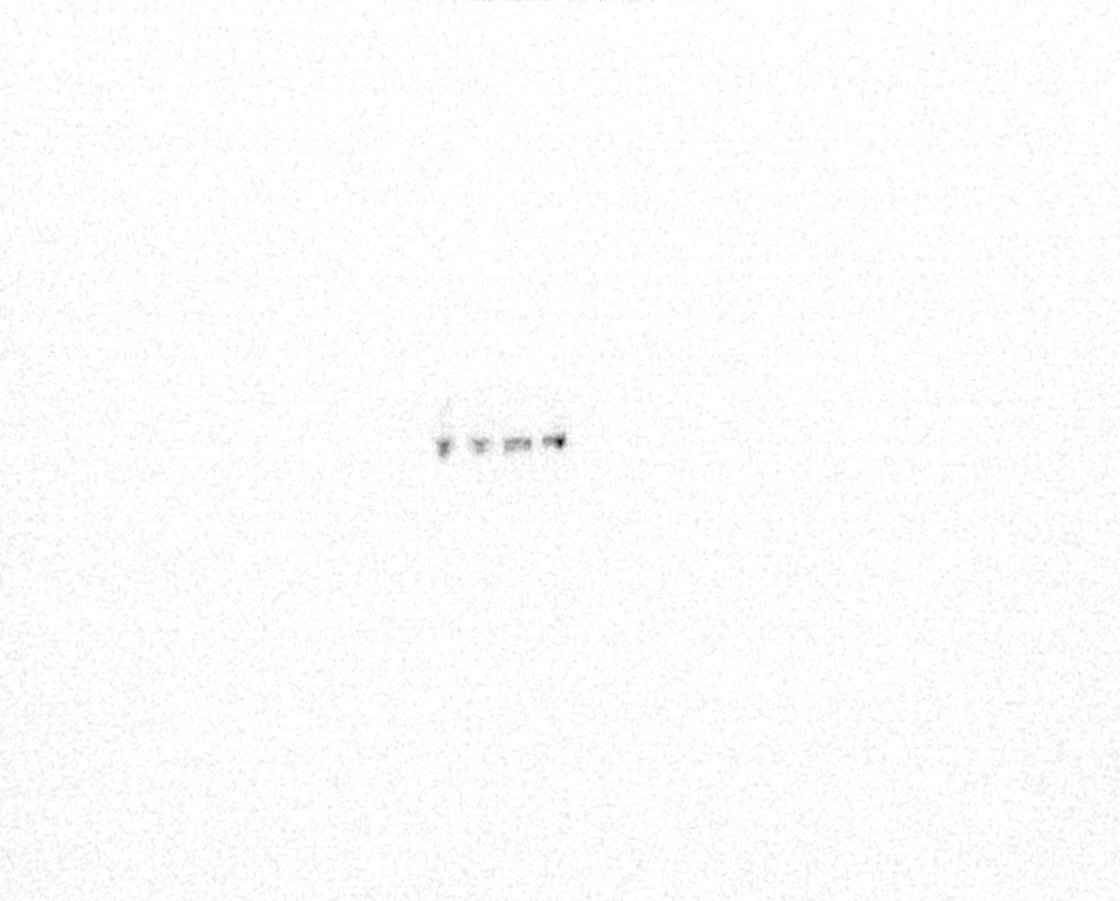

Supplement: Supplementary file 17 — Unprocessed western blots for Fig. 7b. [file 42255_2025_1225_MOESM17_ESM.zip › Zuhra_Unmodified_WesternBlot_Fig7/Experiment1_Normoxia_Hif1a.jpg]

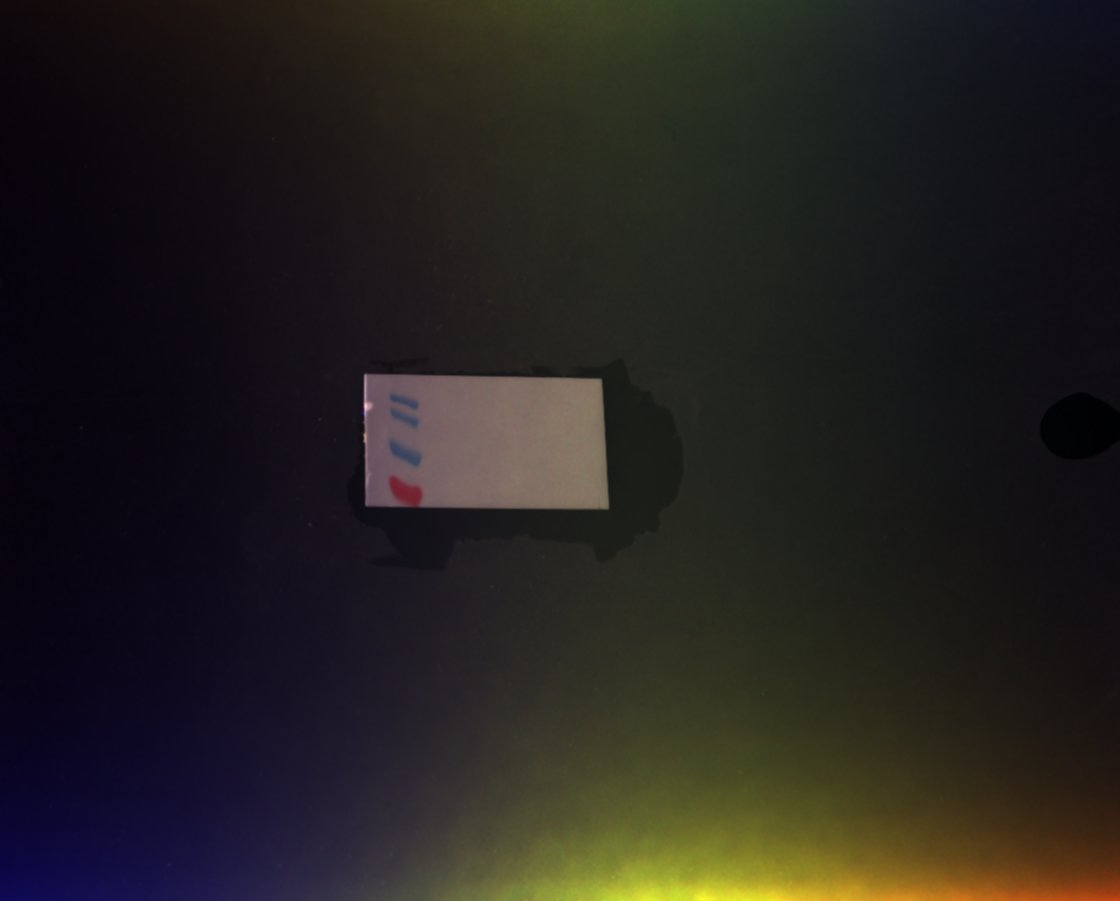

Supplement: Supplementary file 17 — Unprocessed western blots for Fig. 7b. [file 42255_2025_1225_MOESM17_ESM.zip › Zuhra_Unmodified_WesternBlot_Fig7/Experiment1_Normoxia_Hif1a_marker.jpg]

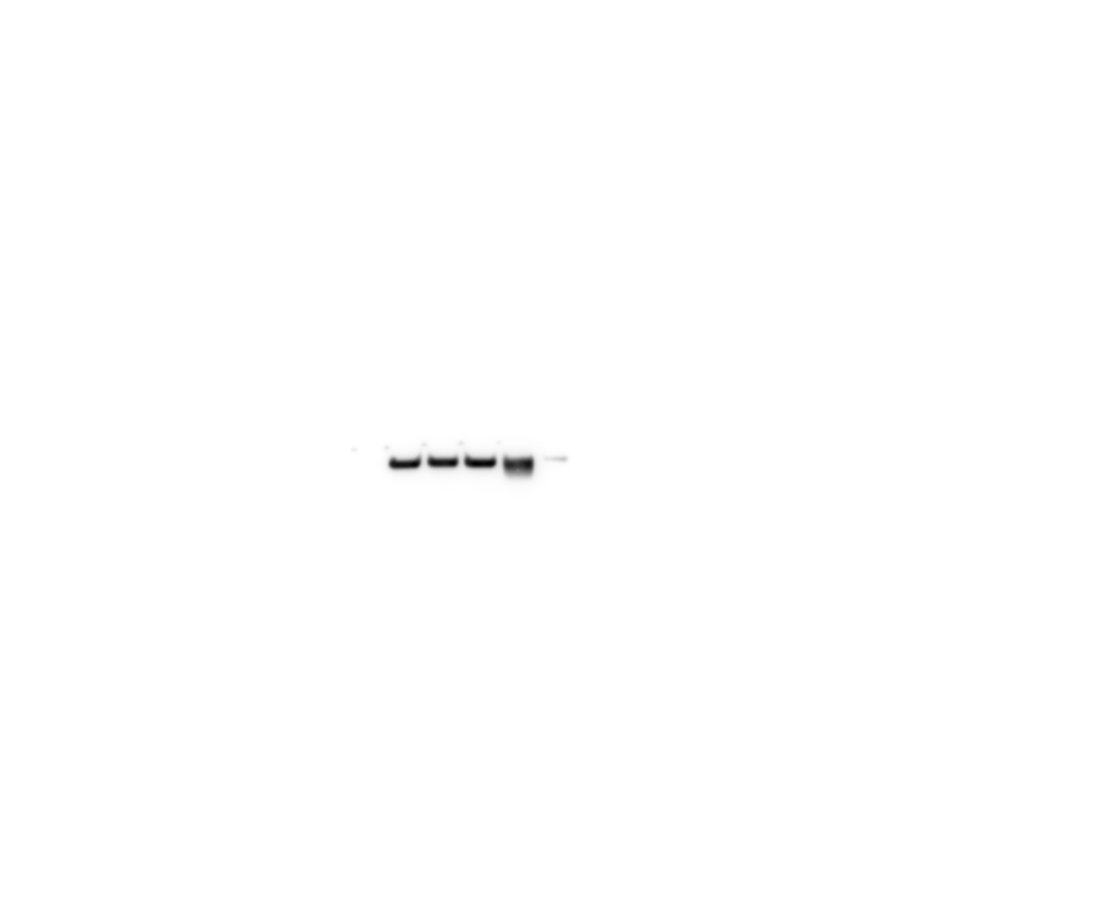

Supplement: Supplementary file 17 — Unprocessed western blots for Fig. 7b. [file 42255_2025_1225_MOESM17_ESM.zip › Zuhra_Unmodified_WesternBlot_Fig7/Experiment2_Hypoxia_actin.jpg]

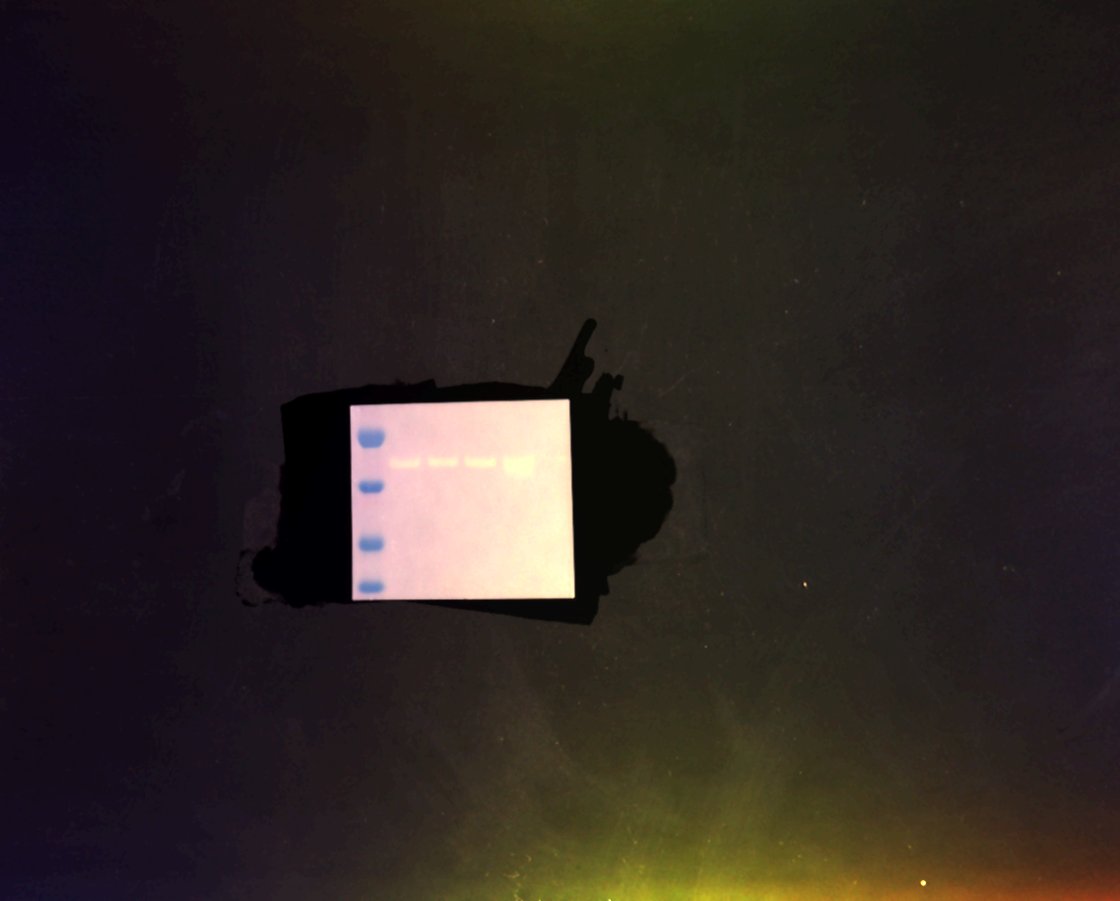

Supplement: Supplementary file 17 — Unprocessed western blots for Fig. 7b. [file 42255_2025_1225_MOESM17_ESM.zip › Zuhra_Unmodified_WesternBlot_Fig7/Experiment2_Hypoxia_actin_marker.jpg]

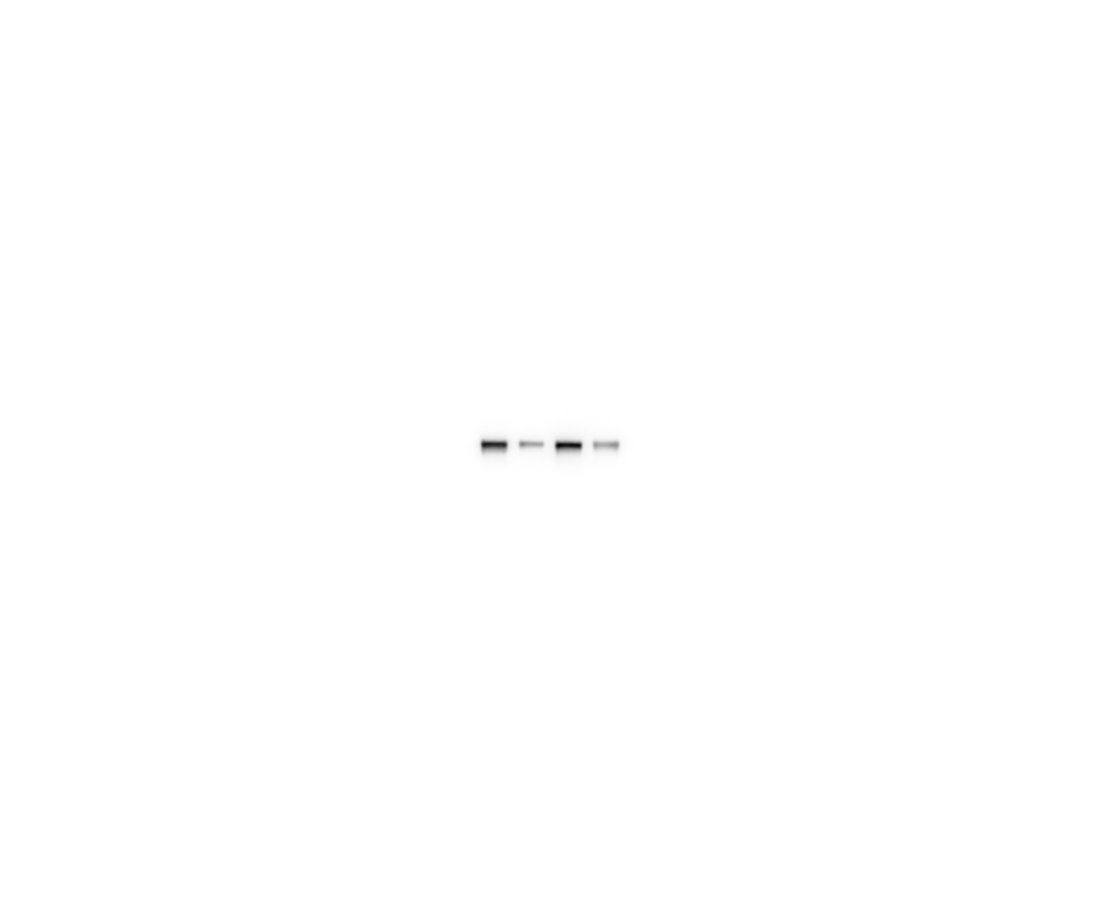

Supplement: Supplementary file 17 — Unprocessed western blots for Fig. 7b. [file 42255_2025_1225_MOESM17_ESM.zip › Zuhra_Unmodified_WesternBlot_Fig7/Experiment2_Hypoxia_Hif1a.jpg]

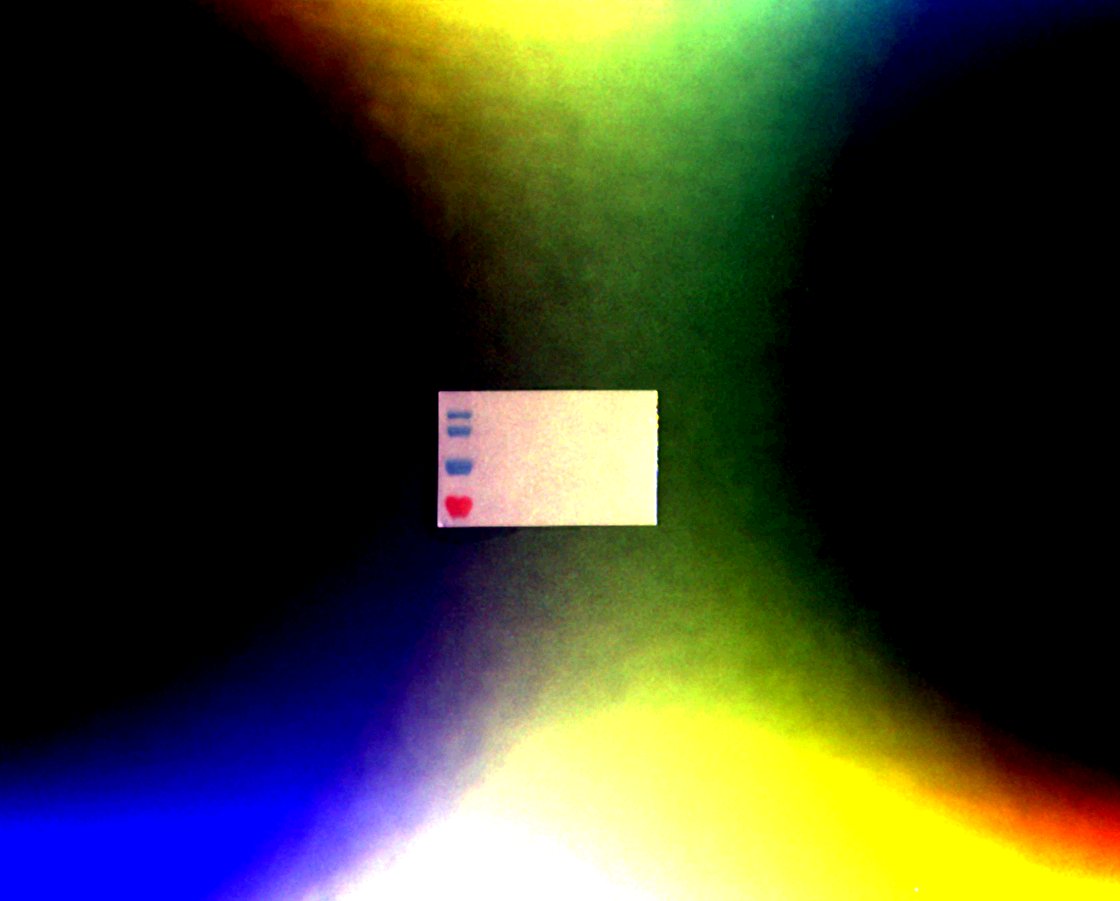

Supplement: Supplementary file 17 — Unprocessed western blots for Fig. 7b. [file 42255_2025_1225_MOESM17_ESM.zip › Zuhra_Unmodified_WesternBlot_Fig7/Experiment2_Hypoxia_Hif1a_marker.jpg]

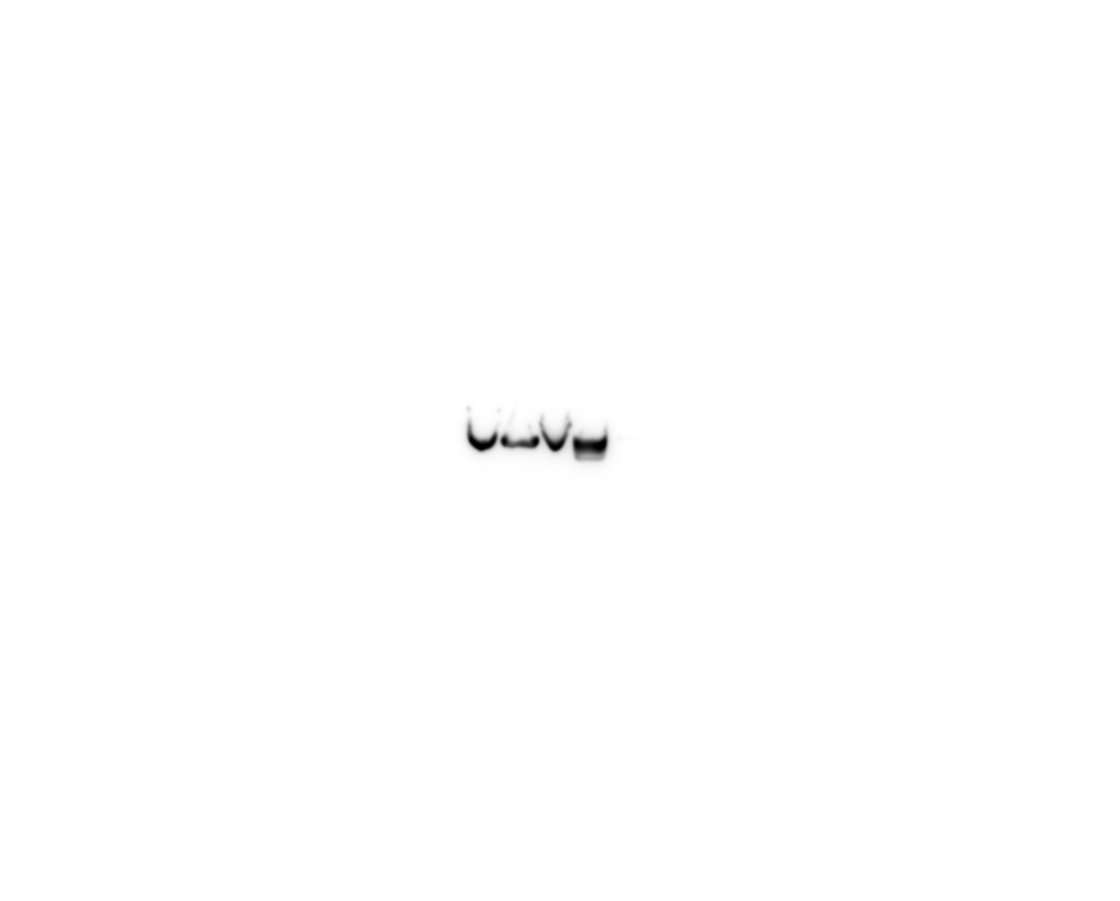

Supplement: Supplementary file 17 — Unprocessed western blots for Fig. 7b. [file 42255_2025_1225_MOESM17_ESM.zip › Zuhra_Unmodified_WesternBlot_Fig7/Experiment2_Normoxia_actin.jpg]

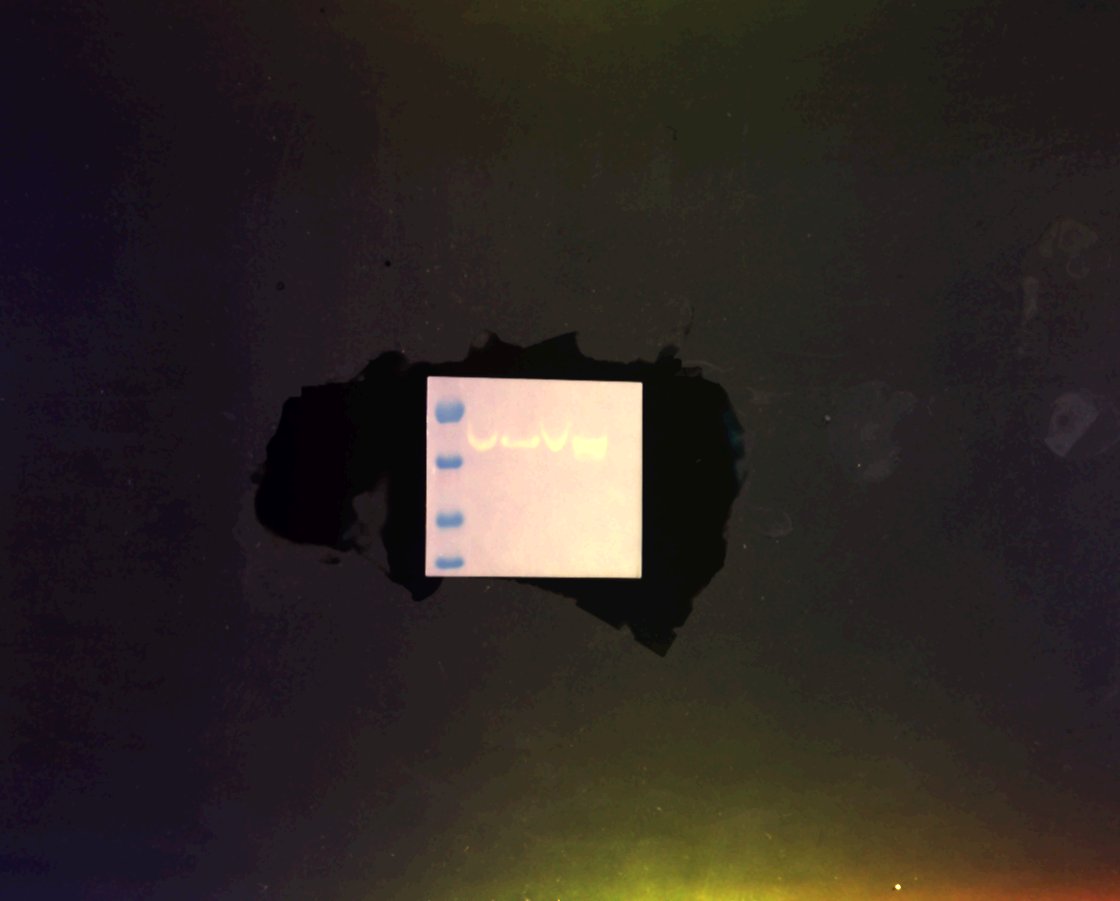

Supplement: Supplementary file 17 — Unprocessed western blots for Fig. 7b. [file 42255_2025_1225_MOESM17_ESM.zip › Zuhra_Unmodified_WesternBlot_Fig7/Experiment2_Normoxia_actin_marker.jpg]

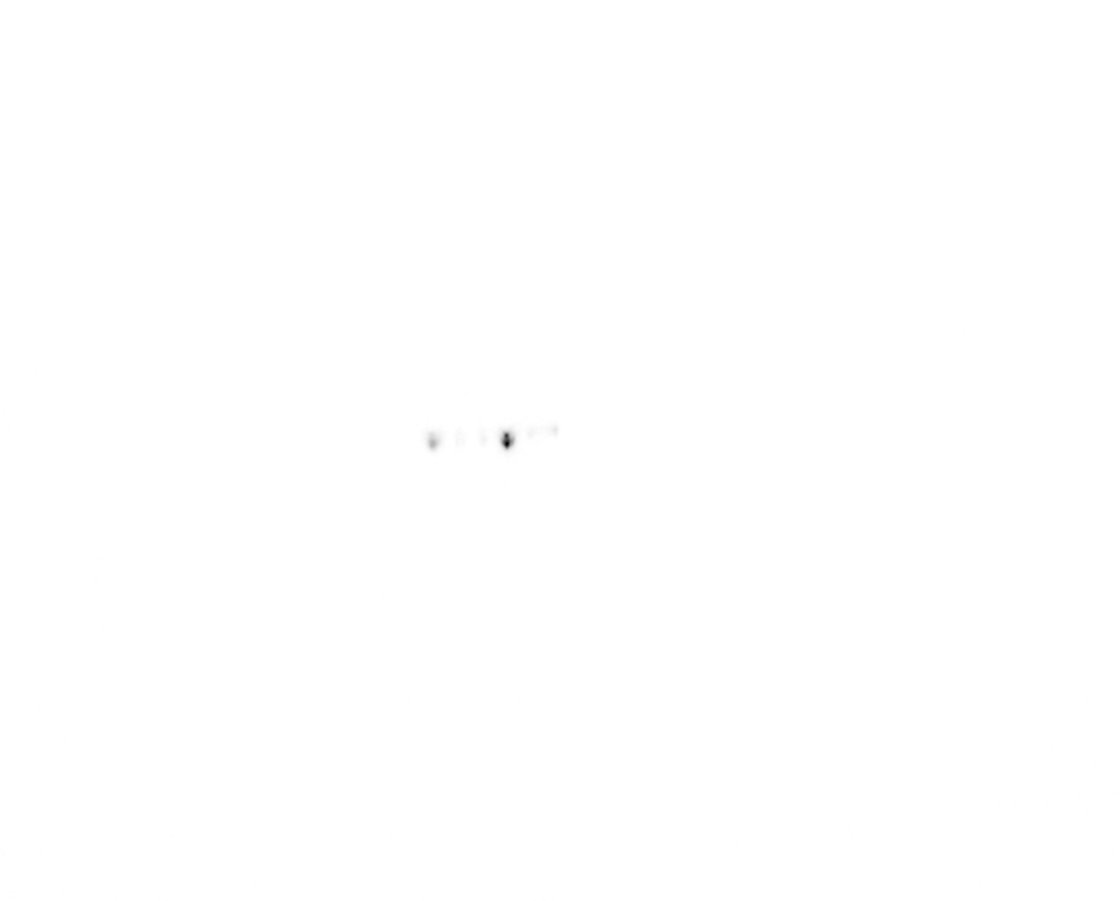

Supplement: Supplementary file 17 — Unprocessed western blots for Fig. 7b. [file 42255_2025_1225_MOESM17_ESM.zip › Zuhra_Unmodified_WesternBlot_Fig7/Experiment2_Normoxia_Hif1a.jpg]

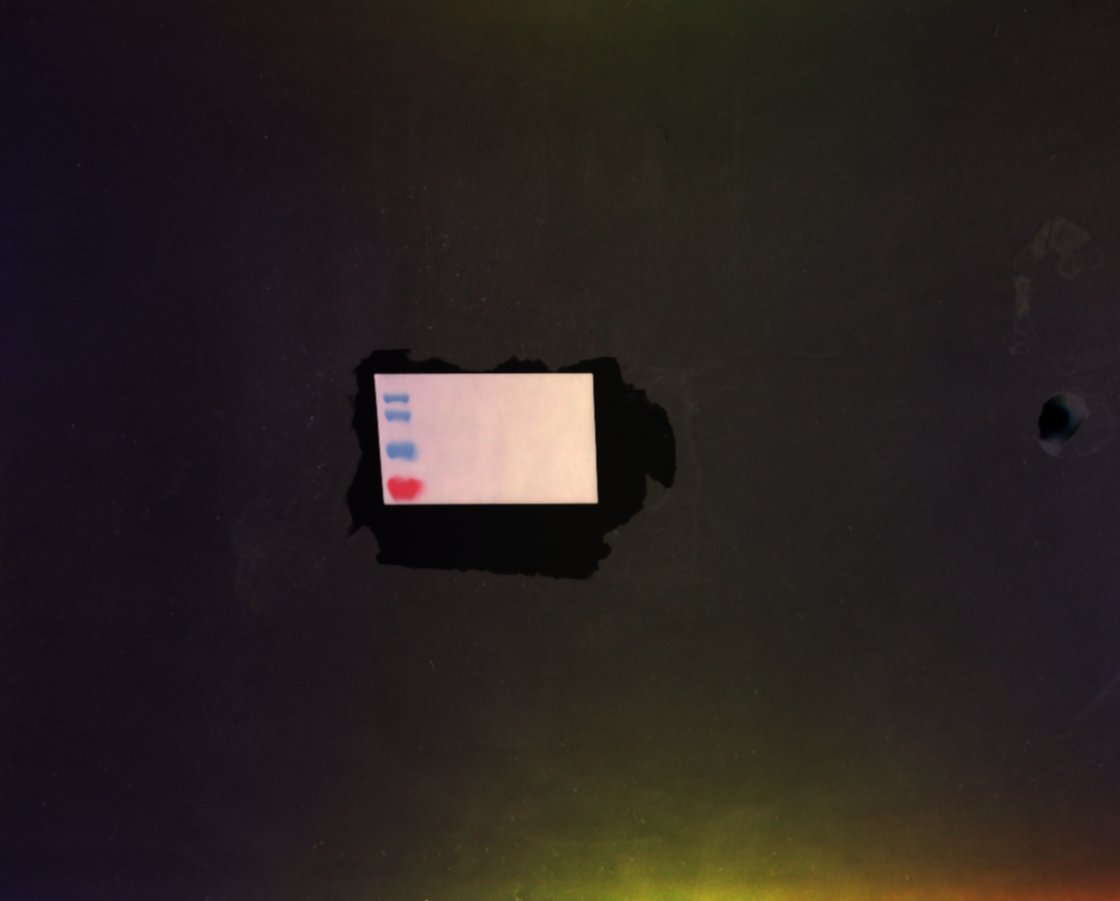

Supplement: Supplementary file 17 — Unprocessed western blots for Fig. 7b. [file 42255_2025_1225_MOESM17_ESM.zip › Zuhra_Unmodified_WesternBlot_Fig7/Experiment2_Normoxia_Hif1a_marker.jpg]

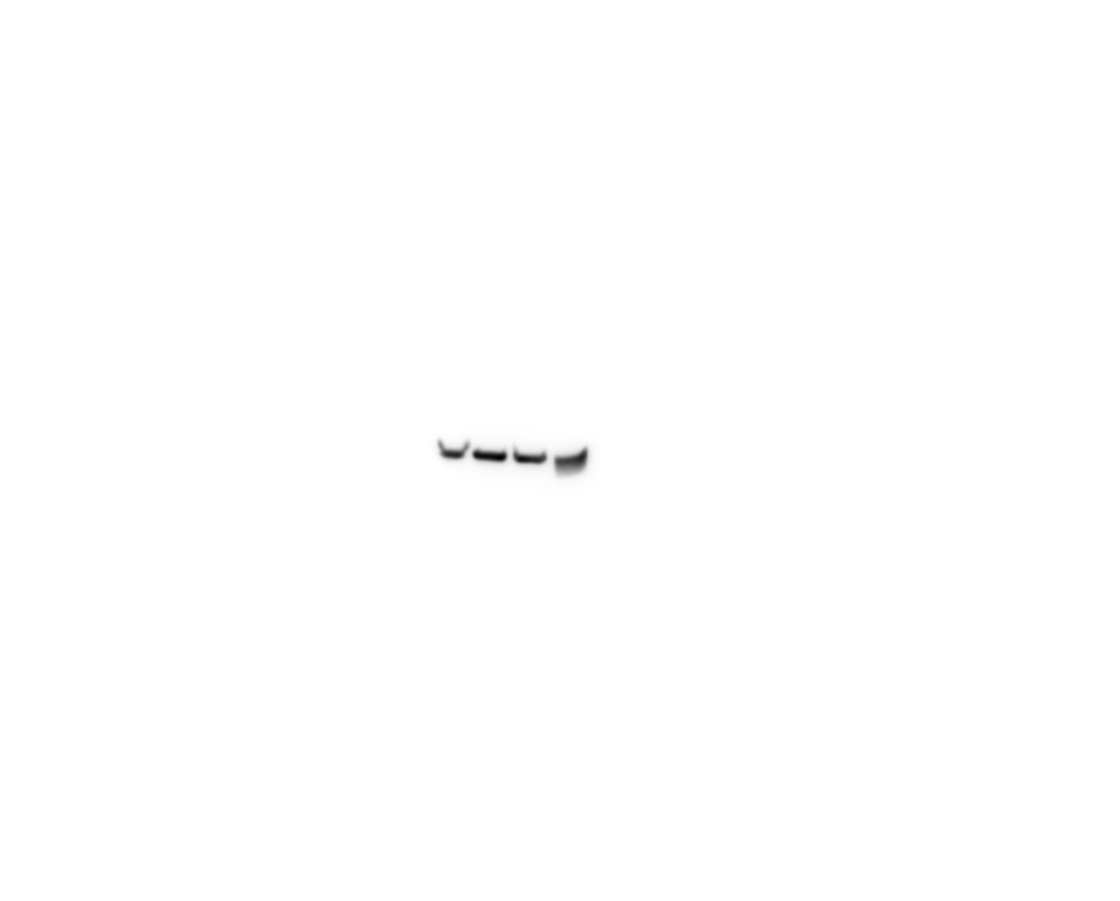

Supplement: Supplementary file 17 — Unprocessed western blots for Fig. 7b. [file 42255_2025_1225_MOESM17_ESM.zip › Zuhra_Unmodified_WesternBlot_Fig7/Experiment3_Hypoxia_actin.jpg]

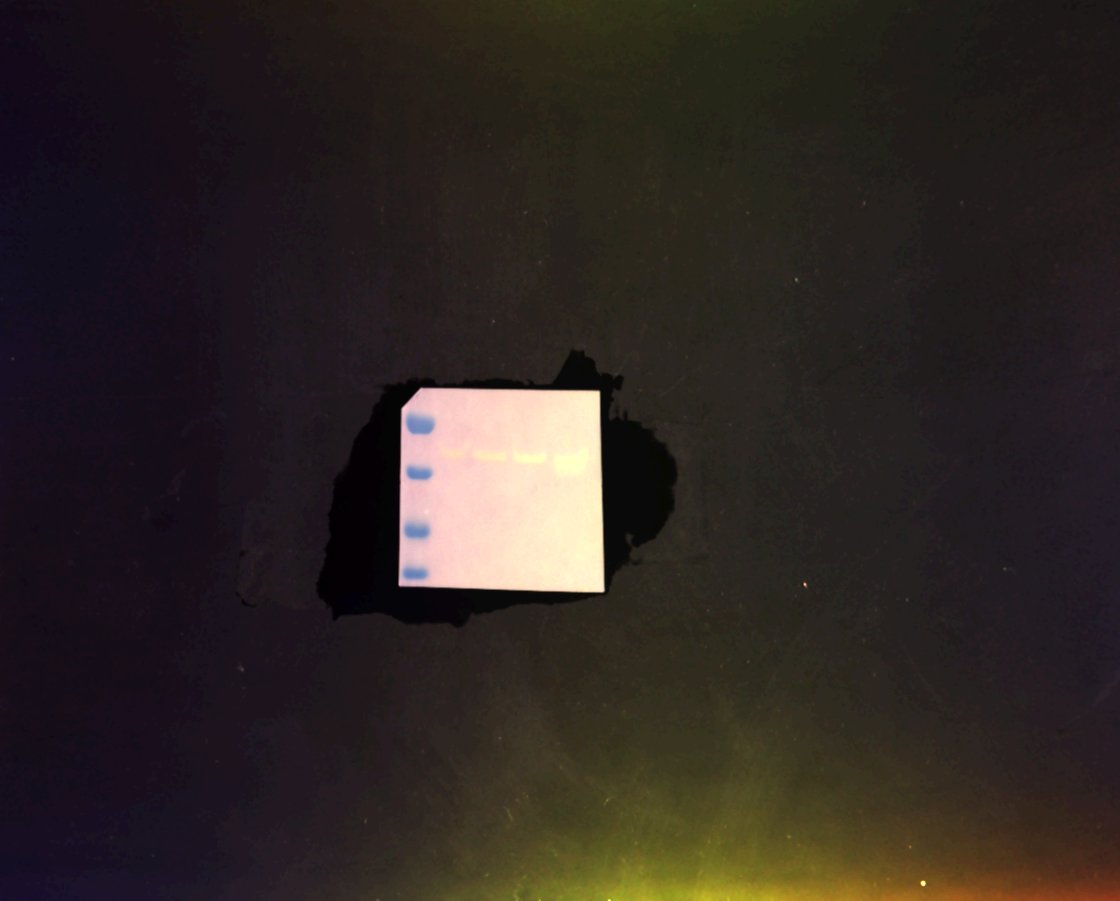

Supplement: Supplementary file 17 — Unprocessed western blots for Fig. 7b. [file 42255_2025_1225_MOESM17_ESM.zip › Zuhra_Unmodified_WesternBlot_Fig7/Experiment3_Hypoxia_actin_marker.jpg]

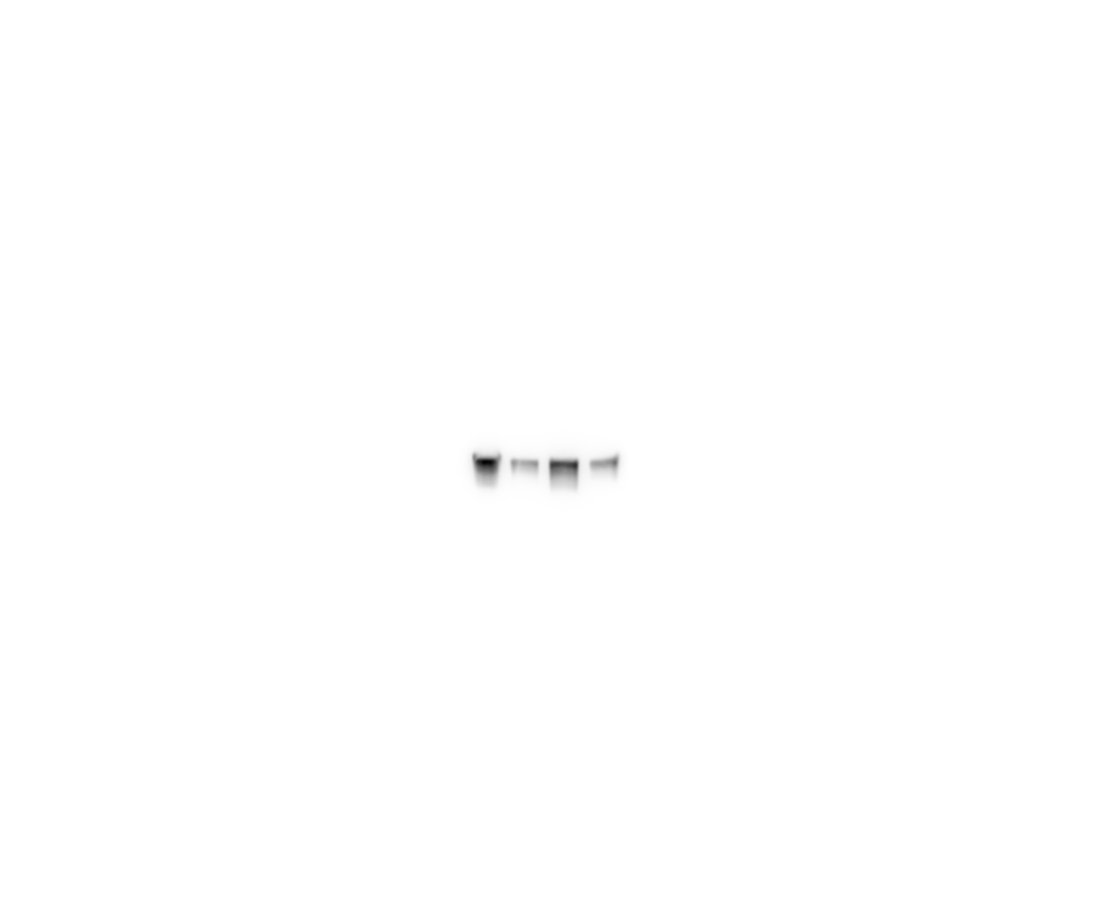

Supplement: Supplementary file 17 — Unprocessed western blots for Fig. 7b. [file 42255_2025_1225_MOESM17_ESM.zip › Zuhra_Unmodified_WesternBlot_Fig7/Experiment3_Hypoxia_Hif1a.jpg]

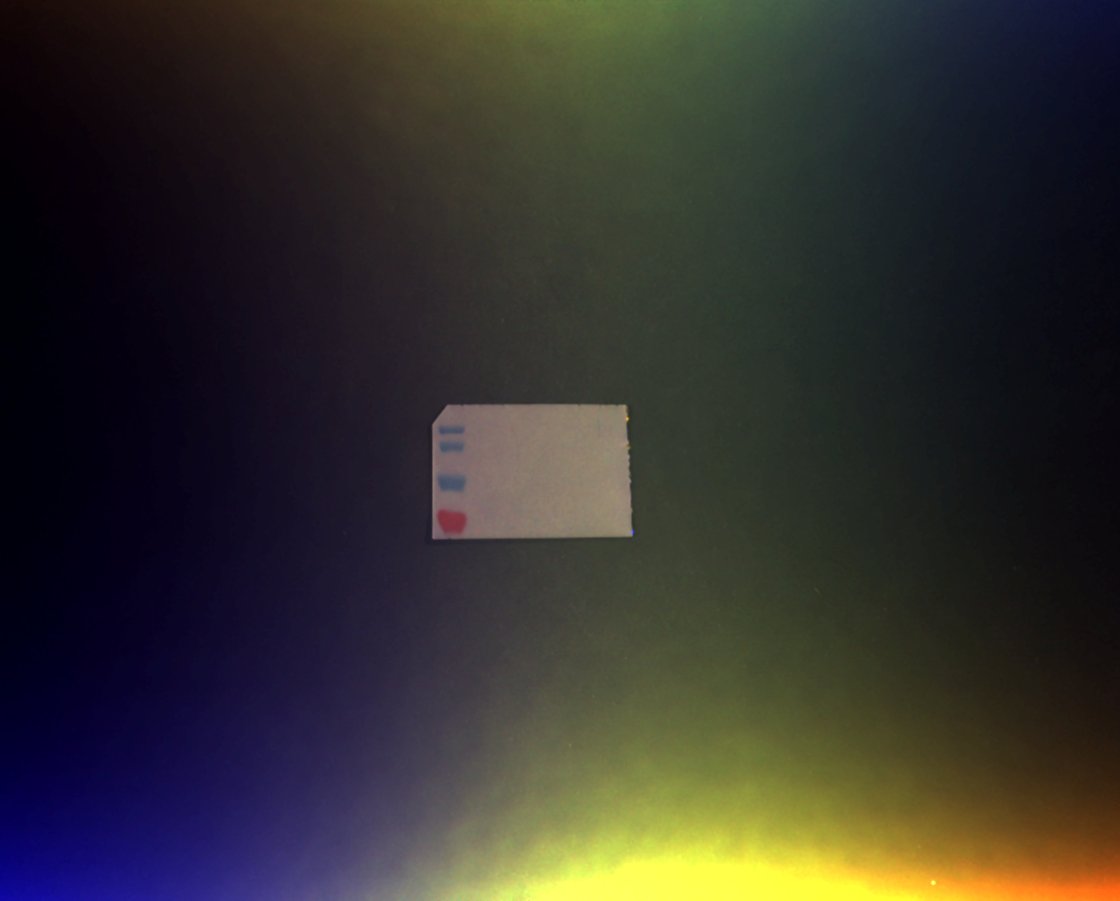

Supplement: Supplementary file 17 — Unprocessed western blots for Fig. 7b. [file 42255_2025_1225_MOESM17_ESM.zip › Zuhra_Unmodified_WesternBlot_Fig7/Experiment3_Hypoxia_Hif1a_marker.jpg]

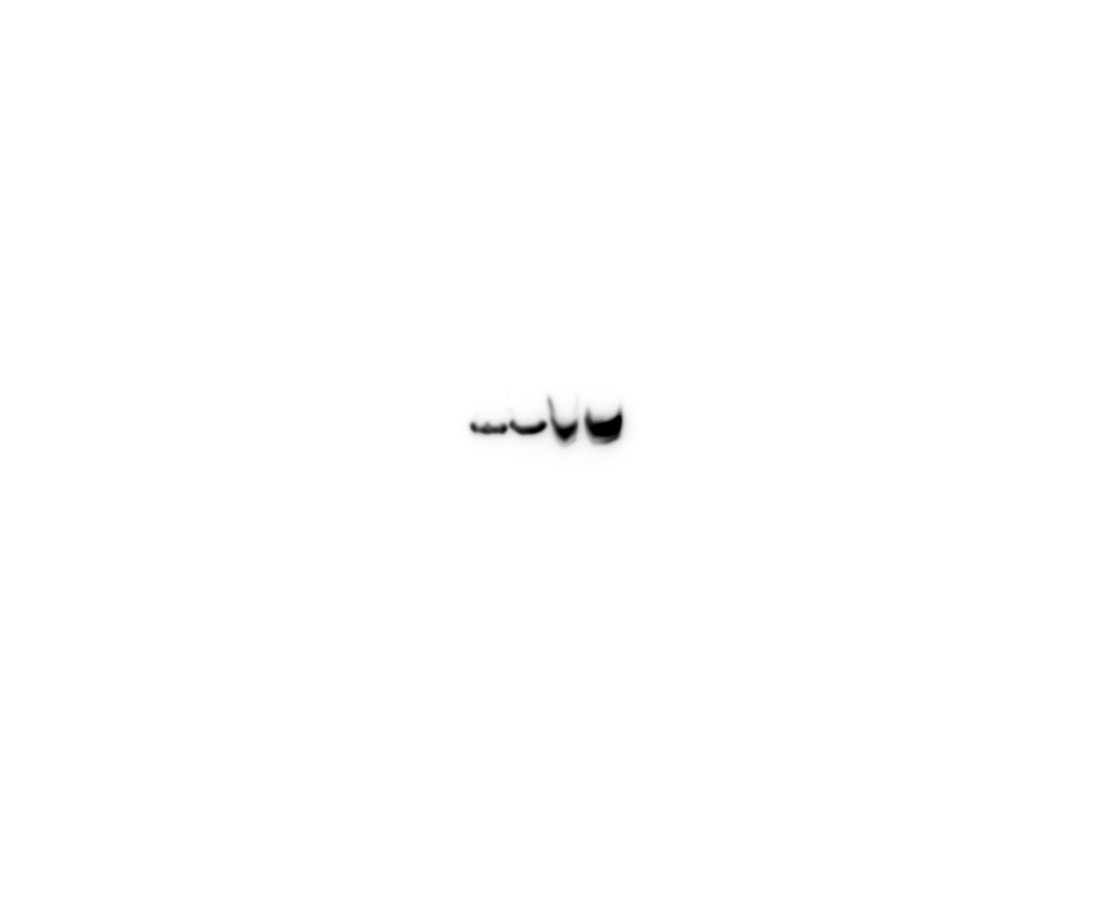

Supplement: Supplementary file 17 — Unprocessed western blots for Fig. 7b. [file 42255_2025_1225_MOESM17_ESM.zip › Zuhra_Unmodified_WesternBlot_Fig7/Experiment3_Normoxia_actin.jpg]

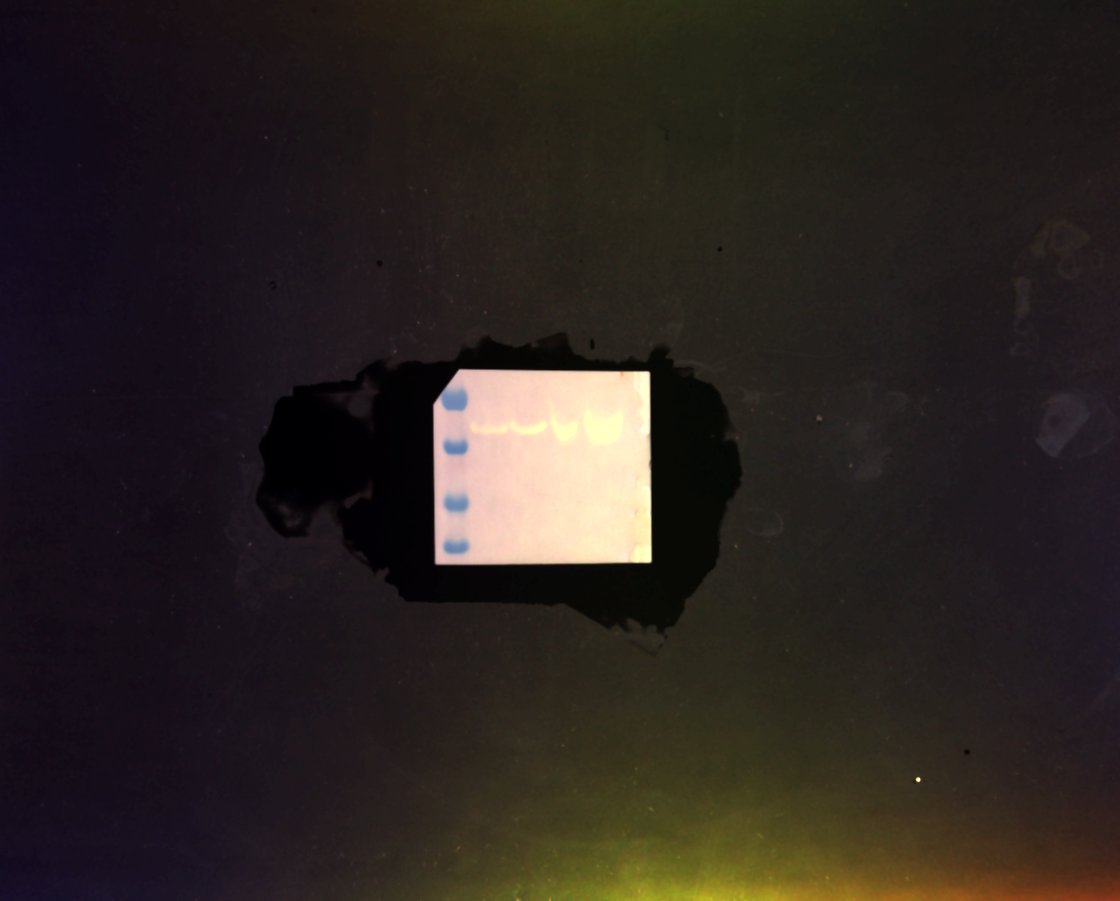

Supplement: Supplementary file 17 — Unprocessed western blots for Fig. 7b. [file 42255_2025_1225_MOESM17_ESM.zip › Zuhra_Unmodified_WesternBlot_Fig7/Experiment3_Normoxia_actin_marker.jpg]

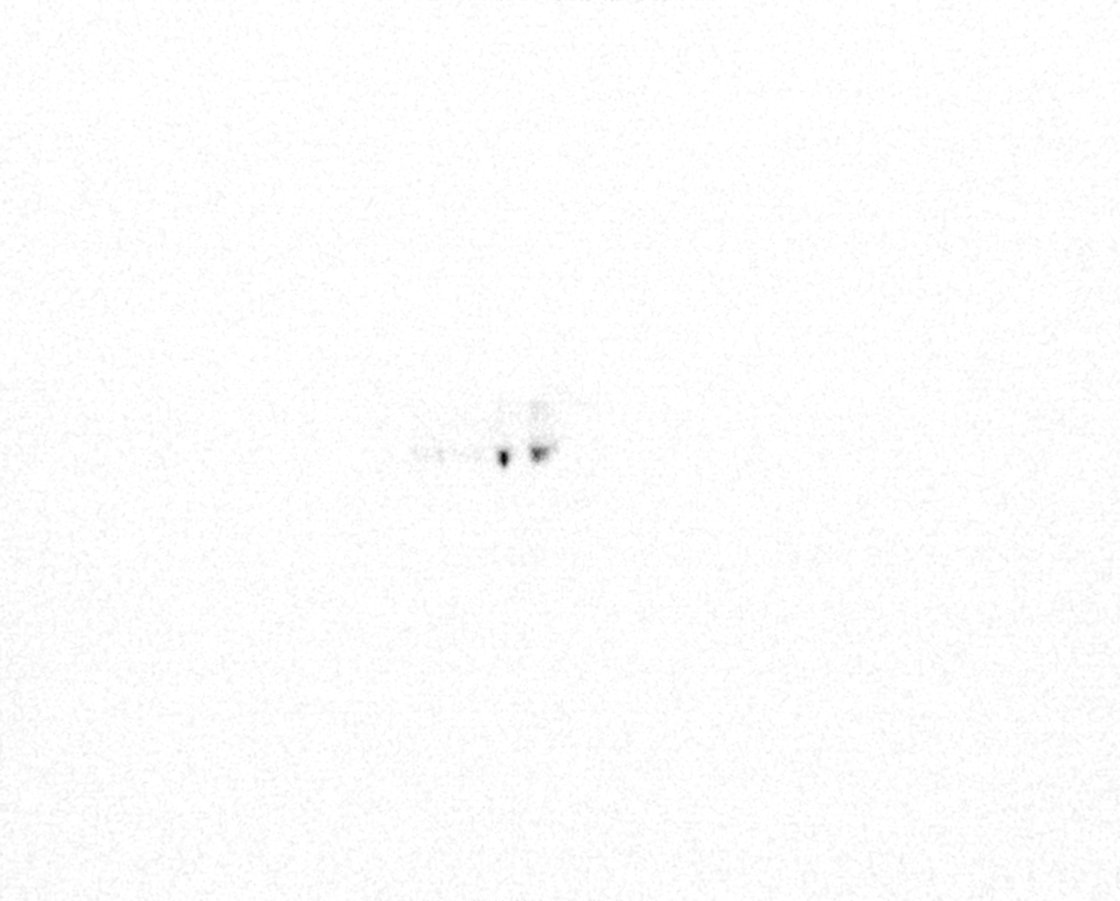

Supplement: Supplementary file 17 — Unprocessed western blots for Fig. 7b. [file 42255_2025_1225_MOESM17_ESM.zip › Zuhra_Unmodified_WesternBlot_Fig7/Experiment3_Normoxia_Hif1a.jpg]

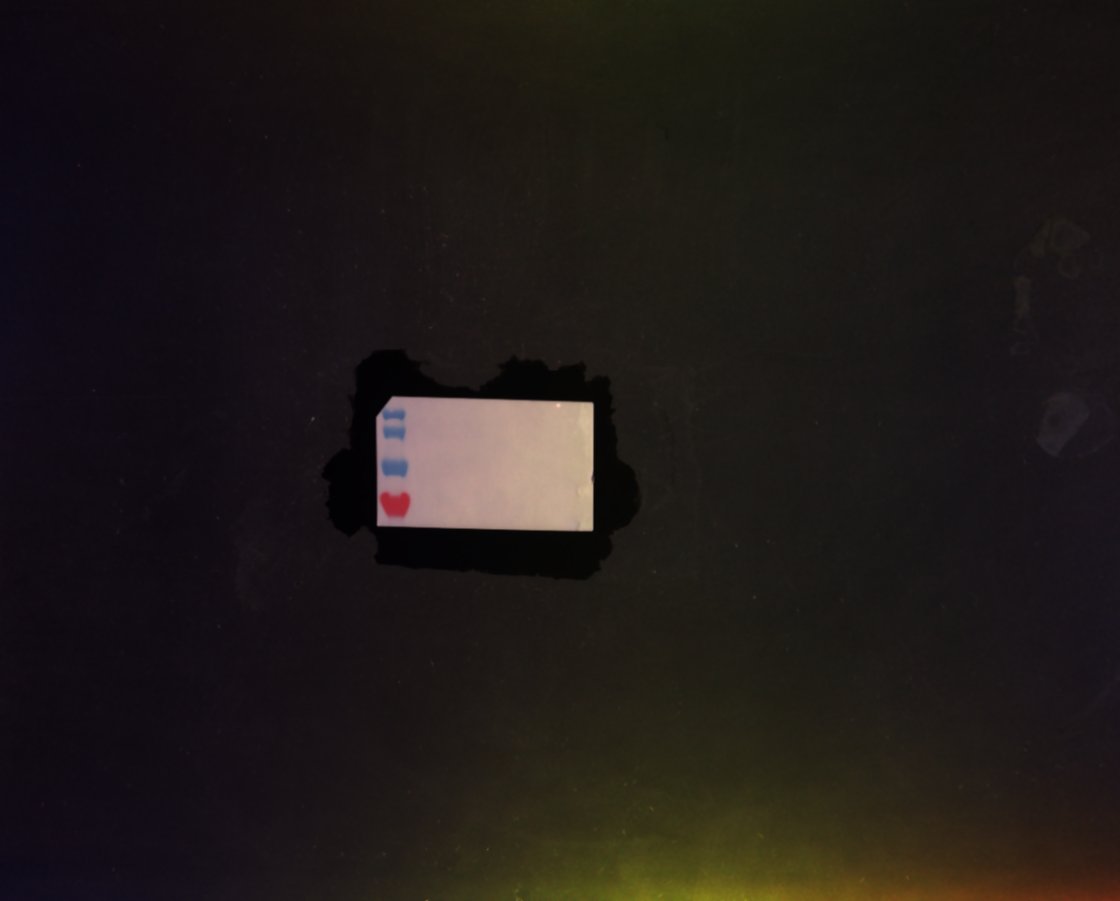

Supplement: Supplementary file 17 — Unprocessed western blots for Fig. 7b. [file 42255_2025_1225_MOESM17_ESM.zip › Zuhra_Unmodified_WesternBlot_Fig7/Experiment3_Normoxia_Hif1a_marker.jpg]

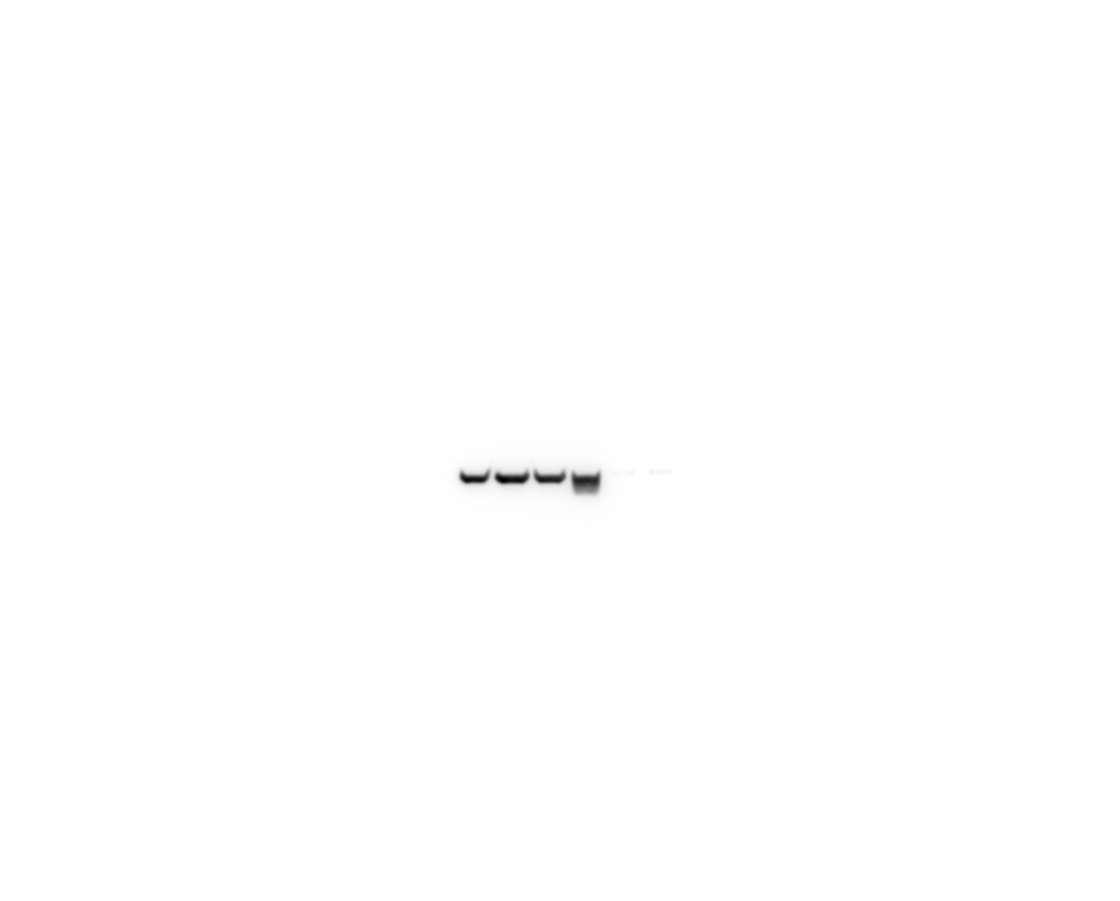

Supplement: Supplementary file 17 — Unprocessed western blots for Fig. 7b. [file 42255_2025_1225_MOESM17_ESM.zip › Zuhra_Unmodified_WesternBlot_Fig7/Experiment4_Hypoxia_actin.jpg]

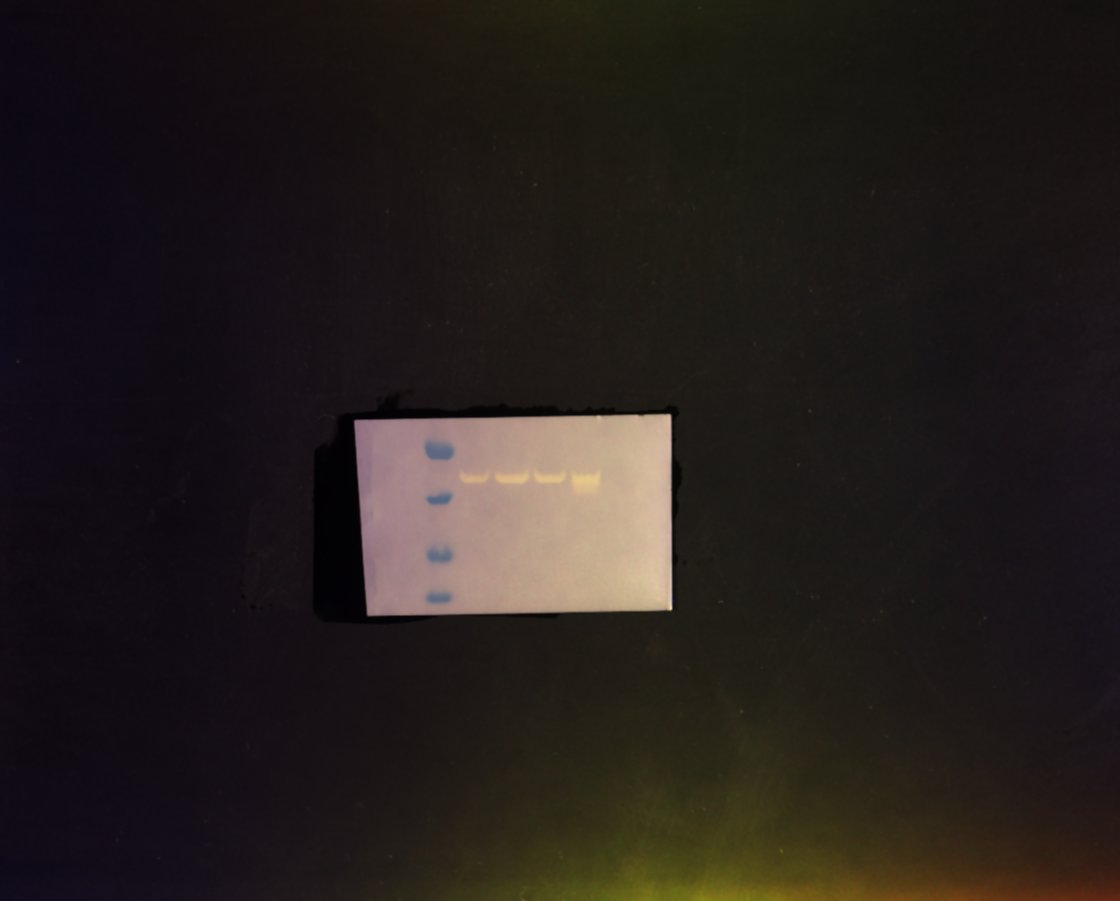

Supplement: Supplementary file 17 — Unprocessed western blots for Fig. 7b. [file 42255_2025_1225_MOESM17_ESM.zip › Zuhra_Unmodified_WesternBlot_Fig7/Experiment4_Hypoxia_actin_marker.jpg]

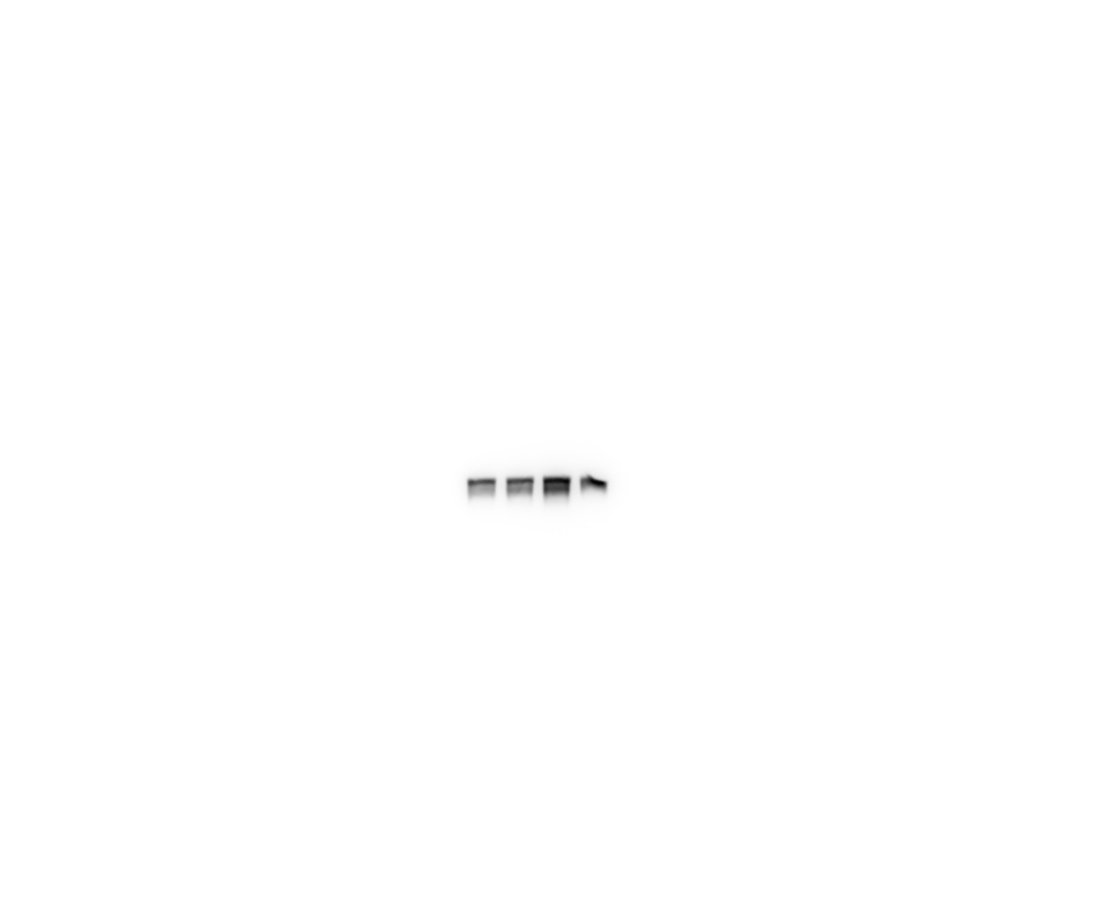

Supplement: Supplementary file 17 — Unprocessed western blots for Fig. 7b. [file 42255_2025_1225_MOESM17_ESM.zip › Zuhra_Unmodified_WesternBlot_Fig7/Experiment4_Hypoxia_Hif1a.jpg]

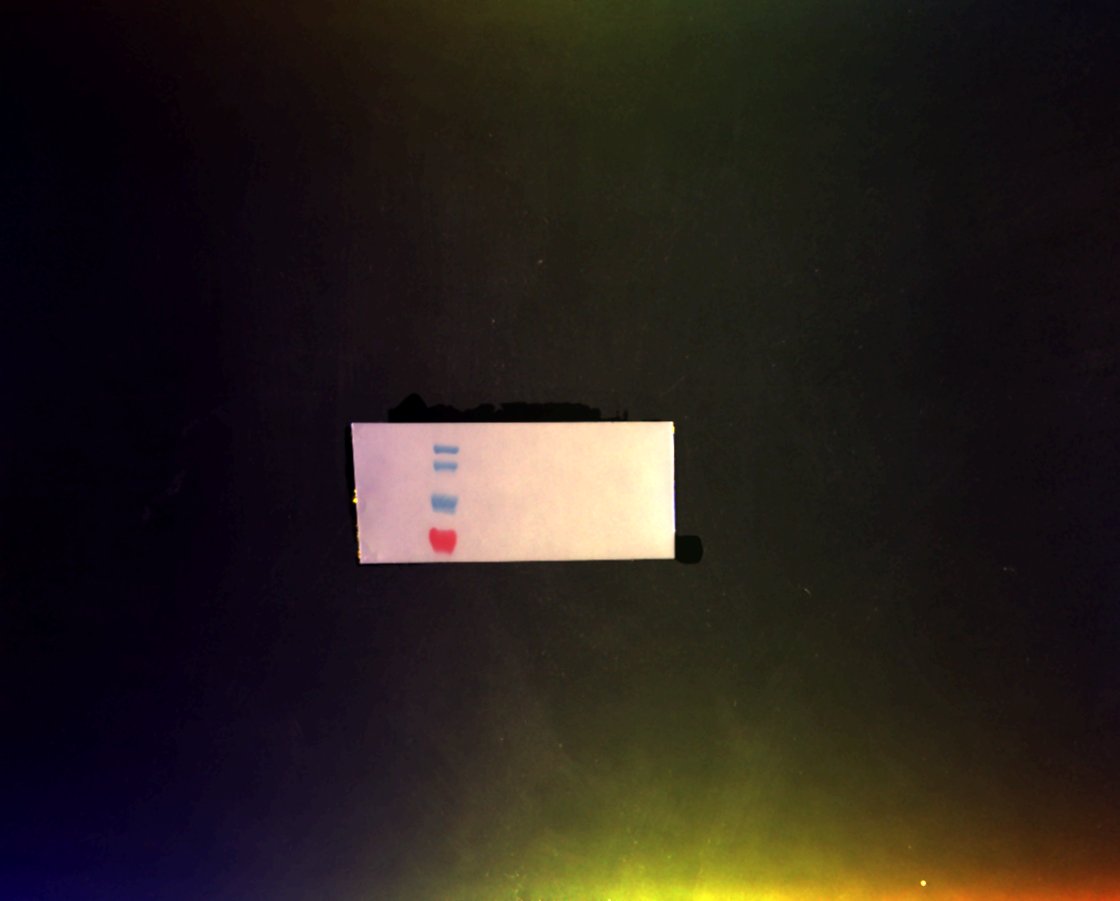

Supplement: Supplementary file 17 — Unprocessed western blots for Fig. 7b. [file 42255_2025_1225_MOESM17_ESM.zip › Zuhra_Unmodified_WesternBlot_Fig7/Experiment4_Hypoxia_Hif1a_marker.jpg]

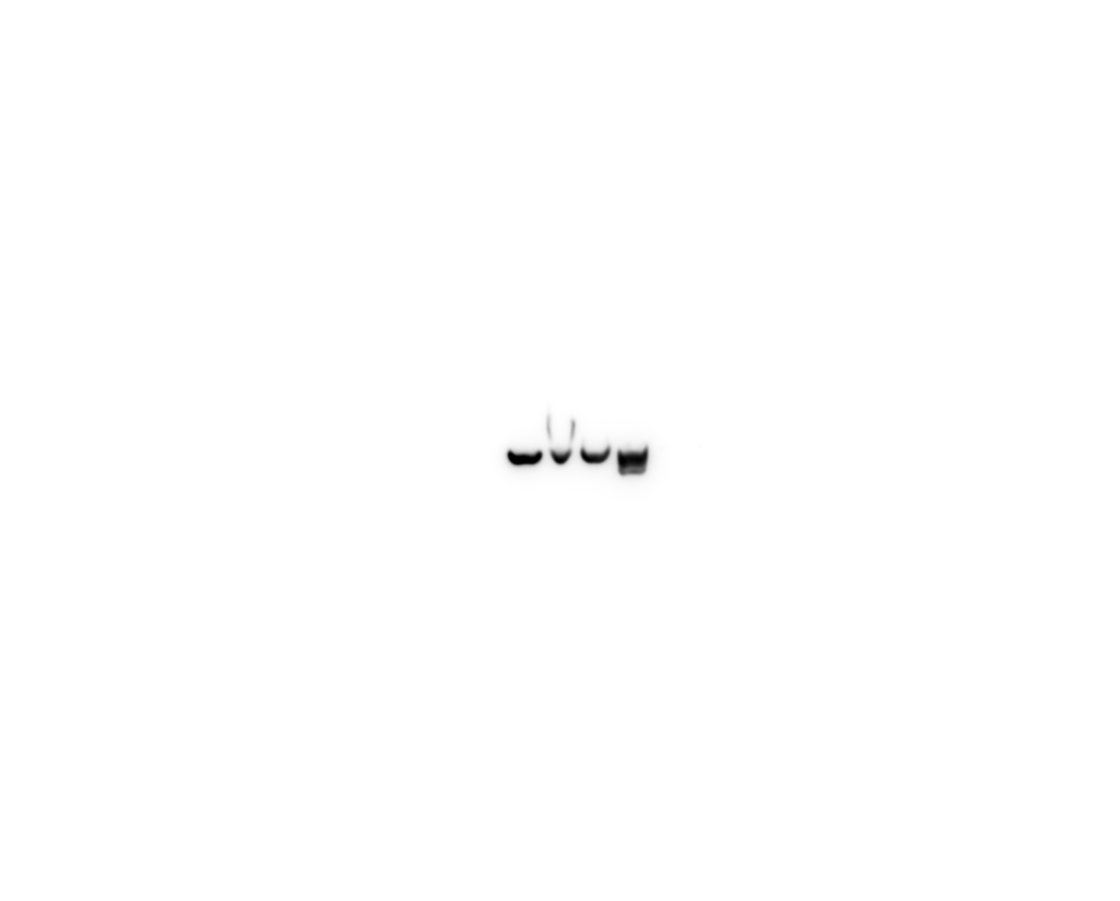

Supplement: Supplementary file 17 — Unprocessed western blots for Fig. 7b. [file 42255_2025_1225_MOESM17_ESM.zip › Zuhra_Unmodified_WesternBlot_Fig7/Experiment4_Normoxia_actin.jpg]

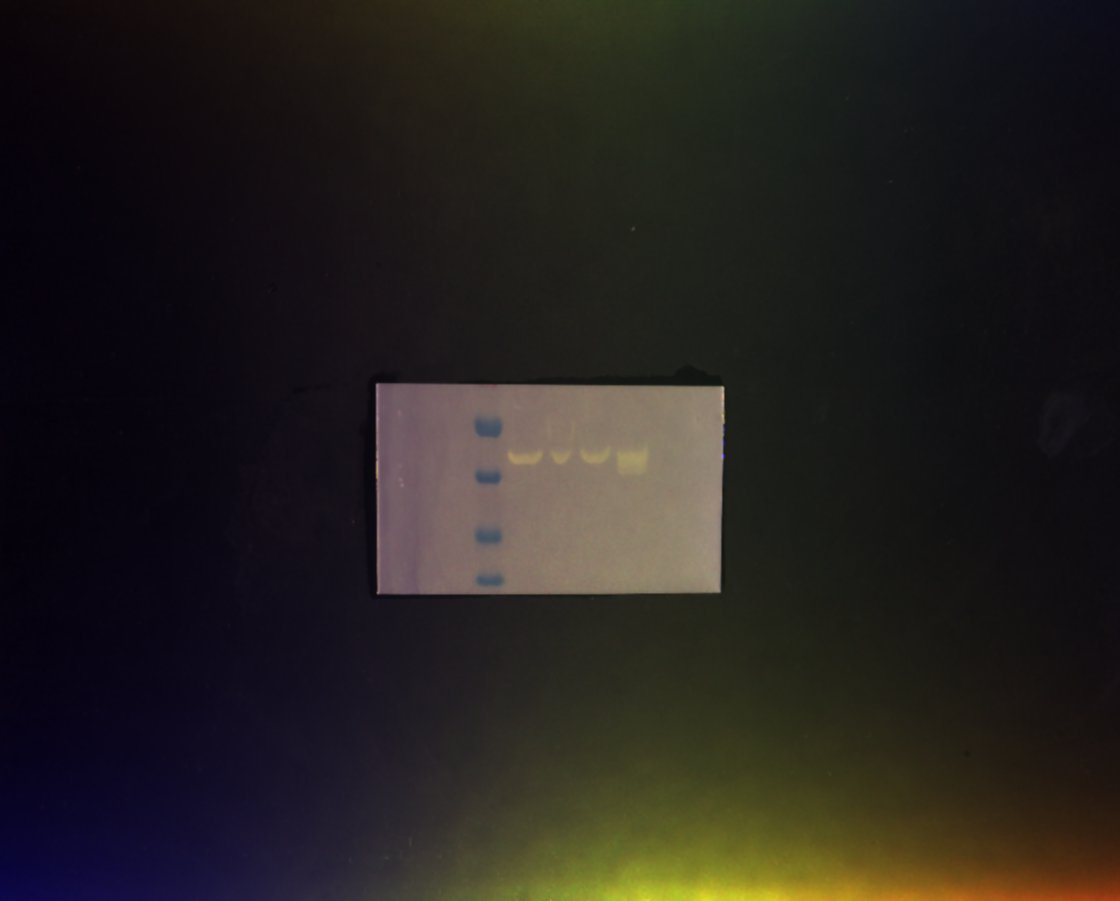

Supplement: Supplementary file 17 — Unprocessed western blots for Fig. 7b. [file 42255_2025_1225_MOESM17_ESM.zip › Zuhra_Unmodified_WesternBlot_Fig7/Experiment4_Normoxia_actin_marker.jpg]

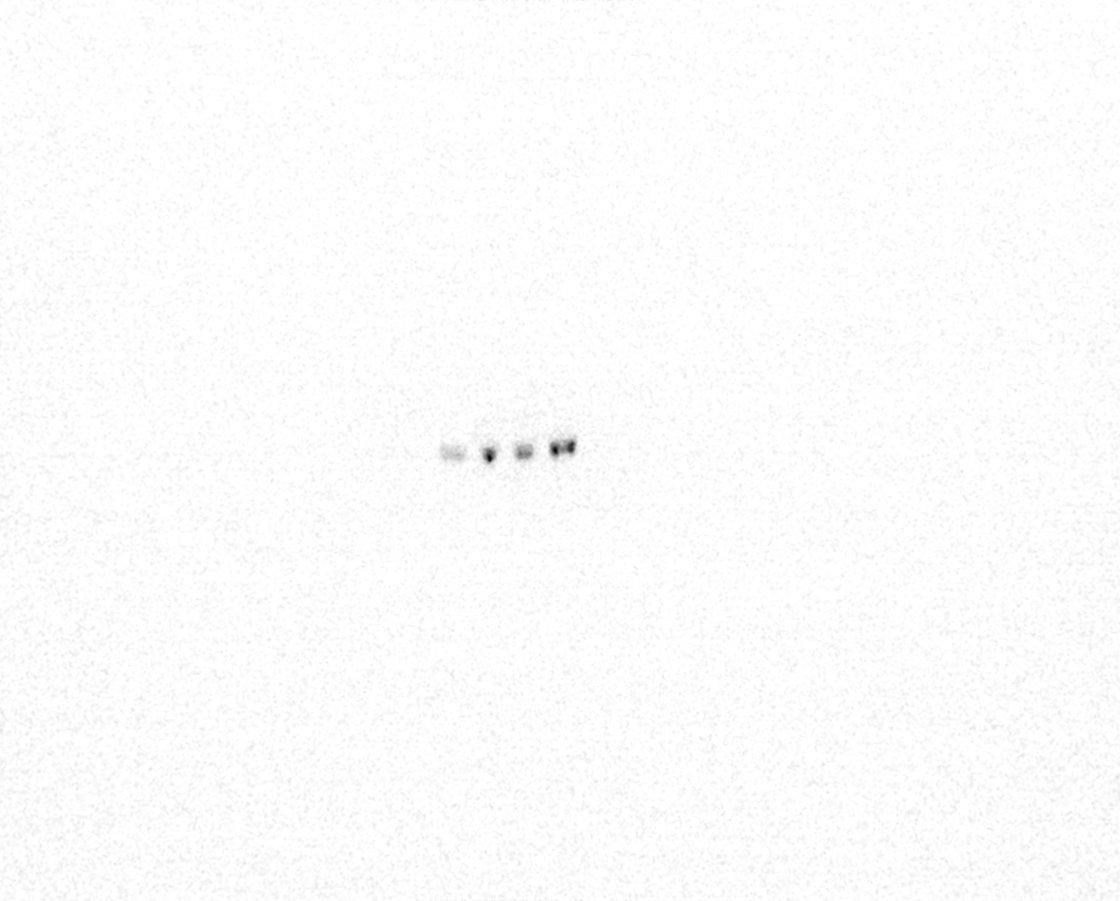

Supplement: Supplementary file 17 — Unprocessed western blots for Fig. 7b. [file 42255_2025_1225_MOESM17_ESM.zip › Zuhra_Unmodified_WesternBlot_Fig7/Experiment4_Normoxia_Hif1a.jpg]

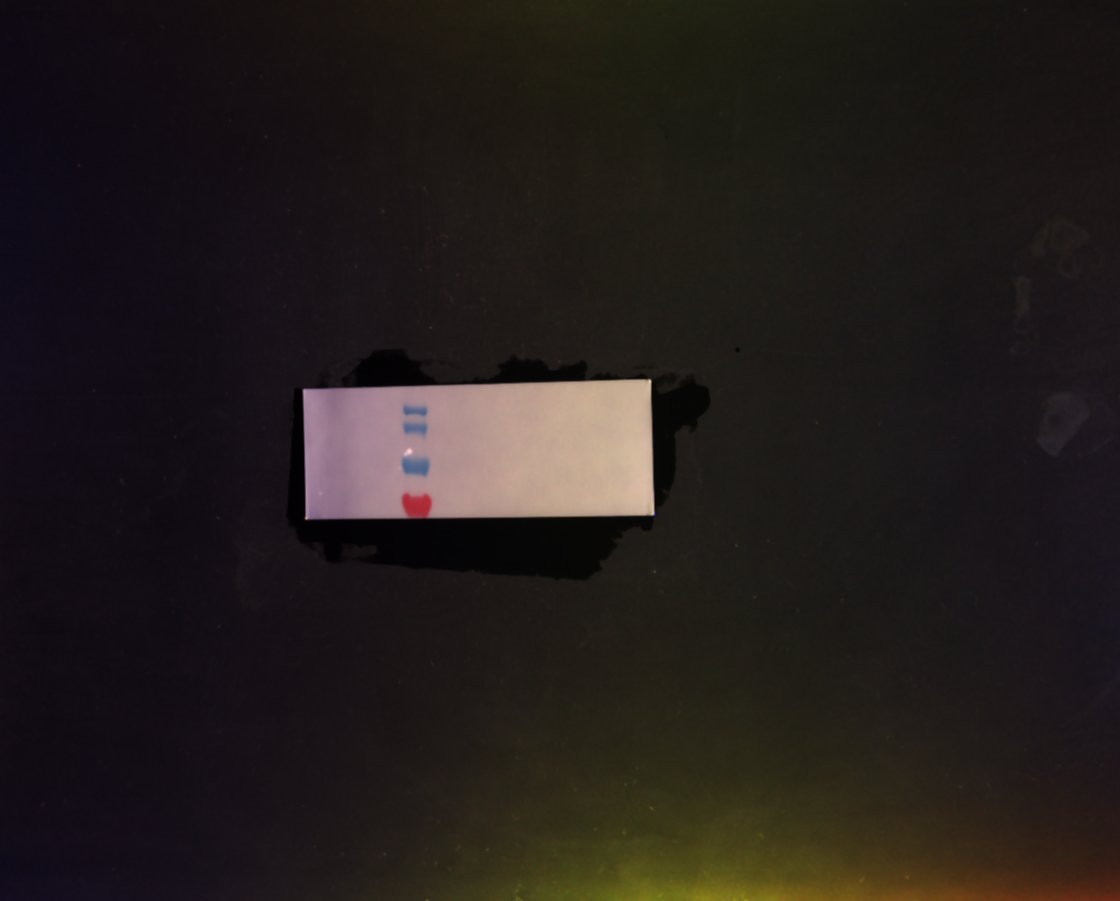

Supplement: Supplementary file 17 — Unprocessed western blots for Fig. 7b. [file 42255_2025_1225_MOESM17_ESM.zip › Zuhra_Unmodified_WesternBlot_Fig7/Experiment4_Normoxia_Hif1a_marker.jpg]

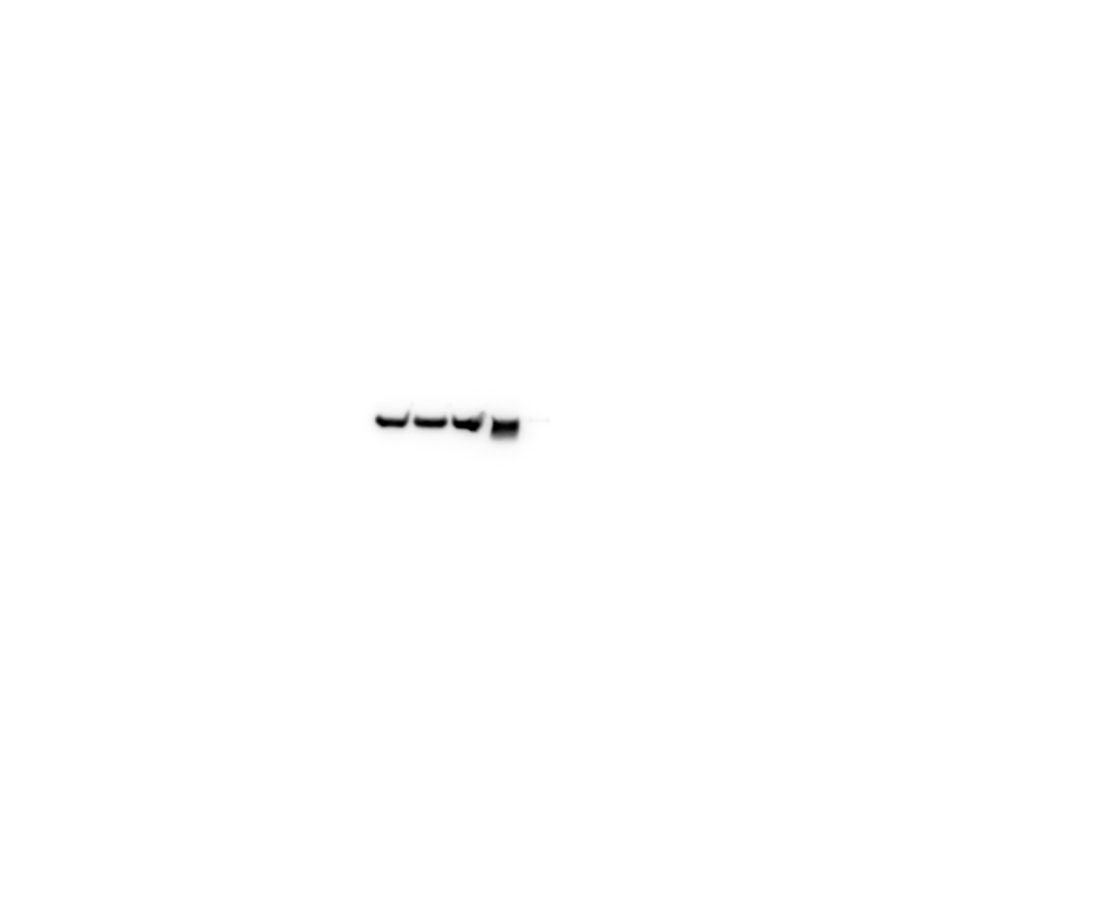

Supplement: Supplementary file 17 — Unprocessed western blots for Fig. 7b. [file 42255_2025_1225_MOESM17_ESM.zip › Zuhra_Unmodified_WesternBlot_Fig7/Experiment5_Hypoxia_actin.jpg]

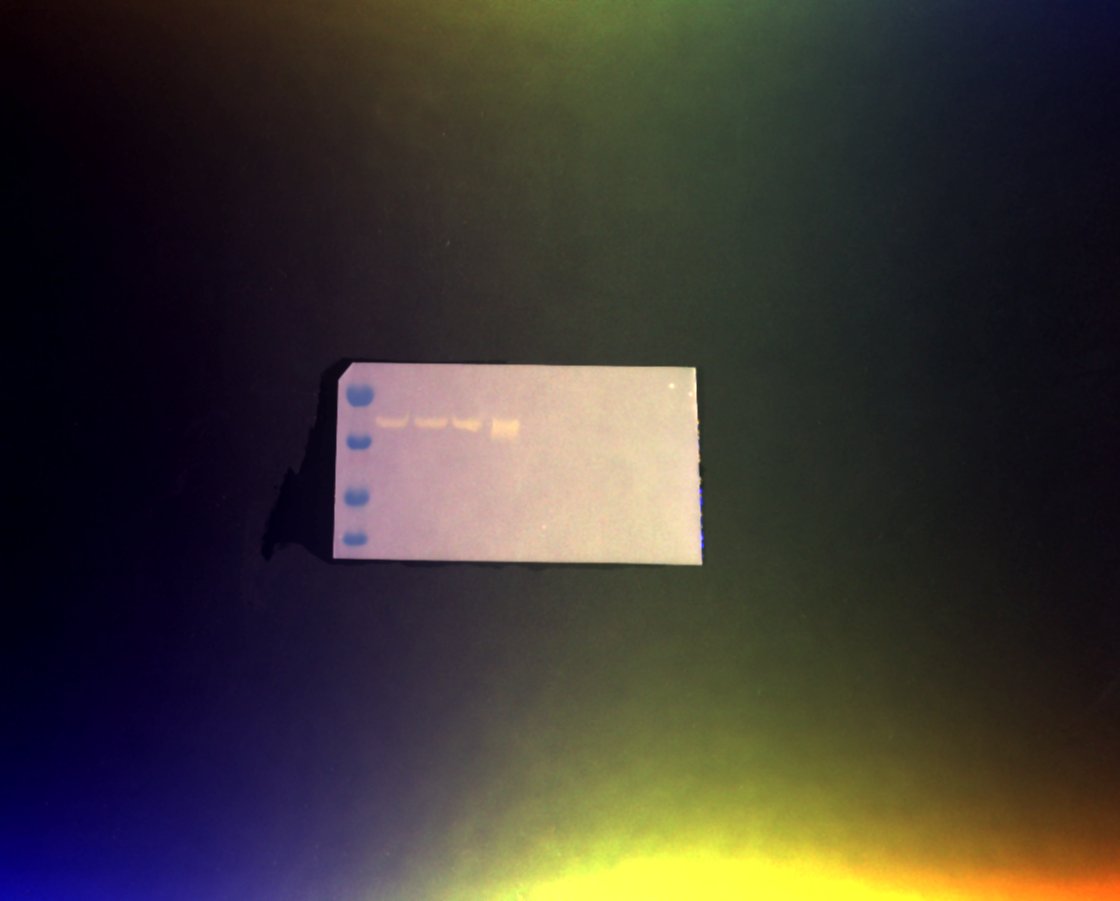

Supplement: Supplementary file 17 — Unprocessed western blots for Fig. 7b. [file 42255_2025_1225_MOESM17_ESM.zip › Zuhra_Unmodified_WesternBlot_Fig7/Experiment5_Hypoxia_actin_marker.jpg]

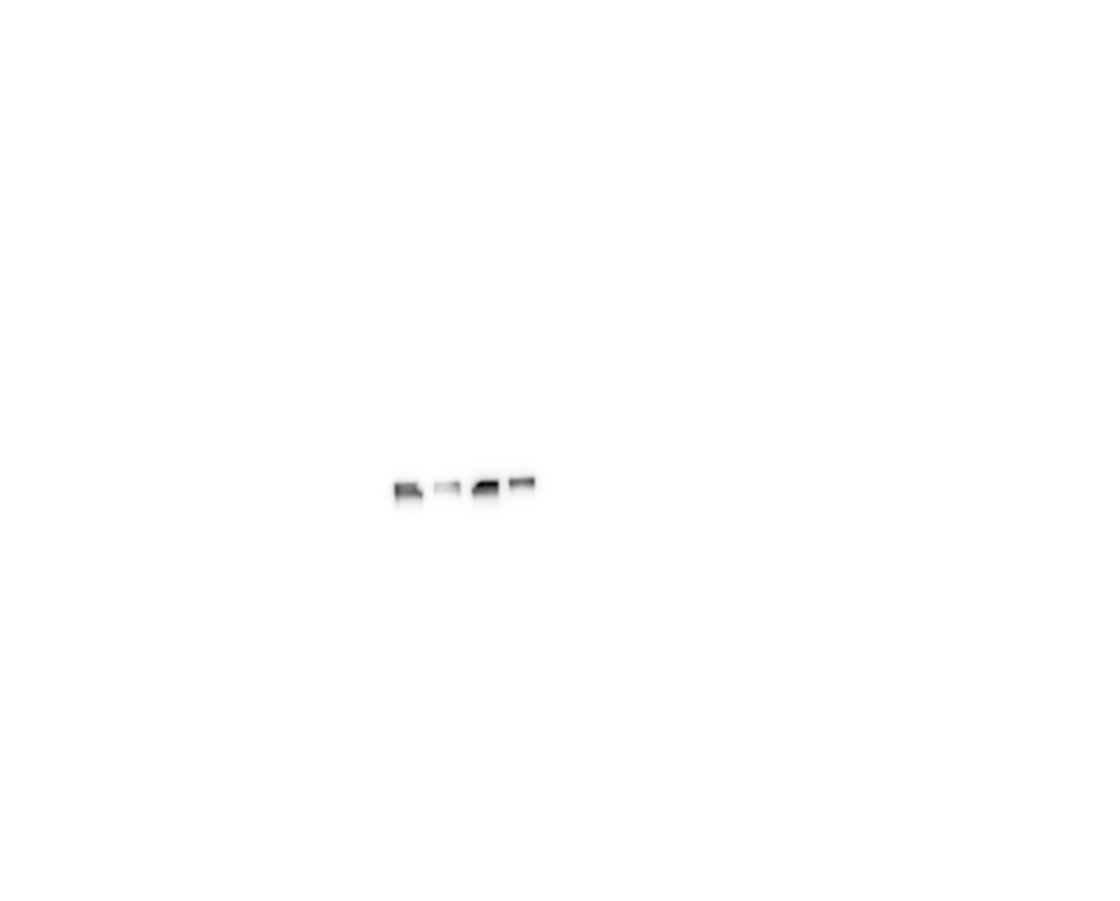

Supplement: Supplementary file 17 — Unprocessed western blots for Fig. 7b. [file 42255_2025_1225_MOESM17_ESM.zip › Zuhra_Unmodified_WesternBlot_Fig7/Experiment5_Hypoxia_Hif1a.jpg]

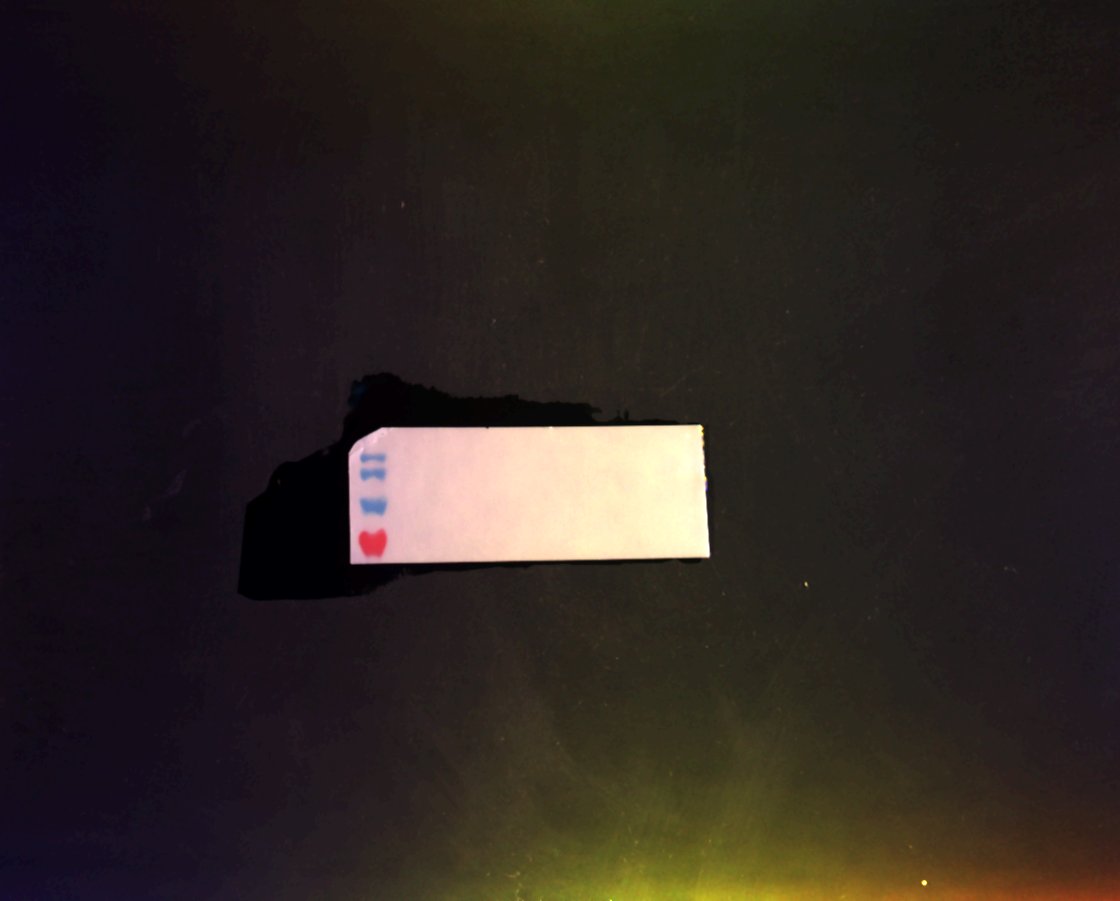

Supplement: Supplementary file 17 — Unprocessed western blots for Fig. 7b. [file 42255_2025_1225_MOESM17_ESM.zip › Zuhra_Unmodified_WesternBlot_Fig7/Experiment5_Hypoxia_Hif1a_marker.jpg]

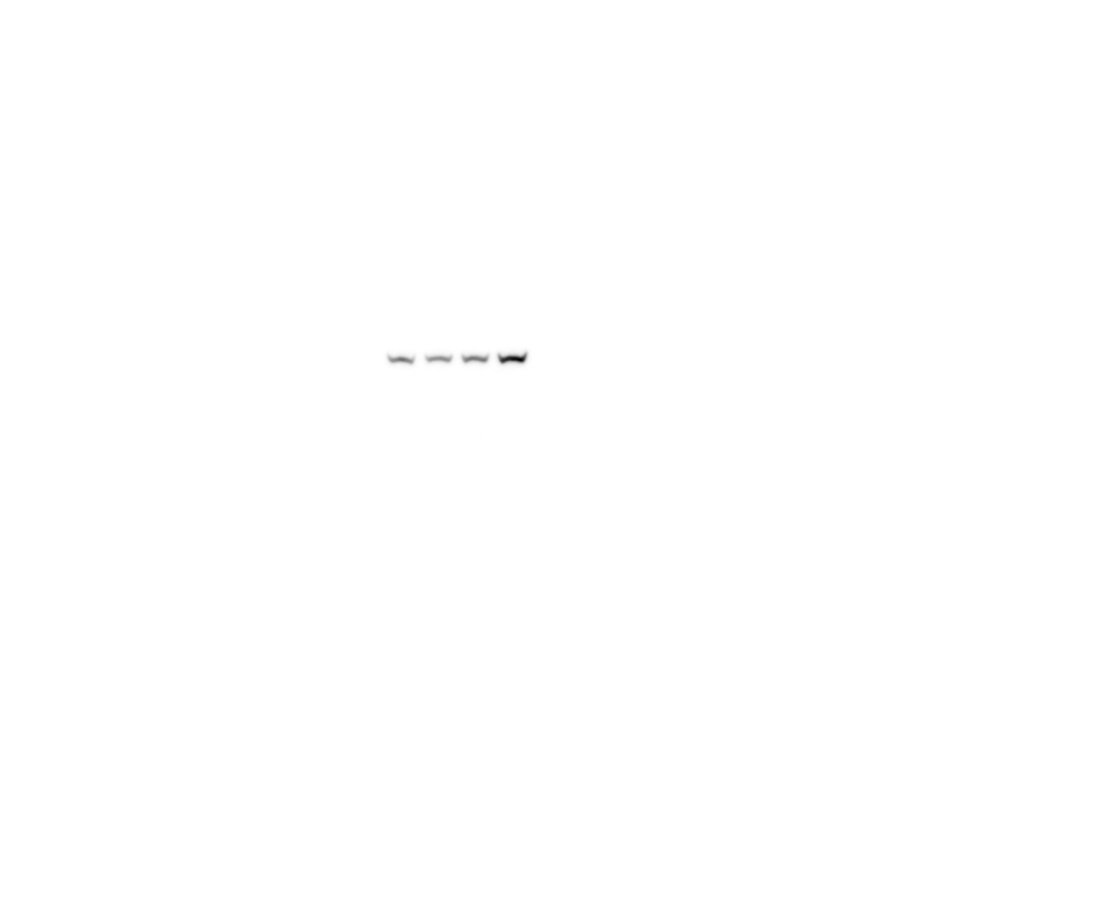

Supplement: Supplementary file 17 — Unprocessed western blots for Fig. 7b. [file 42255_2025_1225_MOESM17_ESM.zip › Zuhra_Unmodified_WesternBlot_Fig7/Experiment5_Normoxia_actin.jpg]

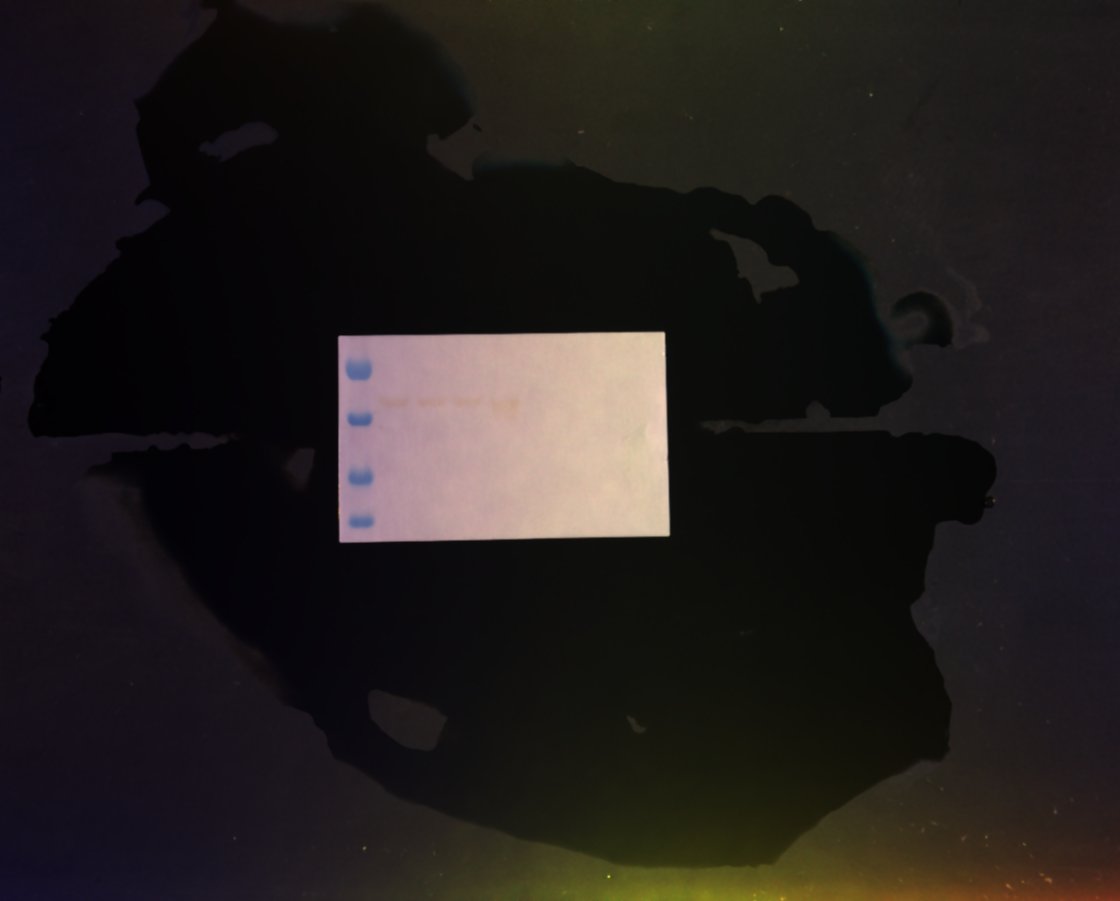

Supplement: Supplementary file 17 — Unprocessed western blots for Fig. 7b. [file 42255_2025_1225_MOESM17_ESM.zip › Zuhra_Unmodified_WesternBlot_Fig7/Experiment5_Normoxia_actin_marker.jpg]

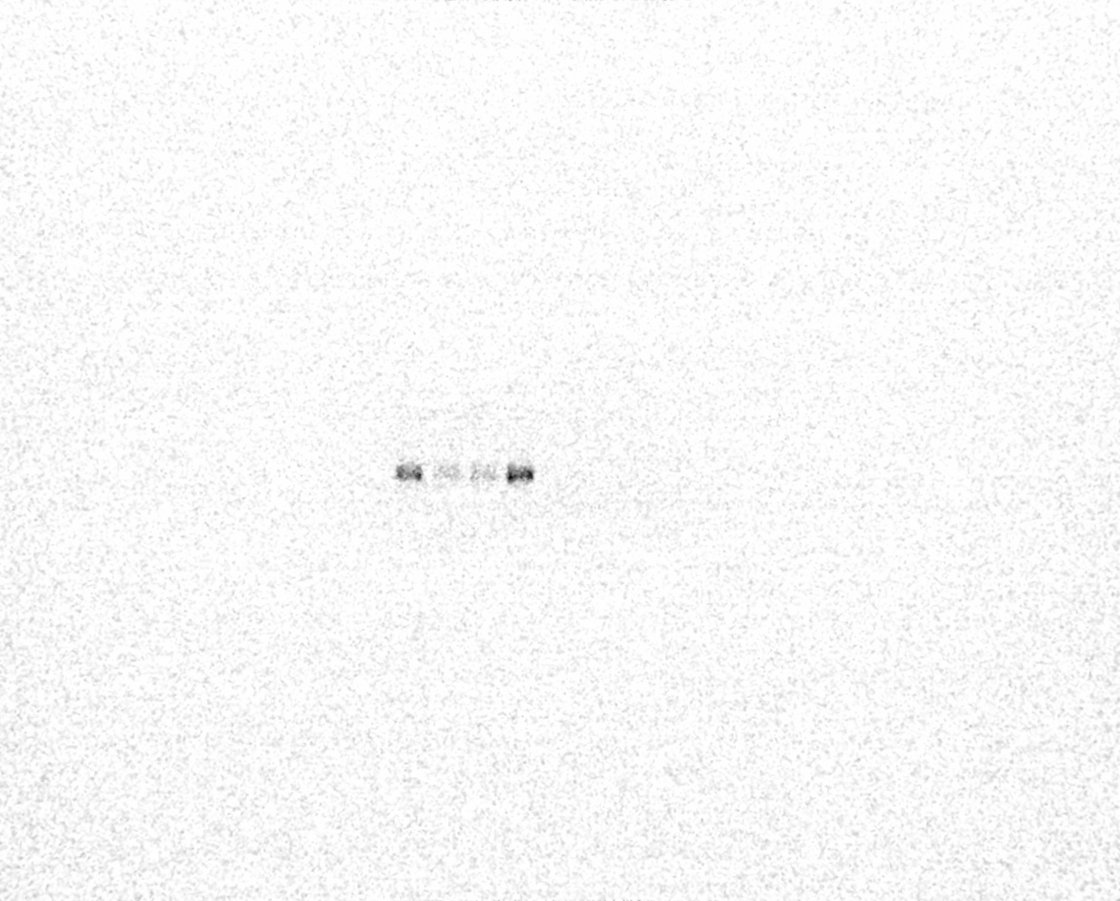

Supplement: Supplementary file 17 — Unprocessed western blots for Fig. 7b. [file 42255_2025_1225_MOESM17_ESM.zip › Zuhra_Unmodified_WesternBlot_Fig7/Experiment5_Normoxia_Hif1a.jpg]

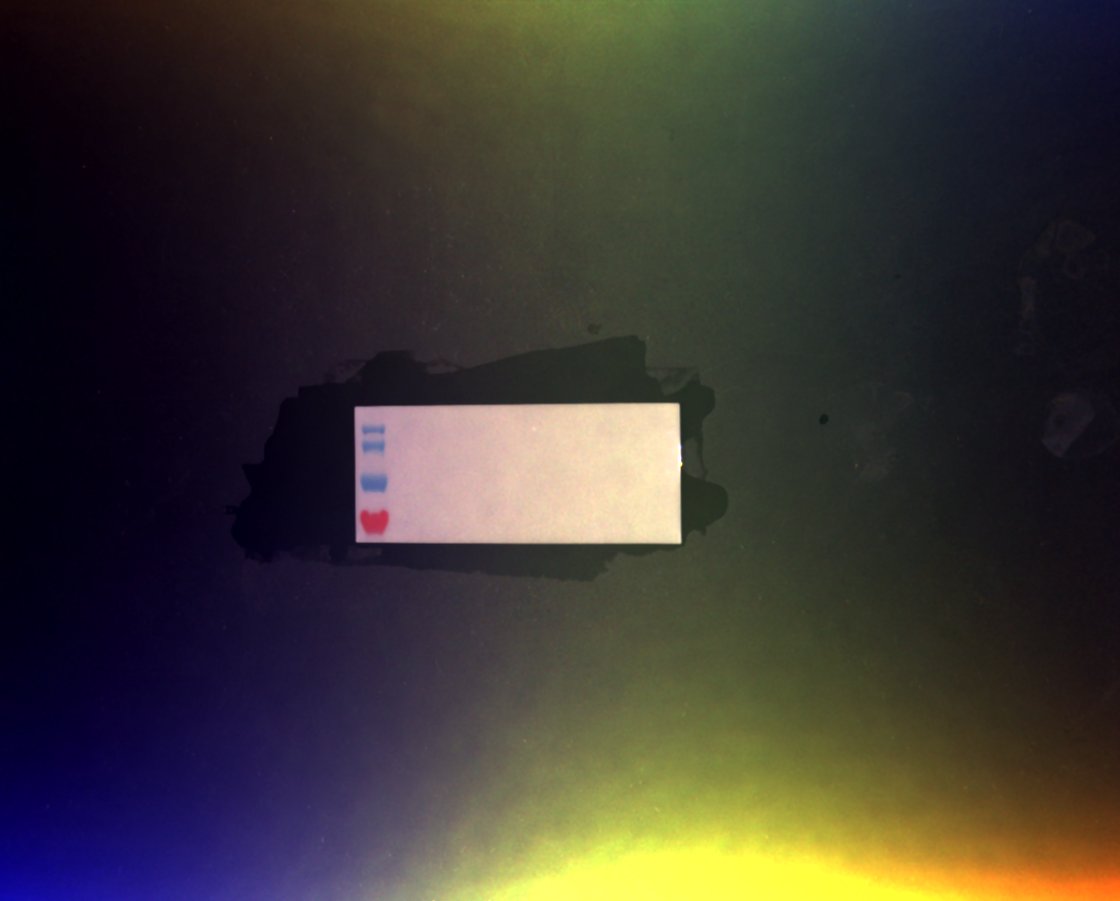

Supplement: Supplementary file 17 — Unprocessed western blots for Fig. 7b. [file 42255_2025_1225_MOESM17_ESM.zip › Zuhra_Unmodified_WesternBlot_Fig7/Experiment5_Normoxia_Hif1a_marker.jpg]

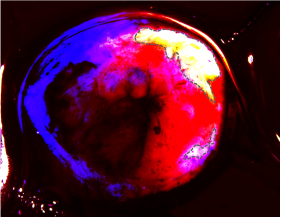

Supplement: Supplementary file 18 — Unmodified images for Fig. 7i. [file 42255_2025_1225_MOESM18_ESM.zip › Zuhra_Microscop_Images_Fig7/myocardial_injury_Amygdalin.tif]

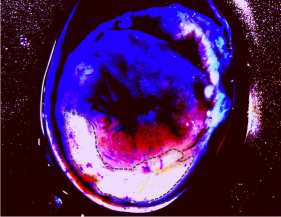

Supplement: Supplementary file 18 — Unmodified images for Fig. 7i. [file 42255_2025_1225_MOESM18_ESM.zip › Zuhra_Microscop_Images_Fig7/myocardial_injury_CTR.tif]

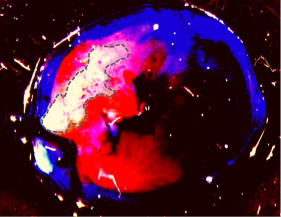

Supplement: Supplementary file 18 — Unmodified images for Fig. 7i. [file 42255_2025_1225_MOESM18_ESM.zip › Zuhra_Microscop_Images_Fig7/myocardial_injury_Glycine.tif]

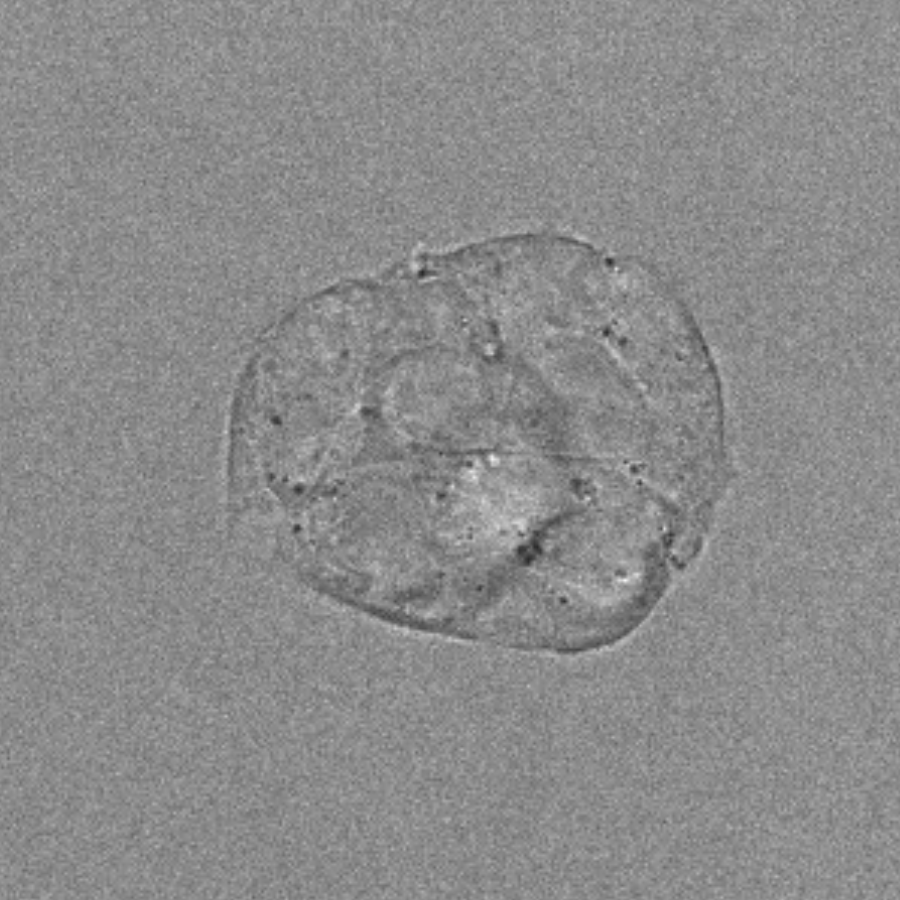

Supplement: Supplementary file 24 — Unmodified confocal images for Extended Data Fig. 3b,c,d. [file 42255_2025_1225_MOESM24_ESM.zip › Zuhra_Microscopy_Images_Extended_Fig3/Zuhra_Microscopy_Images_Extended_Fig3b/CN probe - Calcein/Calcein_HepG2_BF.tif]
