# Supplementary figures and images for: Regulation of mammalian cellular metabolism by endogenous cyanide production (part 2 of 2)
Source: Nat Metab. 2025 Mar 3;7(3):531–55. doi: 10.1038/s42255-025-01225-w (PMC11946912; doi:10.1038/s42255-025-01225-w)

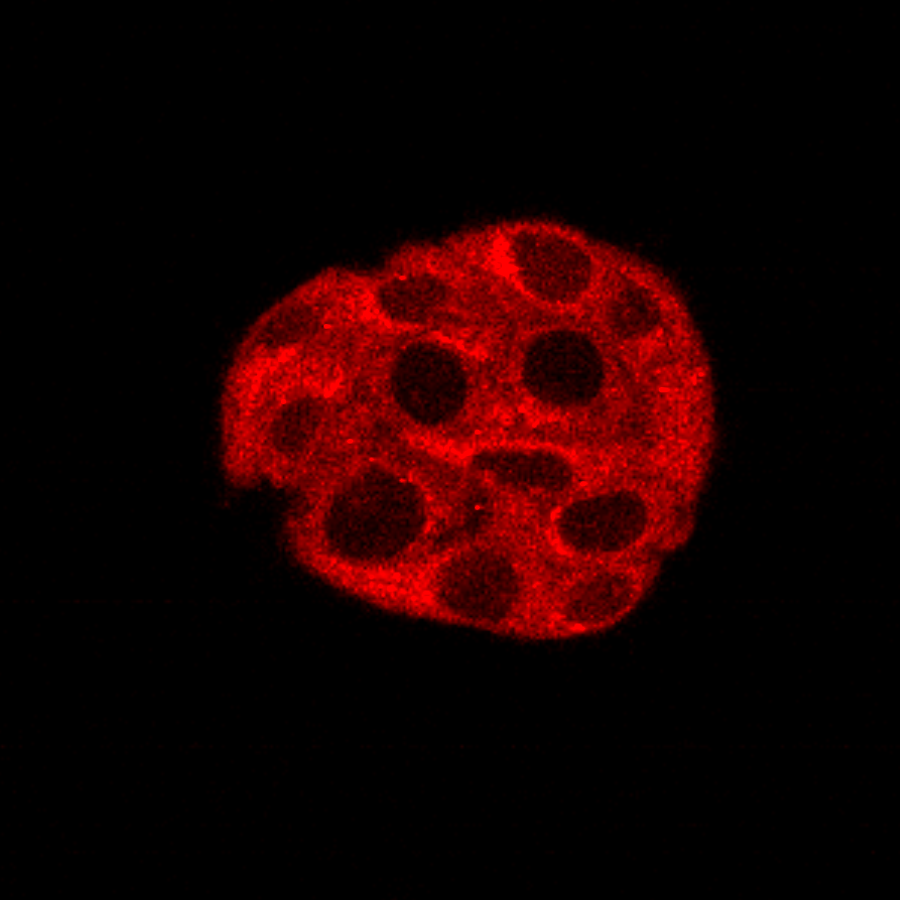

Supplement: Supplementary file 24 — Unmodified confocal images for Extended Data Fig. 3b,c,d. [file 42255_2025_1225_MOESM24_ESM.zip › Zuhra_Microscopy_Images_Extended_Fig3/Zuhra_Microscopy_Images_Extended_Fig3b/CN probe - Calcein/Calcein_HepG2_CP.tif]

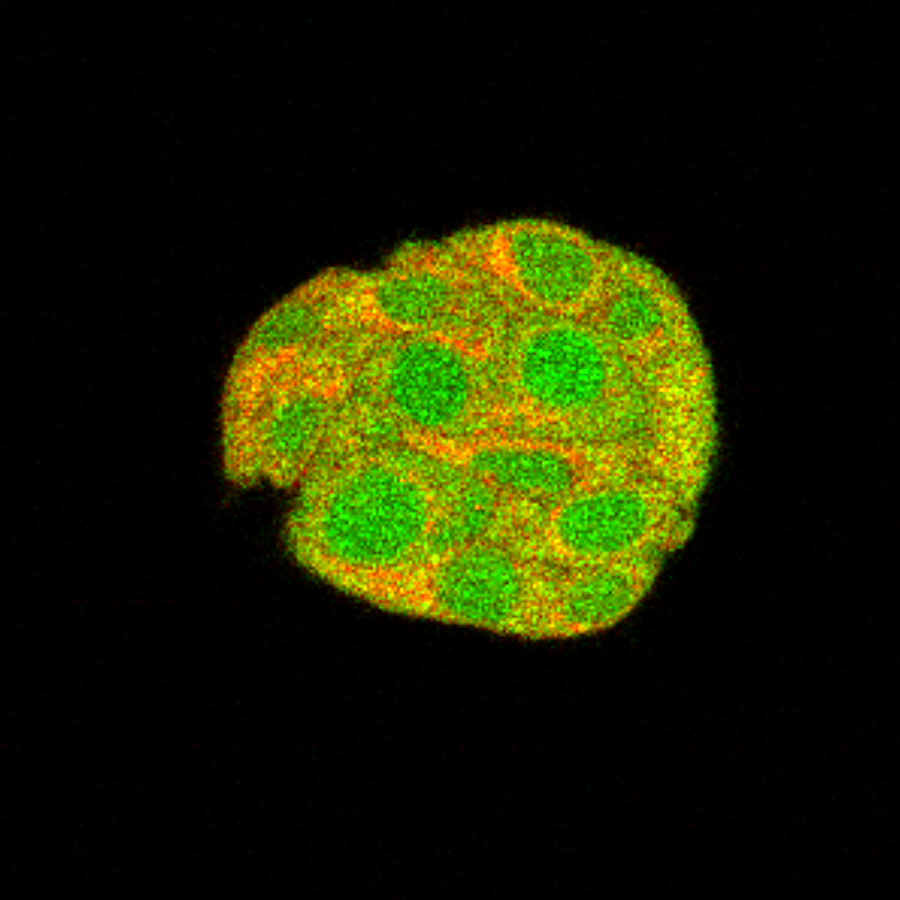

Supplement: Supplementary file 24 — Unmodified confocal images for Extended Data Fig. 3b,c,d. [file 42255_2025_1225_MOESM24_ESM.zip › Zuhra_Microscopy_Images_Extended_Fig3/Zuhra_Microscopy_Images_Extended_Fig3b/CN probe - Calcein/Calcein_HepG2_Merge.tif]

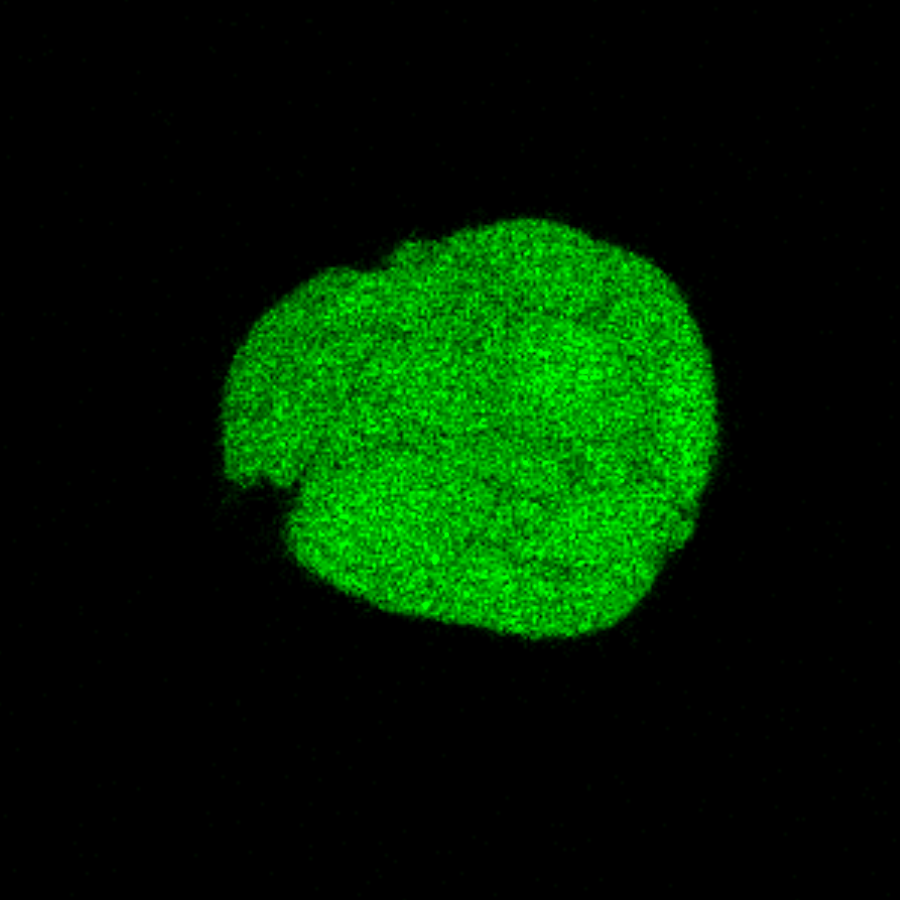

Supplement: Supplementary file 24 — Unmodified confocal images for Extended Data Fig. 3b,c,d. [file 42255_2025_1225_MOESM24_ESM.zip › Zuhra_Microscopy_Images_Extended_Fig3/Zuhra_Microscopy_Images_Extended_Fig3b/CN probe - Calcein/FigS3b_Calcein_HepG2_Calcein.tif]

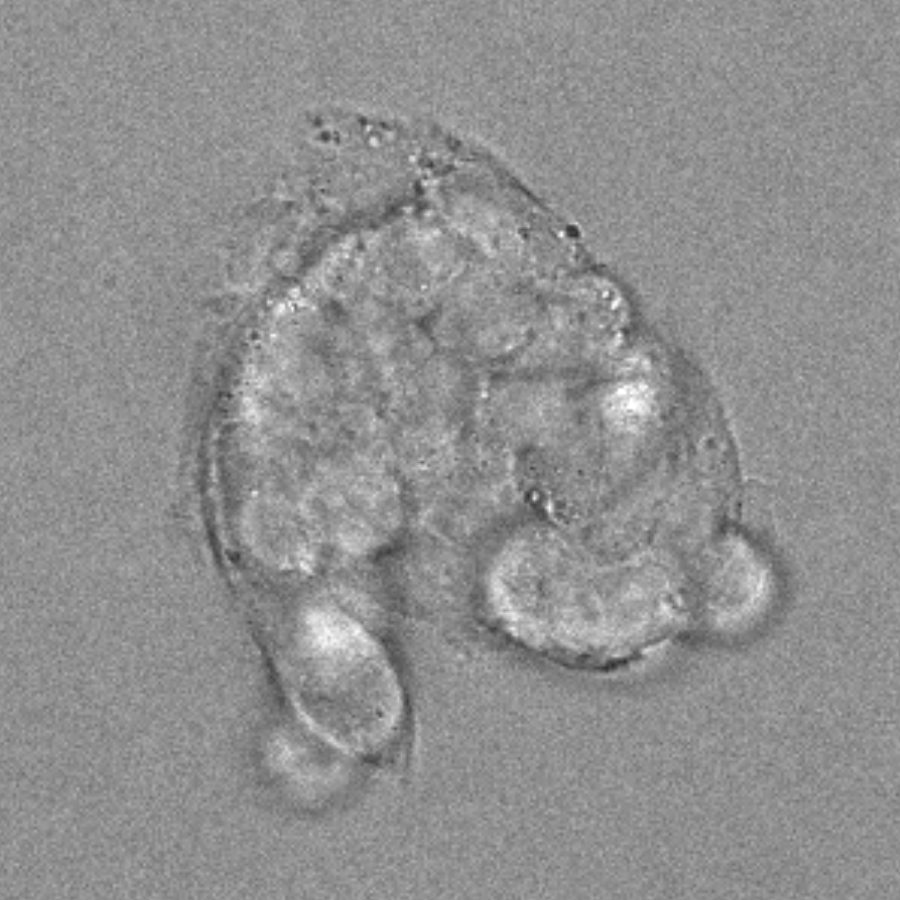

Supplement: Supplementary file 24 — Unmodified confocal images for Extended Data Fig. 3b,c,d. [file 42255_2025_1225_MOESM24_ESM.zip › Zuhra_Microscopy_Images_Extended_Fig3/Zuhra_Microscopy_Images_Extended_Fig3b/CN probe - Cell mask/CellMask_HepG2_BF.tif]

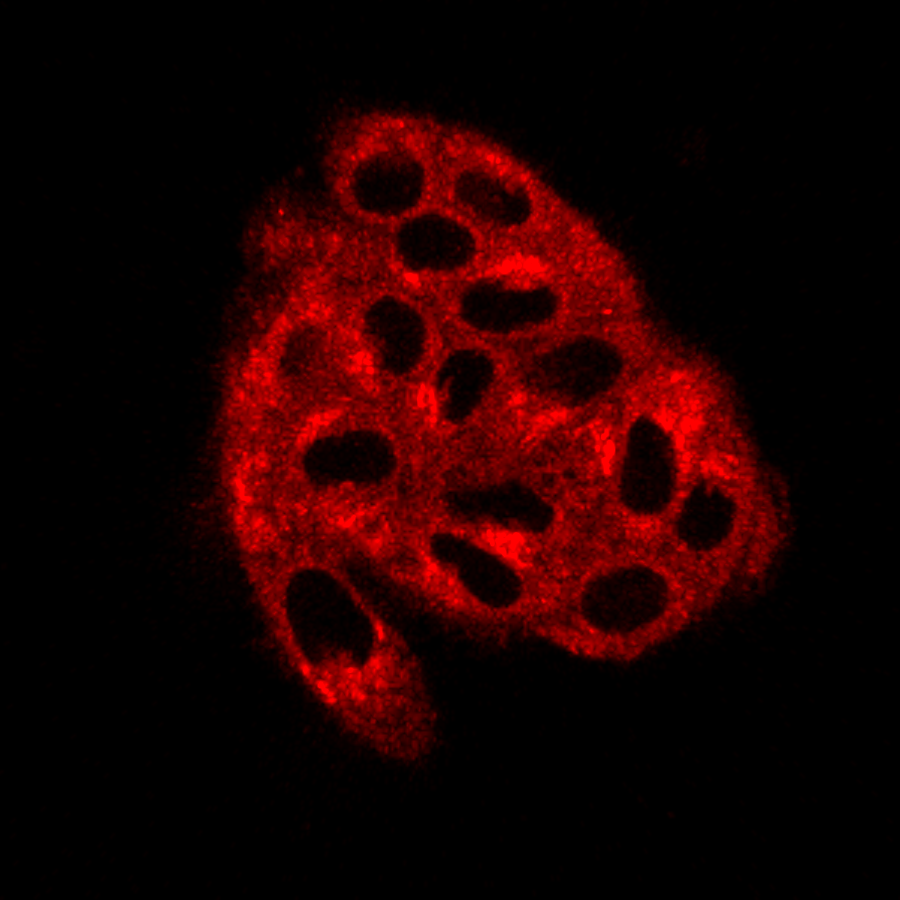

Supplement: Supplementary file 24 — Unmodified confocal images for Extended Data Fig. 3b,c,d. [file 42255_2025_1225_MOESM24_ESM.zip › Zuhra_Microscopy_Images_Extended_Fig3/Zuhra_Microscopy_Images_Extended_Fig3b/CN probe - Cell mask/CellMask_HepG2_CP.tif]

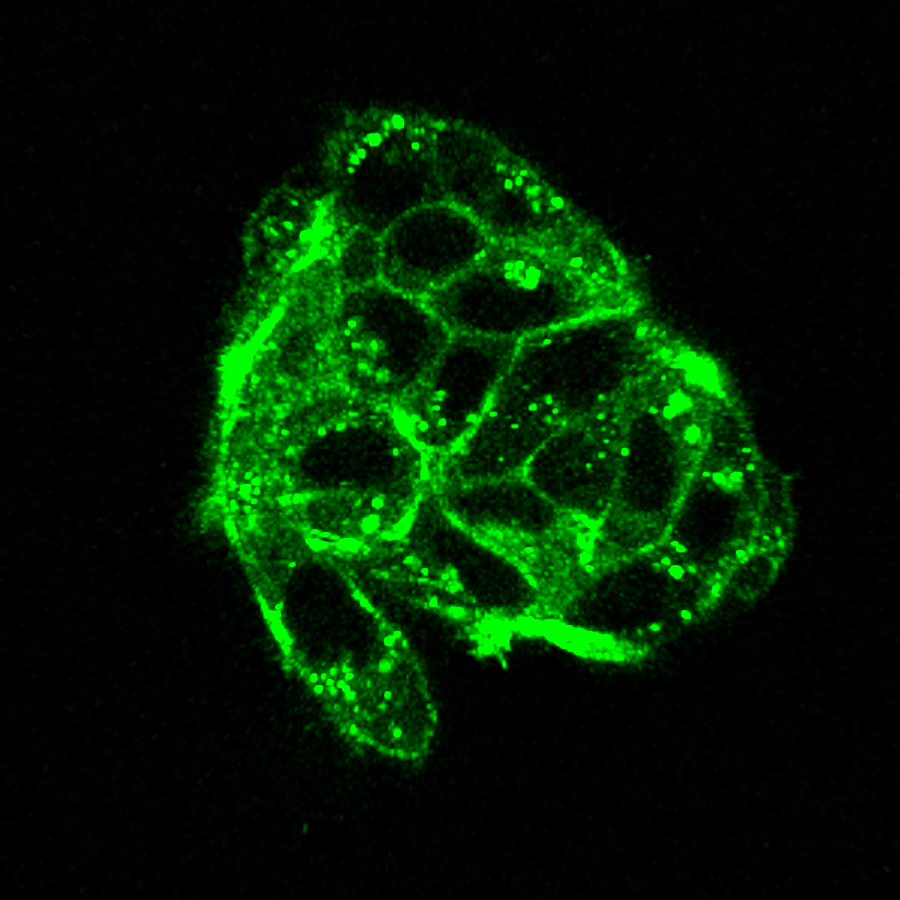

Supplement: Supplementary file 24 — Unmodified confocal images for Extended Data Fig. 3b,c,d. [file 42255_2025_1225_MOESM24_ESM.zip › Zuhra_Microscopy_Images_Extended_Fig3/Zuhra_Microscopy_Images_Extended_Fig3b/CN probe - Cell mask/FigS3b_CellMask_HepG2_CellMask.tif]

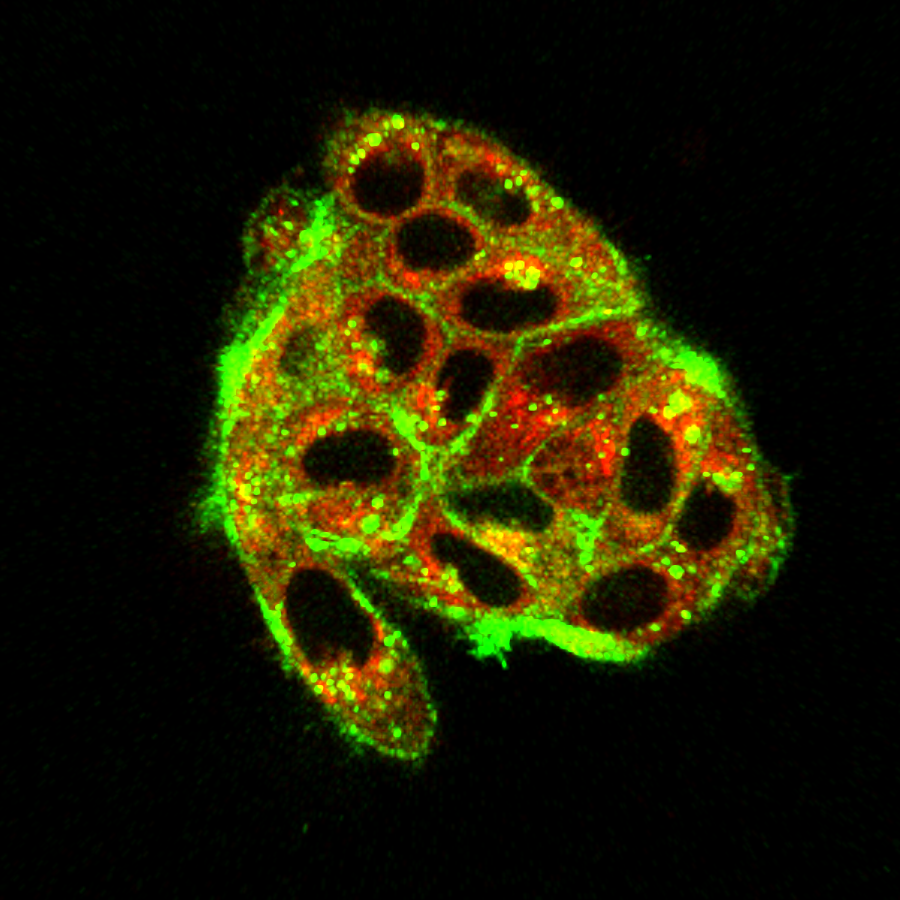

Supplement: Supplementary file 24 — Unmodified confocal images for Extended Data Fig. 3b,c,d. [file 42255_2025_1225_MOESM24_ESM.zip › Zuhra_Microscopy_Images_Extended_Fig3/Zuhra_Microscopy_Images_Extended_Fig3b/CN probe - Cell mask/FigS3b_CellMask_HepG2_Merge.tif]

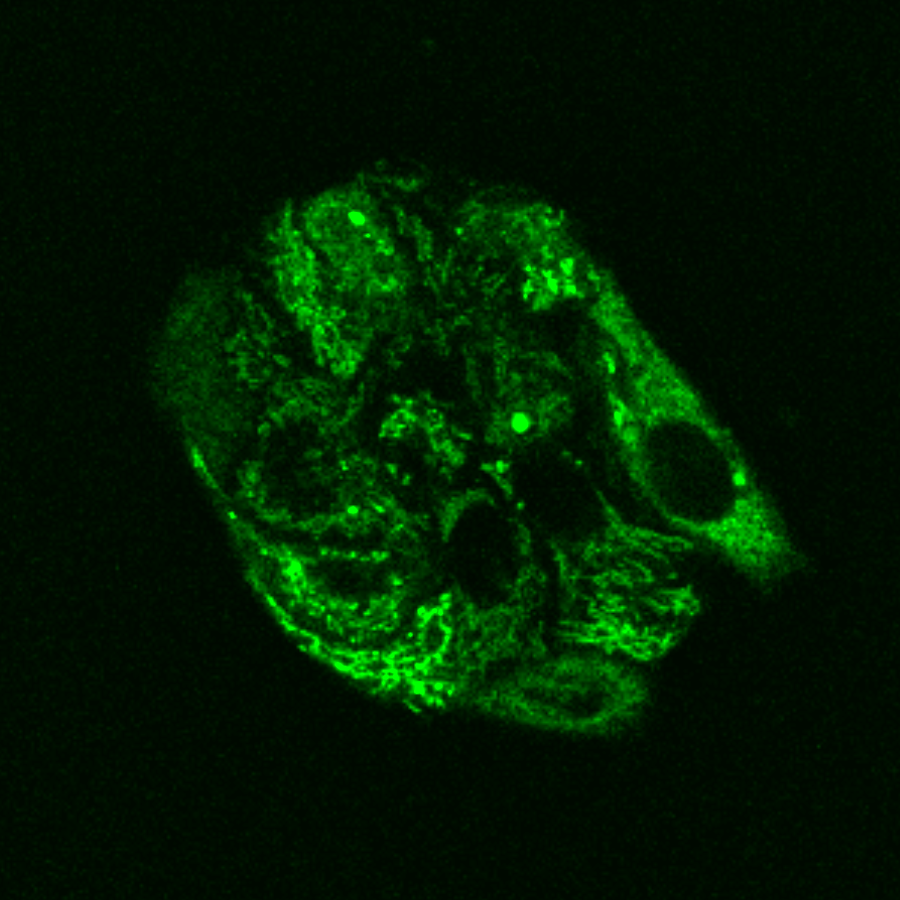

Supplement: Supplementary file 24 — Unmodified confocal images for Extended Data Fig. 3b,c,d. [file 42255_2025_1225_MOESM24_ESM.zip › Zuhra_Microscopy_Images_Extended_Fig3/Zuhra_Microscopy_Images_Extended_Fig3b/CN probe - MTRdR/FigS3b_MTRdR_HepG2_MitoTracker.tif]

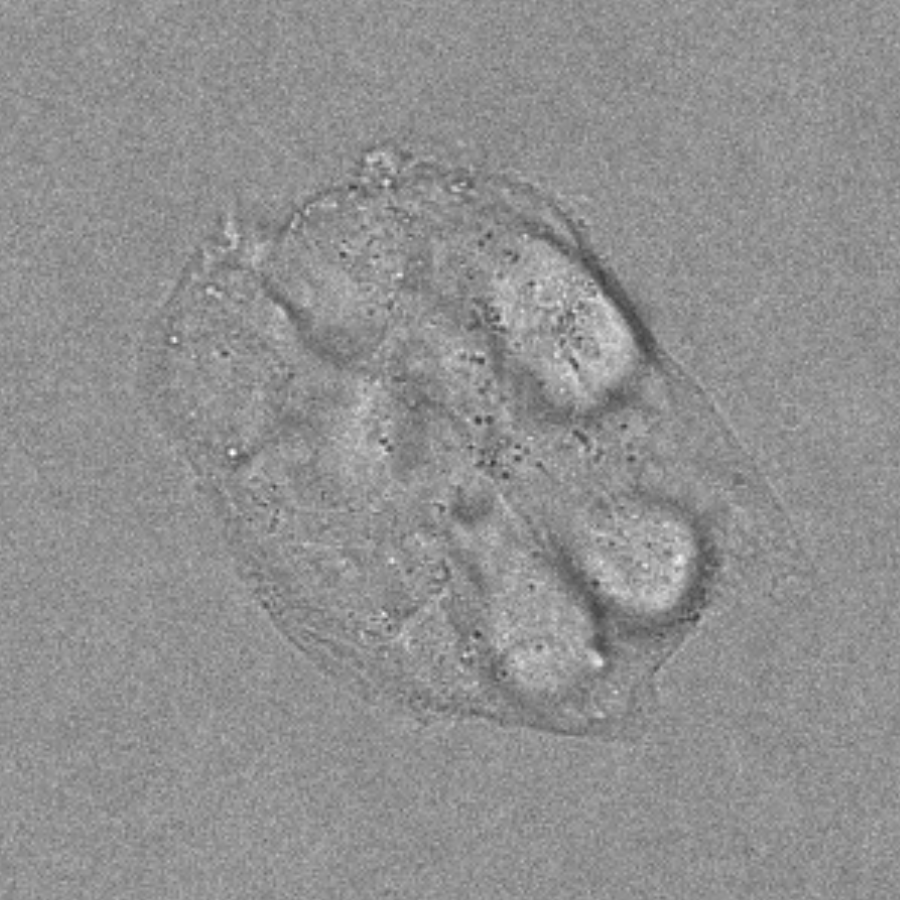

Supplement: Supplementary file 24 — Unmodified confocal images for Extended Data Fig. 3b,c,d. [file 42255_2025_1225_MOESM24_ESM.zip › Zuhra_Microscopy_Images_Extended_Fig3/Zuhra_Microscopy_Images_Extended_Fig3b/CN probe - MTRdR/MTRdR_HepG2_BF.tif]

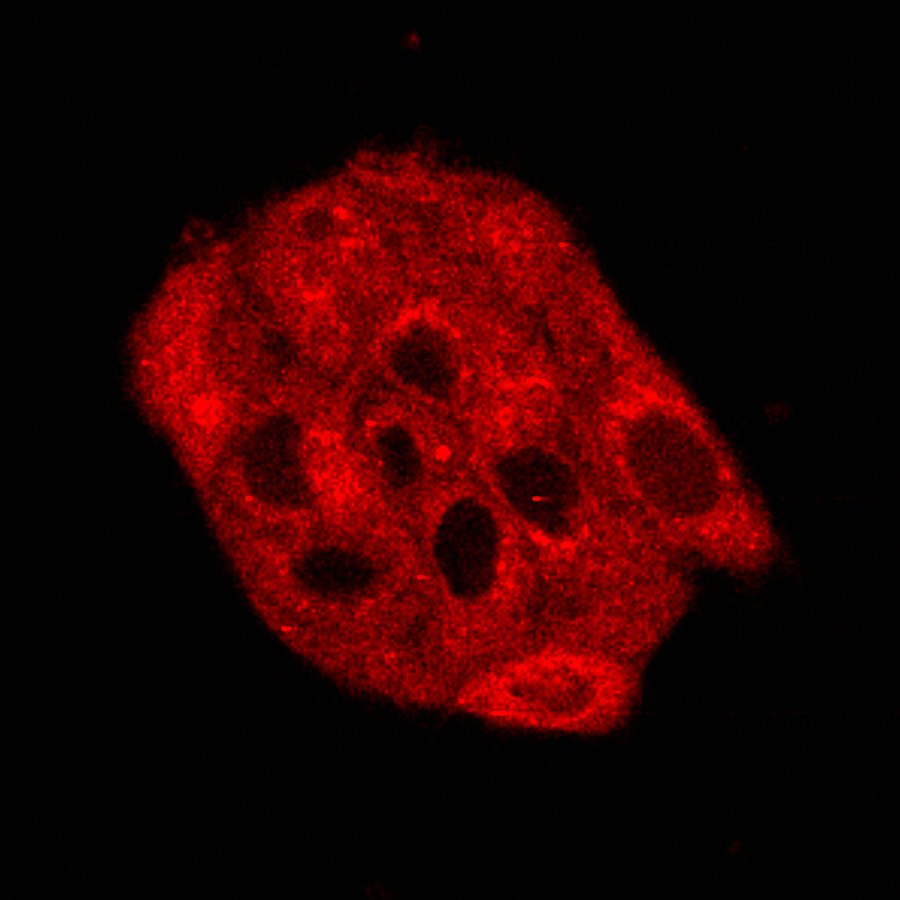

Supplement: Supplementary file 24 — Unmodified confocal images for Extended Data Fig. 3b,c,d. [file 42255_2025_1225_MOESM24_ESM.zip › Zuhra_Microscopy_Images_Extended_Fig3/Zuhra_Microscopy_Images_Extended_Fig3b/CN probe - MTRdR/MTRdR_HepG2_CP.tif]

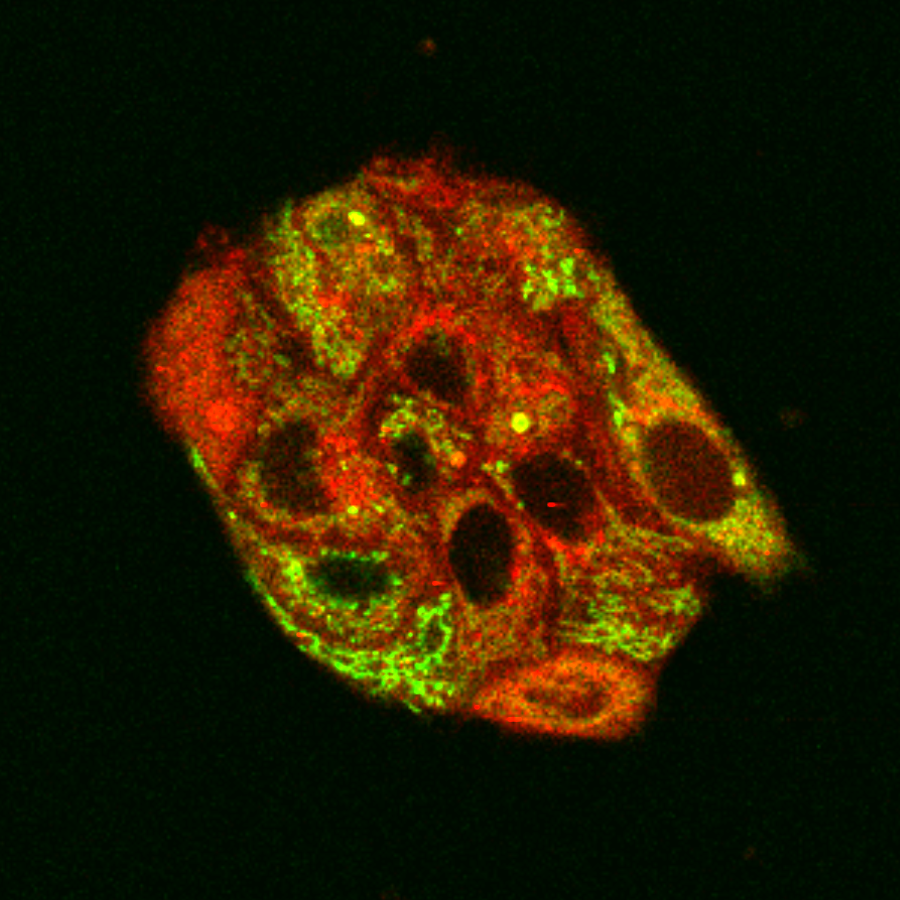

Supplement: Supplementary file 24 — Unmodified confocal images for Extended Data Fig. 3b,c,d. [file 42255_2025_1225_MOESM24_ESM.zip › Zuhra_Microscopy_Images_Extended_Fig3/Zuhra_Microscopy_Images_Extended_Fig3b/CN probe - MTRdR/MTRdR_HepG2_Merge.tif]

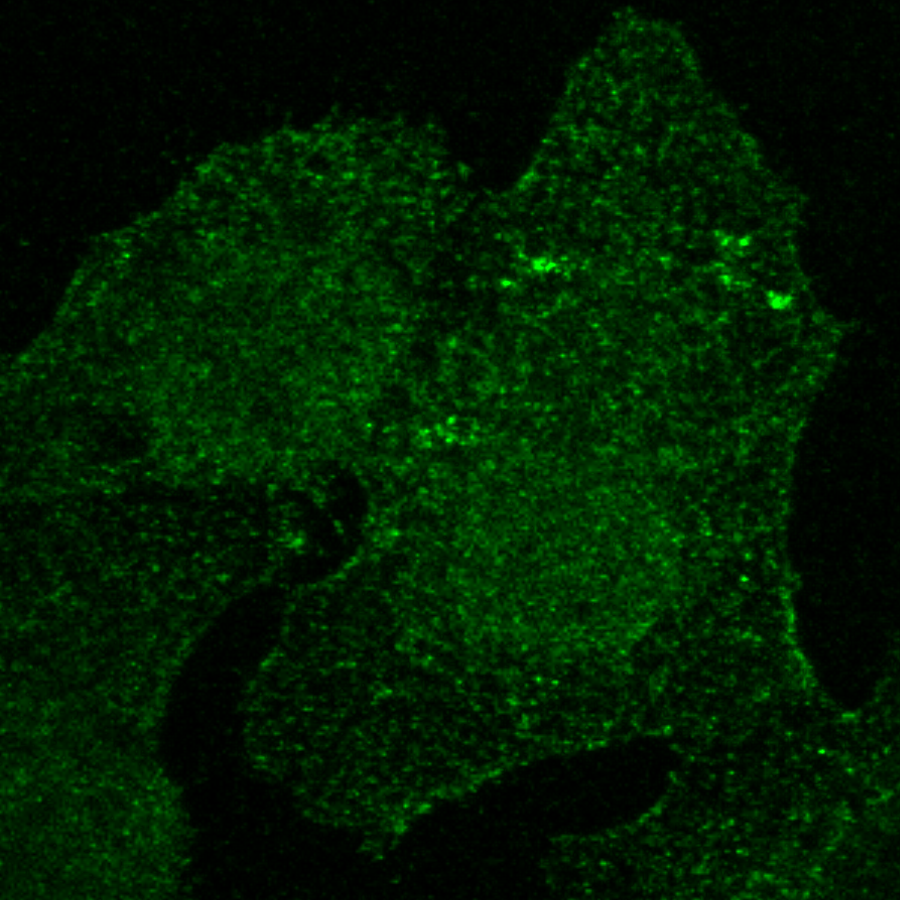

Supplement: Supplementary file 24 — Unmodified confocal images for Extended Data Fig. 3b,c,d. [file 42255_2025_1225_MOESM24_ESM.zip › Zuhra_Microscopy_Images_Extended_Fig3/Zuhra_Microscopy_Images_Extended_Fig3c/ER-MPO/ER-MPO_HepG2_ER.tif]

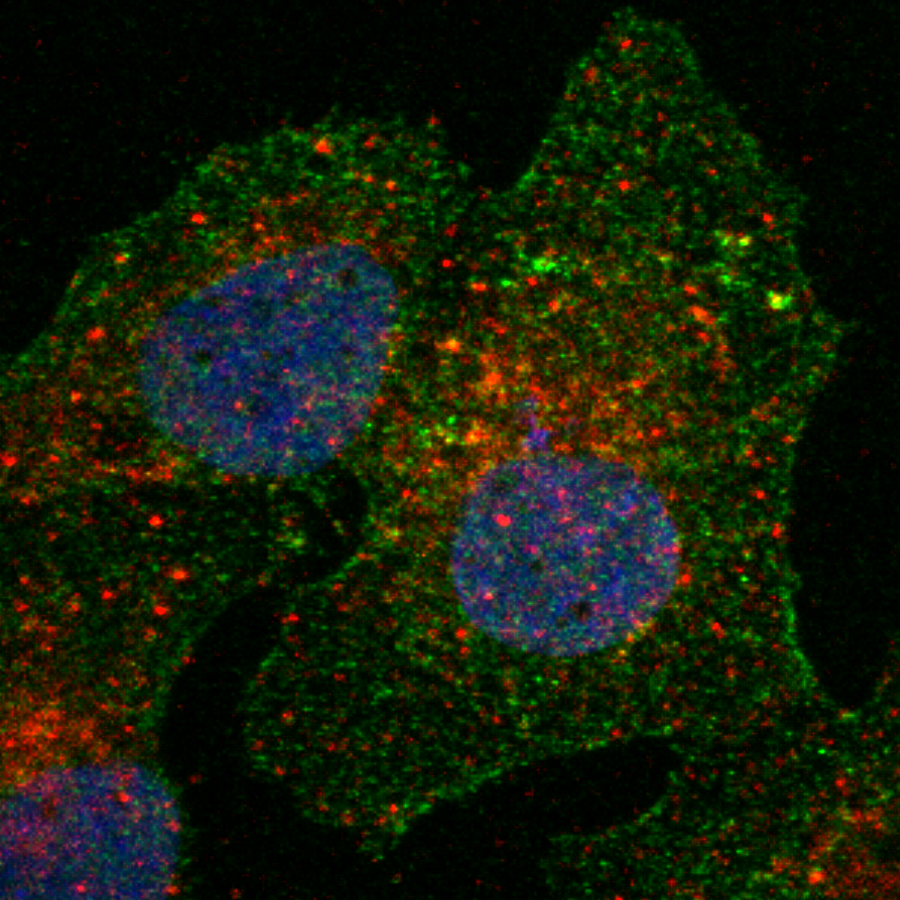

Supplement: Supplementary file 24 — Unmodified confocal images for Extended Data Fig. 3b,c,d. [file 42255_2025_1225_MOESM24_ESM.zip › Zuhra_Microscopy_Images_Extended_Fig3/Zuhra_Microscopy_Images_Extended_Fig3c/ER-MPO/ER-MPO_HepG2_Merge.tif]

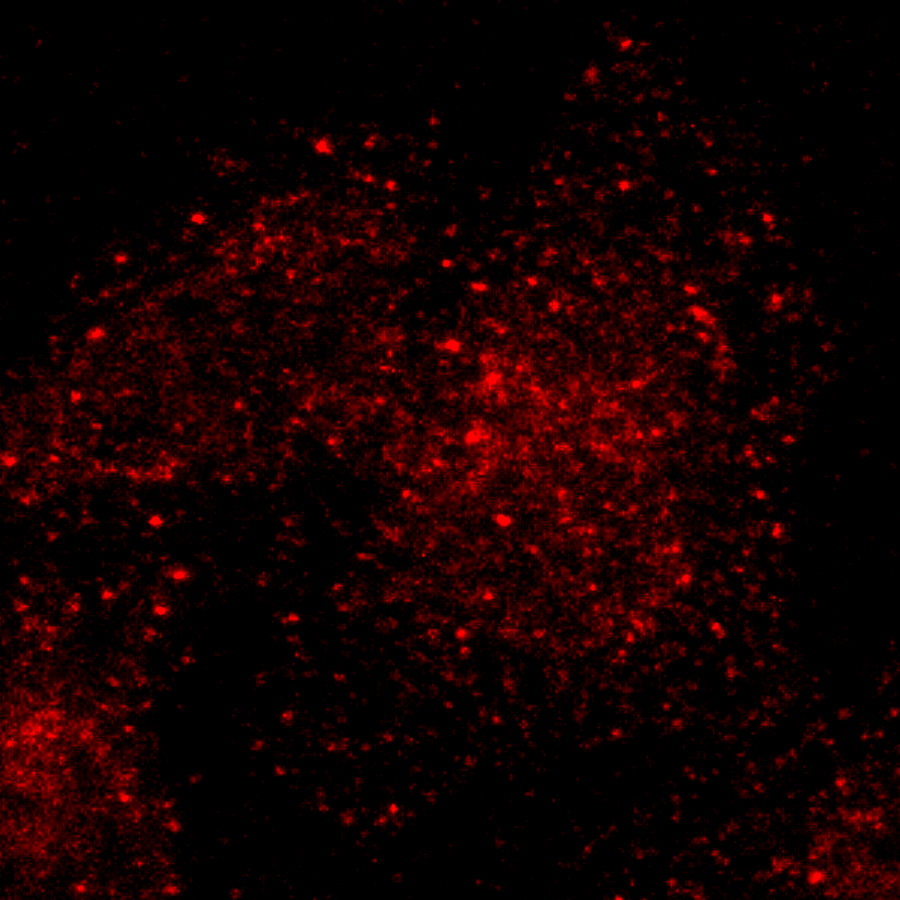

Supplement: Supplementary file 24 — Unmodified confocal images for Extended Data Fig. 3b,c,d. [file 42255_2025_1225_MOESM24_ESM.zip › Zuhra_Microscopy_Images_Extended_Fig3/Zuhra_Microscopy_Images_Extended_Fig3c/ER-MPO/ER-MPO_HepG2_MPO.tif]

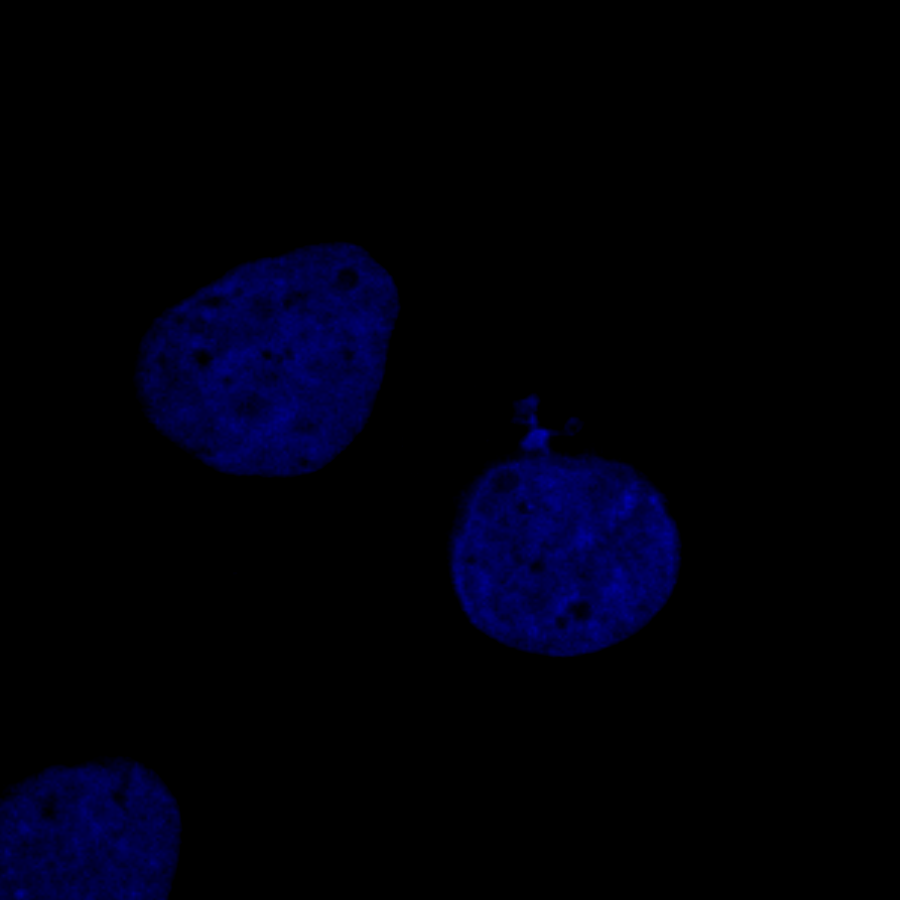

Supplement: Supplementary file 24 — Unmodified confocal images for Extended Data Fig. 3b,c,d. [file 42255_2025_1225_MOESM24_ESM.zip › Zuhra_Microscopy_Images_Extended_Fig3/Zuhra_Microscopy_Images_Extended_Fig3c/ER-MPO/ER-MPO_HepG2_Nuclei.tif]

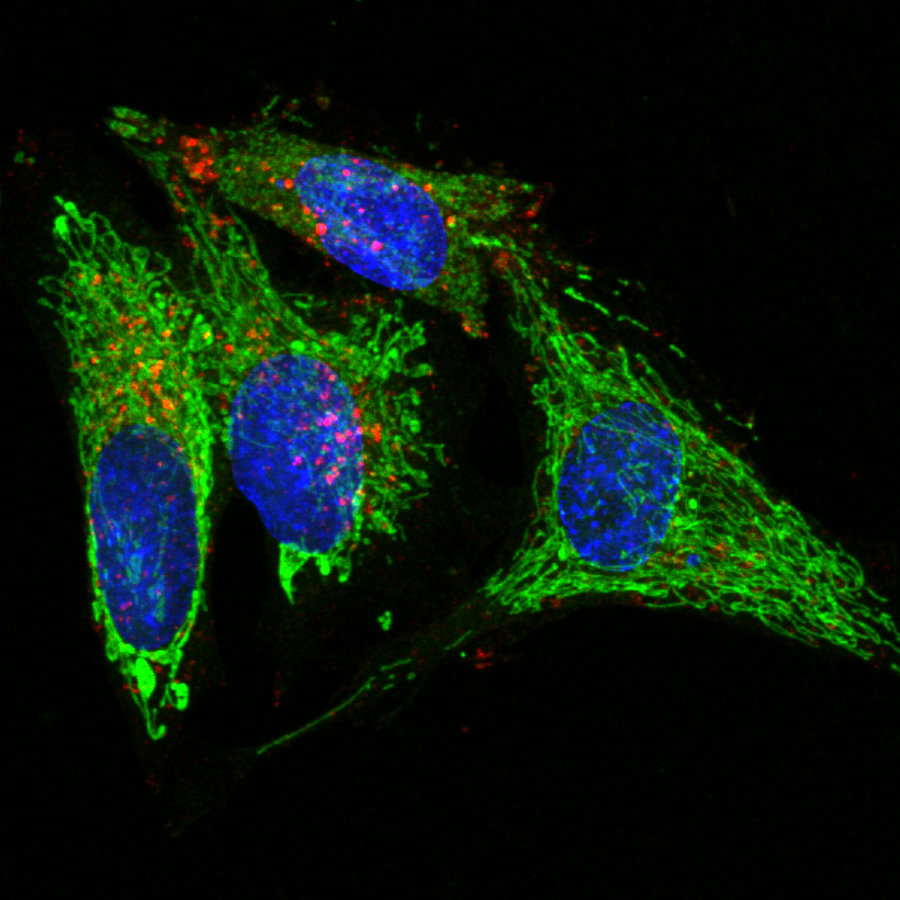

Supplement: Supplementary file 24 — Unmodified confocal images for Extended Data Fig. 3b,c,d. [file 42255_2025_1225_MOESM24_ESM.zip › Zuhra_Microscopy_Images_Extended_Fig3/Zuhra_Microscopy_Images_Extended_Fig3c/Mito-MPO/Mito-MPO_HepG2_Merge.tif]

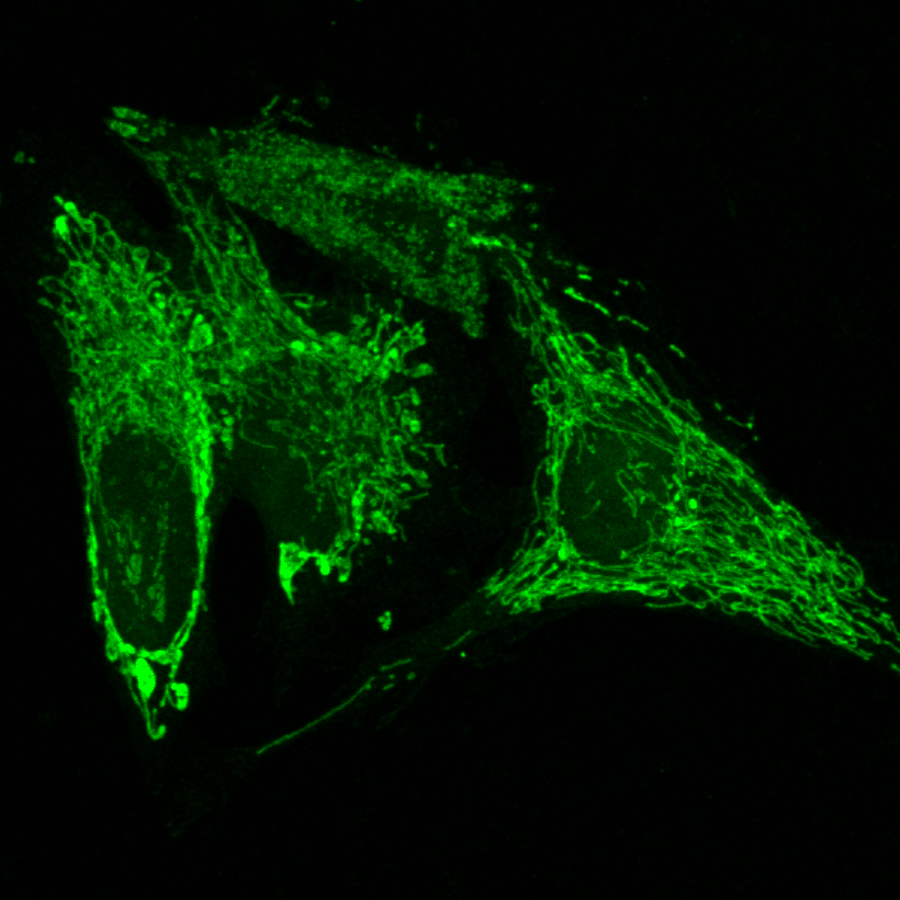

Supplement: Supplementary file 24 — Unmodified confocal images for Extended Data Fig. 3b,c,d. [file 42255_2025_1225_MOESM24_ESM.zip › Zuhra_Microscopy_Images_Extended_Fig3/Zuhra_Microscopy_Images_Extended_Fig3c/Mito-MPO/Mito-MPO_HepG2_Mitochondria.tif]

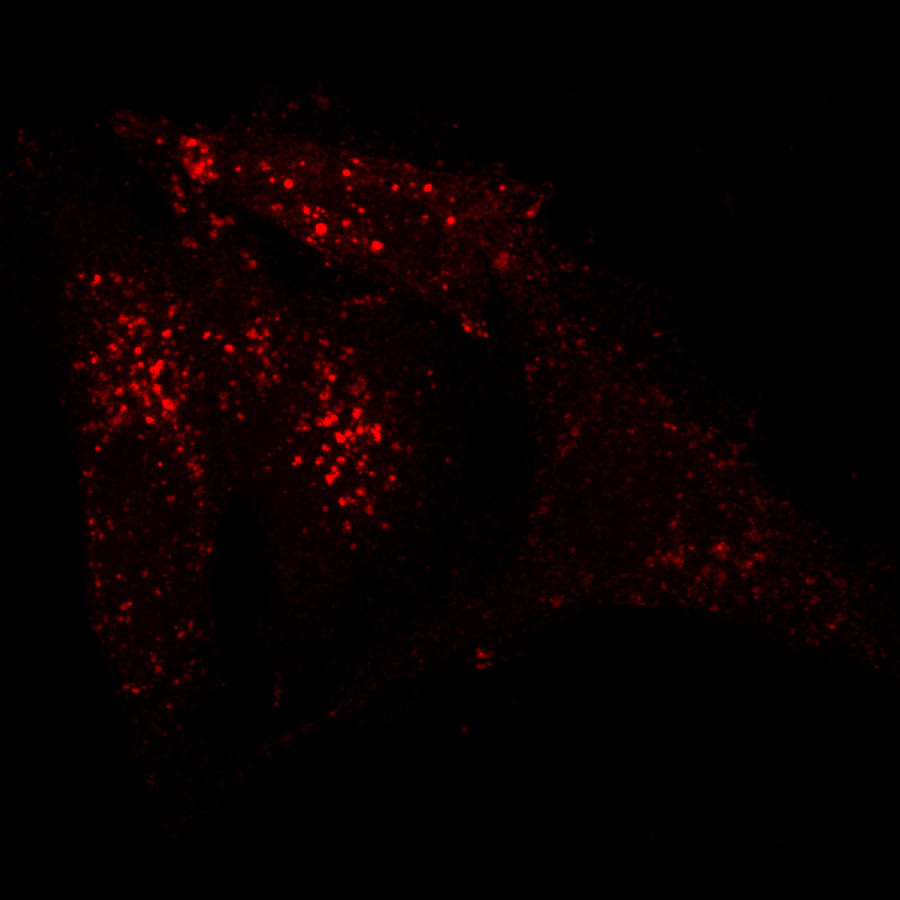

Supplement: Supplementary file 24 — Unmodified confocal images for Extended Data Fig. 3b,c,d. [file 42255_2025_1225_MOESM24_ESM.zip › Zuhra_Microscopy_Images_Extended_Fig3/Zuhra_Microscopy_Images_Extended_Fig3c/Mito-MPO/Mito-MPO_HepG2_MPO.tif]

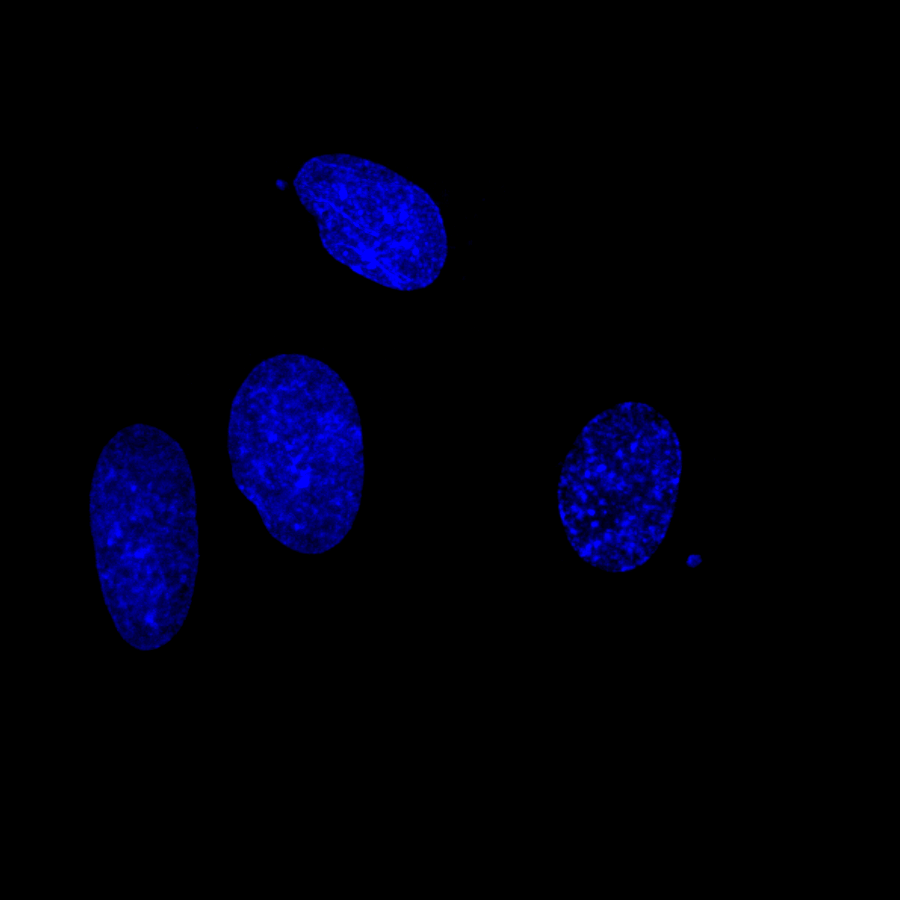

Supplement: Supplementary file 24 — Unmodified confocal images for Extended Data Fig. 3b,c,d. [file 42255_2025_1225_MOESM24_ESM.zip › Zuhra_Microscopy_Images_Extended_Fig3/Zuhra_Microscopy_Images_Extended_Fig3c/Mito-MPO/Mito-MPO_HepG2_Nuclei.tif]

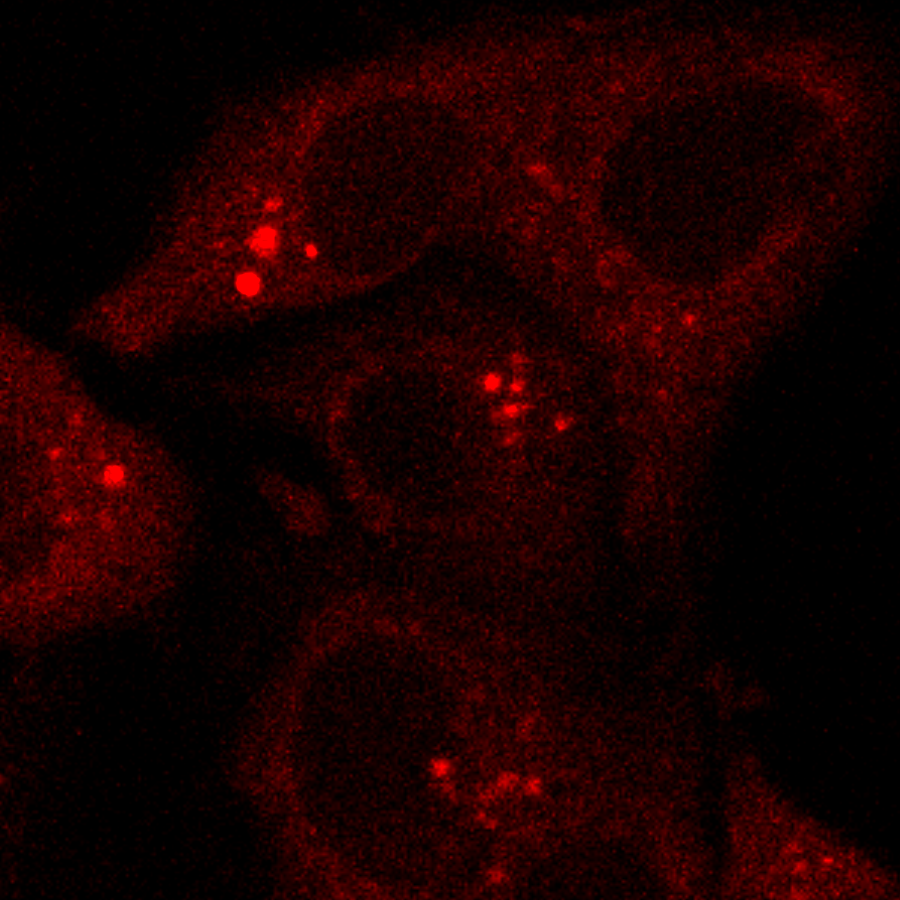

Supplement: Supplementary file 24 — Unmodified confocal images for Extended Data Fig. 3b,c,d. [file 42255_2025_1225_MOESM24_ESM.zip › Zuhra_Microscopy_Images_Extended_Fig3/Zuhra_Microscopy_Images_Extended_Fig3d/HOCl - LysoTracker/HepG2_HOCl.tif]

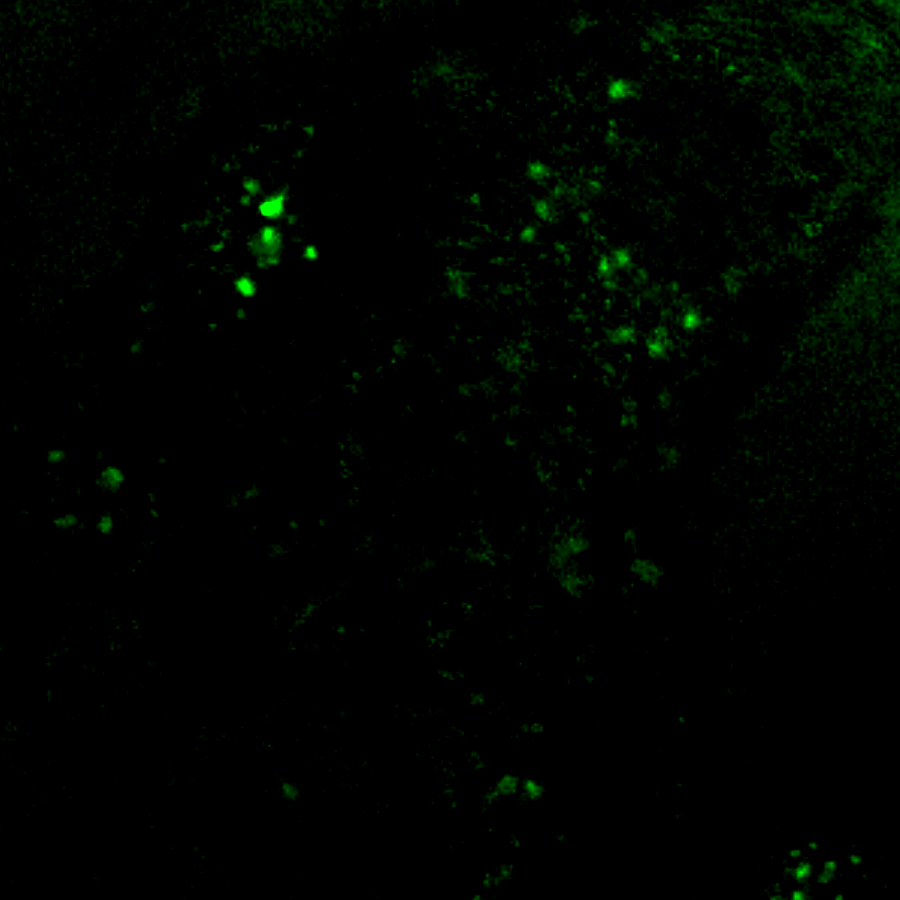

Supplement: Supplementary file 24 — Unmodified confocal images for Extended Data Fig. 3b,c,d. [file 42255_2025_1225_MOESM24_ESM.zip › Zuhra_Microscopy_Images_Extended_Fig3/Zuhra_Microscopy_Images_Extended_Fig3d/HOCl - LysoTracker/HepG2_LysoTracker.tif]

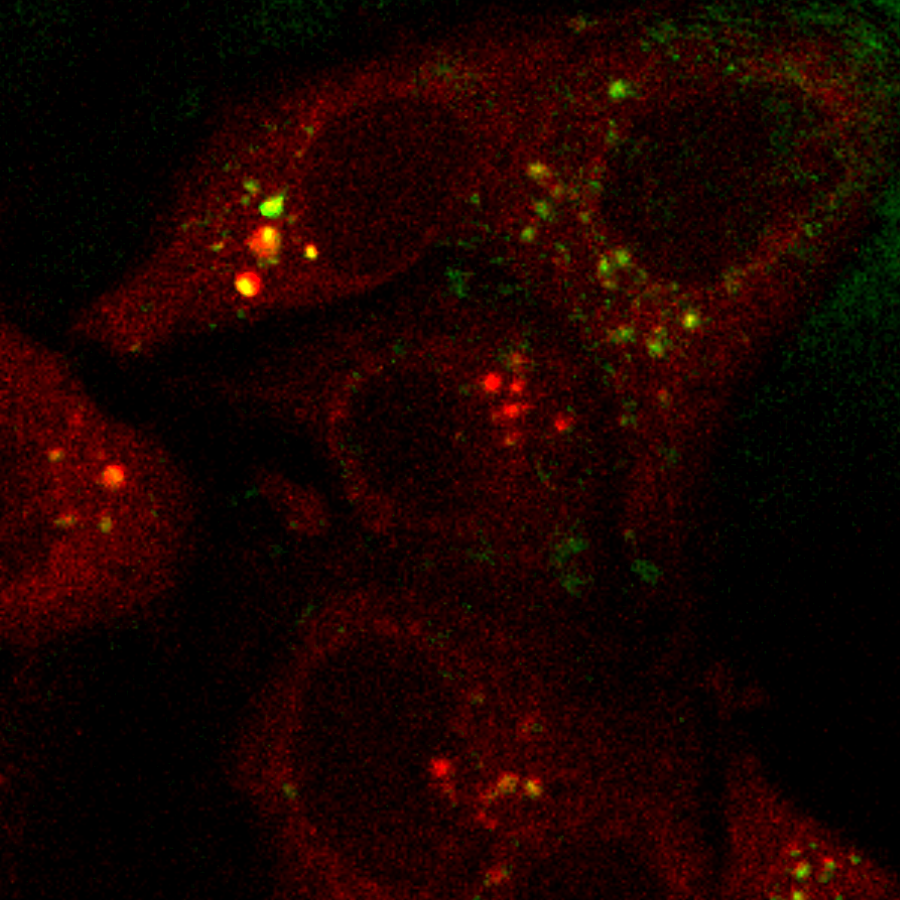

Supplement: Supplementary file 24 — Unmodified confocal images for Extended Data Fig. 3b,c,d. [file 42255_2025_1225_MOESM24_ESM.zip › Zuhra_Microscopy_Images_Extended_Fig3/Zuhra_Microscopy_Images_Extended_Fig3d/HOCl - LysoTracker/HepG2_Merge.tif]

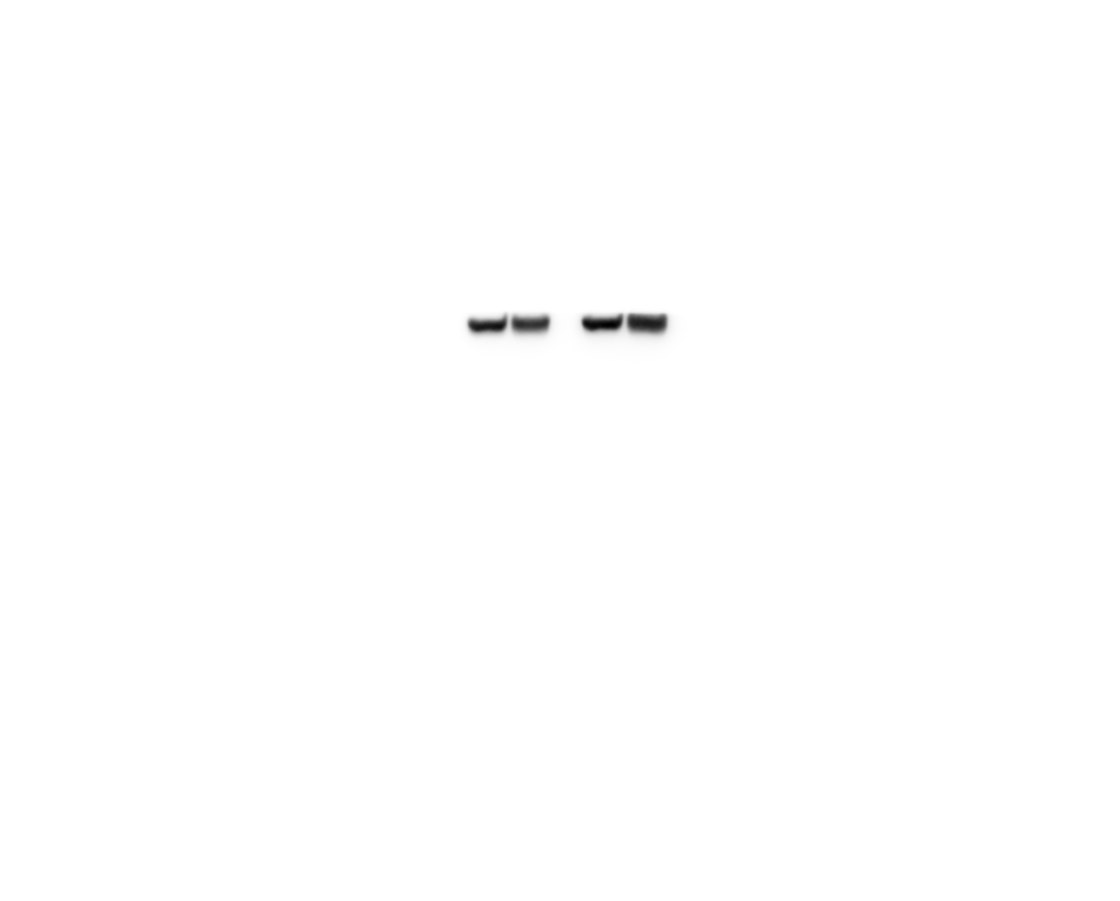

Supplement: Supplementary file 26 — Unprocessed western blots for Extended Data Fig. 4a,b. [file 42255_2025_1225_MOESM26_ESM.zip › Zuhra_WesternBlot_Extended_Fig4/Zuhra_WesternBlot_Extended_Fig4_a/Actin.tif]

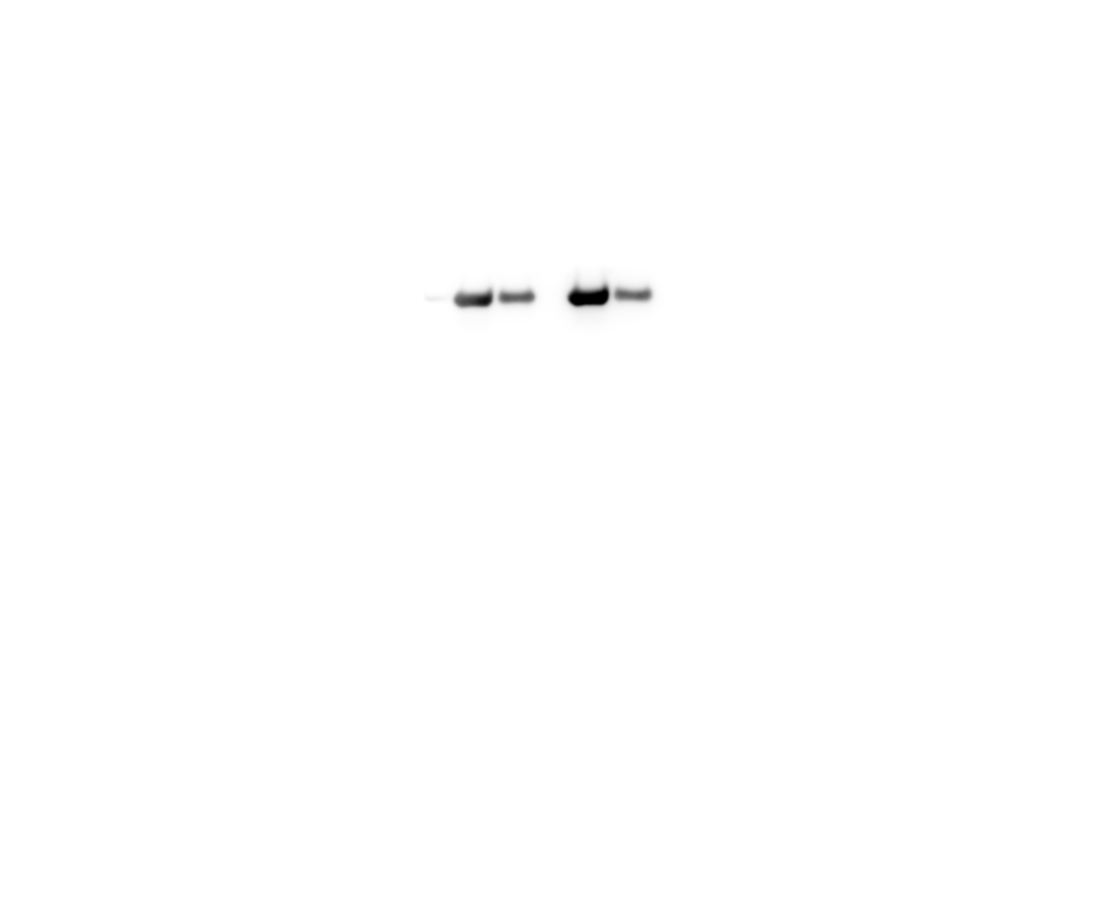

Supplement: Supplementary file 26 — Unprocessed western blots for Extended Data Fig. 4a,b. [file 42255_2025_1225_MOESM26_ESM.zip › Zuhra_WesternBlot_Extended_Fig4/Zuhra_WesternBlot_Extended_Fig4_a/GAPDH.tif]

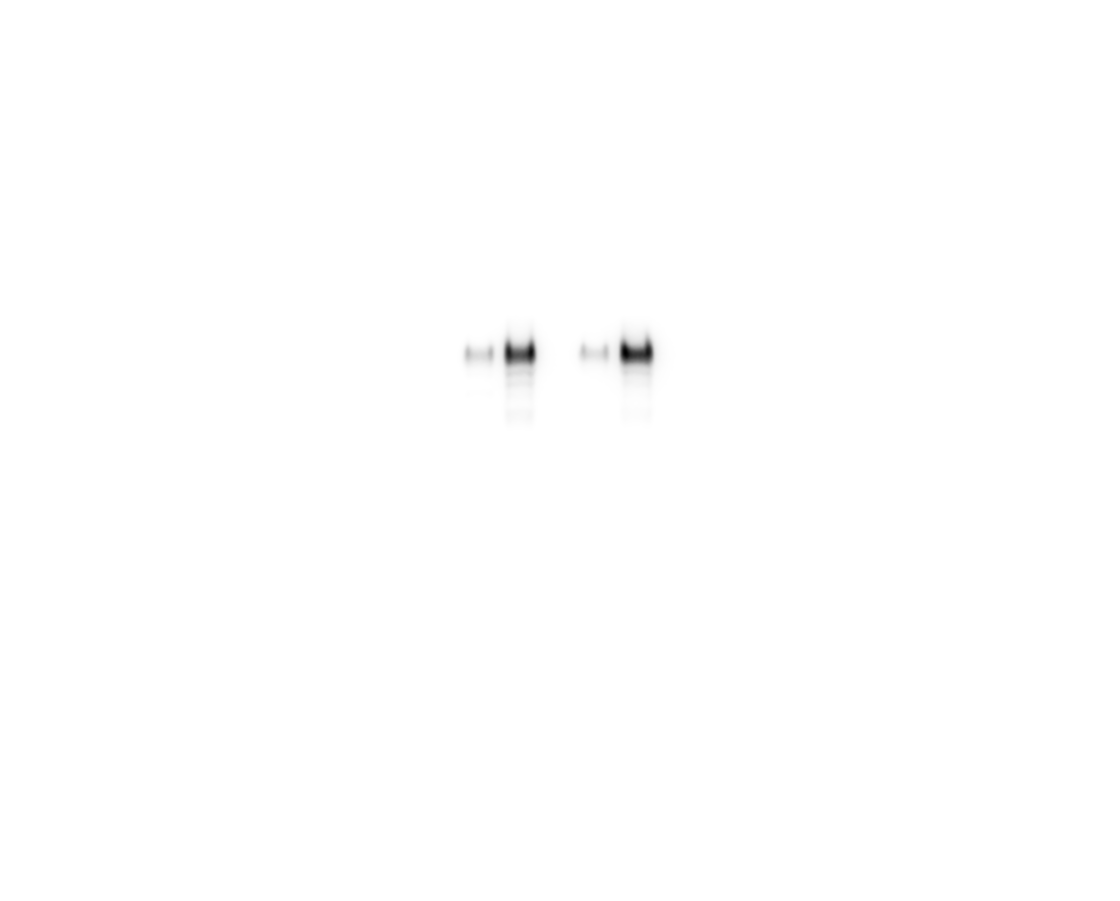

Supplement: Supplementary file 26 — Unprocessed western blots for Extended Data Fig. 4a,b. [file 42255_2025_1225_MOESM26_ESM.zip › Zuhra_WesternBlot_Extended_Fig4/Zuhra_WesternBlot_Extended_Fig4_a/LAMP1.tif]

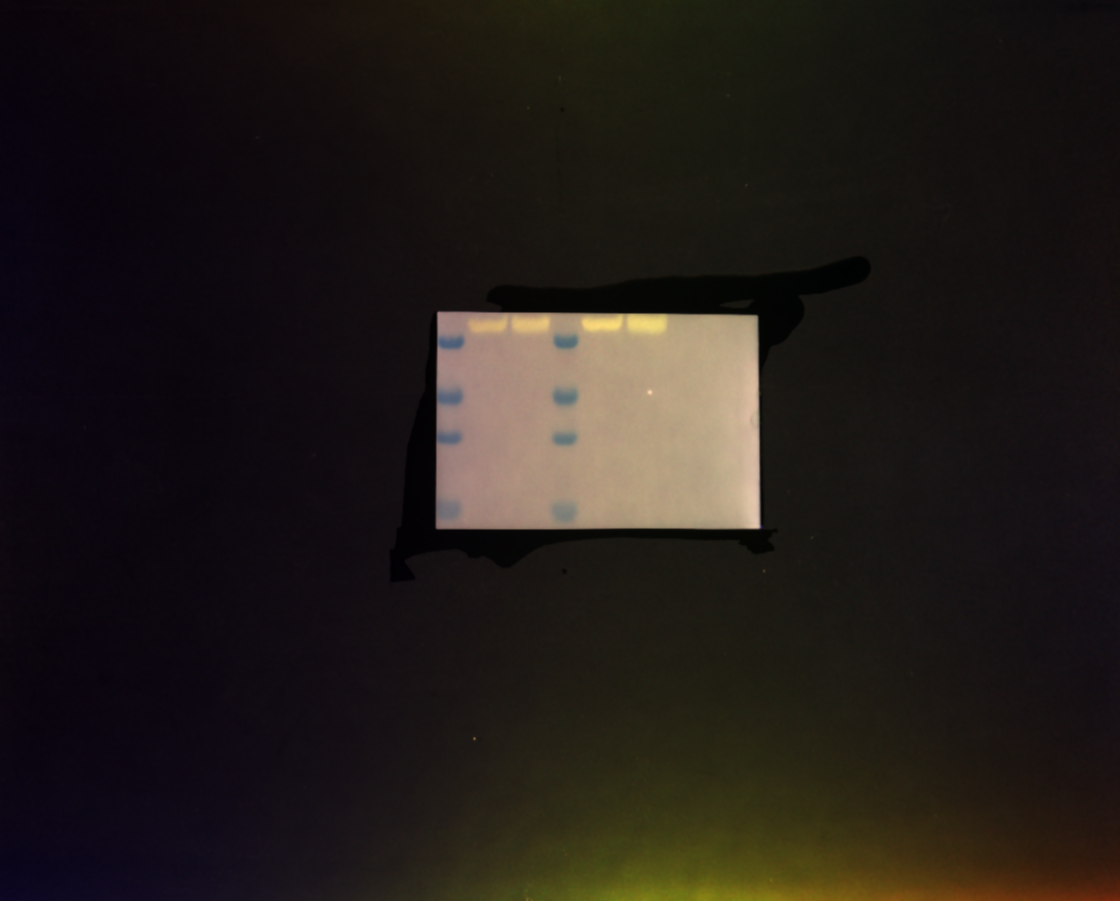

Supplement: Supplementary file 26 — Unprocessed western blots for Extended Data Fig. 4a,b. [file 42255_2025_1225_MOESM26_ESM.zip › Zuhra_WesternBlot_Extended_Fig4/Zuhra_WesternBlot_Extended_Fig4_a/Marker/Actin_marker.tif]

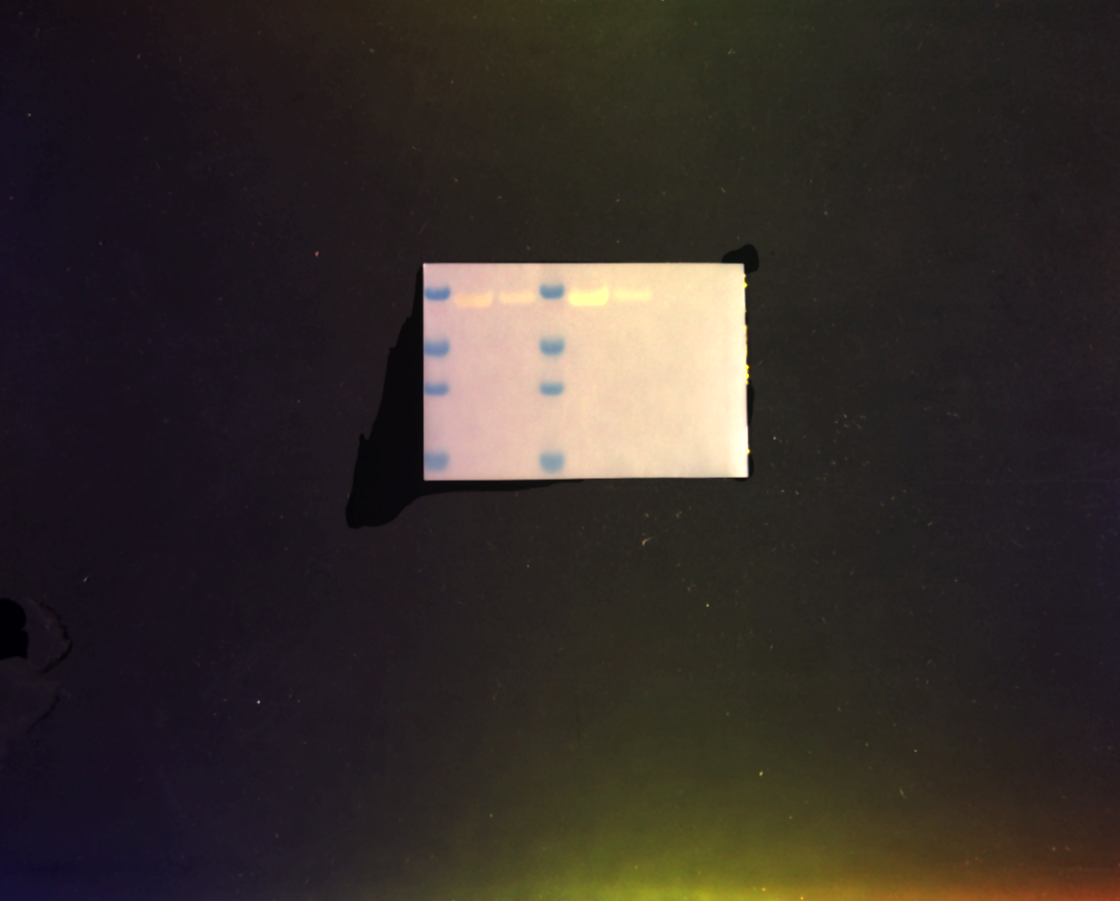

Supplement: Supplementary file 26 — Unprocessed western blots for Extended Data Fig. 4a,b. [file 42255_2025_1225_MOESM26_ESM.zip › Zuhra_WesternBlot_Extended_Fig4/Zuhra_WesternBlot_Extended_Fig4_a/Marker/GAPDH_marker.tif]

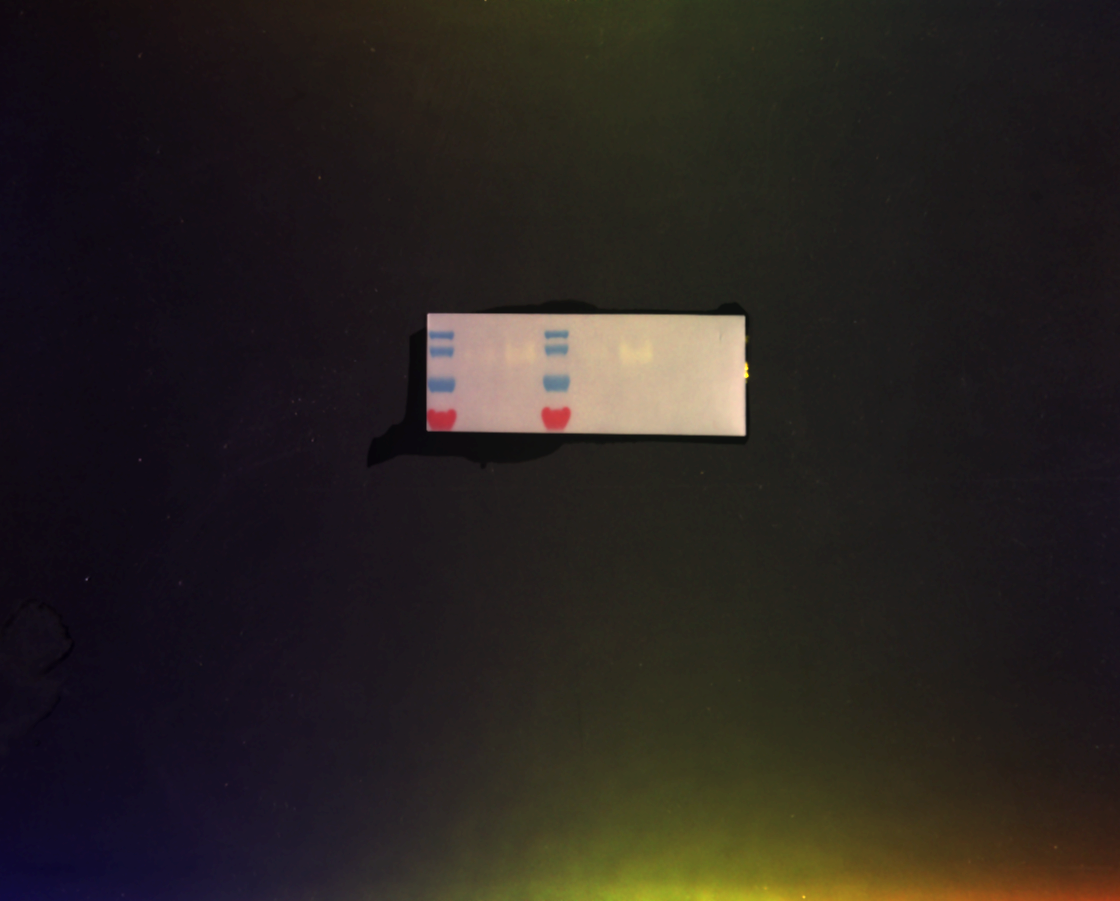

Supplement: Supplementary file 26 — Unprocessed western blots for Extended Data Fig. 4a,b. [file 42255_2025_1225_MOESM26_ESM.zip › Zuhra_WesternBlot_Extended_Fig4/Zuhra_WesternBlot_Extended_Fig4_a/Marker/LAMP1_marker.tif]

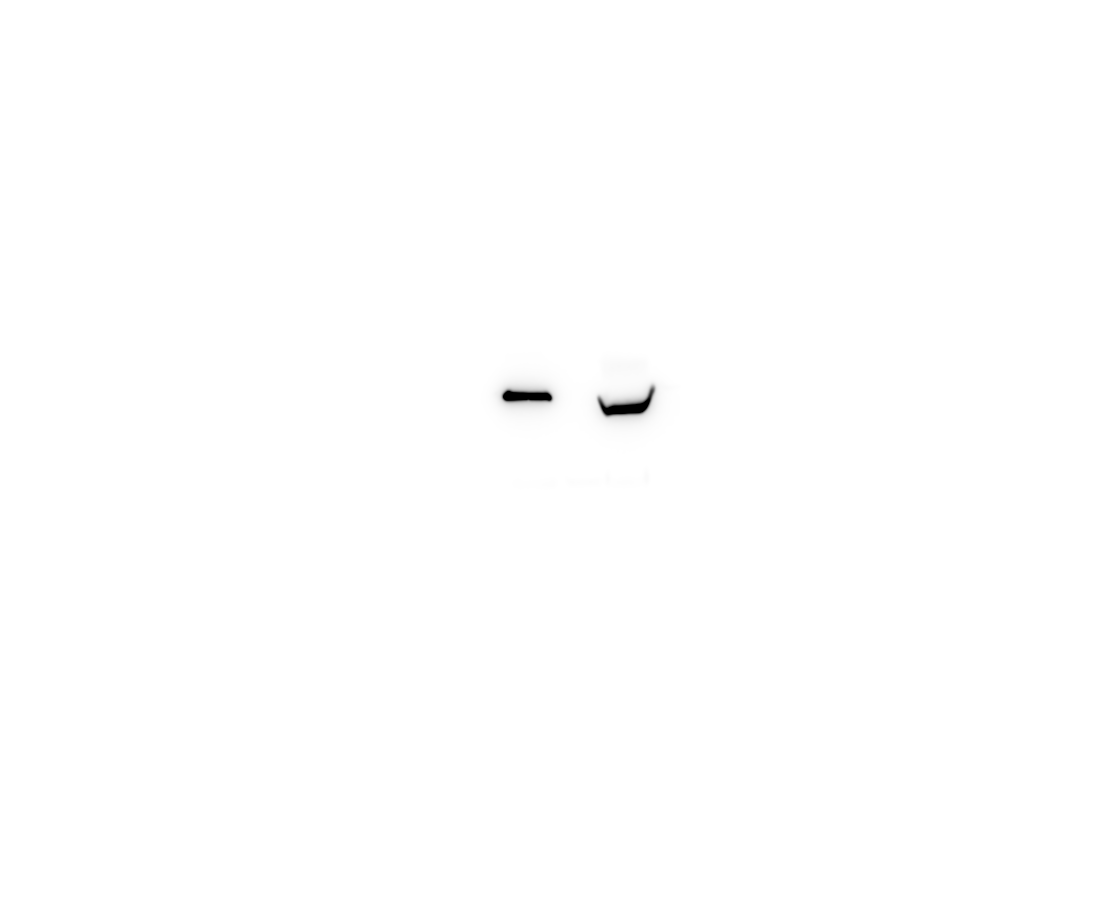

Supplement: Supplementary file 26 — Unprocessed western blots for Extended Data Fig. 4a,b. [file 42255_2025_1225_MOESM26_ESM.zip › Zuhra_WesternBlot_Extended_Fig4/Zuhra_WesternBlot_Extended_Fig4_b/Actin.tif]

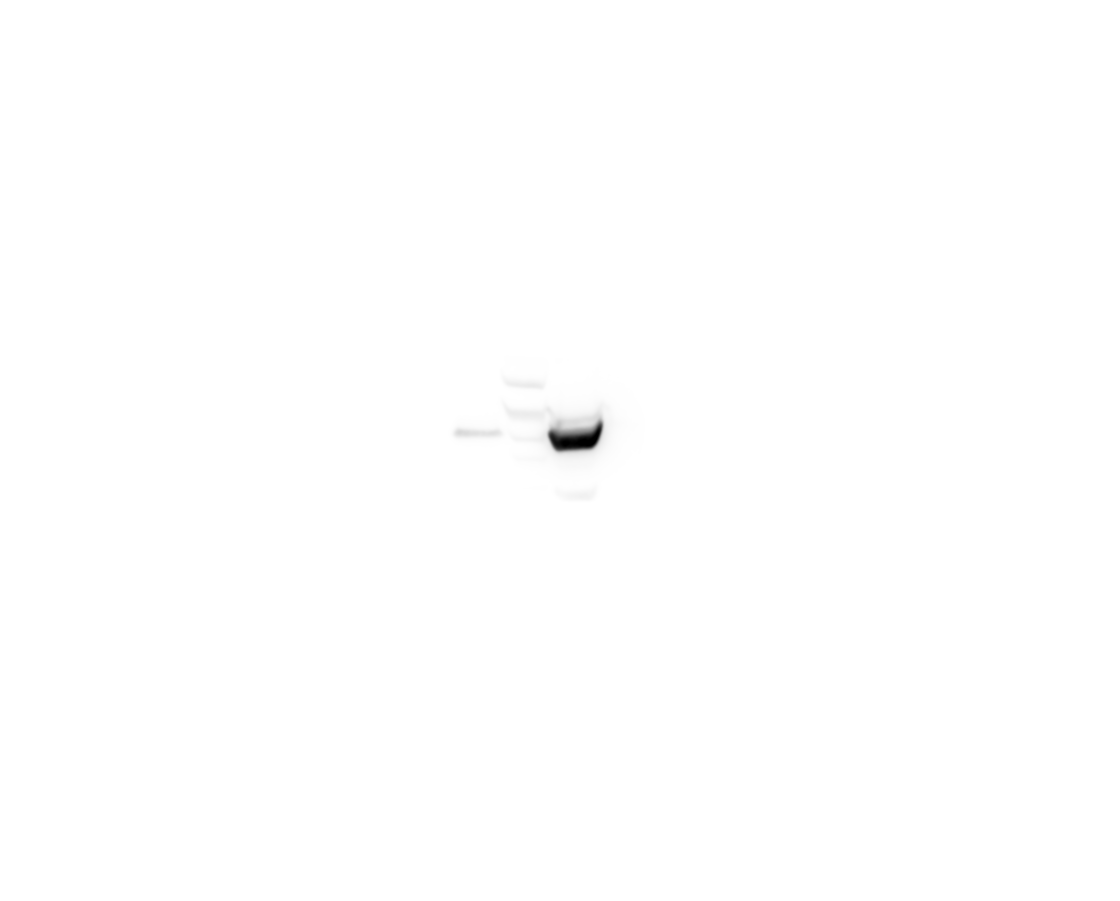

Supplement: Supplementary file 26 — Unprocessed western blots for Extended Data Fig. 4a,b. [file 42255_2025_1225_MOESM26_ESM.zip › Zuhra_WesternBlot_Extended_Fig4/Zuhra_WesternBlot_Extended_Fig4_b/GAPDH.tif]

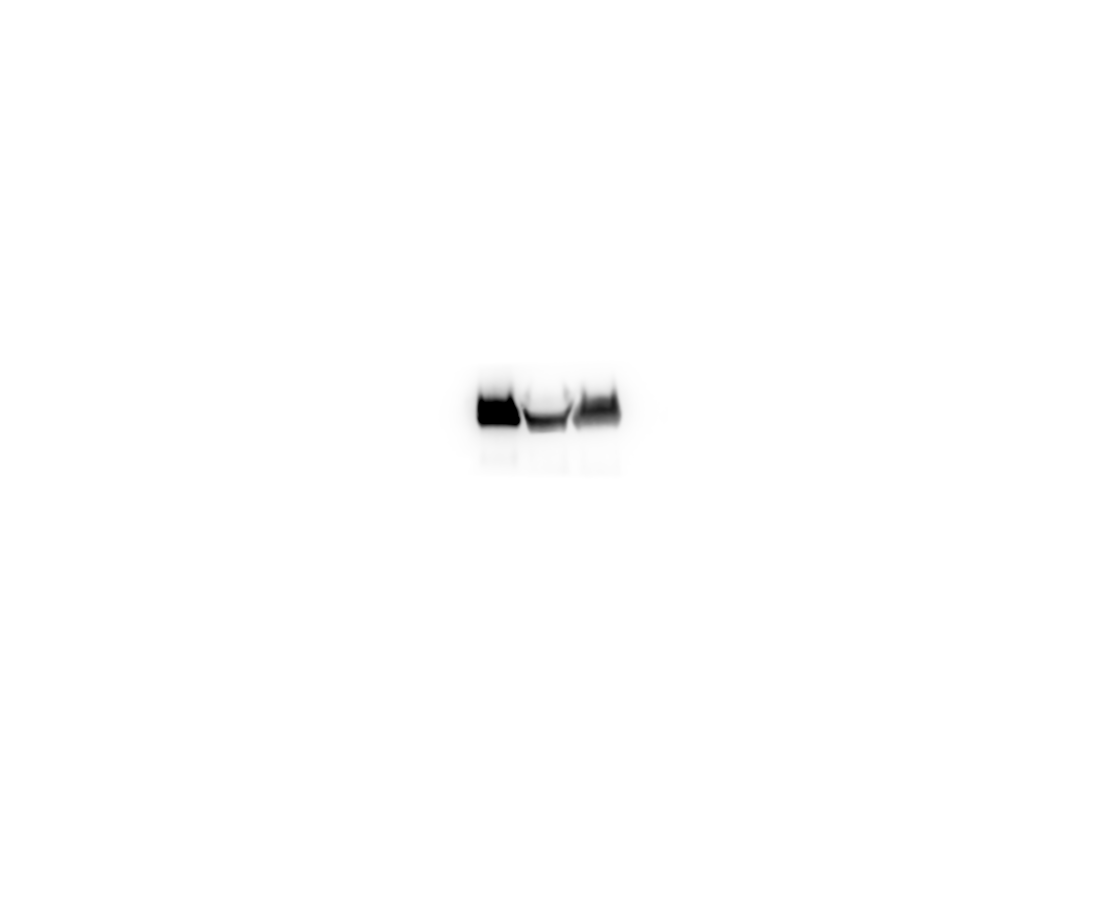

Supplement: Supplementary file 26 — Unprocessed western blots for Extended Data Fig. 4a,b. [file 42255_2025_1225_MOESM26_ESM.zip › Zuhra_WesternBlot_Extended_Fig4/Zuhra_WesternBlot_Extended_Fig4_b/LAMP1.tif]

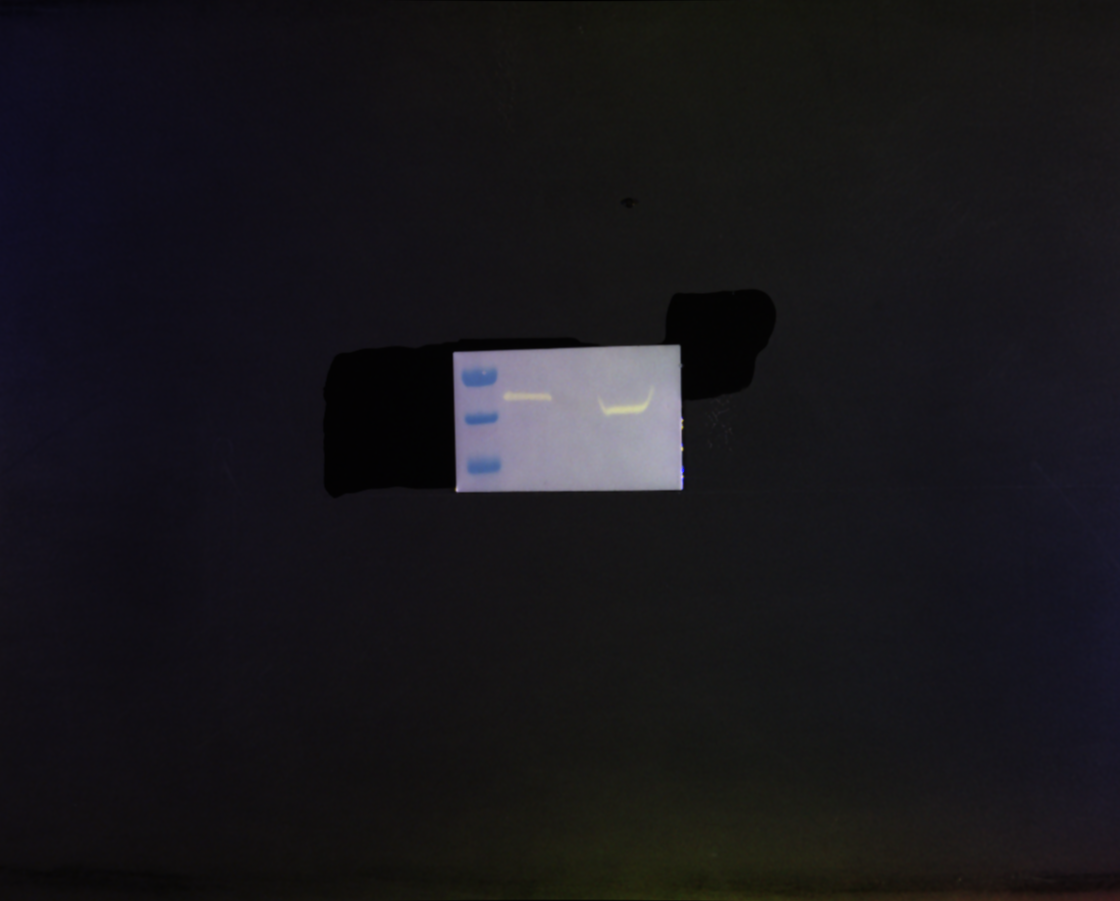

Supplement: Supplementary file 26 — Unprocessed western blots for Extended Data Fig. 4a,b. [file 42255_2025_1225_MOESM26_ESM.zip › Zuhra_WesternBlot_Extended_Fig4/Zuhra_WesternBlot_Extended_Fig4_b/Marker/Actin_marker.tif]

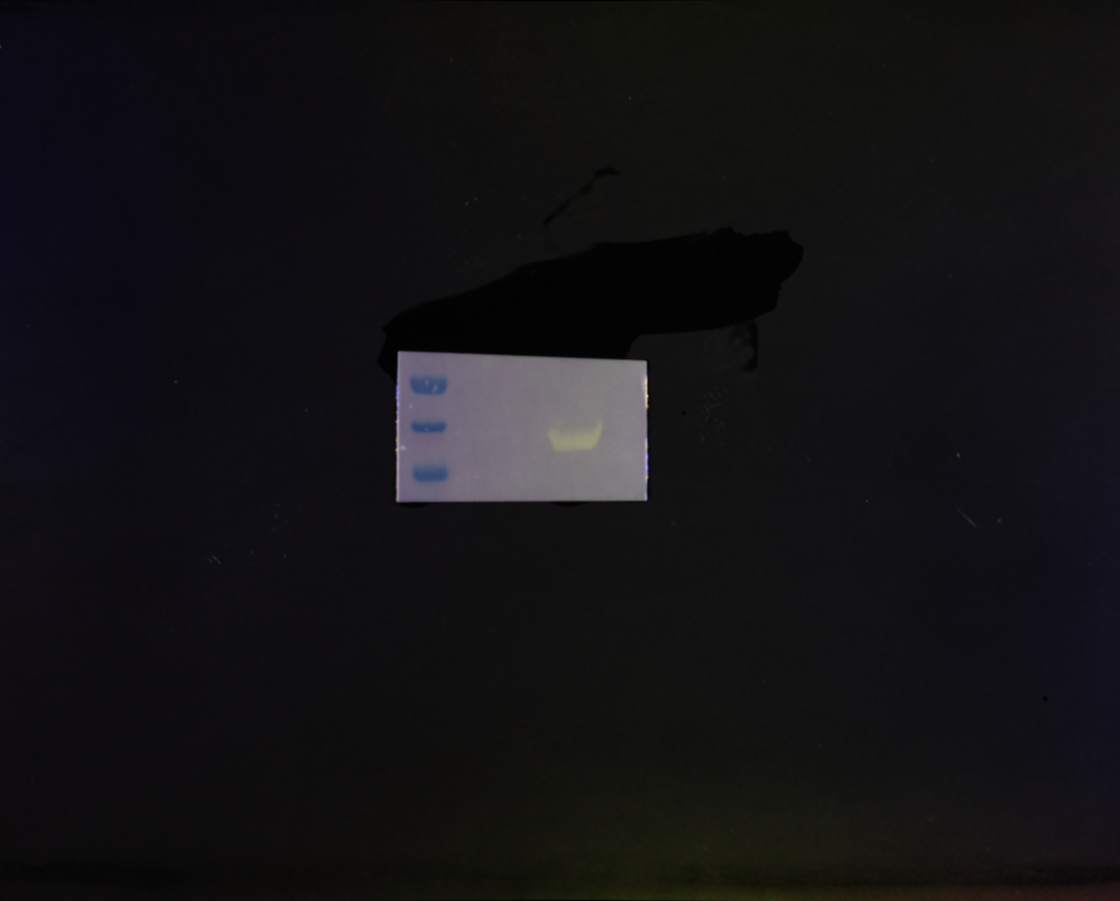

Supplement: Supplementary file 26 — Unprocessed western blots for Extended Data Fig. 4a,b. [file 42255_2025_1225_MOESM26_ESM.zip › Zuhra_WesternBlot_Extended_Fig4/Zuhra_WesternBlot_Extended_Fig4_b/Marker/GAPDH_marker.tif]

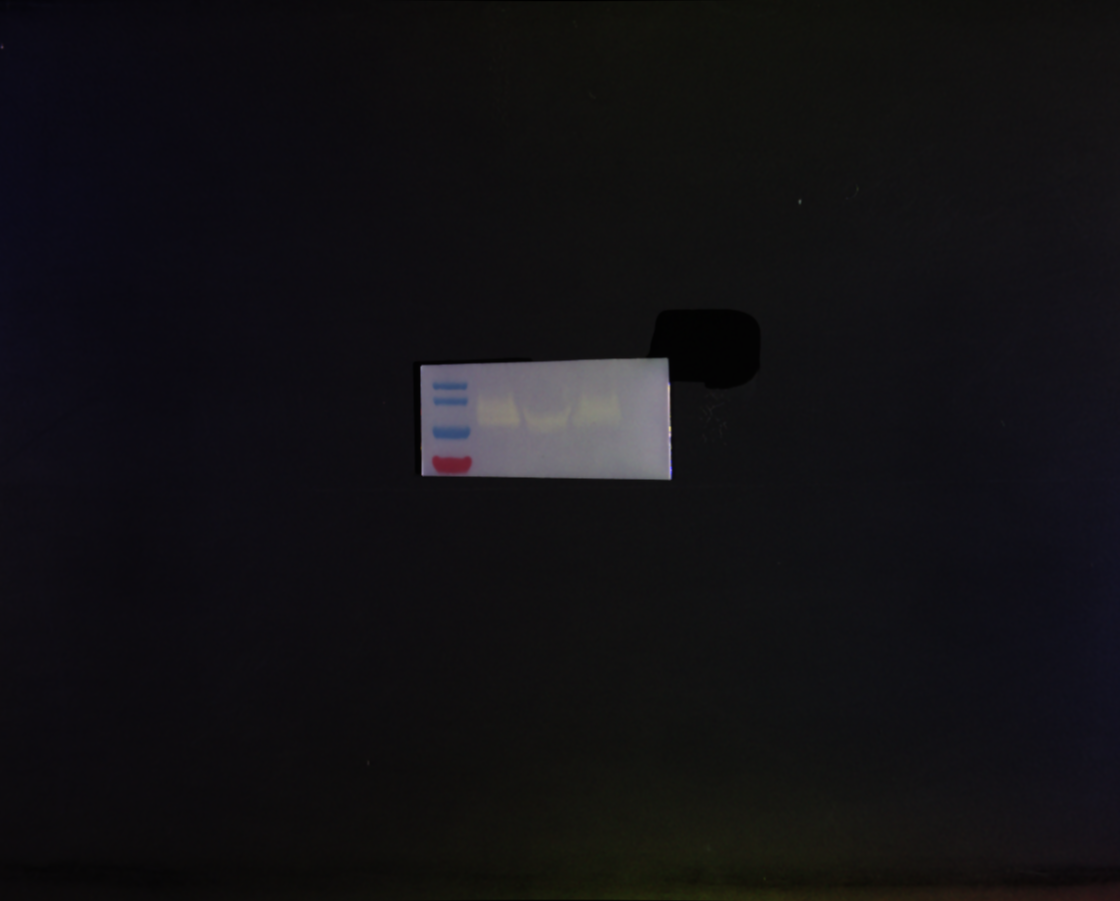

Supplement: Supplementary file 26 — Unprocessed western blots for Extended Data Fig. 4a,b. [file 42255_2025_1225_MOESM26_ESM.zip › Zuhra_WesternBlot_Extended_Fig4/Zuhra_WesternBlot_Extended_Fig4_b/Marker/LAMP1_marker.tif]

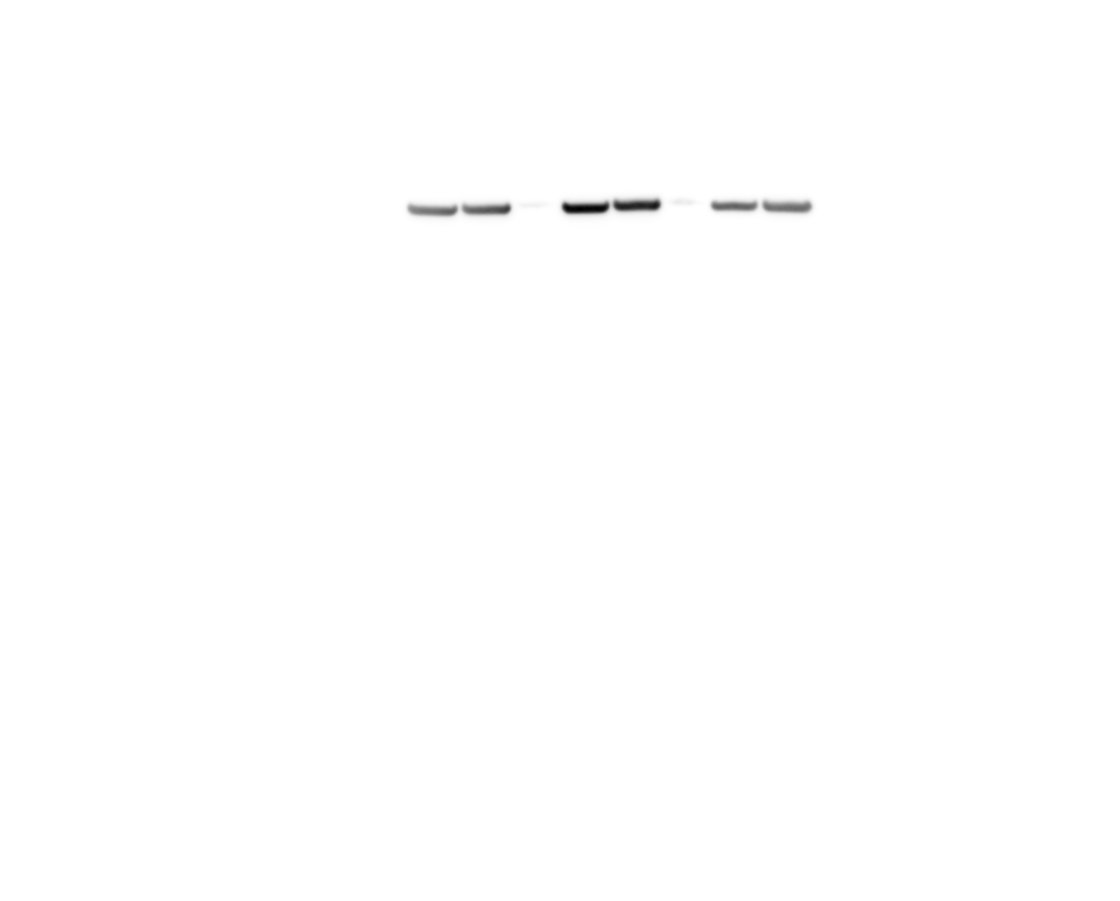

Supplement: Supplementary file 26 — Unprocessed western blots for Extended Data Fig. 4a,b. [file 42255_2025_1225_MOESM26_ESM.zip › Zuhra_WesternBlot_Extended_Fig4/Zuhra_WesternBlot_Extended_Fig4_c/CAT/FigE4g_CAT_Experiment1-2-3_actin.jpg]

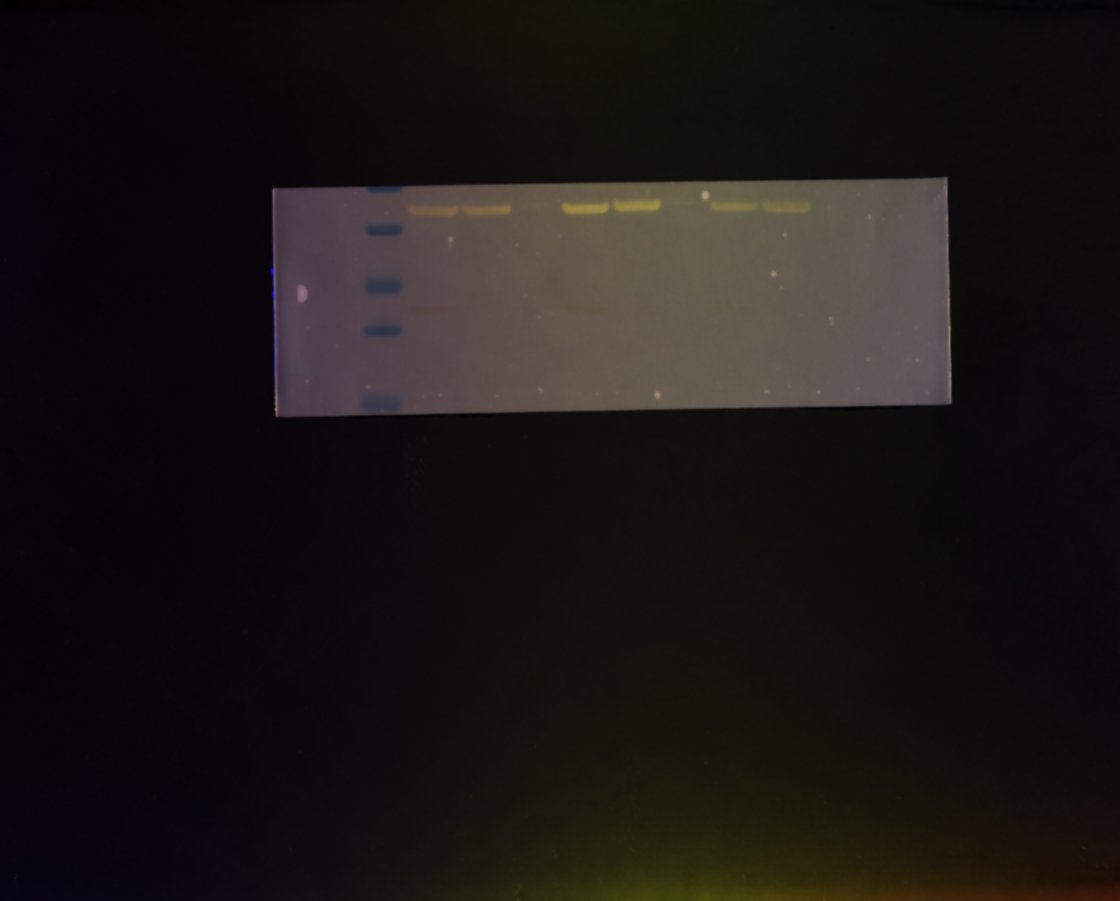

Supplement: Supplementary file 26 — Unprocessed western blots for Extended Data Fig. 4a,b. [file 42255_2025_1225_MOESM26_ESM.zip › Zuhra_WesternBlot_Extended_Fig4/Zuhra_WesternBlot_Extended_Fig4_c/CAT/FigE4g_CAT_Experiment1-2-3_actin_marker.jpg]

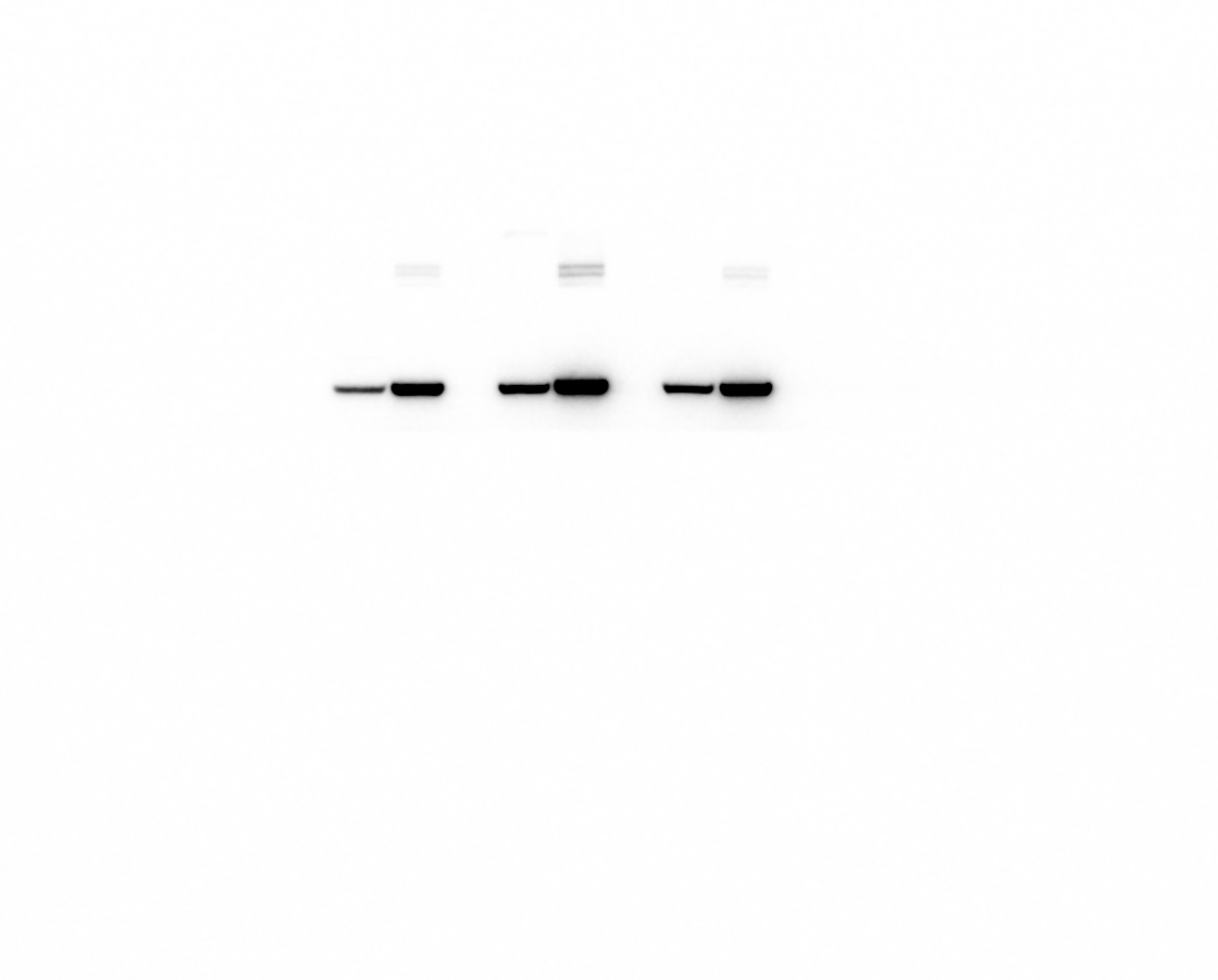

Supplement: Supplementary file 26 — Unprocessed western blots for Extended Data Fig. 4a,b. [file 42255_2025_1225_MOESM26_ESM.zip › Zuhra_WesternBlot_Extended_Fig4/Zuhra_WesternBlot_Extended_Fig4_c/CAT/FigE4g_CAT_Experiment1-2-3_CAT.jpg]

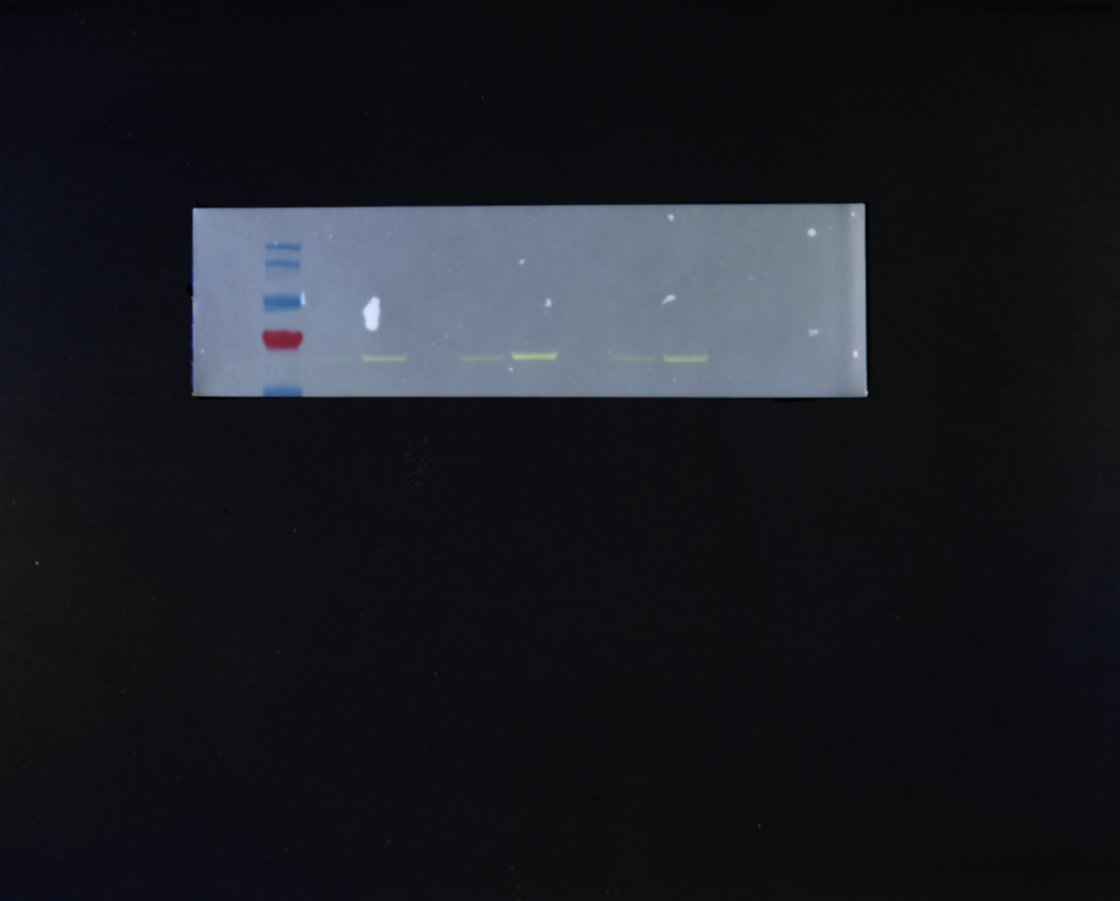

Supplement: Supplementary file 26 — Unprocessed western blots for Extended Data Fig. 4a,b. [file 42255_2025_1225_MOESM26_ESM.zip › Zuhra_WesternBlot_Extended_Fig4/Zuhra_WesternBlot_Extended_Fig4_c/CAT/FigE4g_CAT_Experiment1-2-3_CAT_marker.tif]

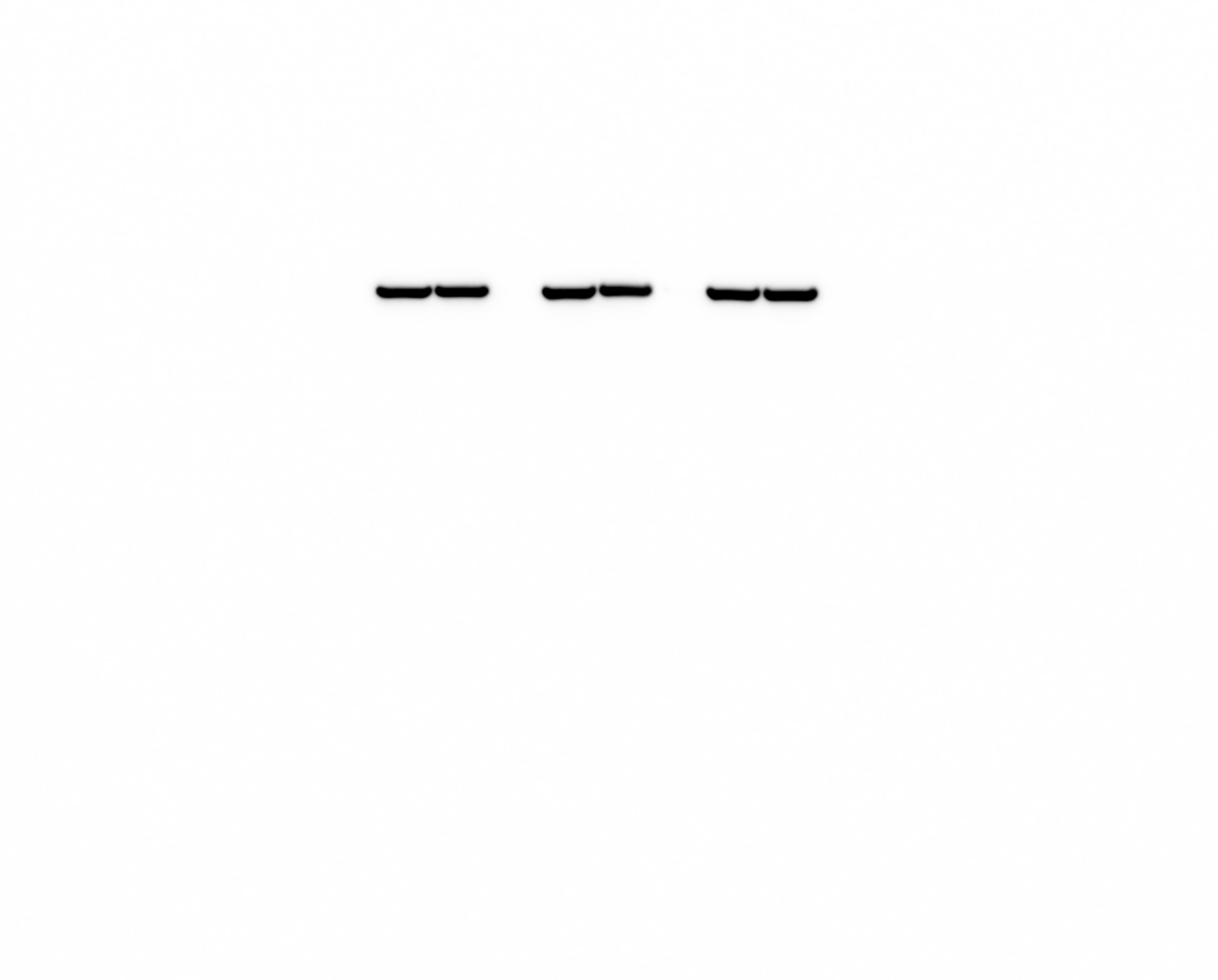

Supplement: Supplementary file 26 — Unprocessed western blots for Extended Data Fig. 4a,b. [file 42255_2025_1225_MOESM26_ESM.zip › Zuhra_WesternBlot_Extended_Fig4/Zuhra_WesternBlot_Extended_Fig4_c/CAT/FigE4g_CAT_Experiment4-5-6_actin.jpg]

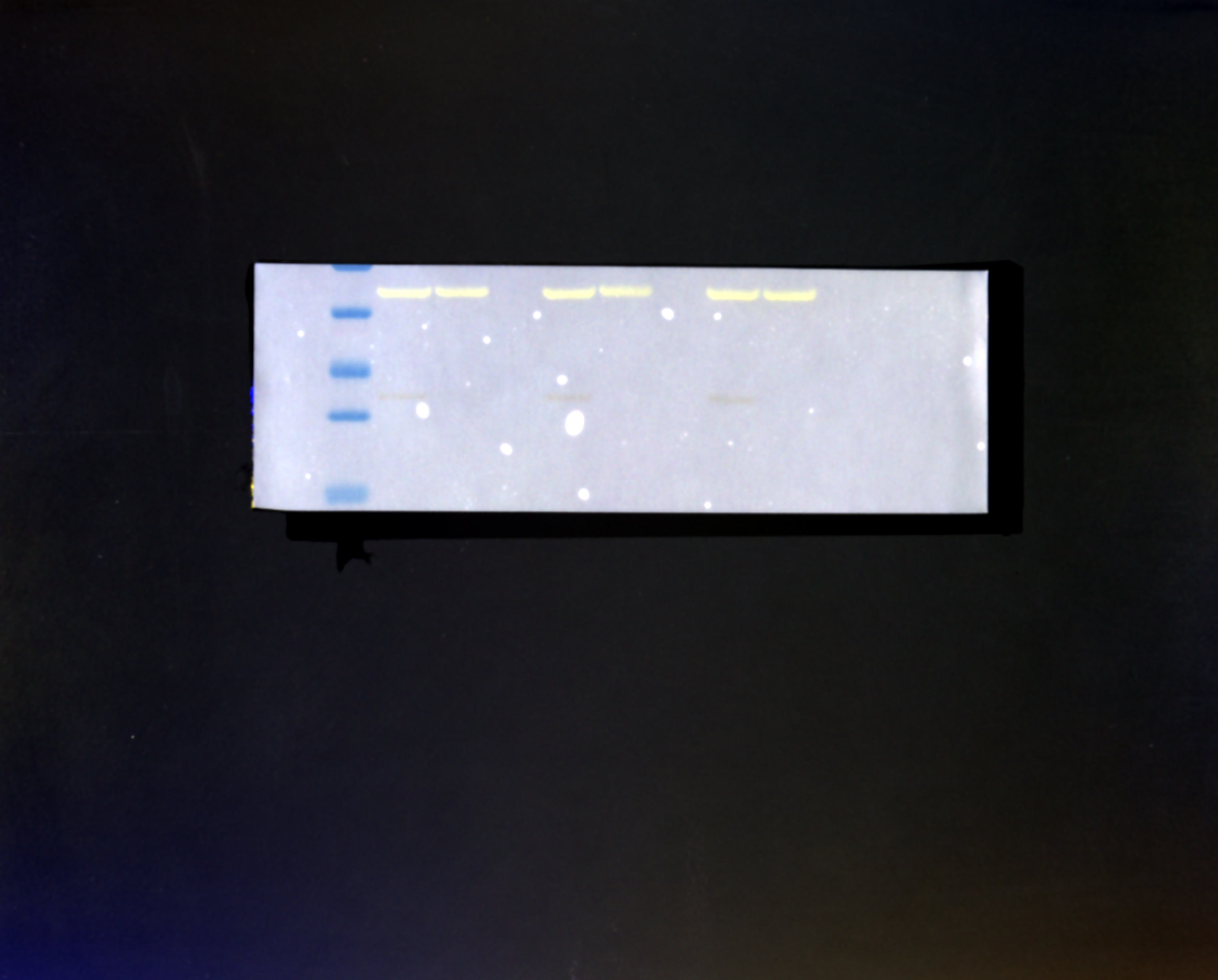

Supplement: Supplementary file 26 — Unprocessed western blots for Extended Data Fig. 4a,b. [file 42255_2025_1225_MOESM26_ESM.zip › Zuhra_WesternBlot_Extended_Fig4/Zuhra_WesternBlot_Extended_Fig4_c/CAT/FigE4g_CAT_Experiment4-5-6_actin_marker.jpg]

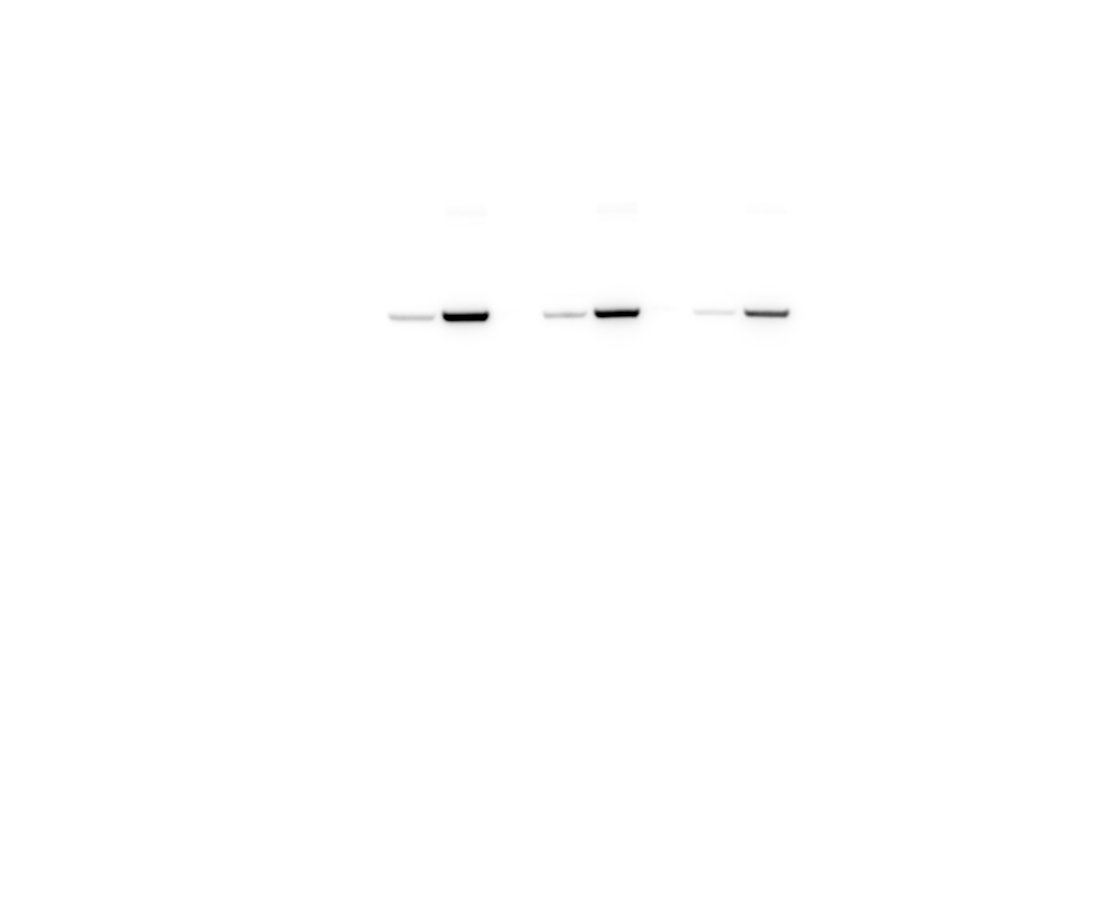

Supplement: Supplementary file 26 — Unprocessed western blots for Extended Data Fig. 4a,b. [file 42255_2025_1225_MOESM26_ESM.zip › Zuhra_WesternBlot_Extended_Fig4/Zuhra_WesternBlot_Extended_Fig4_c/CAT/FigE4g_CAT_Experiment4-5-6_CAT.jpg]

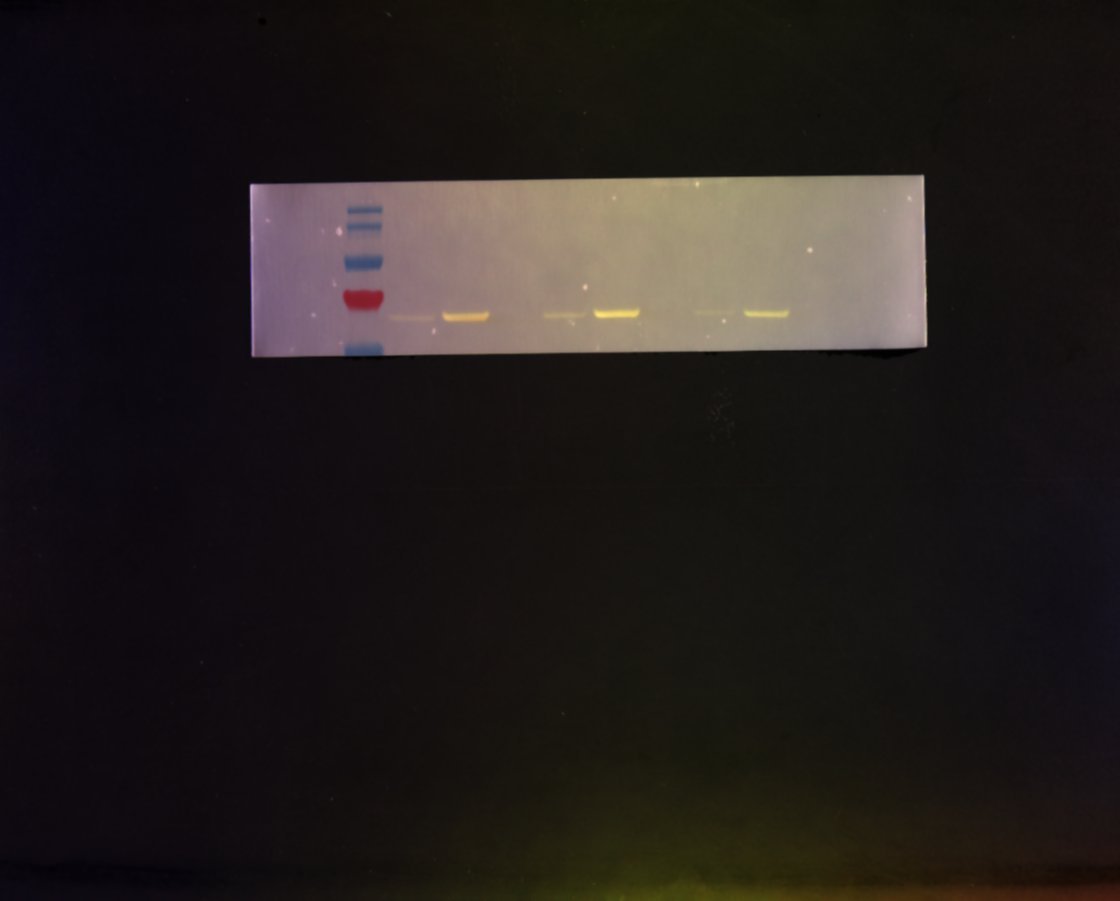

Supplement: Supplementary file 26 — Unprocessed western blots for Extended Data Fig. 4a,b. [file 42255_2025_1225_MOESM26_ESM.zip › Zuhra_WesternBlot_Extended_Fig4/Zuhra_WesternBlot_Extended_Fig4_c/CAT/FigE4g_CAT_Experiment4-5-6_CAT_marker.tif]

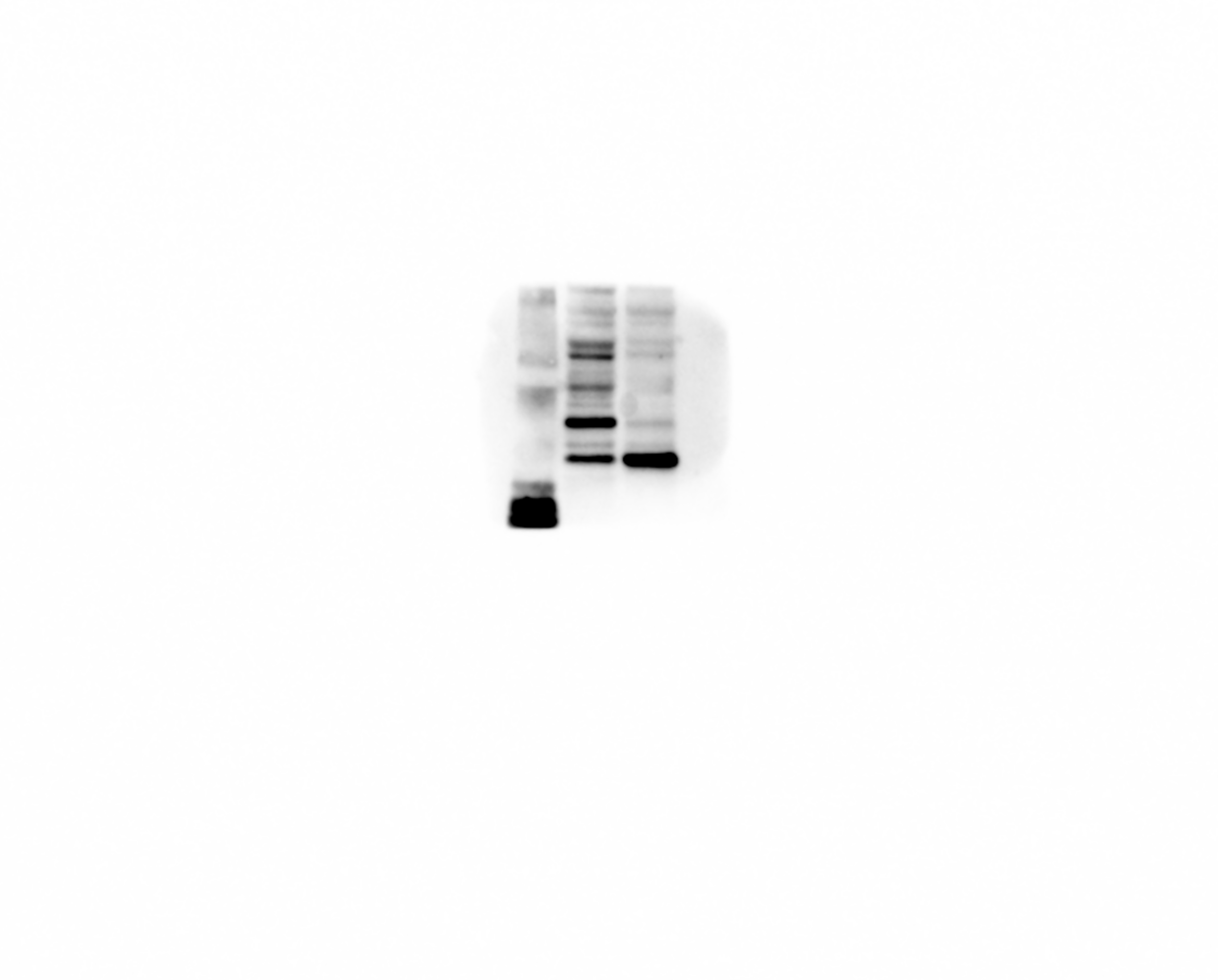

Supplement: Supplementary file 26 — Unprocessed western blots for Extended Data Fig. 4a,b. [file 42255_2025_1225_MOESM26_ESM.zip › Zuhra_WesternBlot_Extended_Fig4/Zuhra_WesternBlot_Extended_Fig4_c/GSTA1/FigE4g_GSTA1_Experiment1_GSTA1.jpg]

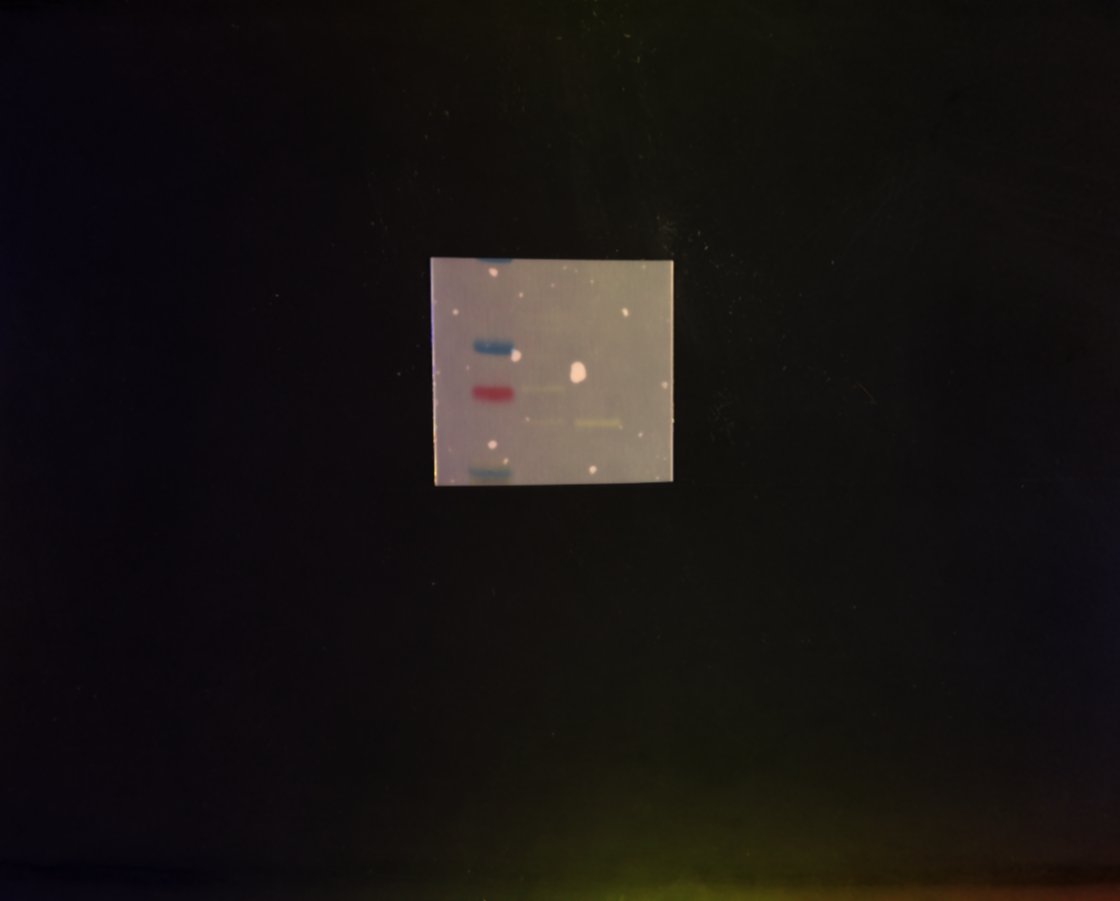

Supplement: Supplementary file 26 — Unprocessed western blots for Extended Data Fig. 4a,b. [file 42255_2025_1225_MOESM26_ESM.zip › Zuhra_WesternBlot_Extended_Fig4/Zuhra_WesternBlot_Extended_Fig4_c/GSTA1/FigE4g_GSTA1_Experiment1_GSTA1_marker.jpg]

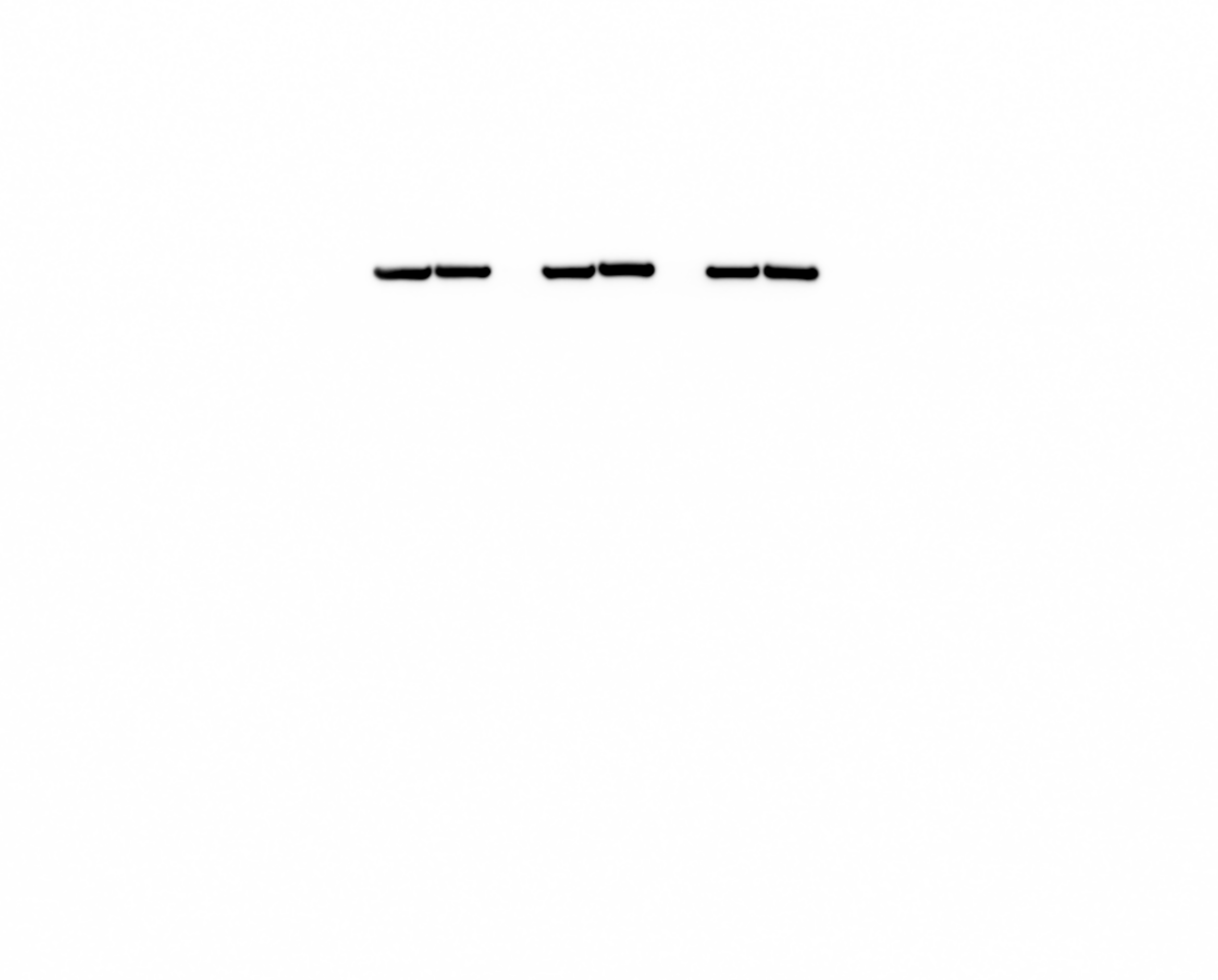

Supplement: Supplementary file 26 — Unprocessed western blots for Extended Data Fig. 4a,b. [file 42255_2025_1225_MOESM26_ESM.zip › Zuhra_WesternBlot_Extended_Fig4/Zuhra_WesternBlot_Extended_Fig4_c/GSTA1/FigE4g_GSTA1_Experiment2-3-4_actin.jpg]

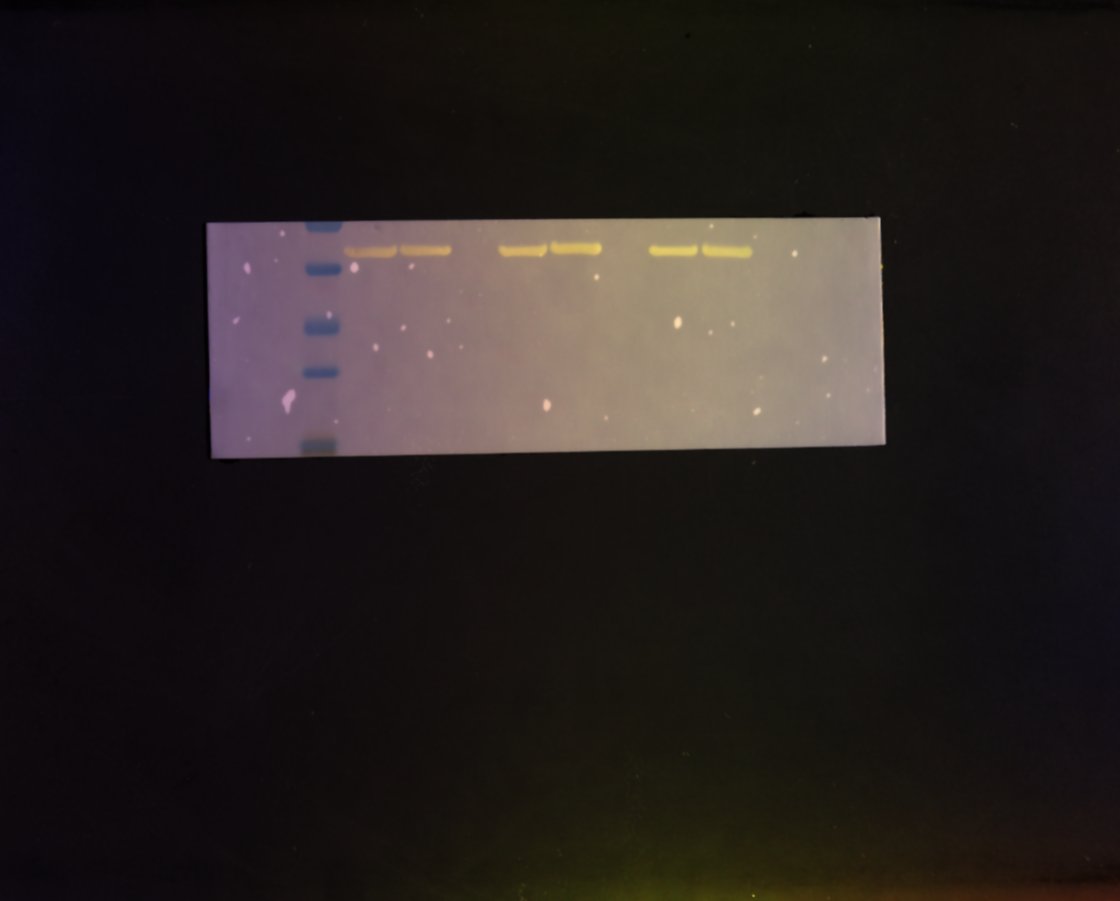

Supplement: Supplementary file 26 — Unprocessed western blots for Extended Data Fig. 4a,b. [file 42255_2025_1225_MOESM26_ESM.zip › Zuhra_WesternBlot_Extended_Fig4/Zuhra_WesternBlot_Extended_Fig4_c/GSTA1/FigE4g_GSTA1_Experiment2-3-4_actin_marker.jpg]

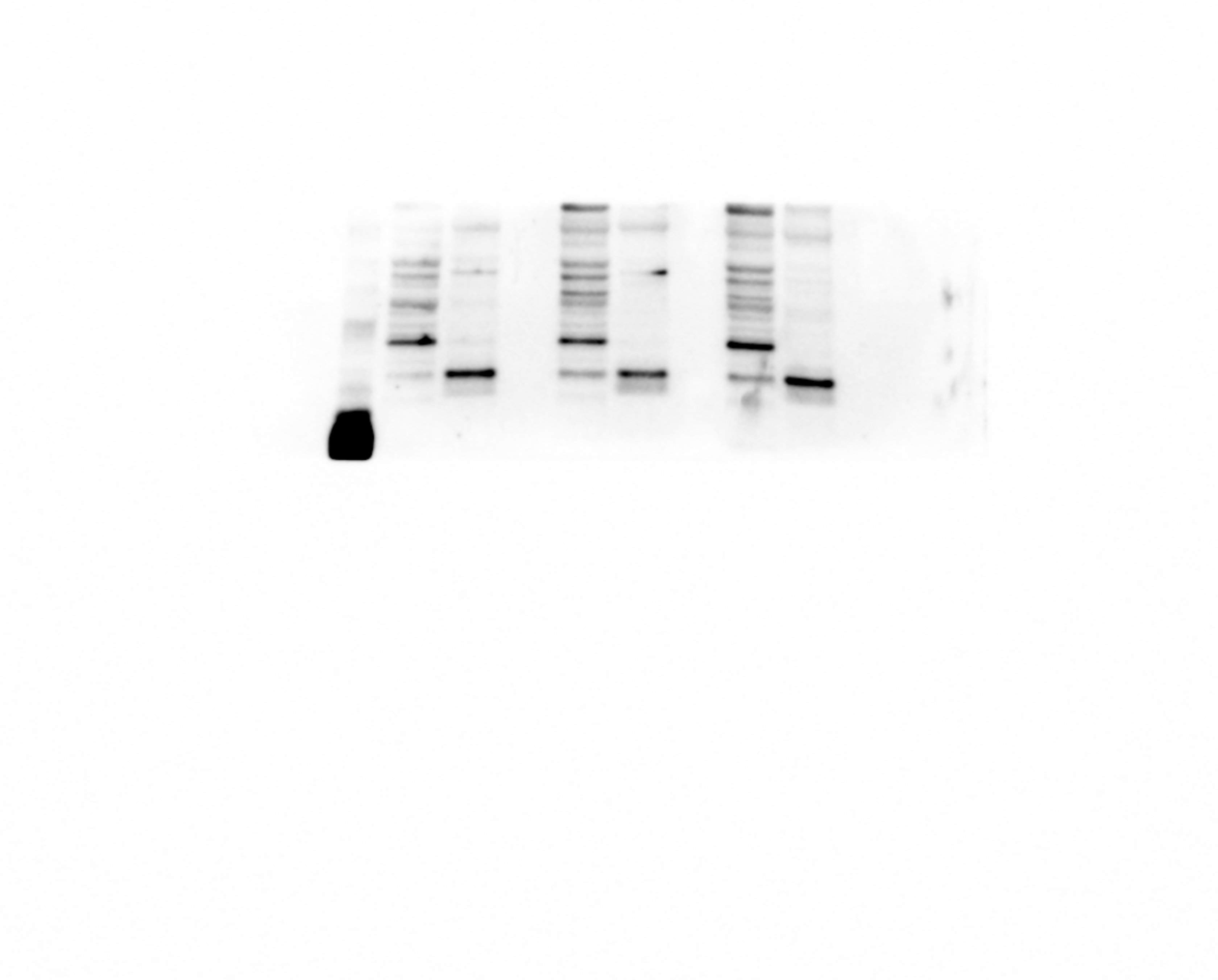

Supplement: Supplementary file 26 — Unprocessed western blots for Extended Data Fig. 4a,b. [file 42255_2025_1225_MOESM26_ESM.zip › Zuhra_WesternBlot_Extended_Fig4/Zuhra_WesternBlot_Extended_Fig4_c/GSTA1/FigE4g_GSTA1_Experiment2-3-4_GSTA1.jpg]

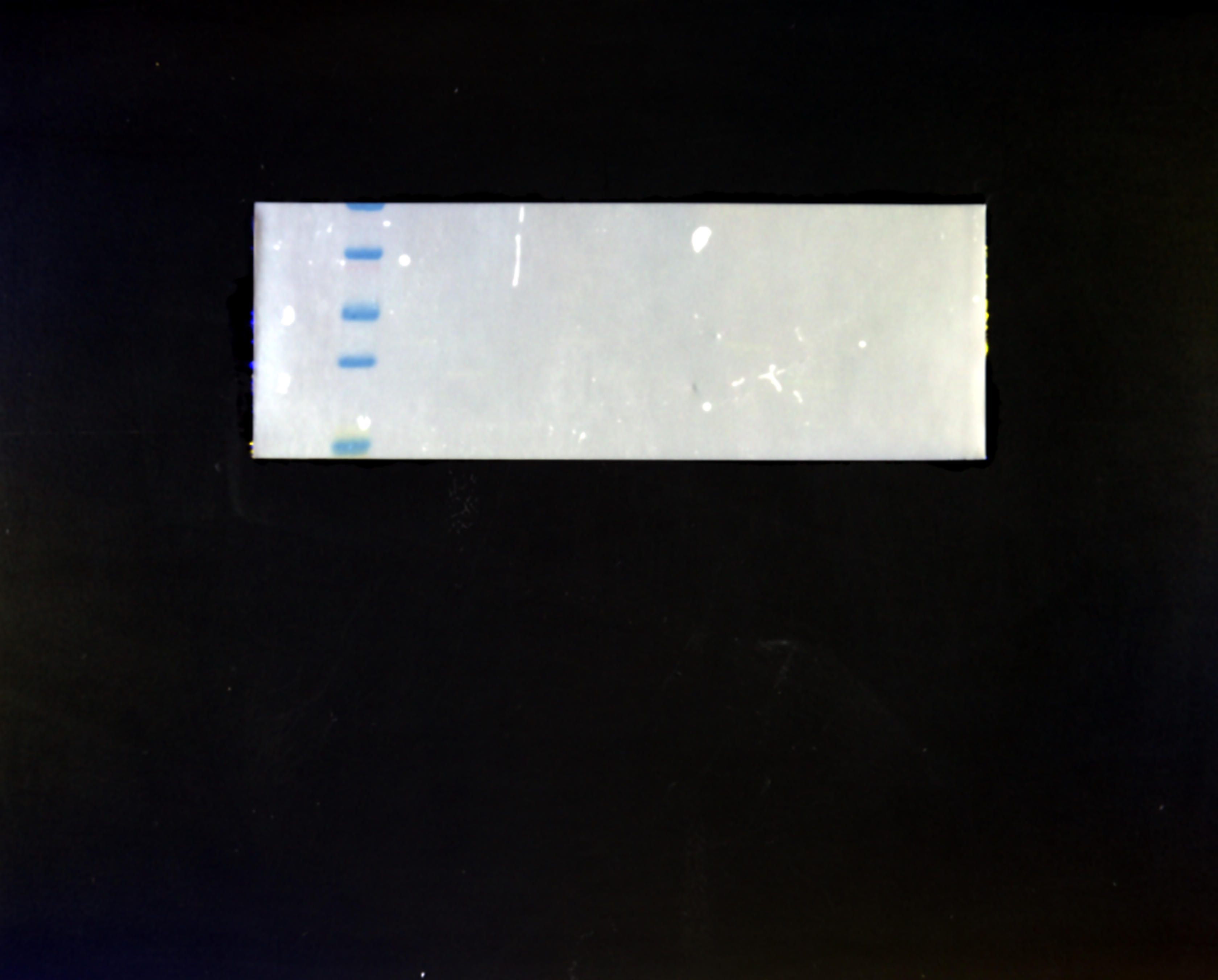

Supplement: Supplementary file 26 — Unprocessed western blots for Extended Data Fig. 4a,b. [file 42255_2025_1225_MOESM26_ESM.zip › Zuhra_WesternBlot_Extended_Fig4/Zuhra_WesternBlot_Extended_Fig4_c/GSTA1/FigE4g_GSTA1_Experiment2-3-4_GSTA1_marker.jpg]

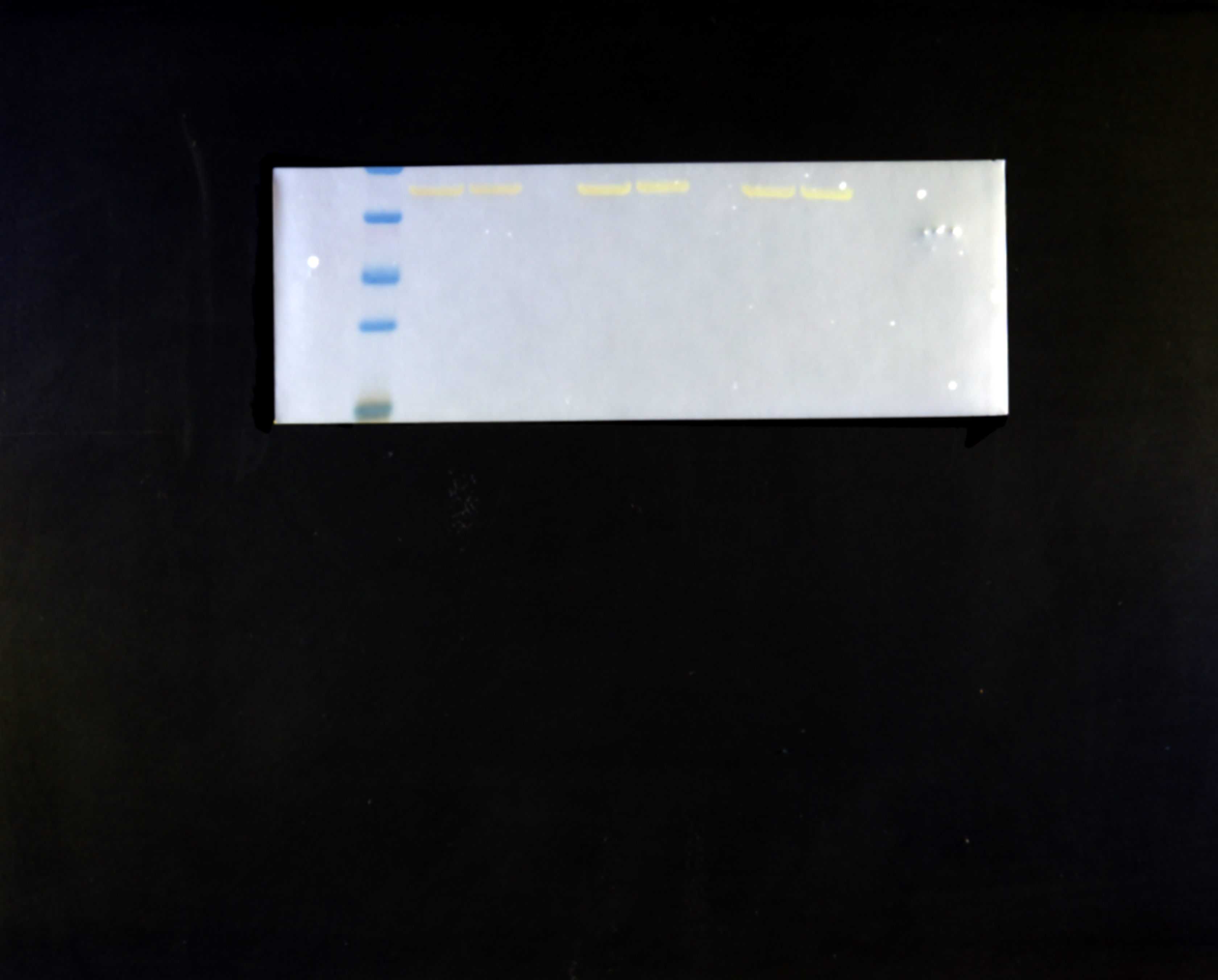

Supplement: Supplementary file 26 — Unprocessed western blots for Extended Data Fig. 4a,b. [file 42255_2025_1225_MOESM26_ESM.zip › Zuhra_WesternBlot_Extended_Fig4/Zuhra_WesternBlot_Extended_Fig4_c/GSTA1/FigE4g_GSTA1_Experiment5-6-7_actin_marker.jpg]

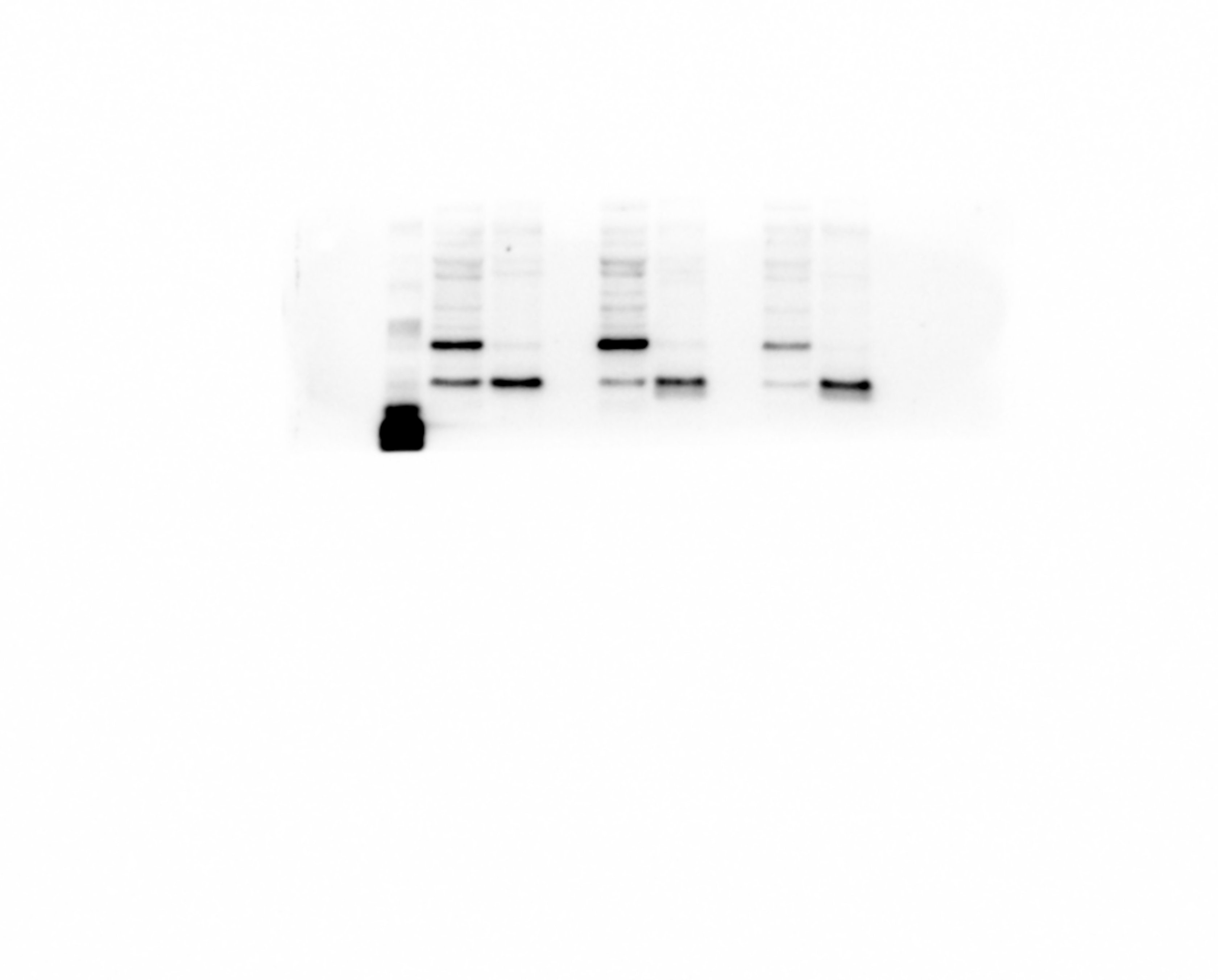

Supplement: Supplementary file 26 — Unprocessed western blots for Extended Data Fig. 4a,b. [file 42255_2025_1225_MOESM26_ESM.zip › Zuhra_WesternBlot_Extended_Fig4/Zuhra_WesternBlot_Extended_Fig4_c/GSTA1/FigE4g_GSTA1_Experiment5-6-7_GSTA1.jpg]

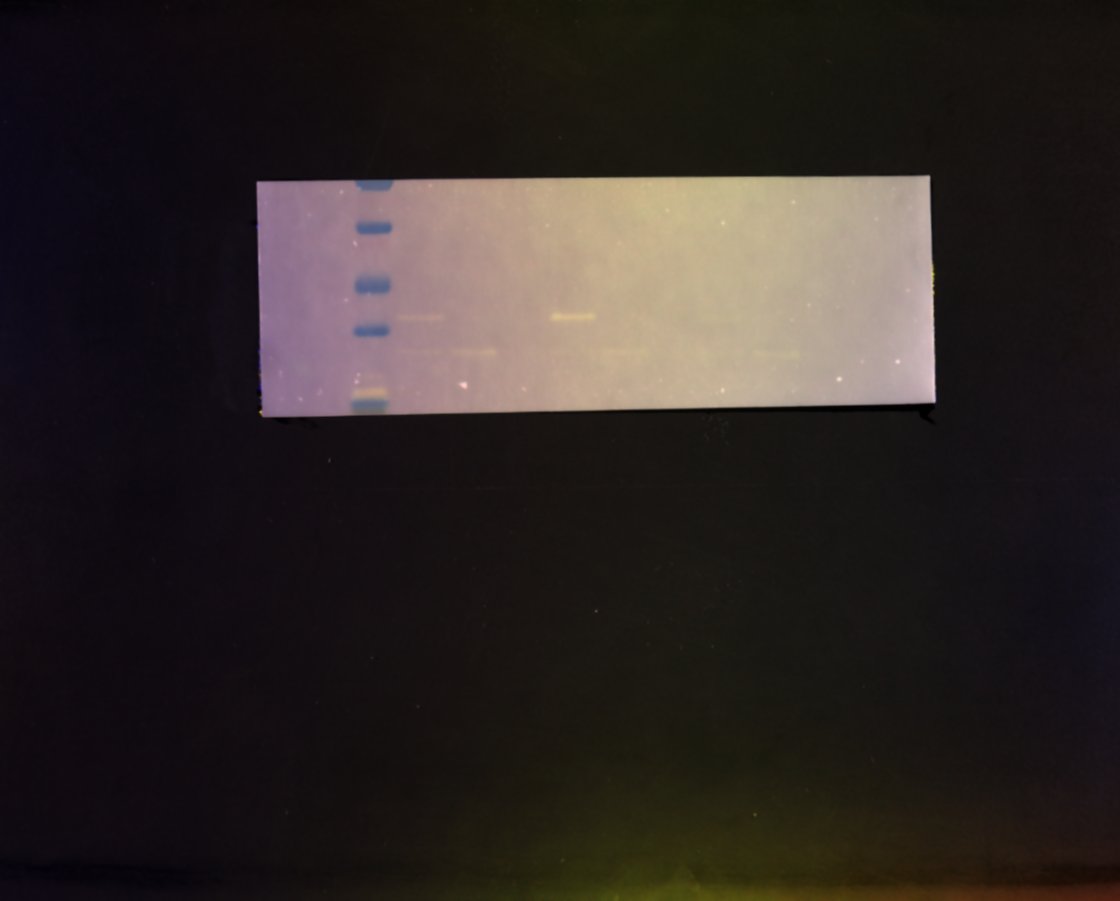

Supplement: Supplementary file 26 — Unprocessed western blots for Extended Data Fig. 4a,b. [file 42255_2025_1225_MOESM26_ESM.zip › Zuhra_WesternBlot_Extended_Fig4/Zuhra_WesternBlot_Extended_Fig4_c/GSTA1/FigE4g_GSTA1_Experiment5-6-7_GSTA1_marker.jpg]

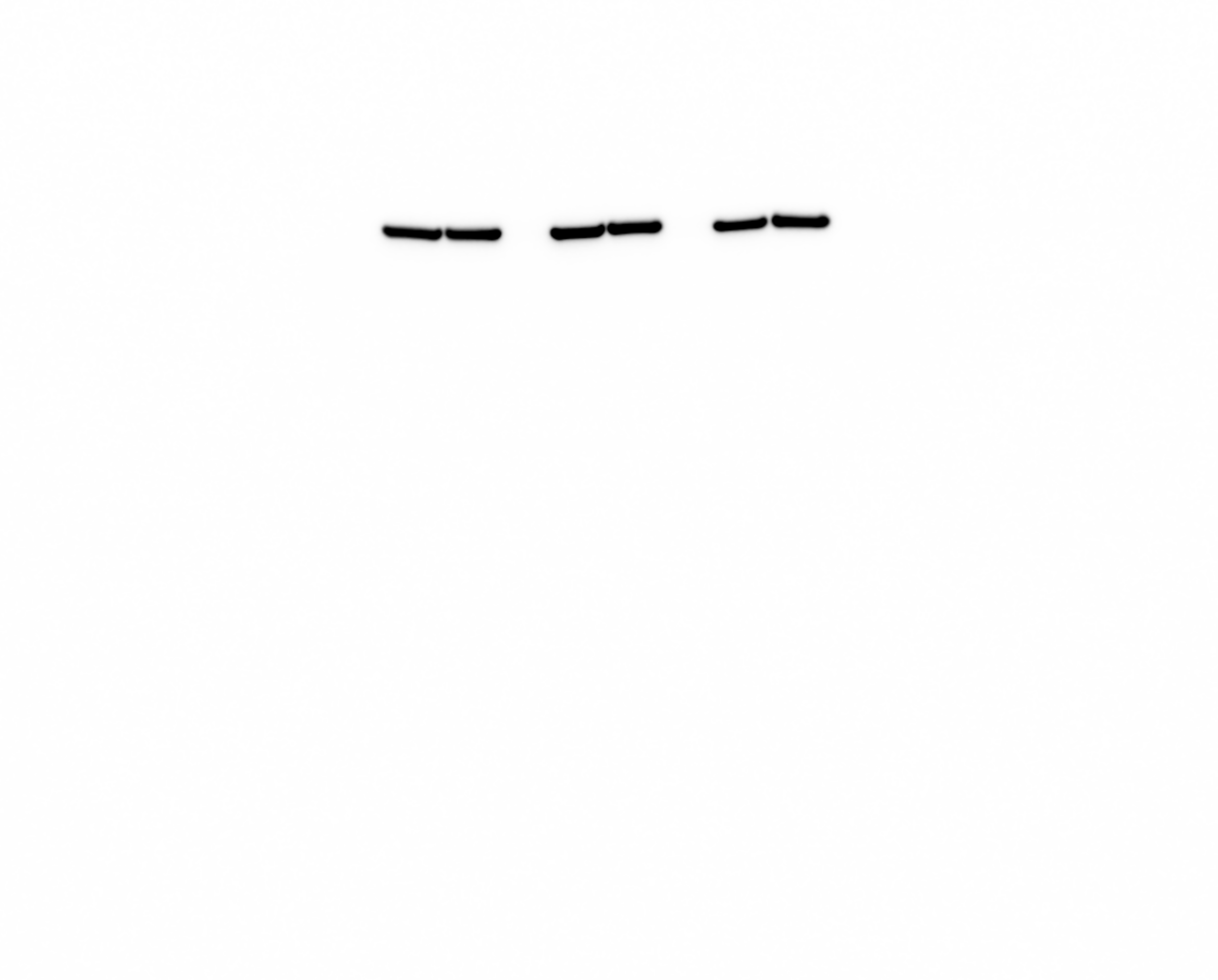

Supplement: Supplementary file 26 — Unprocessed western blots for Extended Data Fig. 4a,b. [file 42255_2025_1225_MOESM26_ESM.zip › Zuhra_WesternBlot_Extended_Fig4/Zuhra_WesternBlot_Extended_Fig4_c/GSTA2/FigE4g_GSTA2_Experiment1-2-3_actin.jpg]

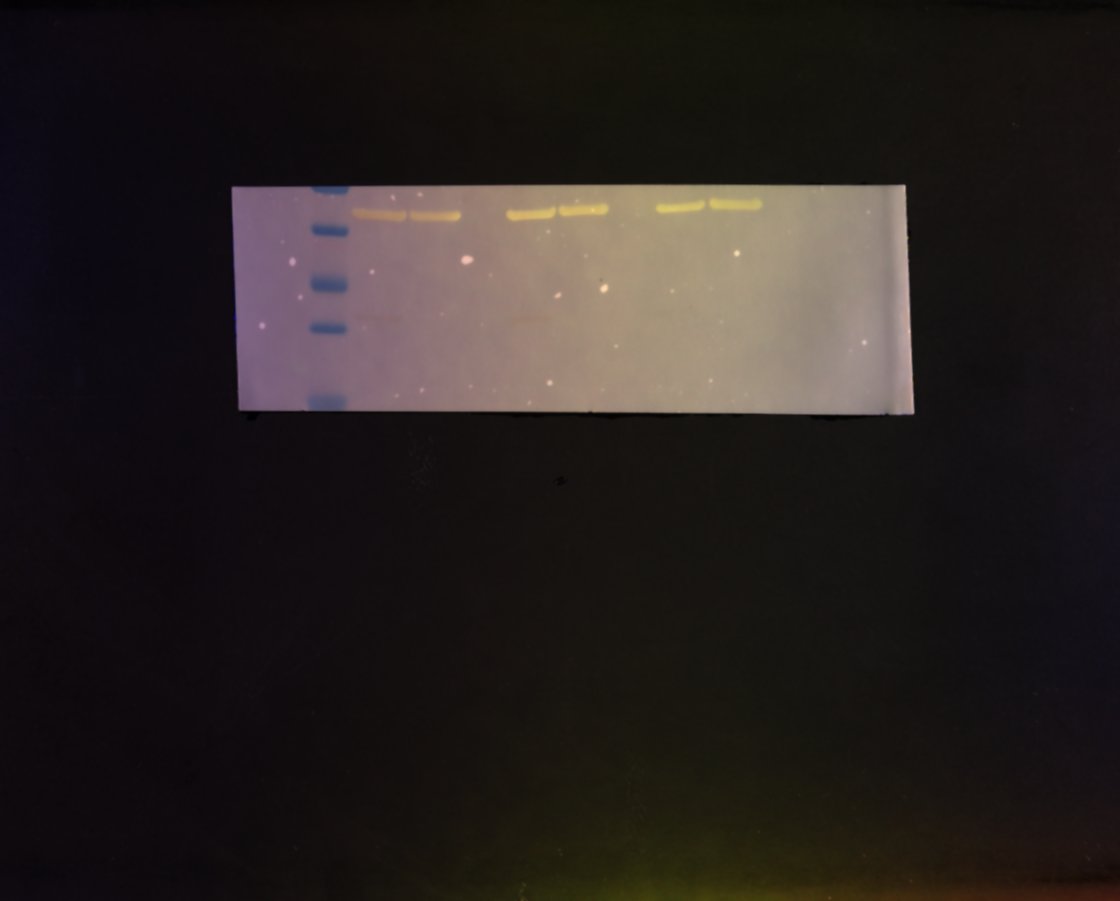

Supplement: Supplementary file 26 — Unprocessed western blots for Extended Data Fig. 4a,b. [file 42255_2025_1225_MOESM26_ESM.zip › Zuhra_WesternBlot_Extended_Fig4/Zuhra_WesternBlot_Extended_Fig4_c/GSTA2/FigE4g_GSTA2_Experiment1-2-3_actin_marker.jpg]

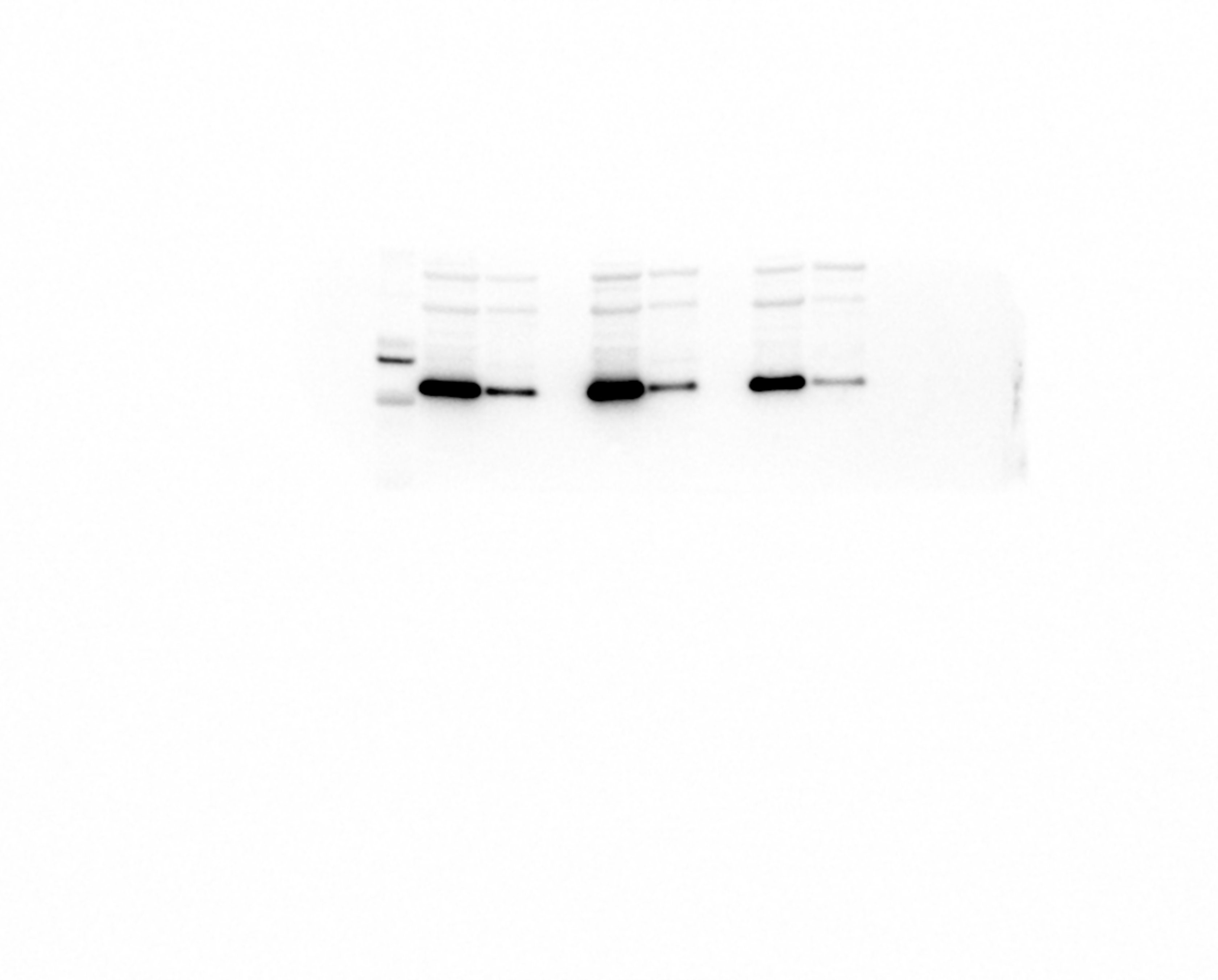

Supplement: Supplementary file 26 — Unprocessed western blots for Extended Data Fig. 4a,b. [file 42255_2025_1225_MOESM26_ESM.zip › Zuhra_WesternBlot_Extended_Fig4/Zuhra_WesternBlot_Extended_Fig4_c/GSTA2/FigE4g_GSTA2_Experiment1-2-3_GSTA2.jpg]

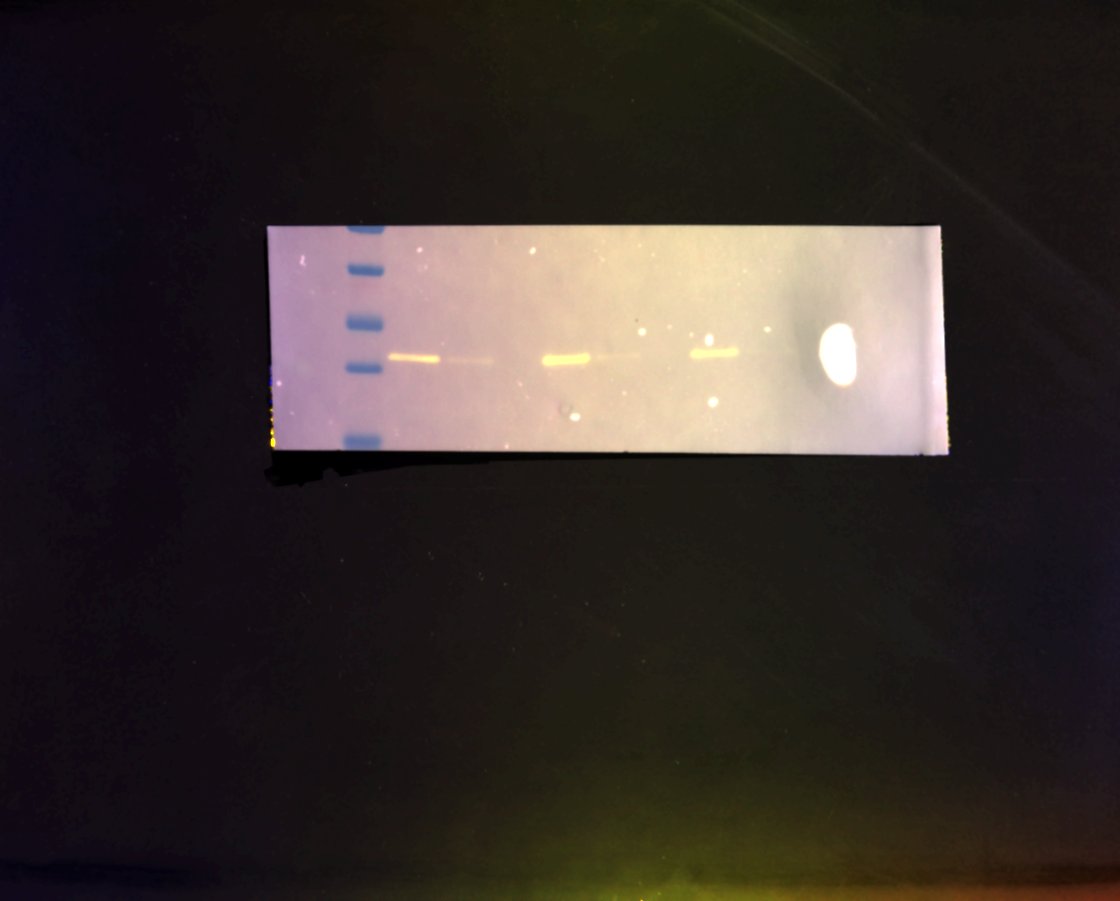

Supplement: Supplementary file 26 — Unprocessed western blots for Extended Data Fig. 4a,b. [file 42255_2025_1225_MOESM26_ESM.zip › Zuhra_WesternBlot_Extended_Fig4/Zuhra_WesternBlot_Extended_Fig4_c/GSTA2/FigE4g_GSTA2_Experiment1-2-3_GSTA2_marker.jpg]

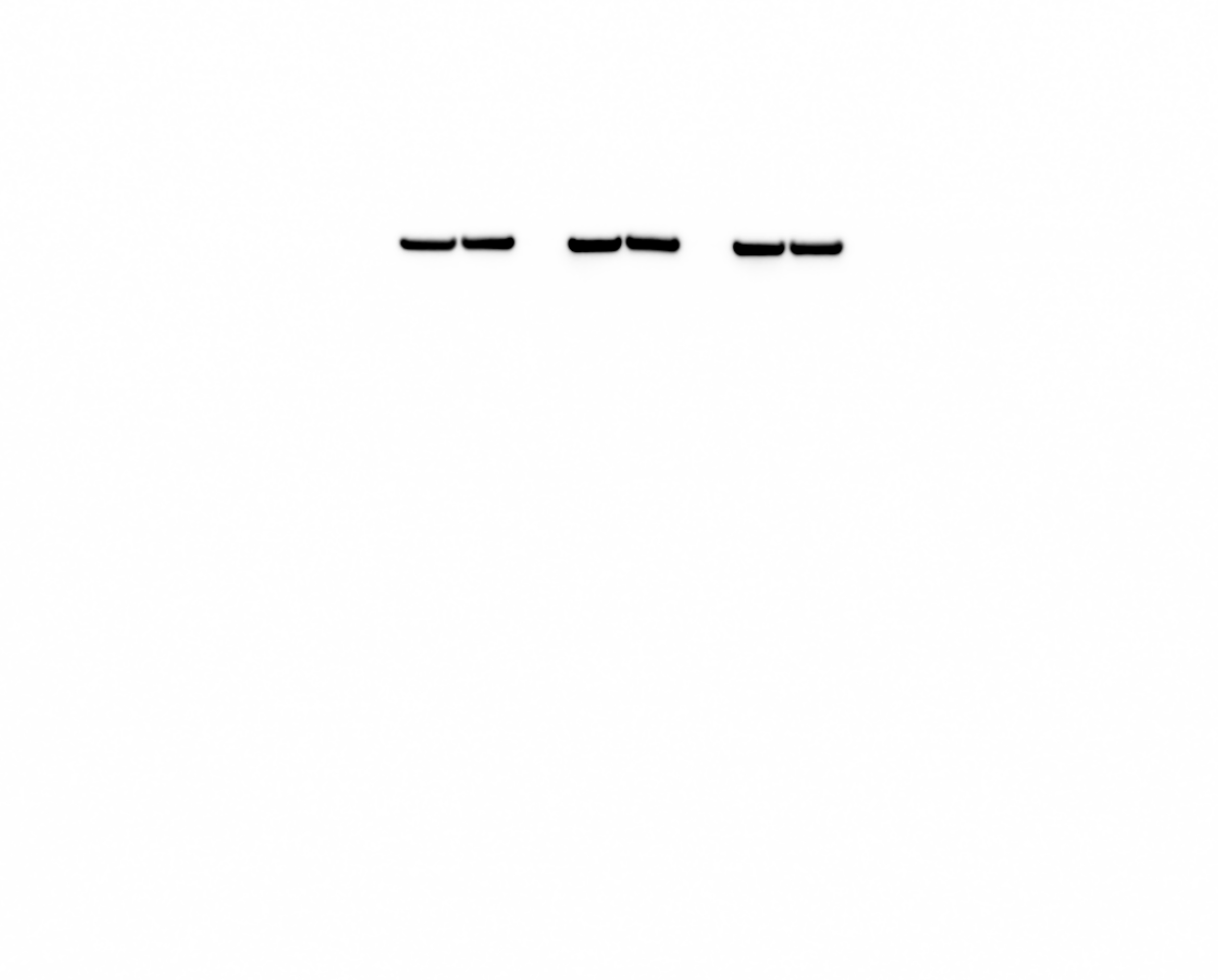

Supplement: Supplementary file 26 — Unprocessed western blots for Extended Data Fig. 4a,b. [file 42255_2025_1225_MOESM26_ESM.zip › Zuhra_WesternBlot_Extended_Fig4/Zuhra_WesternBlot_Extended_Fig4_c/GSTA2/FigE4g_GSTA2_Experiment4-5-6_actin.jpg]

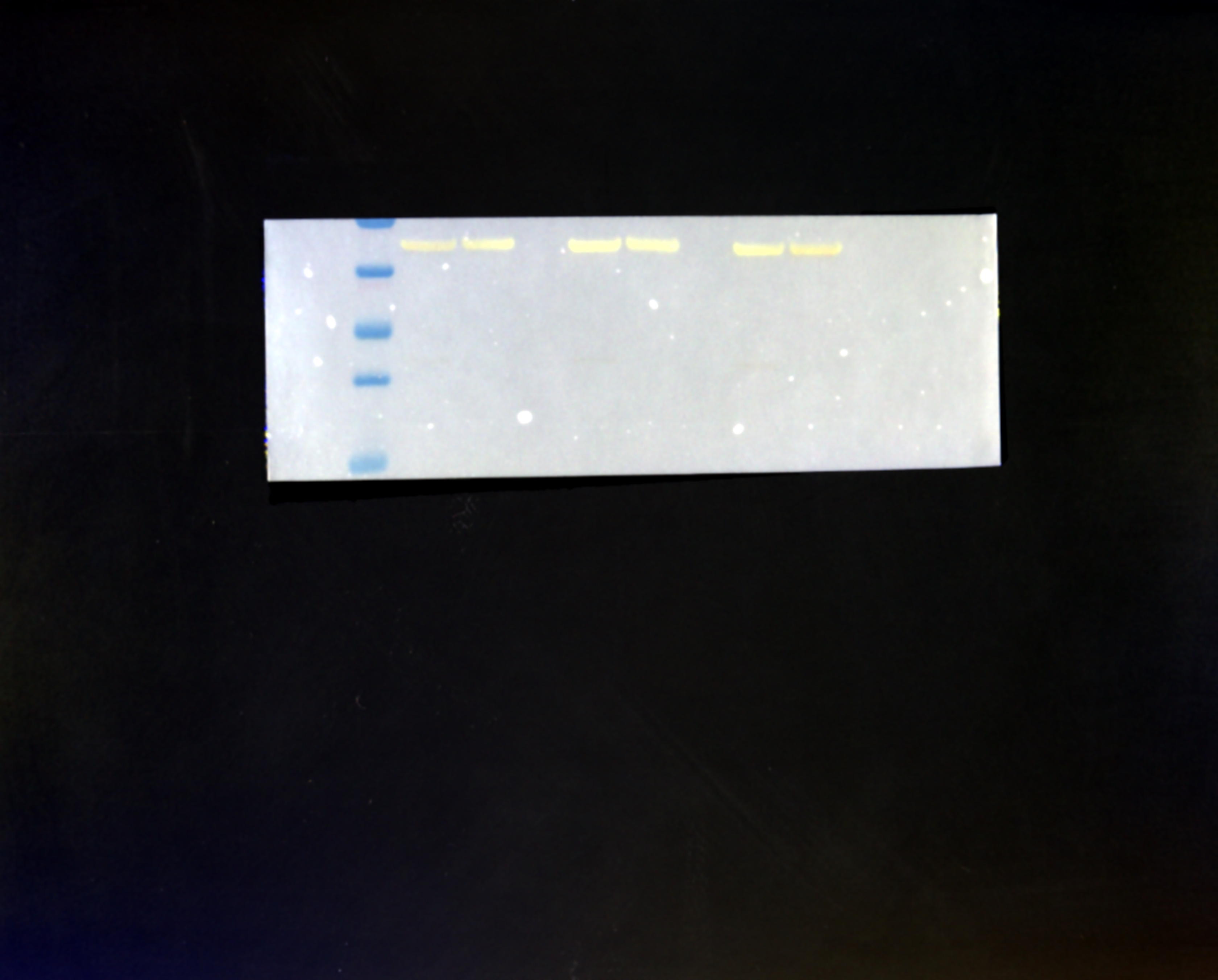

Supplement: Supplementary file 26 — Unprocessed western blots for Extended Data Fig. 4a,b. [file 42255_2025_1225_MOESM26_ESM.zip › Zuhra_WesternBlot_Extended_Fig4/Zuhra_WesternBlot_Extended_Fig4_c/GSTA2/FigE4g_GSTA2_Experiment4-5-6_actin_marker.jpg]

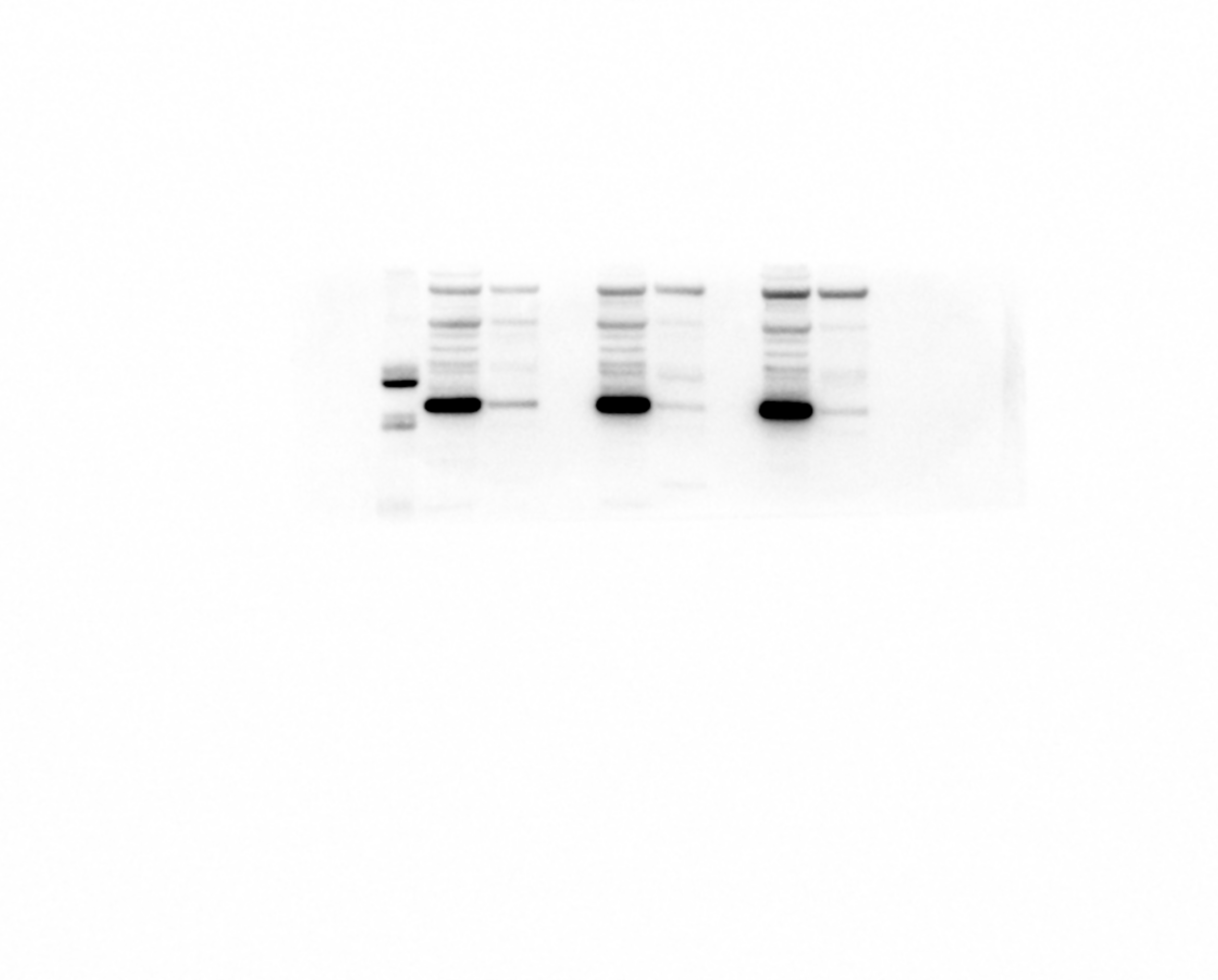

Supplement: Supplementary file 26 — Unprocessed western blots for Extended Data Fig. 4a,b. [file 42255_2025_1225_MOESM26_ESM.zip › Zuhra_WesternBlot_Extended_Fig4/Zuhra_WesternBlot_Extended_Fig4_c/GSTA2/FigE4g_GSTA2_Experiment4-5-6_GSTA2.jpg]

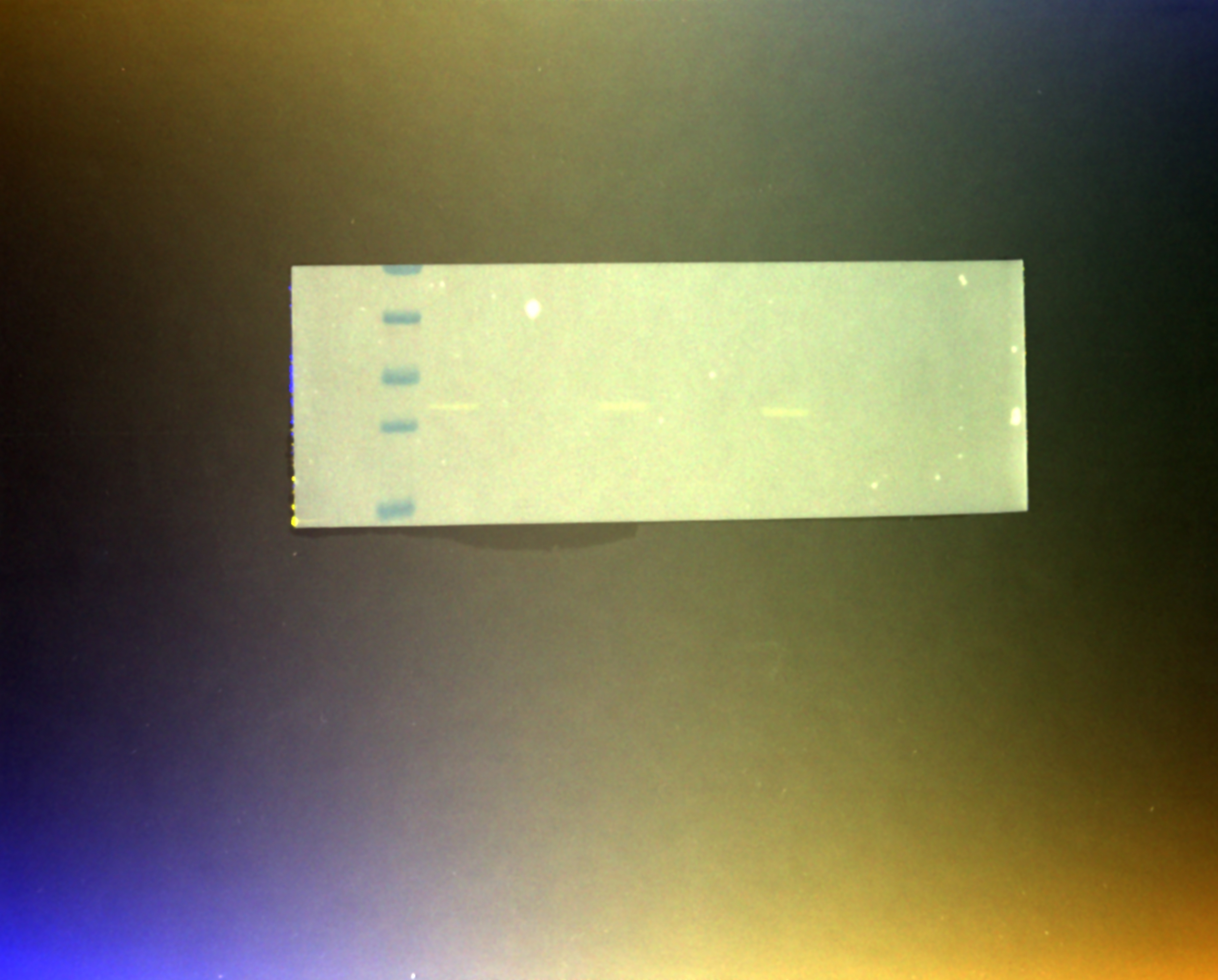

Supplement: Supplementary file 26 — Unprocessed western blots for Extended Data Fig. 4a,b. [file 42255_2025_1225_MOESM26_ESM.zip › Zuhra_WesternBlot_Extended_Fig4/Zuhra_WesternBlot_Extended_Fig4_c/GSTA2/FigE4g_GSTA2_Experiment4-5-6_GSTA2_marker.jpg]

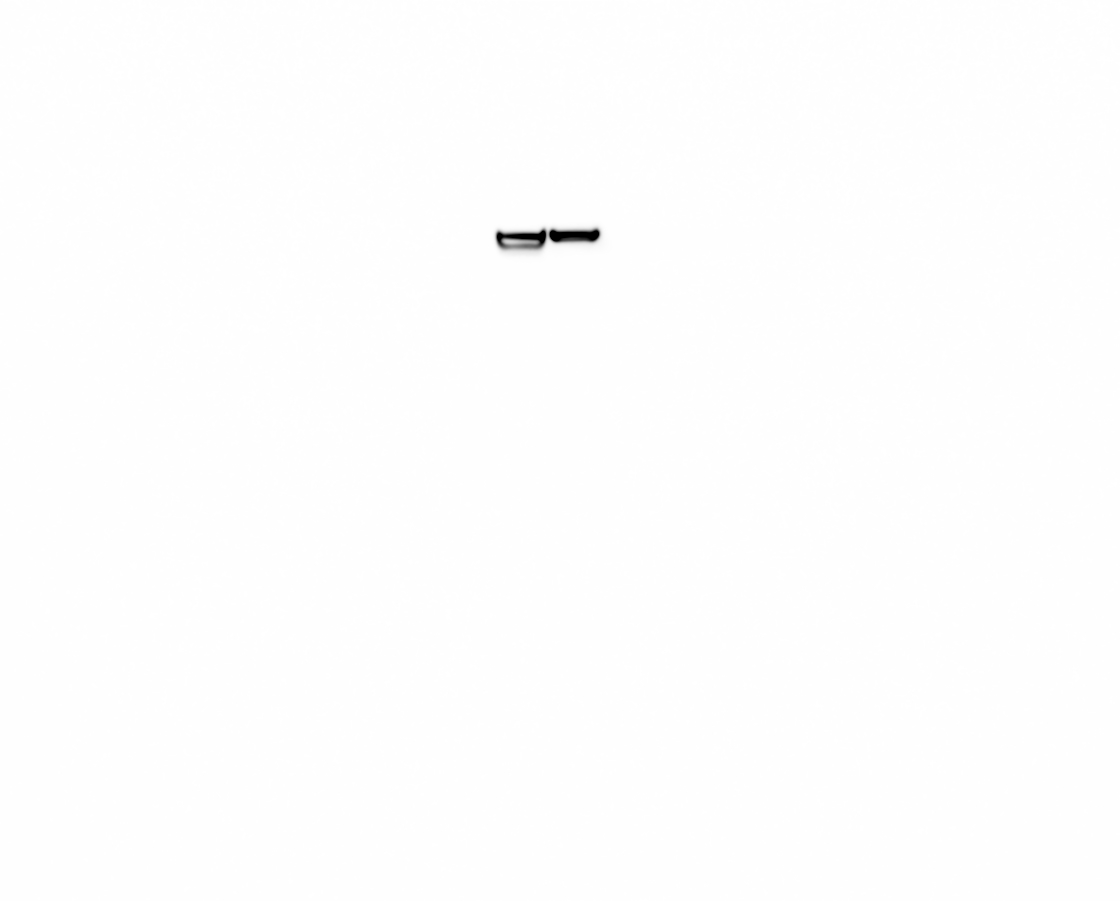

Supplement: Supplementary file 26 — Unprocessed western blots for Extended Data Fig. 4a,b. [file 42255_2025_1225_MOESM26_ESM.zip › Zuhra_WesternBlot_Extended_Fig4/Zuhra_WesternBlot_Extended_Fig4_c/MGST1/FigE4g_MGST1_Experiment1_actin.jpg]

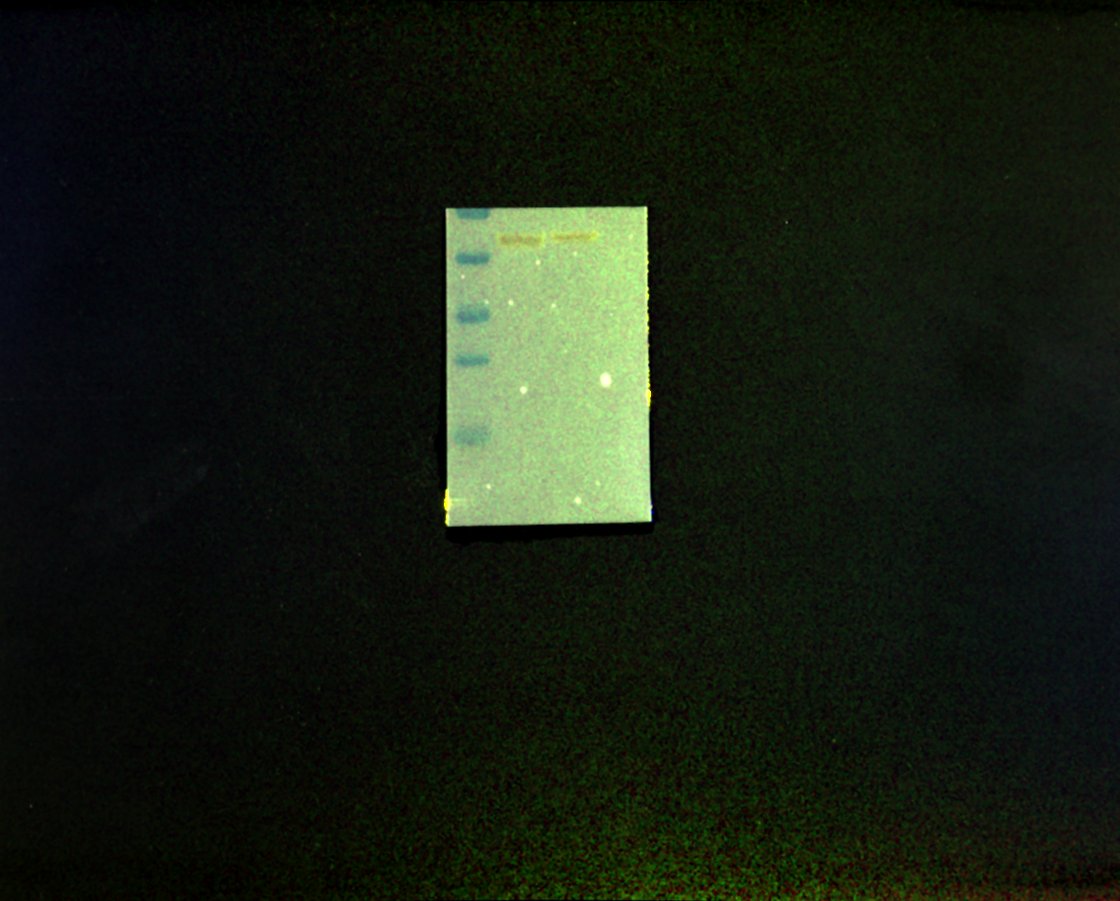

Supplement: Supplementary file 26 — Unprocessed western blots for Extended Data Fig. 4a,b. [file 42255_2025_1225_MOESM26_ESM.zip › Zuhra_WesternBlot_Extended_Fig4/Zuhra_WesternBlot_Extended_Fig4_c/MGST1/FigE4g_MGST1_Experiment1_actin_marker.jpg]

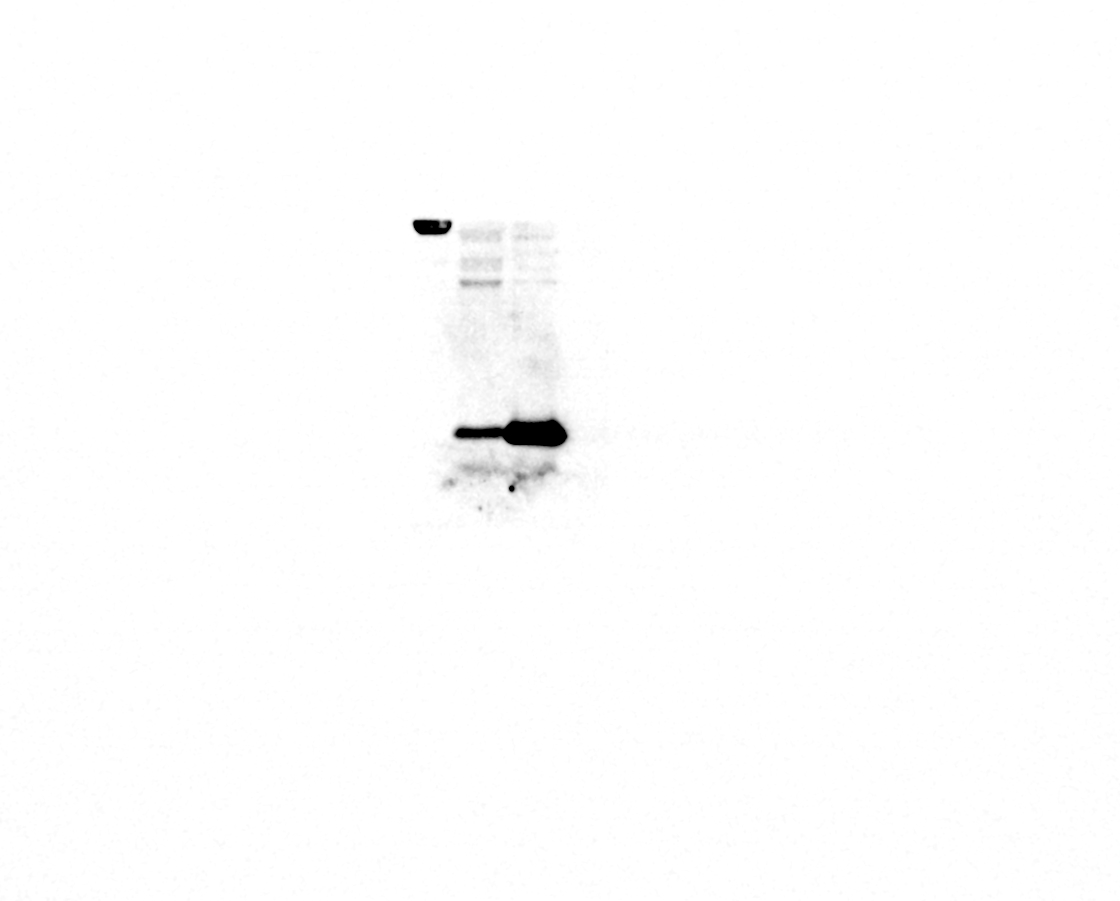

Supplement: Supplementary file 26 — Unprocessed western blots for Extended Data Fig. 4a,b. [file 42255_2025_1225_MOESM26_ESM.zip › Zuhra_WesternBlot_Extended_Fig4/Zuhra_WesternBlot_Extended_Fig4_c/MGST1/FigE4g_MGST1_Experiment1_MGST1.jpg]

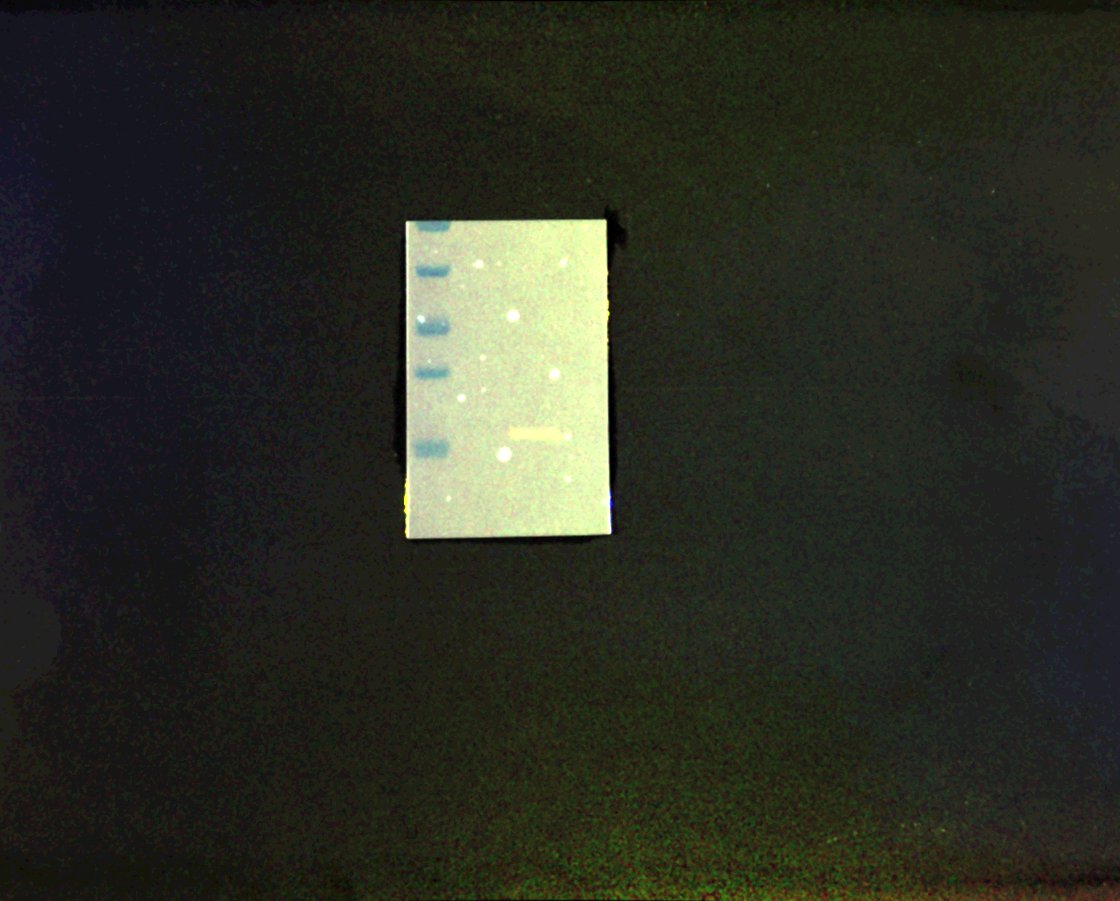

Supplement: Supplementary file 26 — Unprocessed western blots for Extended Data Fig. 4a,b. [file 42255_2025_1225_MOESM26_ESM.zip › Zuhra_WesternBlot_Extended_Fig4/Zuhra_WesternBlot_Extended_Fig4_c/MGST1/FigE4g_MGST1_Experiment1_MGST1_marker.jpg]

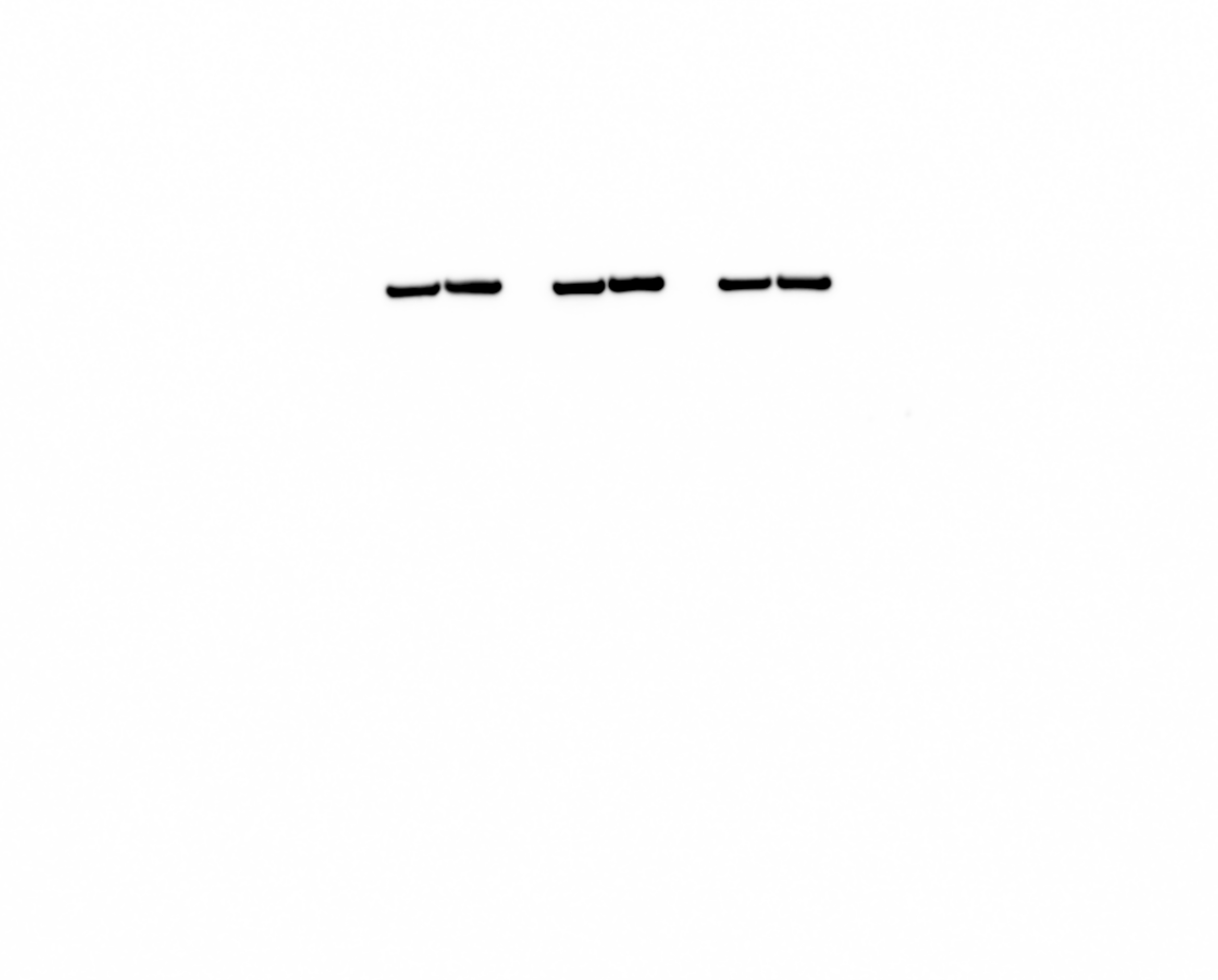

Supplement: Supplementary file 26 — Unprocessed western blots for Extended Data Fig. 4a,b. [file 42255_2025_1225_MOESM26_ESM.zip › Zuhra_WesternBlot_Extended_Fig4/Zuhra_WesternBlot_Extended_Fig4_c/MGST1/FigE4g_MGST1_Experiment2-3-4_actin.jpg]

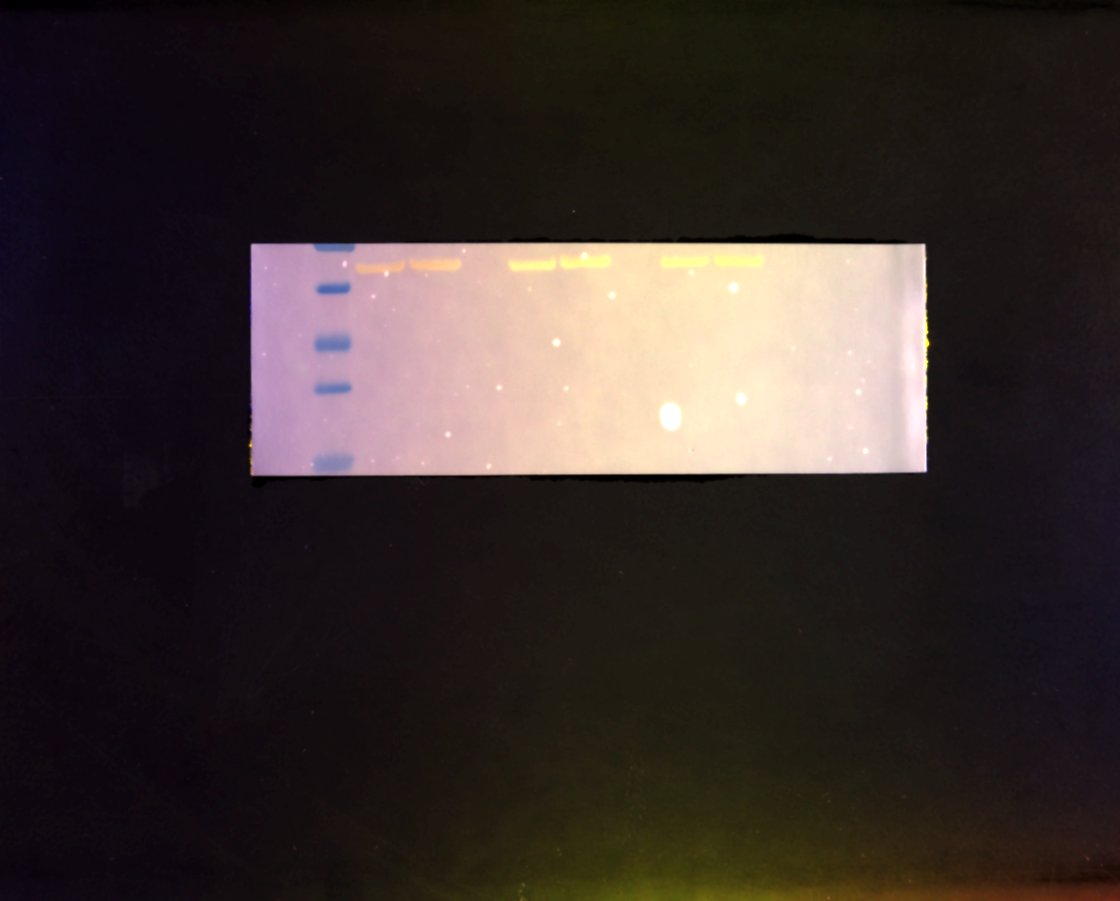

Supplement: Supplementary file 26 — Unprocessed western blots for Extended Data Fig. 4a,b. [file 42255_2025_1225_MOESM26_ESM.zip › Zuhra_WesternBlot_Extended_Fig4/Zuhra_WesternBlot_Extended_Fig4_c/MGST1/FigE4g_MGST1_Experiment2-3-4_actin_marker.jpg]

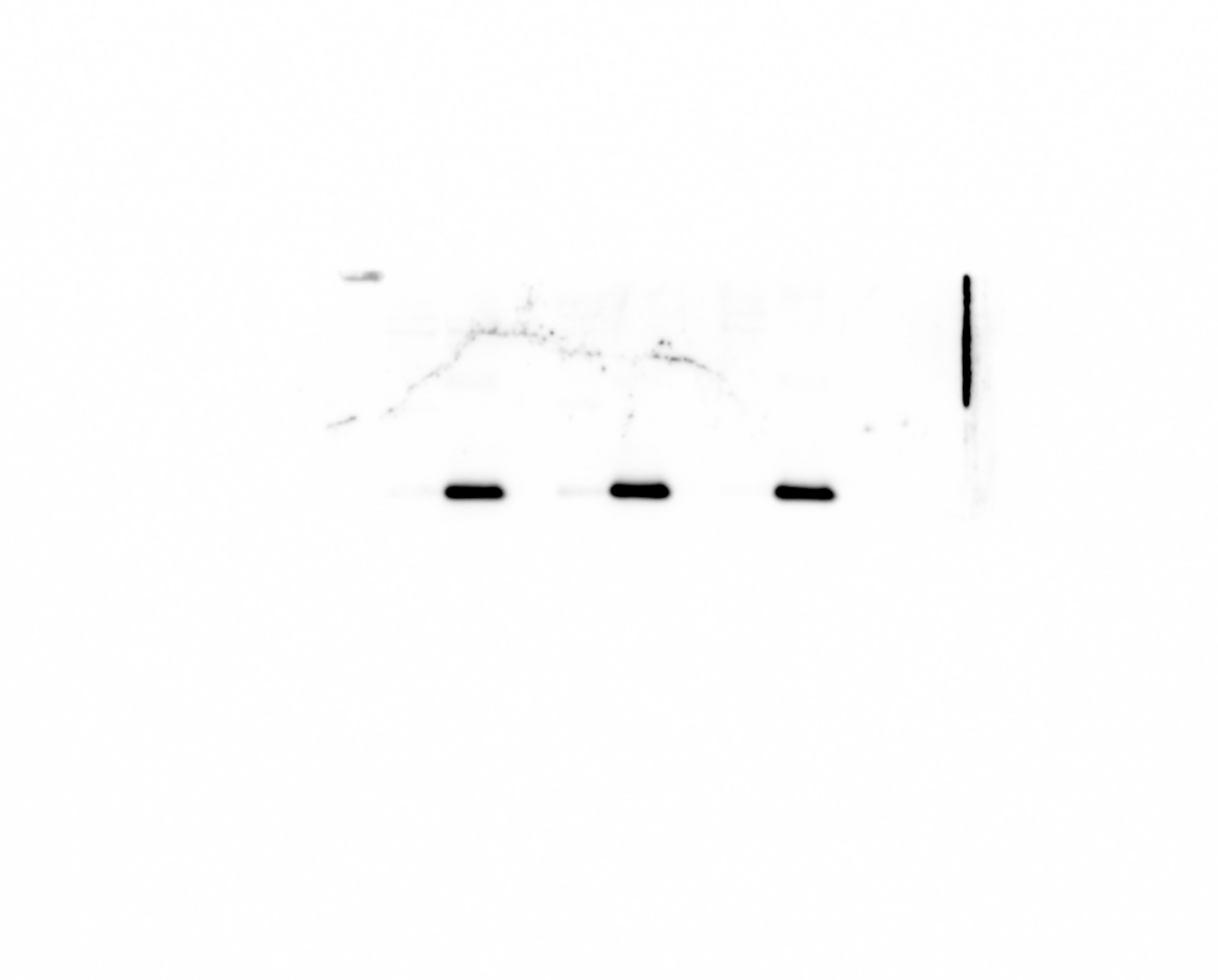

Supplement: Supplementary file 26 — Unprocessed western blots for Extended Data Fig. 4a,b. [file 42255_2025_1225_MOESM26_ESM.zip › Zuhra_WesternBlot_Extended_Fig4/Zuhra_WesternBlot_Extended_Fig4_c/MGST1/FigE4g_MGST1_Experiment2-3-4_MGST1.jpg]

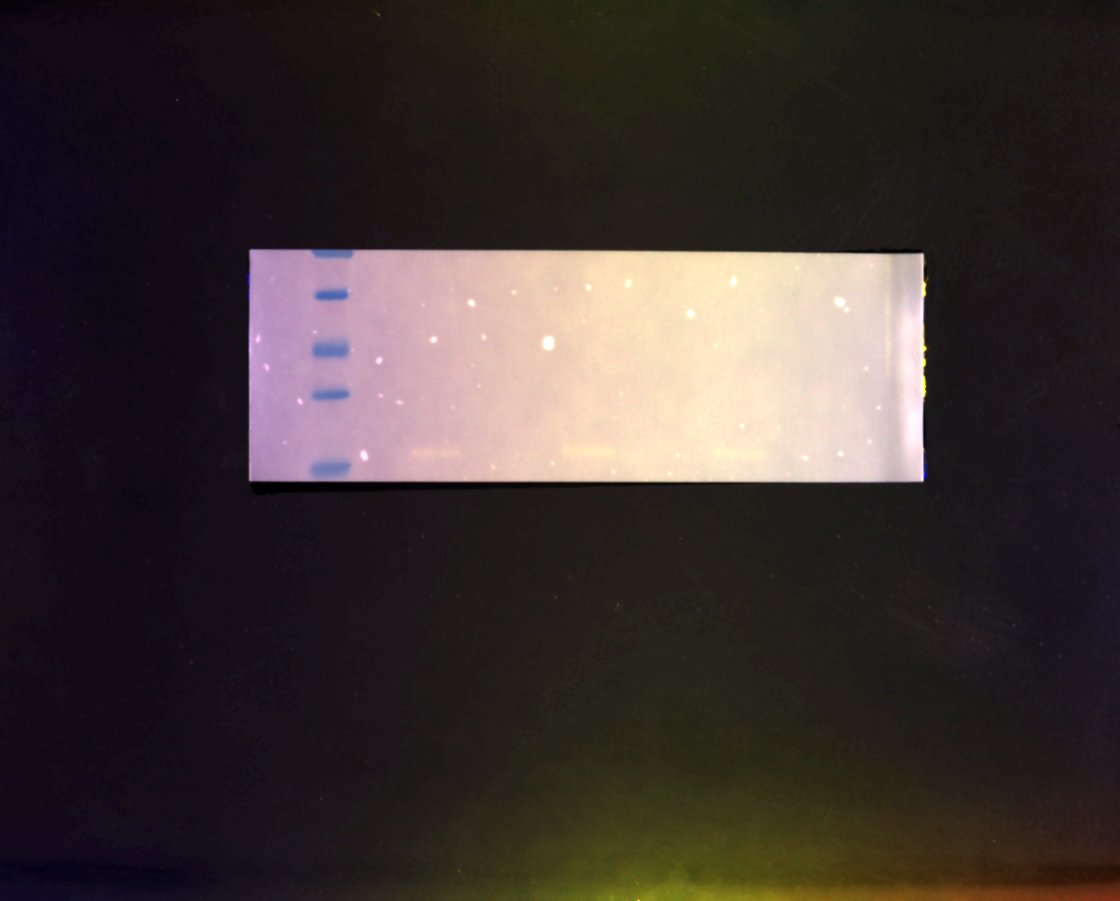

Supplement: Supplementary file 26 — Unprocessed western blots for Extended Data Fig. 4a,b. [file 42255_2025_1225_MOESM26_ESM.zip › Zuhra_WesternBlot_Extended_Fig4/Zuhra_WesternBlot_Extended_Fig4_c/MGST1/FigE4g_MGST1_Experiment2-3-4_MGST1_marker.jpg]

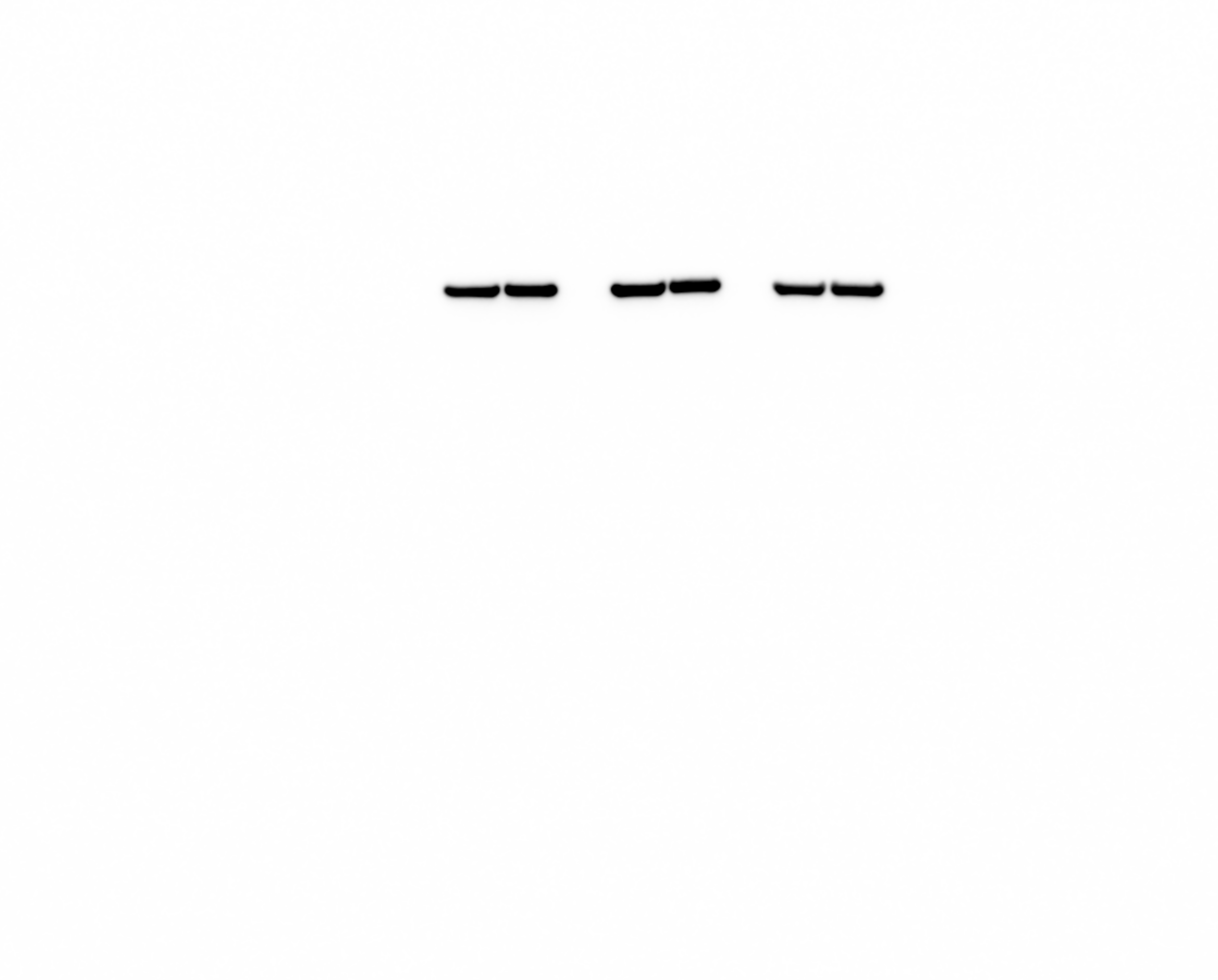

Supplement: Supplementary file 26 — Unprocessed western blots for Extended Data Fig. 4a,b. [file 42255_2025_1225_MOESM26_ESM.zip › Zuhra_WesternBlot_Extended_Fig4/Zuhra_WesternBlot_Extended_Fig4_c/MGST1/FigE4g_MGST1_Experiment5-6-7_actin.jpg]

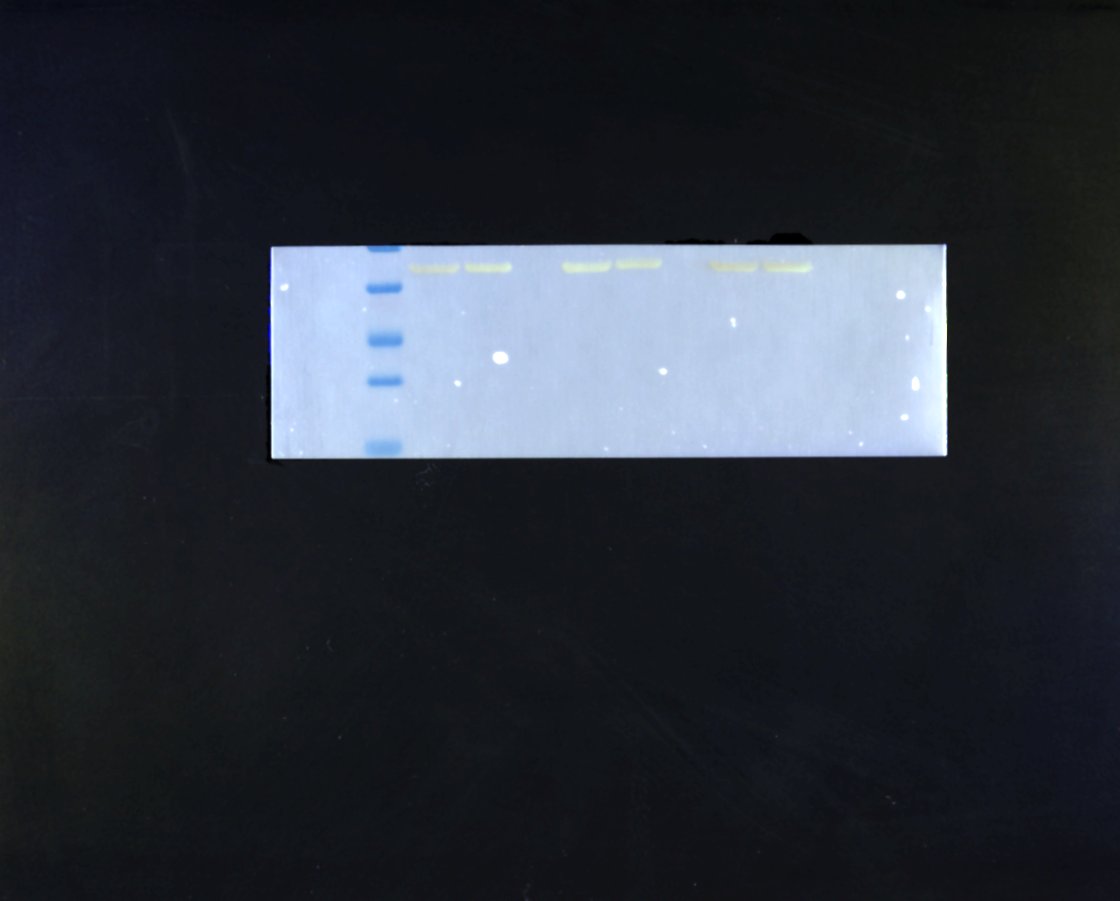

Supplement: Supplementary file 26 — Unprocessed western blots for Extended Data Fig. 4a,b. [file 42255_2025_1225_MOESM26_ESM.zip › Zuhra_WesternBlot_Extended_Fig4/Zuhra_WesternBlot_Extended_Fig4_c/MGST1/FigE4g_MGST1_Experiment5-6-7_actin_marker.jpg]

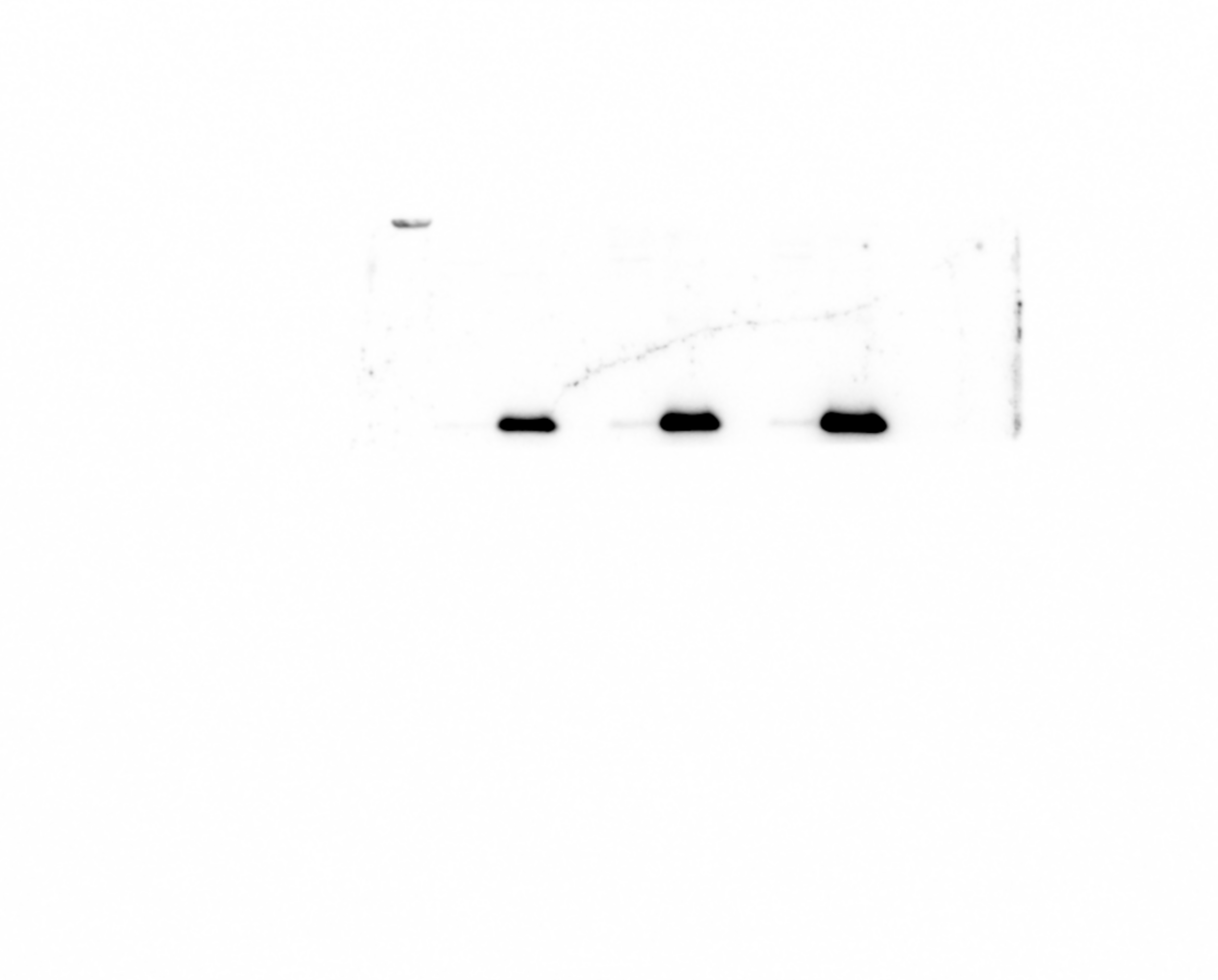

Supplement: Supplementary file 26 — Unprocessed western blots for Extended Data Fig. 4a,b. [file 42255_2025_1225_MOESM26_ESM.zip › Zuhra_WesternBlot_Extended_Fig4/Zuhra_WesternBlot_Extended_Fig4_c/MGST1/FigE4g_MGST1_Experiment5-6-7_MGST1.jpg]

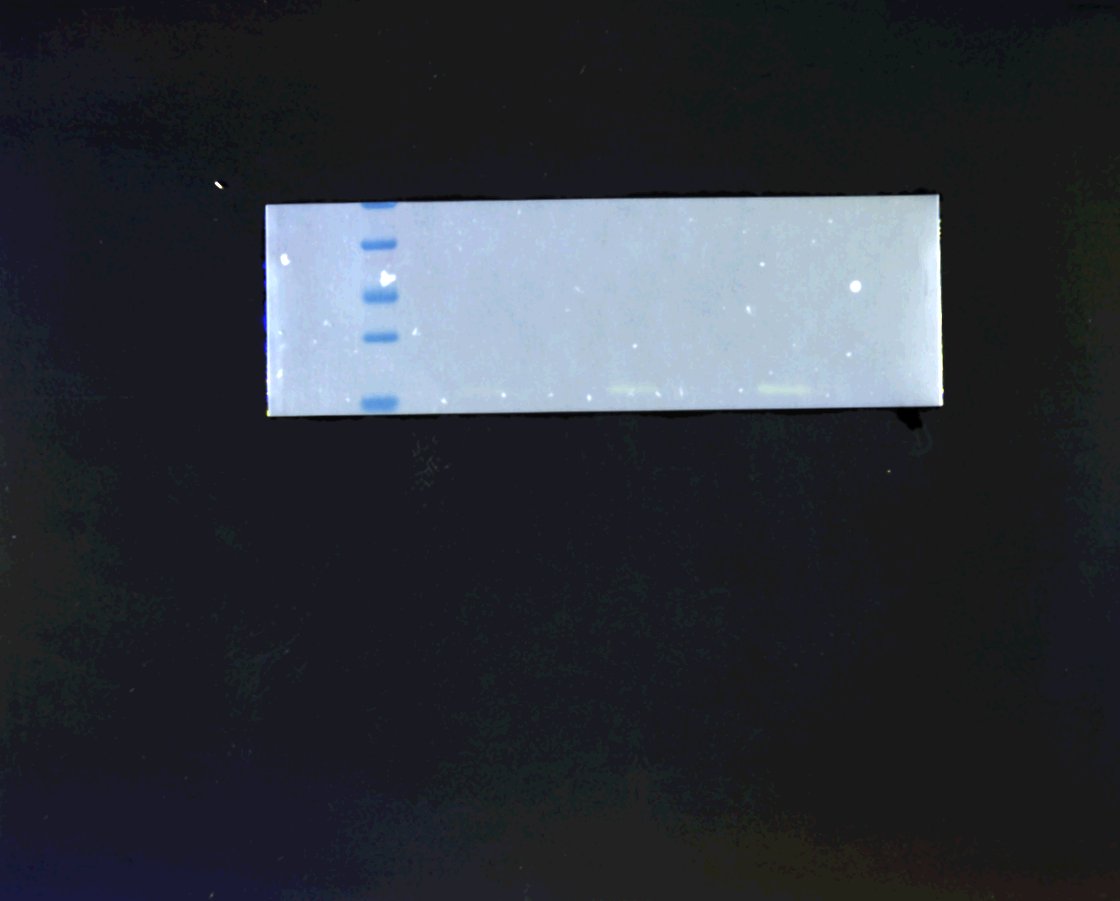

Supplement: Supplementary file 26 — Unprocessed western blots for Extended Data Fig. 4a,b. [file 42255_2025_1225_MOESM26_ESM.zip › Zuhra_WesternBlot_Extended_Fig4/Zuhra_WesternBlot_Extended_Fig4_c/MGST1/FigE4g_MGST1_Experiment5-6-7_MGST1_marker.jpg]

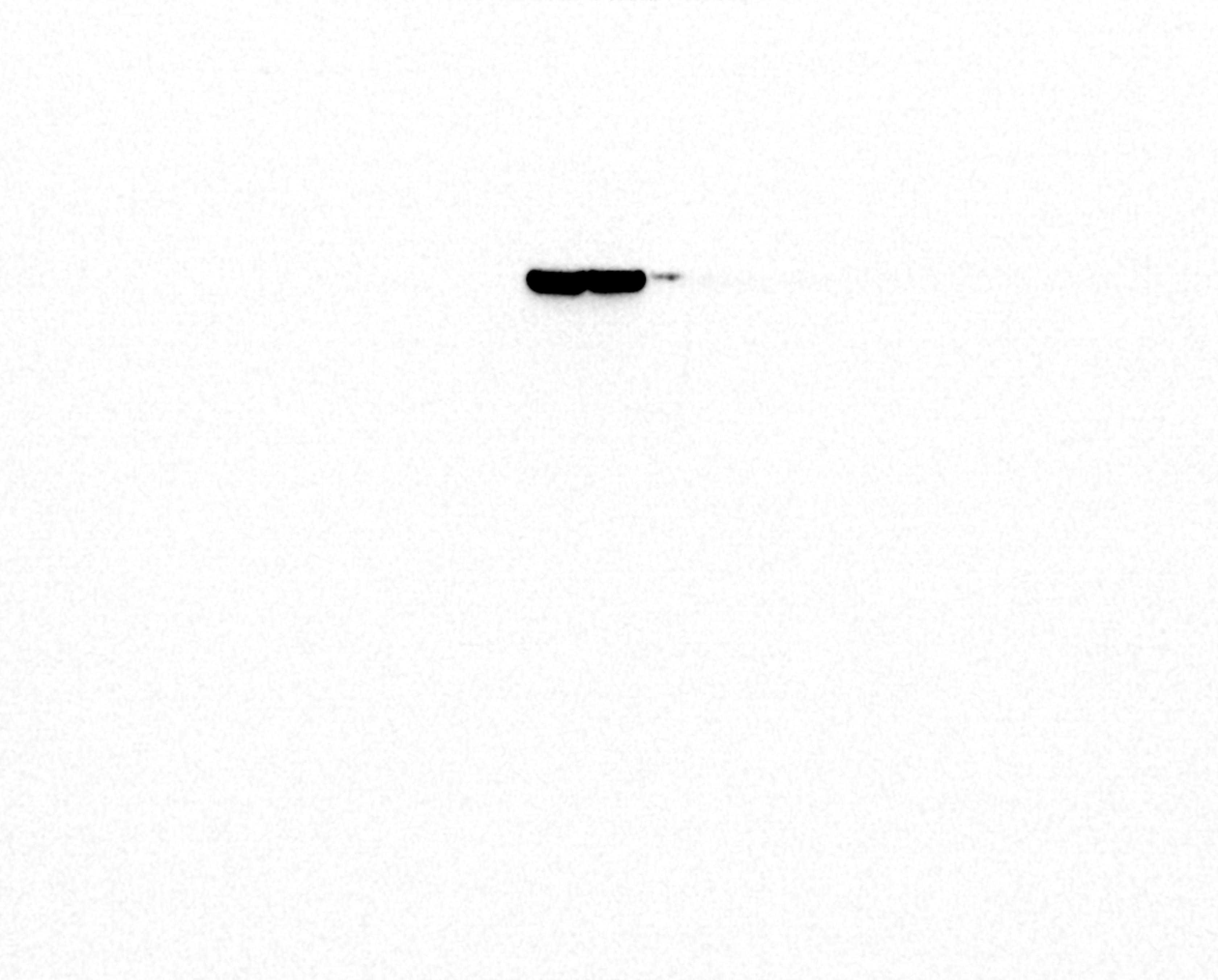

Supplement: Supplementary file 26 — Unprocessed western blots for Extended Data Fig. 4a,b. [file 42255_2025_1225_MOESM26_ESM.zip › Zuhra_WesternBlot_Extended_Fig4/Zuhra_WesternBlot_Extended_Fig4_c/PRDX3/FigE4g_PRDX3_Experiment1_actin.jpg]

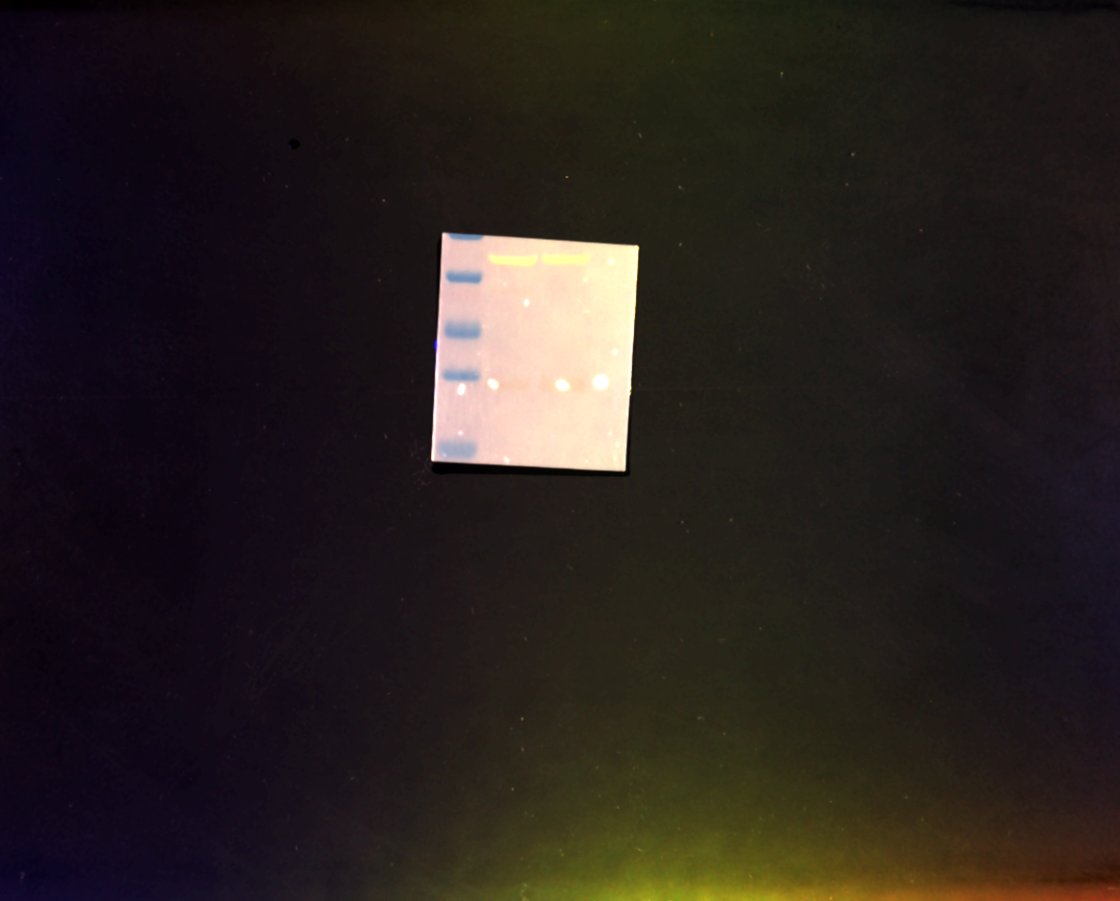

Supplement: Supplementary file 26 — Unprocessed western blots for Extended Data Fig. 4a,b. [file 42255_2025_1225_MOESM26_ESM.zip › Zuhra_WesternBlot_Extended_Fig4/Zuhra_WesternBlot_Extended_Fig4_c/PRDX3/FigE4g_PRDX3_Experiment1_actin_marker.tif]

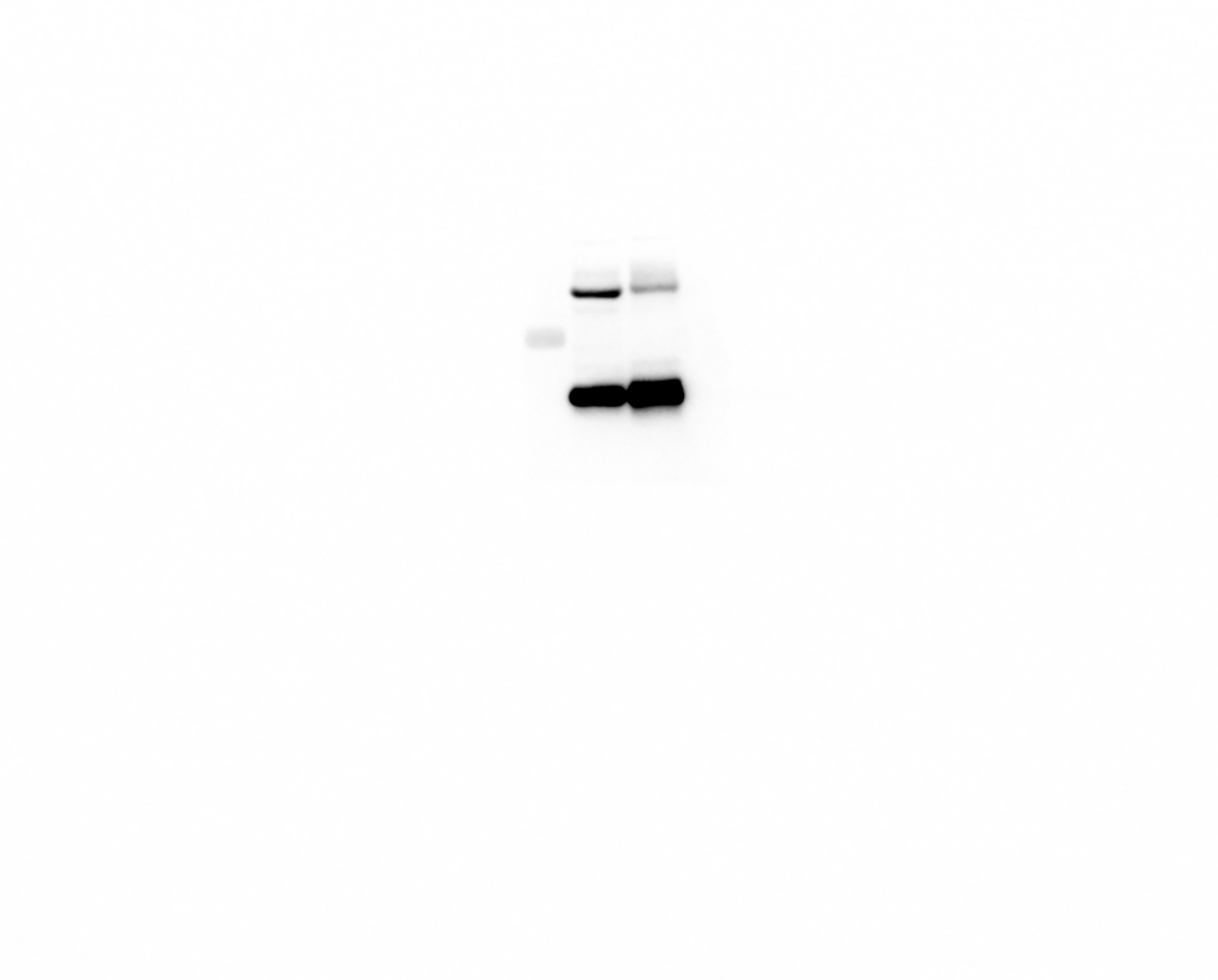

Supplement: Supplementary file 26 — Unprocessed western blots for Extended Data Fig. 4a,b. [file 42255_2025_1225_MOESM26_ESM.zip › Zuhra_WesternBlot_Extended_Fig4/Zuhra_WesternBlot_Extended_Fig4_c/PRDX3/FigE4g_PRDX3_Experiment1_PRDX3.jpg]

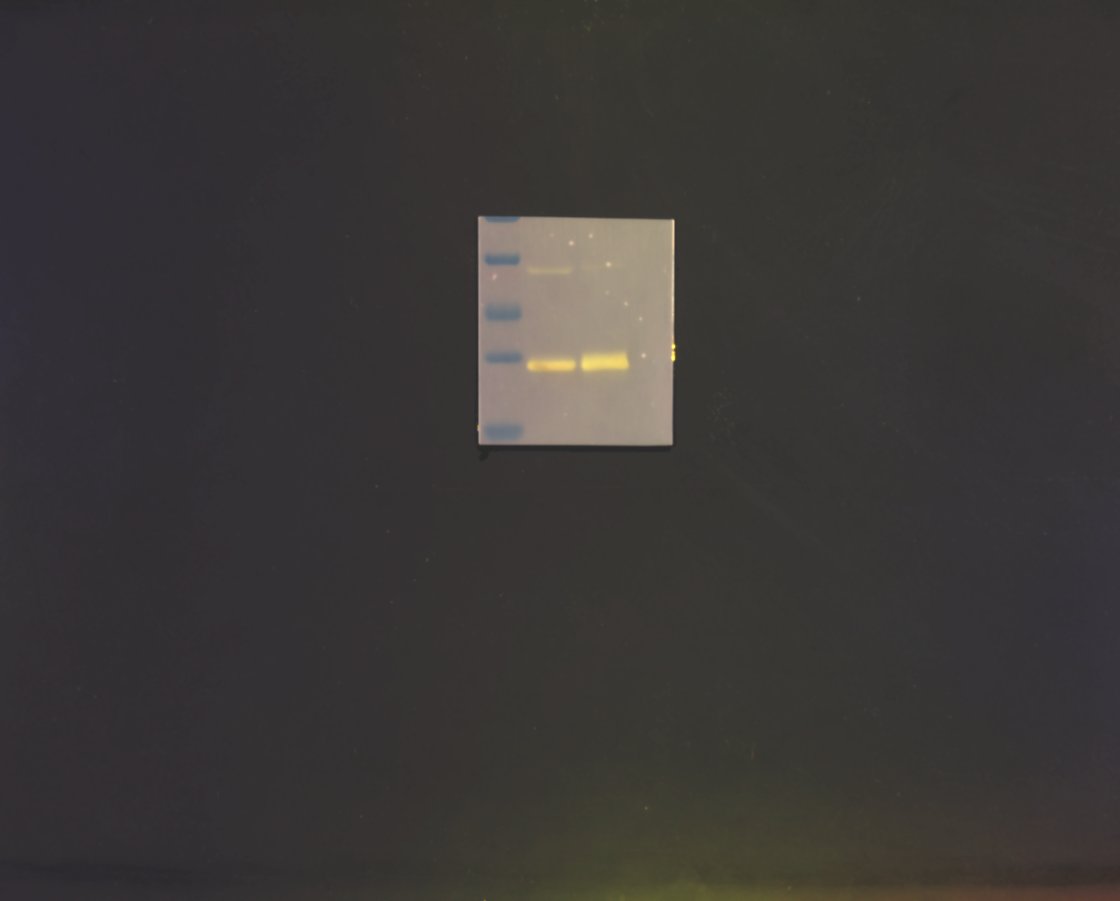

Supplement: Supplementary file 26 — Unprocessed western blots for Extended Data Fig. 4a,b. [file 42255_2025_1225_MOESM26_ESM.zip › Zuhra_WesternBlot_Extended_Fig4/Zuhra_WesternBlot_Extended_Fig4_c/PRDX3/FigE4g_PRDX3_Experiment1_PRDX3_marker.tif]

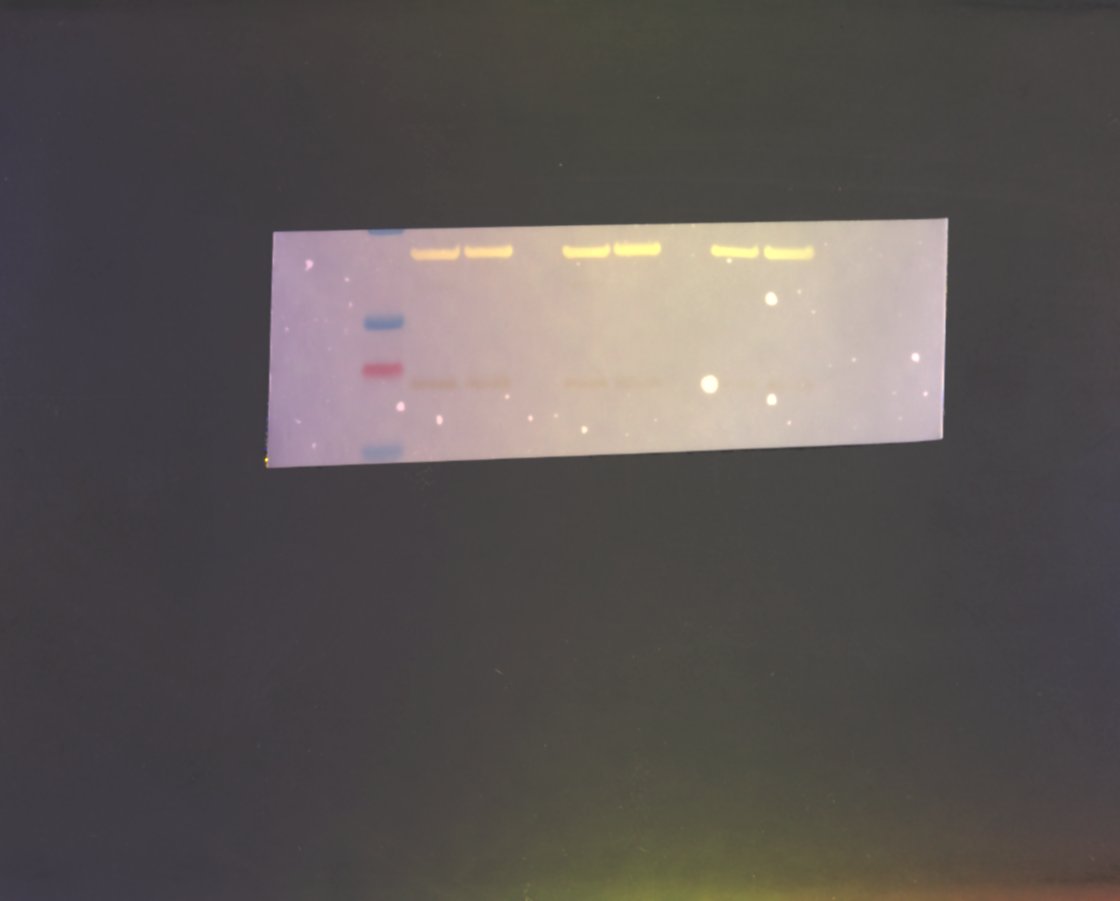

Supplement: Supplementary file 26 — Unprocessed western blots for Extended Data Fig. 4a,b. [file 42255_2025_1225_MOESM26_ESM.zip › Zuhra_WesternBlot_Extended_Fig4/Zuhra_WesternBlot_Extended_Fig4_c/PRDX3/FigE4g_PRDX3_Experiment2-3-4_actin_marker.tif]

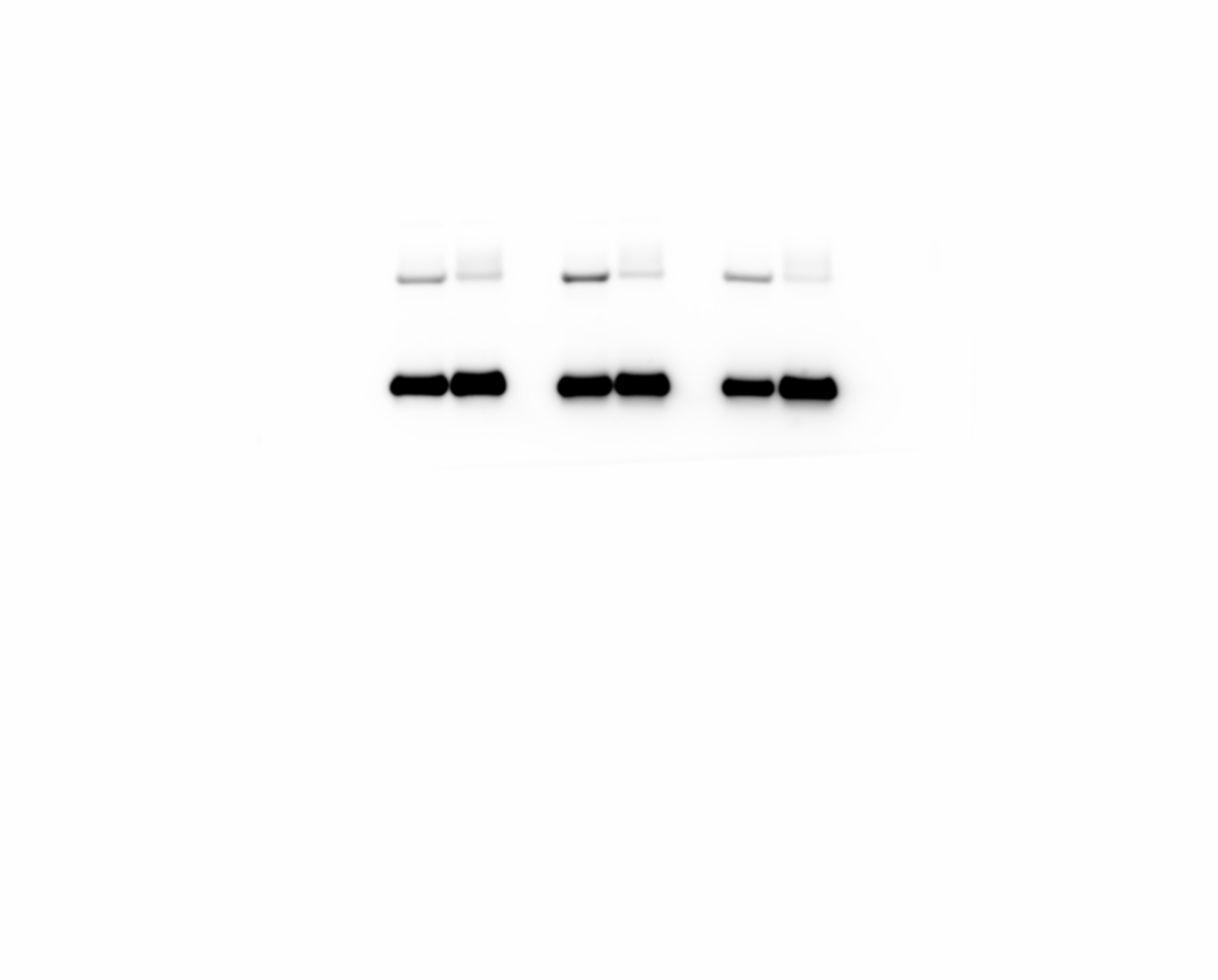

Supplement: Supplementary file 26 — Unprocessed western blots for Extended Data Fig. 4a,b. [file 42255_2025_1225_MOESM26_ESM.zip › Zuhra_WesternBlot_Extended_Fig4/Zuhra_WesternBlot_Extended_Fig4_c/PRDX3/FigE4g_PRDX3_Experiment2-3-4_PRDX3.jpg]

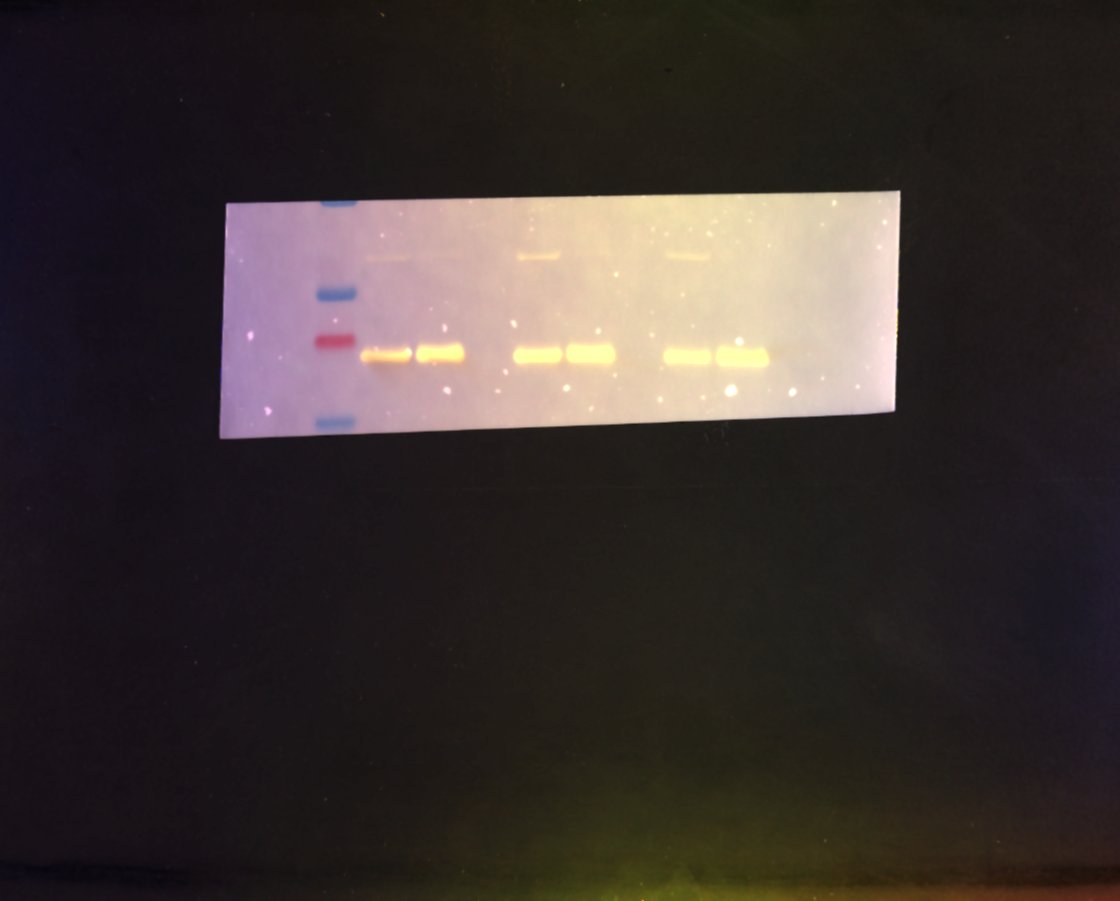

Supplement: Supplementary file 26 — Unprocessed western blots for Extended Data Fig. 4a,b. [file 42255_2025_1225_MOESM26_ESM.zip › Zuhra_WesternBlot_Extended_Fig4/Zuhra_WesternBlot_Extended_Fig4_c/PRDX3/FigE4g_PRDX3_Experiment2-3-4_PRDX3_marker.tif]

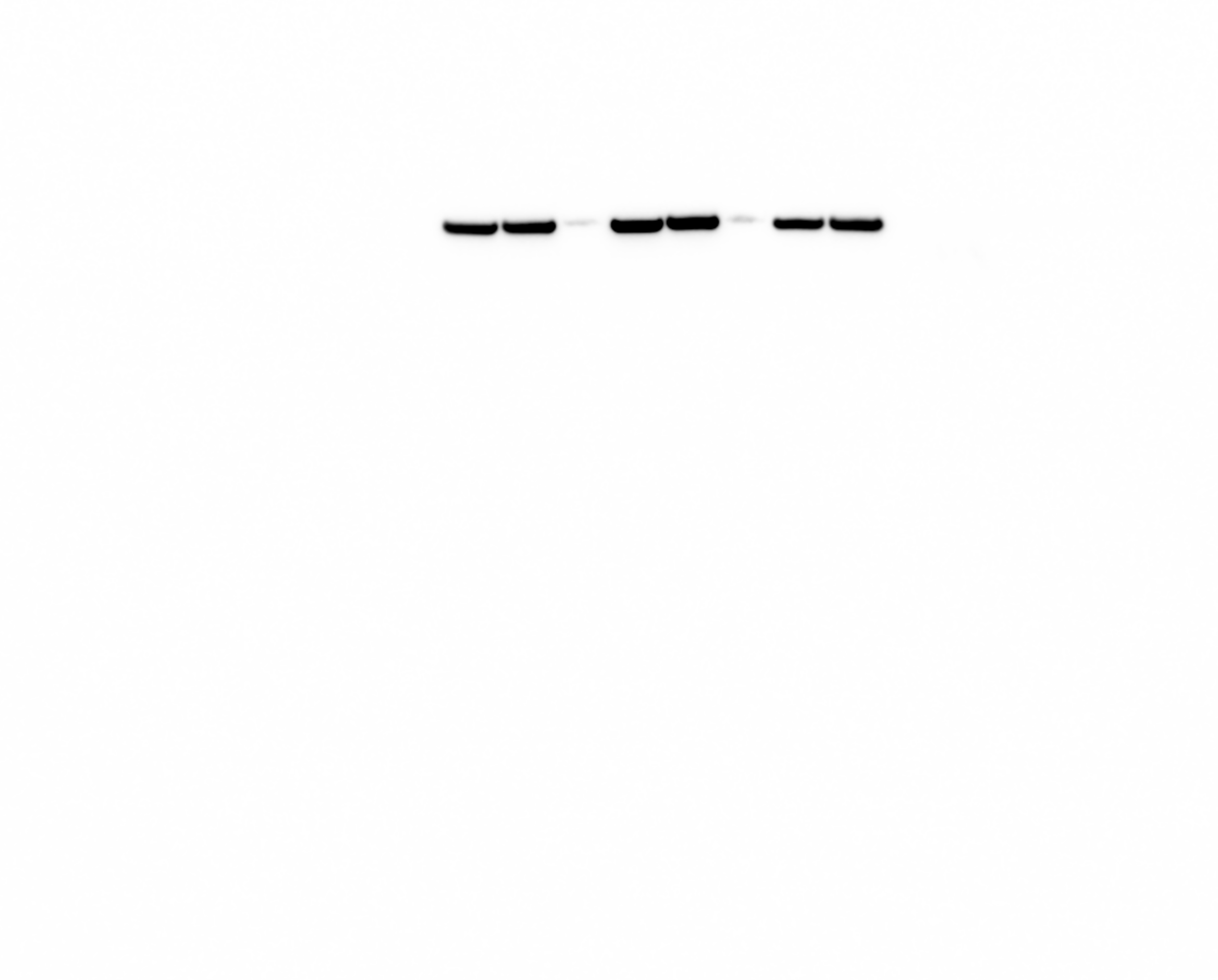

Supplement: Supplementary file 26 — Unprocessed western blots for Extended Data Fig. 4a,b. [file 42255_2025_1225_MOESM26_ESM.zip › Zuhra_WesternBlot_Extended_Fig4/Zuhra_WesternBlot_Extended_Fig4_c/PRDX6/FigE4g_PRDX6_Experiment1-2-3_actin.jpg]

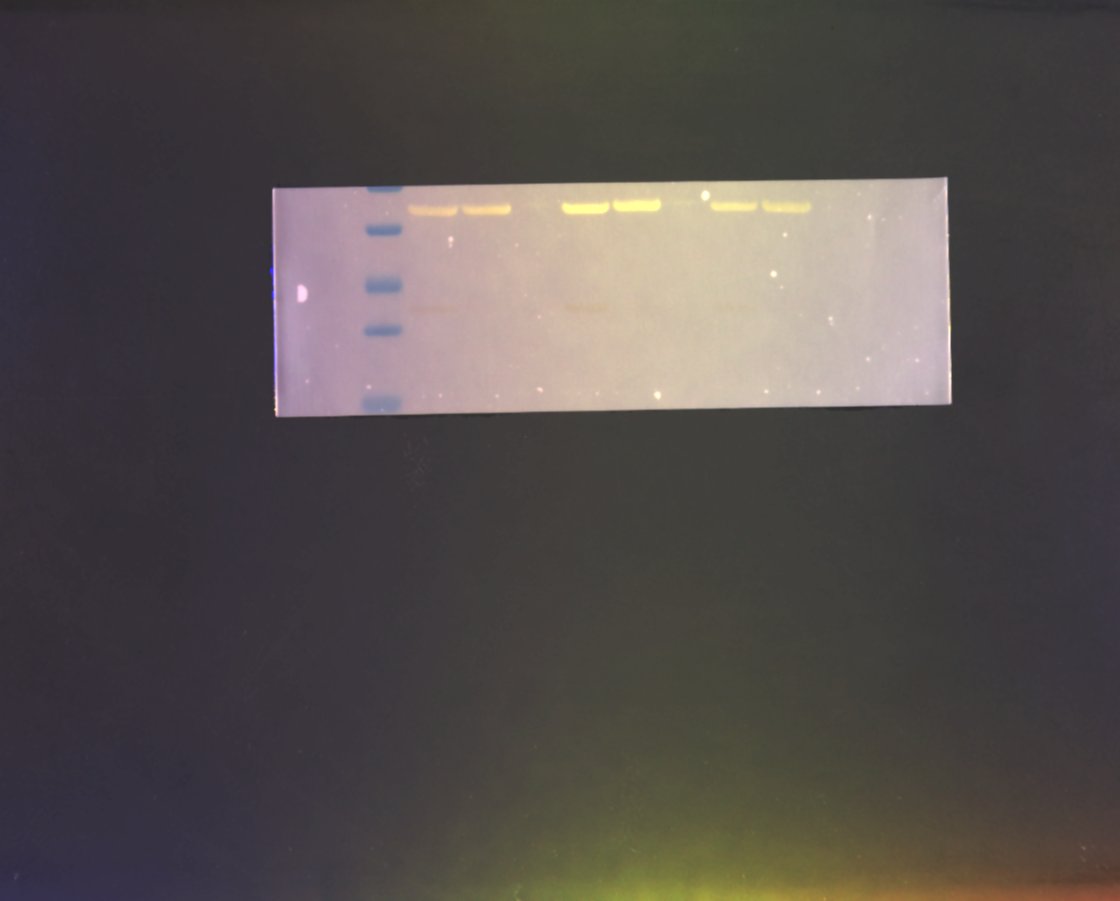

Supplement: Supplementary file 26 — Unprocessed western blots for Extended Data Fig. 4a,b. [file 42255_2025_1225_MOESM26_ESM.zip › Zuhra_WesternBlot_Extended_Fig4/Zuhra_WesternBlot_Extended_Fig4_c/PRDX6/FigE4g_PRDX6_Experiment1-2-3_actin_marker.jpg]

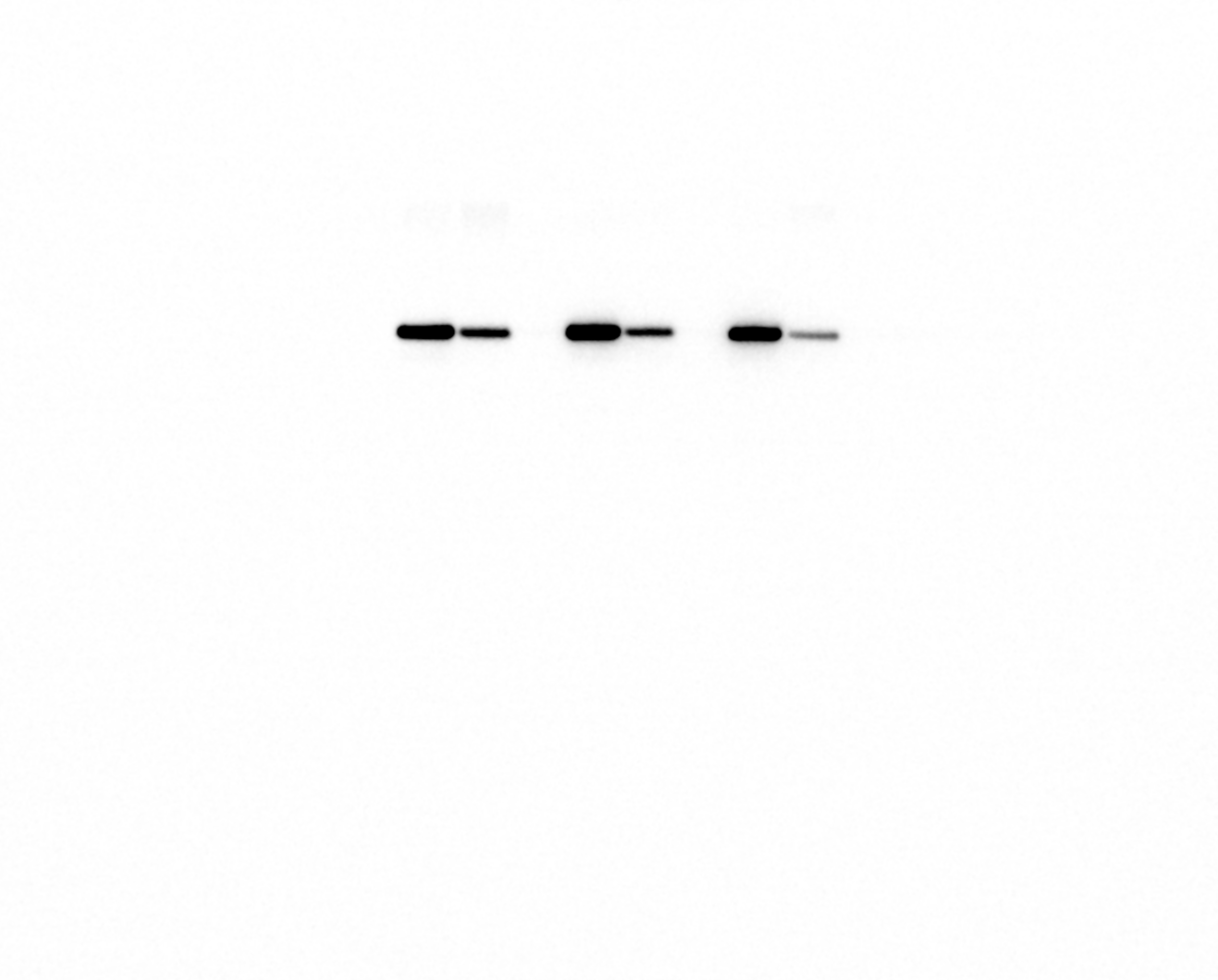

Supplement: Supplementary file 26 — Unprocessed western blots for Extended Data Fig. 4a,b. [file 42255_2025_1225_MOESM26_ESM.zip › Zuhra_WesternBlot_Extended_Fig4/Zuhra_WesternBlot_Extended_Fig4_c/PRDX6/FigE4g_PRDX6_Experiment1-2-3_PRDX6.jpg]

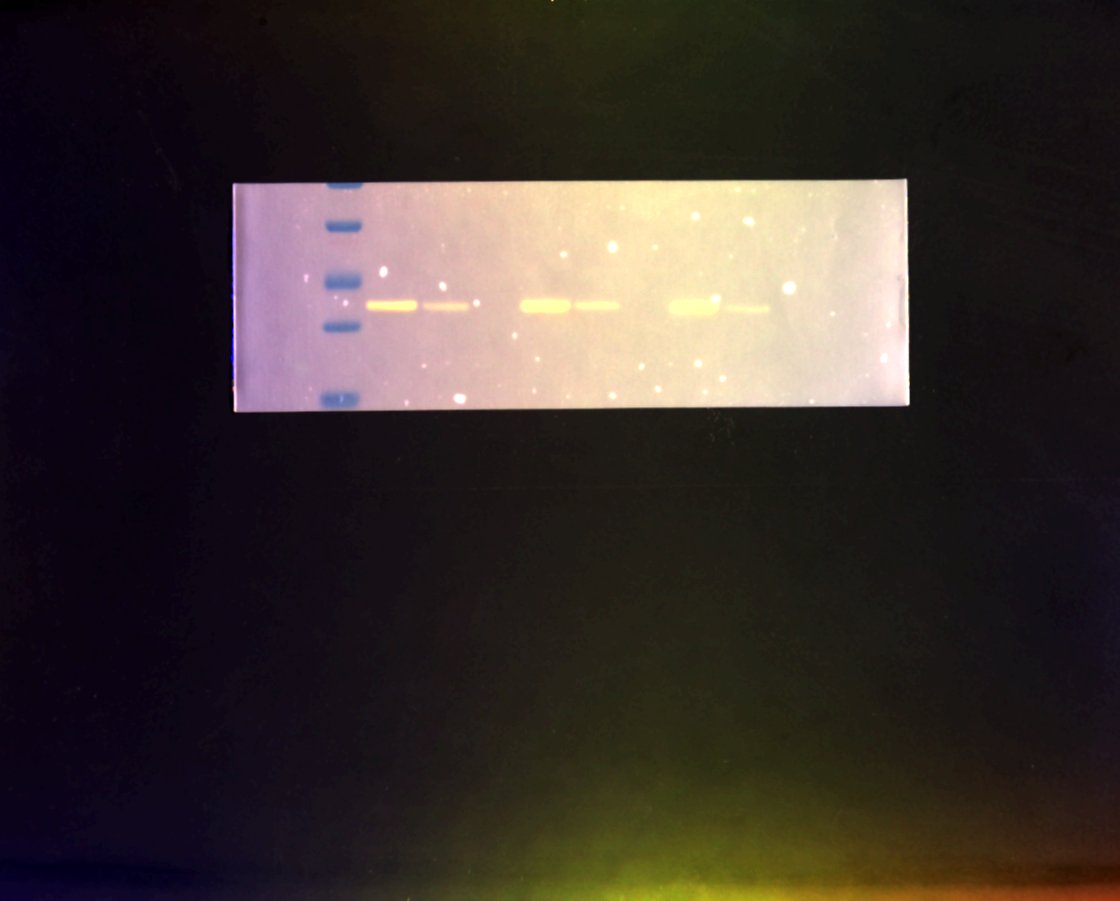

Supplement: Supplementary file 26 — Unprocessed western blots for Extended Data Fig. 4a,b. [file 42255_2025_1225_MOESM26_ESM.zip › Zuhra_WesternBlot_Extended_Fig4/Zuhra_WesternBlot_Extended_Fig4_c/PRDX6/FigE4g_PRDX6_Experiment1-2-3_PRDX6_marker.tif]

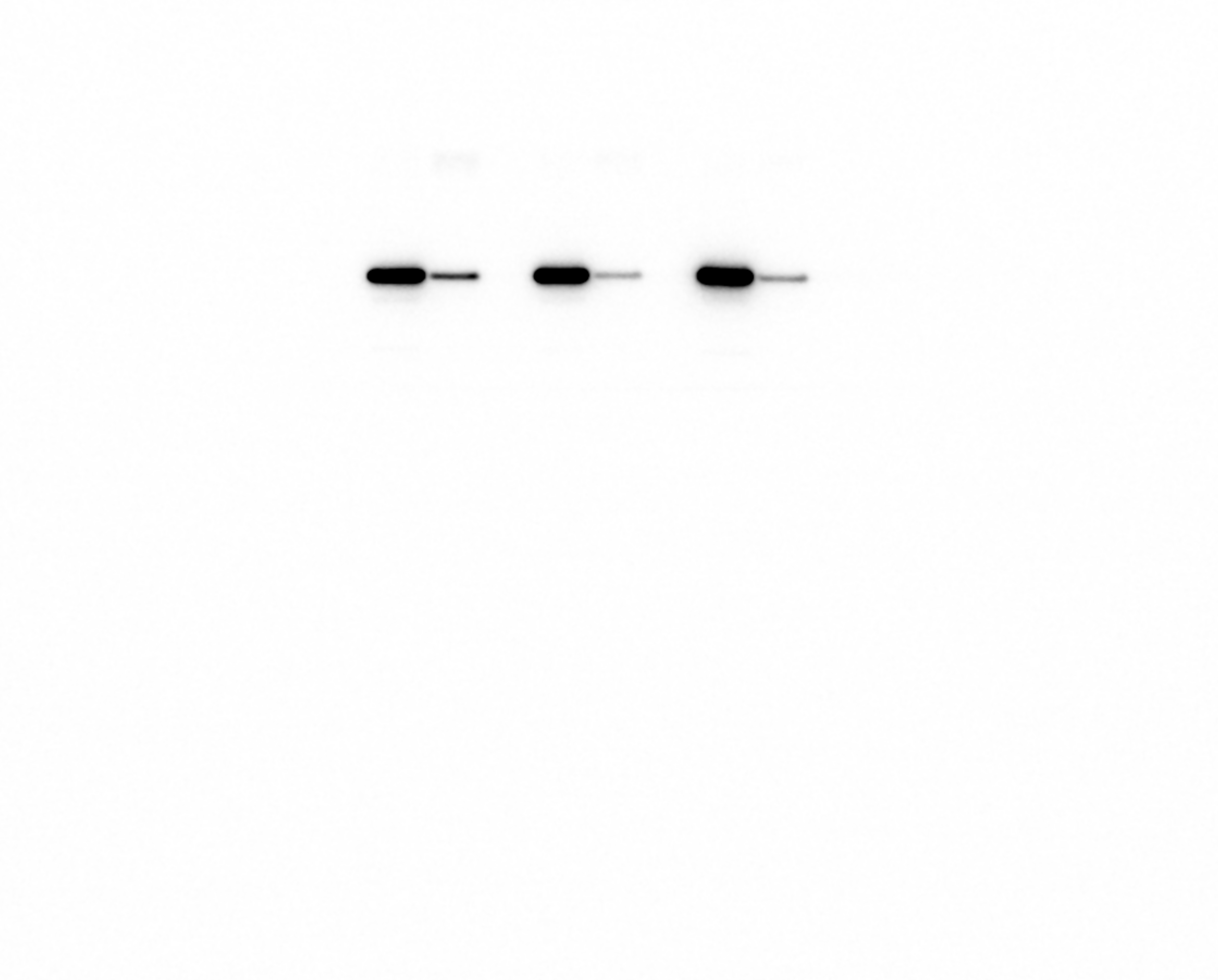

Supplement: Supplementary file 26 — Unprocessed western blots for Extended Data Fig. 4a,b. [file 42255_2025_1225_MOESM26_ESM.zip › Zuhra_WesternBlot_Extended_Fig4/Zuhra_WesternBlot_Extended_Fig4_c/PRDX6/FigE4g_PRDX6_Experiment4-5-6_PRDX6.jpg]

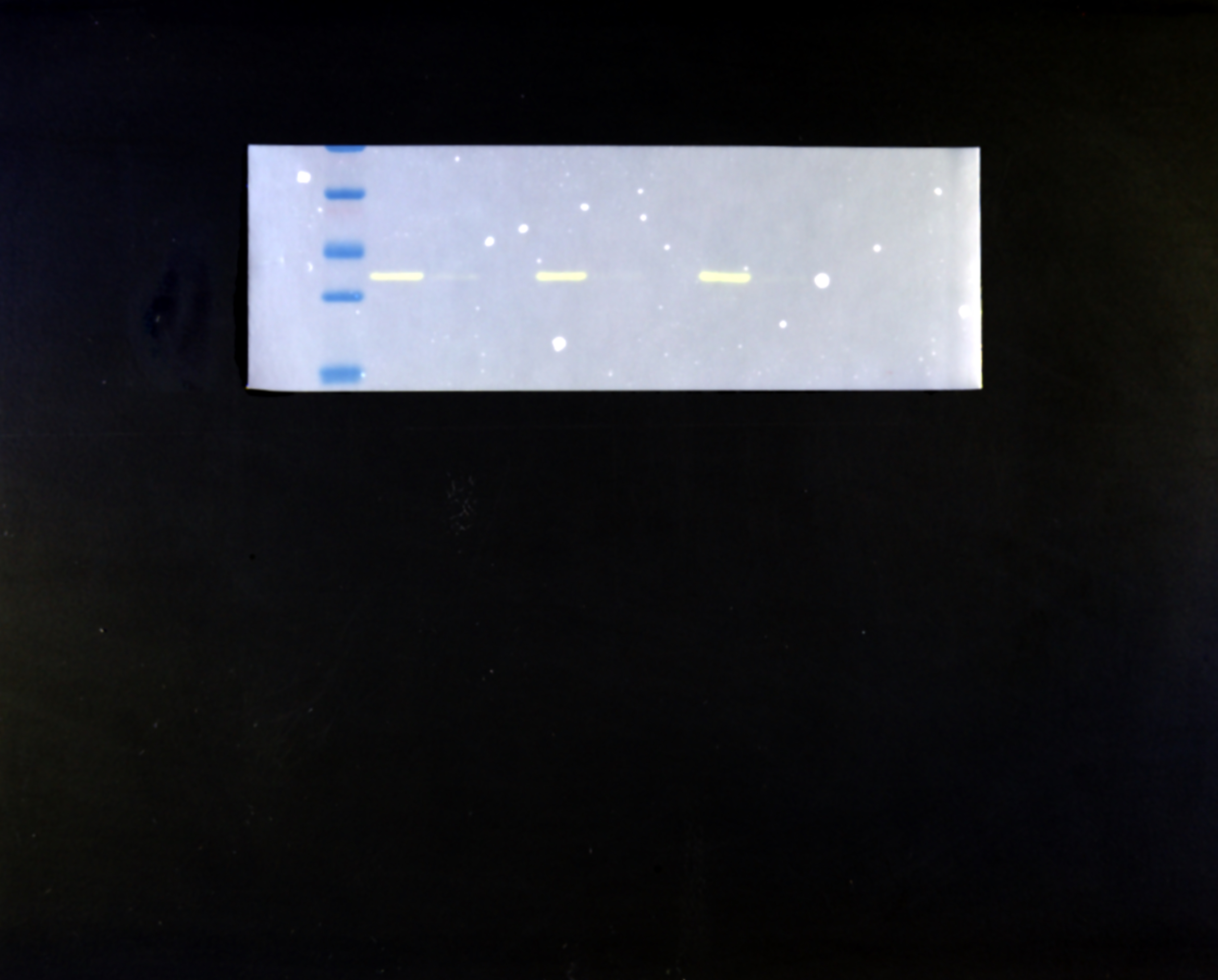

Supplement: Supplementary file 26 — Unprocessed western blots for Extended Data Fig. 4a,b. [file 42255_2025_1225_MOESM26_ESM.zip › Zuhra_WesternBlot_Extended_Fig4/Zuhra_WesternBlot_Extended_Fig4_c/PRDX6/FigE4g_PRDX6_Experiment4-5-6_PRDX6_marker.jpg]
